# Supplementary material for: Chalcogen Derivatives for the Treatment of African Trypanosomiasis: Biological Evaluation of Thio- and Seleno-Semicarbazones and Their Azole Derivatives
Source: ACS Omega. 2025 Jun 5;10(23):24872–86. doi: 10.1021/acsomega.5c02014 (PMC12177781; doi:10.1021/acsomega.5c02014)
Supplement: Supplementary file 1 [file ao5c02014_si_001.pdf]

SUPPLEMENTARY MATERIAL

Chalcogen derivatives for the treatment of African trypanosomiasis: biological evaluation of thio and seleno- semicarbazones and their azole derivatives

Mercedes Rubio-Hernández,[†,‡] Thaiz R. Teixeira,[§] Tina P. Nguyen,[§] Mai Shingyoji,[§] Elany Barbosa da Silva,[§] Anthony J. O'Donoghue,[§] Conor R. Caffrey,[*,§] Silvia Pérez-Silanes[*,†,‡] and Nuria Martínez-Sáez [*,‡]

[†] ISTUN Institute of Tropical Health, Department of Pharmaceutical Sciences, Universidad de Navarra, 31008 Pamplona, Spain.

[‡] Department of Pharmaceutical Sciences, Universidad de Navarra, 31008 Pamplona, Spain.

[§] Center for Discovery and Innovation in Parasitic Diseases, Skaggs School of Pharmacy and Pharmaceutical Sciences, University of California San Diego, La Jolla, California 92093, United States.

***E-mail: sperez@unav.es.**

***E-mail: nmartinezsa@unav.es.**

***E-mail: ccaffrey@health.ucsd.edu.**

Contents:

1. Biological data (**Table S1**)
2. Inhibition of *Tbr*CATL and *h*CatL by selected *S*-compounds (**Figure S1**)
3. Molecular Dynamics data (**Table S2**)
4. DPPH data (**Table S3**)
5. *In silico* ADME and drug-likeness predictions (**Table S4**)
6. Chemical data (IR, ^{1}H-NMR, ^{13}C-NMR, ^{77}Se-NMR and qNMR spectra of final compounds) (**Figures S2-S95**)
7. Bidimensional spectra of compounds ***S*O7** and ***Se*O7 (Figures S96** and **S97)**
8. Comparative SARs for inhibition of *T. brucei* and *T. cruzi* growth, and the inhibition of their respective proteases, *Tbr*CATL and Cz (**Table S5**)

1. Biological data

Table S1. Data for the 44 compounds screened against *T. brucei*, HEK293 and HepG2 cells.

Structure	Compound	*T. brucei* inhibition (%)[a]	HEK293 inhibition (%)[b]	HepG2 inhibition (%)[c]	EC_{50} *T. brucei* (μM)[d]	CC_{50} HEK293 (μM)[e]	SI_{HEK293} [f]	CC_{50} HepG2 (μM)[g]	SI_{HepG2} [h]
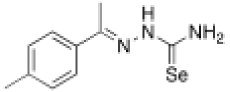	***Se*O1**	100 ± 1	100 ± 1	80 ± 1	0.98 ± 0.12	4.27 ± 0.22	4	4.17 ± 0.60	4
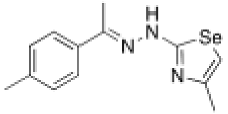	***Se*C1** [i]	72 ± 6	77 ± 2	65 ± 9	9.80 ± 0.63	11.97 ± 4.94	1	10.98 ± 0.88	1
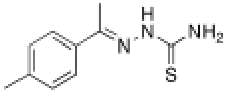	**SO1**	44 ± 7	30 ± 3	50 ± 4	ND	ND	ND	ND	ND
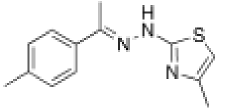	**SC1** [i]	30 ± 6	24 ± 7	4 ± 15	ND	ND	ND	ND	ND
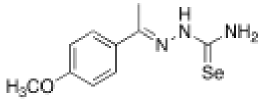	***Se*O2**	100 ± 3	100 ± 2	78 ± 3	2.36 ± 0.02	5.81 ± 0.11	2	4.12 ± 0.44	2
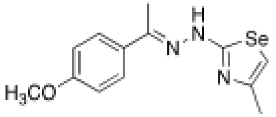	***Se*C2** [i]	23 ± 7	80 ± 6	40 ± 5	ND	ND	ND	ND	ND
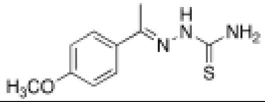	**SO2**	6 ± 2	22 ± 7	34 ± 9	ND	ND	ND	ND	ND

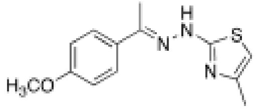	**SC2** [i]	27 ± 5	33 ± 8	6 ± 3	ND	ND	ND	ND	ND
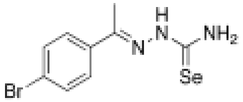	***Se*O3**	100 ± 2	100 ± 0	71 ± 15	0.47 ± 0.02	2.82 ± 0.11	6	2.70 ± 0.16	6
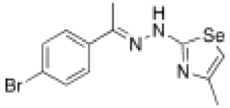	***Se*C3** [i]	99 ± 2	88 ± 3	80 ± 2	6.04 ± 0.72	5.90 ± 1.22	1	4.68 ± 0.14	1
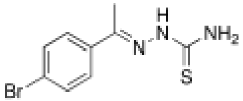	**SO3**	28 ± 4	76 ± 2	65 ± 4	ND	ND	ND	ND	ND
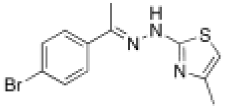	**SC3** [i]	38 ± 9	64 ± 2	46 ± 7	ND	ND	ND	ND	ND
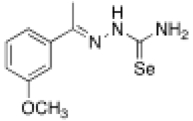	***Se*O4**	100 ± 1	100 ± 2	74 ± 4	3.28 ± 0.29	3.25 ± 0.30	1	4.18 ± 0.25	1
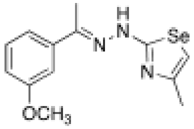	***Se*C4** [i]	36 ± 4	60 ± 7	66 ± 5	ND	ND	ND	ND	ND
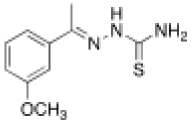	**SO4**	29 ± 5	31 ± 4	28 ± 6	ND	ND	ND	ND	ND

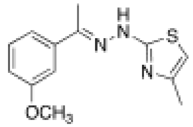	**SC4** [i]	54 ± 5	40 ± 4	52 ± 3	ND	ND	ND	ND	ND
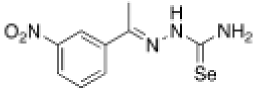	***Se*O5**	100 ± 1	100 ± 0	85 ± 0	5.83 ± 0.72	2.29 ± 0.26	0	5.54 ± 1.47	1
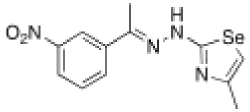	***Se*C5** [i]	100 ± 2	81 ± 7	77 ± 6	10.53 ± 1.02	9.00 ± 1.36	1	11.27 ± 0.97	1
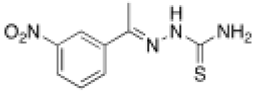	**SO5**	31 ± 5	14 ± 9	39 ± 9	ND	ND	ND	ND	ND
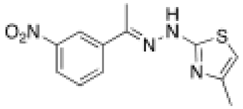	**SC5** [i]	40 ± 8	30 ± 3	17 ± 2	ND	ND	ND	ND	ND
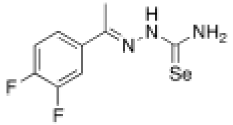	***Se*O6**	100 ± 0	100 ± 0	75 ± 1	2.57 ± 0.15	3.69 ± 0.37	2	4.43 ± 0.11	2
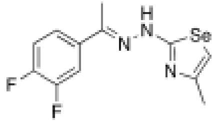	***Se*C6** [i]	37 ± 4	48 ± 9	55 ± 5	ND	ND	ND	ND	ND
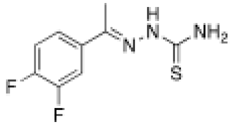	**SO6**	44 ± 4	36 ± 5	36 ± 1	ND	ND	ND	ND	ND

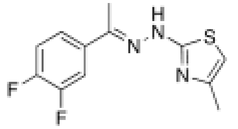	**SC6** [i]	22 ± 3	35 ± 1	0 ± 7	ND	ND	ND	ND	ND
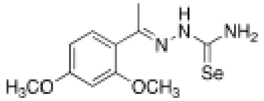	***Se*O7**	12 ± 1	99 ± 2	2 ± 3	ND	ND	ND	ND	ND
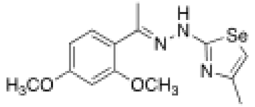	***Se*C7** [i]	15 ± 4	38 ± 7	4 ± 6	ND	ND	ND	ND	ND
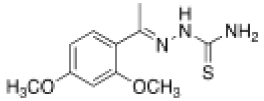	**SO7**	34 ± 5	13 ± 3	1 ± 1	ND	ND	ND	ND	ND
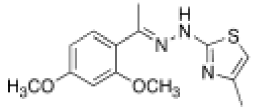	**SC7** [i]	39 ± 8	23 ± 1	0 ± 7	ND	ND	ND	ND	ND
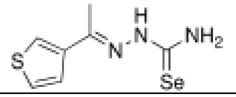	***Se*O8**	11 ± 1	100 ± 1	62 ± 2	ND	ND	ND	ND	ND
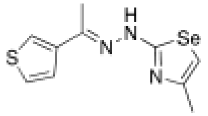	***Se*C8** [i]	25 ± 6	37 ± 3	22 ± 3	ND	ND	ND	ND	ND
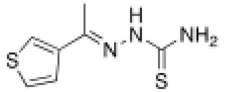	**SO8**	23 ± 2	13 ± 1	0 ± 6	ND	ND	ND	ND	ND
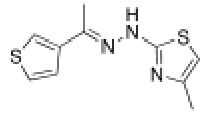	**SC8** [i]	0 ± 1	28 ± 1	0 ± 6	ND	ND	ND	ND	ND

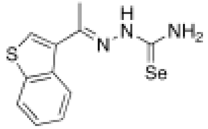	***Se*O9**	100 ± 1	100 ± 0	67 ± 5	4.80 ± 0.33	3.46 ± 0.12	1	4.93 ± 0.41	1
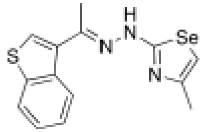	***Se*C9** [i]	58 ± 1	73 ± 9	85 ± 5	ND	ND	ND	ND	ND
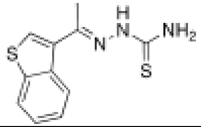	**SO9**	18 ± 6	58 ± 6	17 ± 5	ND	ND	ND	ND	ND
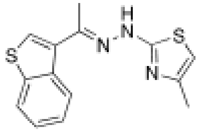	**SC9** [i]	39 ± 7	55 ± 7	9 ± 10	ND	ND	ND	ND	ND
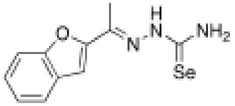	***Se*O10**	100 ± 1	100 ± 1	82 ± 2	1.86 ± 0.33	3.00 ± 0.15	2	5.08 ± 0.27	3
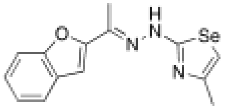	***Se*C10** [i]	51 ± 7	65 ± 4	69 ± 10	ND	ND	ND	ND	ND
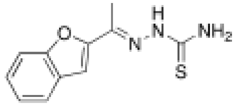	**SO10**	0 ± 8	28 ± 3	54 ± 5	ND	ND	ND	ND	ND
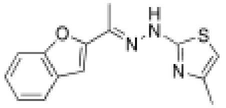	**SC10** [i]	46 ± 6	42 ± 0	49 ± 9	ND	ND	ND	ND	ND

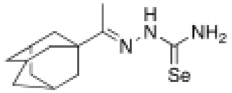	***Se*O11**	100 ± 1	100 ± 4	66 ± 5	5.16 ± 1.06	10.50 ± 0.36	2	6.01 ± 0.69	1
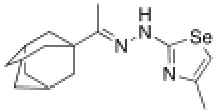	***Se*C11** [i]	37 ± 0	64 ± 10	66 ± 2	ND	ND	ND	ND	ND
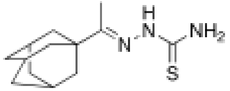	**SO11**	0 ± 4	75 ± 0	0 ± 12	ND	ND	ND	ND	ND
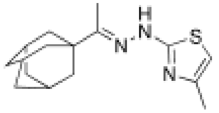	**SC11** [i]	19 ± 8	39 ± 3	0 ± 13	ND	ND	ND	ND	ND
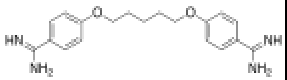	**Pentamidine**	100 ± 2	NA		0.014 ± 0.002	NA			
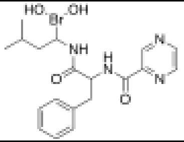	**Bortezomib**	ND	100 ± 2	100 ±1	ND	0.015 ± 0.002		0.015 ± 0.003	

[a]*T. brucei* growth inhibition was calculated at 10 µM and is shown as a percentage ± SD. [b]HEK293 growth inhibition was calculated at 10 µM and is shown as percentage ± SD. [c]HepG2 growth inhibition was calculated at 10 µM and is shown as percentage ± SD. [d]EC_{50} values (mean ± SD) were calculated from three independent experiments, each performed in duplicate. [e]CC_{50} HEK293 (µM) values (mean ± SD) were calculated from three independent experiments, each performed in duplicate. [f]SI is the selectivity index of the compound HEK293 CC_{50}/EC_{50}. [g]CC_{50} HepG2 (µM) values (mean ± SD) were calculated from three independent experiments, each performed in duplicate. [h]SI is the selectivity index of the compound HepG2 CC_{50}/EC_{50}. [i]These compounds were tested in their hydrochloride form.
ND: no data.

2. Inhibition of *Tbr*CATL and *h*CatL by selected *S*-compounds

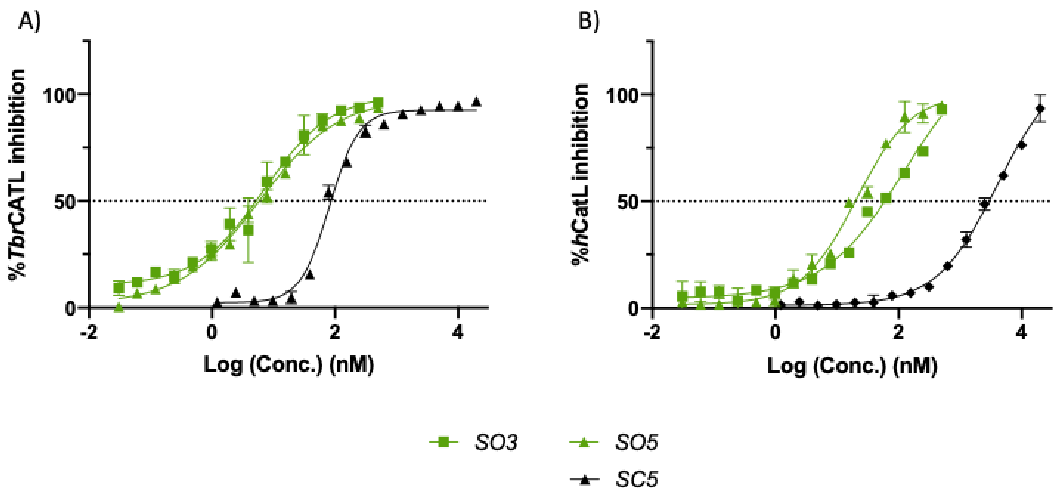

Figure S1. Inhibition of (**A**) *Tbr*CATL and (**B**) *h*CatL by *S*-compounds.

3. Molecular Dynamics data

Table S2. Average main interaction distances between the ligands and the protein as complexes, and their occurrence during the MD simulation trajectories.

Ligand	Type of interaction	Involved Atoms	Distance (Å)	Occurrence (%)
***Se*O1**	H bond	Asp 161 (O)- Ligand N2 (H3)	2.8	49
	H bond	Gly 66 (O)-Ligand N2 (H3)	2.8	14
	H bond	Gly 66 (O)-Ligand N1 (H1)	2.9	26
	H bond	Gly 62 (O)-Ligand N1 (H2)	2.9	36
	CH-π	Leu 67 (Cδ2)- phenyl ring	3.7	48
	CH-π	Ala 138 (Cβ)- phenyl ring	3.6	5
***Se*O3**	H bond	Asp 161 (O)- Ligand N1 (H1)	2.8	10
	H bond	Gly 66 (O)-Ligand N2 (H3)	2.9	10
	H bond	Gly 66 (O)-Ligand N1 (H1)	2.8	5
	CH-π	Leu 67 (Cδ2)- phenyl ring	3.7	48
	CH-π	Ala 138 (Cβ)- phenyl ring	3.7	51
***Se*O5**	CH-π	Leu67 (Cδ2)- phenyl ring	3.8	10
	CH-π	Ala 138 (Cβ)- phenyl ring	3.8	19
	Π-Hole	Gly66 (O)-Ligand N4	3.2	11
	Π-Hole	His162(O)-Ligand N4	3.4	2
***Se*C3**	H bond	Leu 160 (NH)-Ligand N1	2.9	5
	H bond	Leu 160 (O)-Ligand N2(H)	2.9	5
	H bond	Gln 159 (Oε)-Ligand N2(H)	2.9	1
	CH-π	Leu67 (Cδ2)- phenyl ring	3.7	15
	CH-π	Ala 138 (Cβ)- phenyl ring	3.7	18
***Se*C5**	CH-π	Leu67 (Cδ2)- phenyl ring	3.7	24
	CH-π	Ala 138 (Cβ)- phenyl ring	3.8	13
	Π-Hole	Gly66 (O)-Ligand N4	3.2	29
	Π-Hole	His162(O)-Ligand N4	3.3	5.2

4. DPPH data

Table S3. DPPH data for three compound concentrations after 2 h.

Compound	0.06 mg/ml[a]	0.03 mg/ml[a]	0.015 mg/ml[a]
***Se*O1**	77.33 ± 1.76	78.00 ± 1.16	45.67 ± 9.56
***Se*C1**	72.33 ± 2.33	71.67 ± 2.40	71.67 ± 2.85
***S*O1**	45.00 ± 8.33	NT	NT
***S*C1**	60.00 ± 4.16	54.33 ± 1.86	55.00 ± 1.53
***Se*O3**	77.67 ± 1.45	75.00 ± 2.65	23.00 ± 13.43
***Se*C3**	75.67 ± 1.45	71.00 ± 3.06	35.00 ± 18.82
***S*O3**	29.33 ± 24.77	NT	NT
***S*C3**	67.00 ± 2.31	69.33 ± 2.33	68.00 ± 3.06
***Se*O5**	75.67 ± 1.67	63.33 ± 6.89	22.00 ± 17.67
***Se*C5**	78.33 ± 1.76	78.00 ± 1.73	77.33 ± 0.88
***S*O5**	23.33 ± 19.46	NT	NT
***S*C5**	66.67 ± 1.86	68.67 ± 2.03	66.33 ± 1.86
**ASC[b]**	80.33 ± 0.67	80.33 ± 0.33	76.00 ± 3.52
**TRO[b]**	79.67 ± 0.67	79.33 ± 0.882	79.33 ± 1.33

[a]mg/ml (expressed as the mean ± SEM of three independent experiments, each performed in triplicate). [b]ASC: ascorbic acid; TRO: Trolox. Both compounds were used as positive controls. NT: not tested.

5. ***In silico* predictions *of* ADME and drug-likeness properties**

Table S4. ADME and drug-likeness properties predicted for 44 compounds using SwissADME (http://www.swissadme.ch/)

Compound	MW[a]	Log P[b]	Solubility[c]	Drug elimination parameters					Drug-likeness			
				CYP1A2 inhibitor	CYP2C19 inhibitor	CYP2C9 inhibitor	CYP2D6 inhibitor	CYP3A4 inhibitor	Lipinski violations	Ghose violations	Veber violations	Egan violations
***Se*O1**	254.19	0.85	Soluble	No	No	No	No	No	0	0	0	0
***Se*C1**	328.70	1.99	Moderately soluble	No	No	No	No	No	0	0	0	0
***S*O1**	207.30	1.89	Soluble	No	No	No	No	No	0	0	0	0
***S*C1**	281.80	3.16	Moderately soluble	Yes	No	No	No	No	0	0	0	0
***Se*O2**	270.19	0.50	Soluble	No	No	No	No	No	0	0	0	0
***Se*C2**	344.70	1.64	Moderately soluble	No	No	No	No	No	0	0	0	0
***S*O2**	223.29	1.67	Soluble	Yes	No	No	No	No	0	0	0	0
***S*C2**	297.80	2.82	Moderately soluble	Yes	No	No	No	No	0	0	0	0
***Se*O3**	319.06	1.13	Soluble	No	No	No	No	No	0	0	0	0
***Se*C3**	393.57	2.26	Moderately soluble	No	No	No	No	No	0	0	0	0
***S*O3**	272.16	2.19	Soluble	Yes	No	No	No	No	0	0	0	0
***S*C3**	346.67	3.44	Moderately soluble	Yes	No	No	No	No	0	0	0	0
***Se*O4**	270.19	0.50	Soluble	No	No	No	No	No	0	0	0	0

***Se*C4**	344.70	1.64	Moderately soluble	No	No	No	No	No	0	0	0	0
***S*O4**	223.29	1.67	Soluble	Yes	No	No	No	No	0	0	0	0
***S*C4**	297.80	2.82	Moderately soluble	Yes	No	No	No	No	0	0	0	0
***Se*O5**	285.16	-0.12	Soluble	No	No	No	No	No	0	0	0	0
***Se*C5**	359.67	1.01	Moderately soluble	Yes	No	No	No	No	0	0	0	0
***S*O5**	238.27	0.91	Soluble	Yes	No	No	No	No	0	0	0	0
***S*C5**	312.78	2.19	Moderately soluble	Yes	Yes	No	No	No	0	0	0	0
***Se*O6**	276.14	1.16	Soluble	No	No	No	No	No	0	0	0	0
***Se*C6**	350.65	2.29	Moderately soluble	No	No	No	No	No	0	0	0	0
***S*O6**	229.25	2.29	Soluble	Yes	No	No	No	No	0	0	0	0
***S*C6**	303.76	3.47	Moderately soluble	No	No	No	No	No	0	0	0	0
***Se*O7**	300.22	0.46	Soluble	No	No	No	No	No	0	0	0	0
***Se*C7**	374.72	1.59	Moderately soluble	No	No	No	Yes	No	0	0	0	0
***S*O7**	253.32	1.70	Soluble	Yes	No	No	No	No	0	0	0	0
***S*C7**	327.83	2.76	Moderately soluble	Yes	Yes	No	No	No	0	0	0	0
***Se*O8**	246.19	0.43	Very soluble	No	No	No	No	No	0	0	0	0

***Se*C8**	320.70	1.68	Soluble	No	No	No	No	No	0	0	0	0
***S*O8**	199.30	1.53	Very soluble	Yes	No	No	No	No	0	0	0	0
***S*C8**	273.81	2.86	Moderately soluble	No	No	No	No	No	0	0	0	0
***Se*O9**	296.25	1.41	Soluble	No	No	No	No	No	0	0	0	0
***Se*C9**	370.76	2.61	Moderately soluble	No	Yes	No	No	Yes	0	0	0	0
***S*O9**	249.36	2.63	Soluble	Yes	Yes	Yes	No	No	0	0	0	0
***S*C9**	323.86	3.79	Moderately soluble	Yes	Yes	Yes	No	No	0	0	0	0
***Se*O10**	280.18	0.85	Soluble	No	No	No	No	No	0	0	0	0
***Se*C10**	370.74	2.35	Moderately soluble	Yes	No	No	Yes	Yes	0	0	0	0
***S*O10**	233.29	2.03	Soluble	Yes	Yes	No	No	No	0	0	0	0
***S*C10**	307.80	3.22	Moderately soluble	Yes	Yes	No	No	No	0	0	0	0
***Se*O11**	298.29	1.32	Soluble	No	No	No	No	No	0	0	0	0
***Se*C11**	336.33	2.13	Soluble	No	No	No	Yes	No	0	0	0	0
***S*O11**	239.38	2.62	Soluble	No	No	Yes	No	No	0	0	0	0
***S*C11**	325.90	3.68	Moderately soluble	No	Yes	Yes	No	No	0	0	0	0
**Pentamidine**	340.42	2.72	Soluble	No	No	Yes	Yes	No	0	0	0	0
**Bortezomib**	453.33	1.26	Soluble	No	Yes	No	Yes	Yes	0	0	1	0

[a]MW: Molecular weight. [b]Log P: The partition coefficient between n-octanol and water (log $P_{o/w}$) is the classical descriptor for lipophilicity. It is expressed as the arithmetic mean of the values predicted by the five proposed methods as described in Daina, A., *et al.* (Sci. Rep. **7**, 42717 (2017)). [c]Solubility: calculated according to the Estimated SOLubility (ESOL) model by Delaney, J.S. (J. Chem. Inf. Model. 44, 1000–1005 (2004)).

6. Chemical data (IR, ^{1}H-NMR, ^{13}C-NMR, ^{77}Se-NMR and qNMR spectra of final compounds)

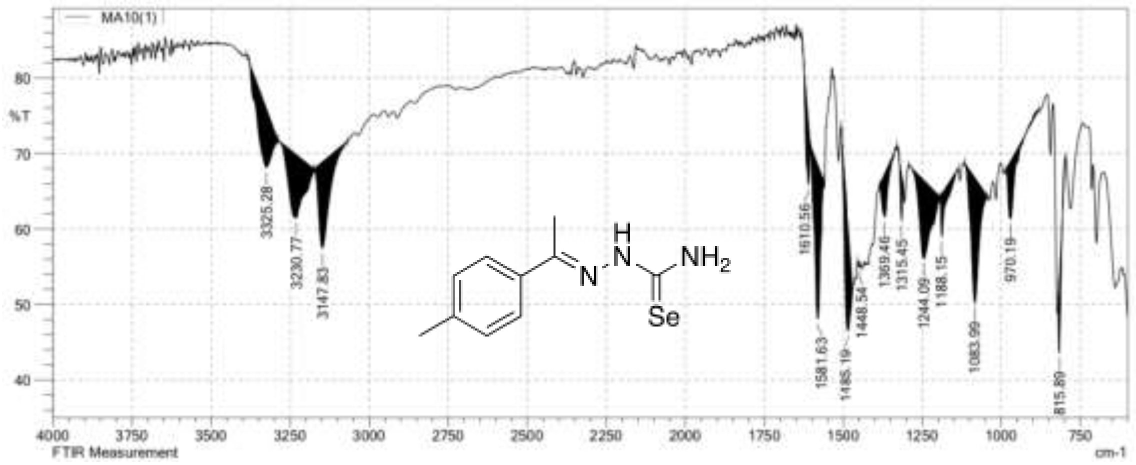

Figure S2. IR (up) and ^{1}H-NMR (down) of compound ***Se*O1**.

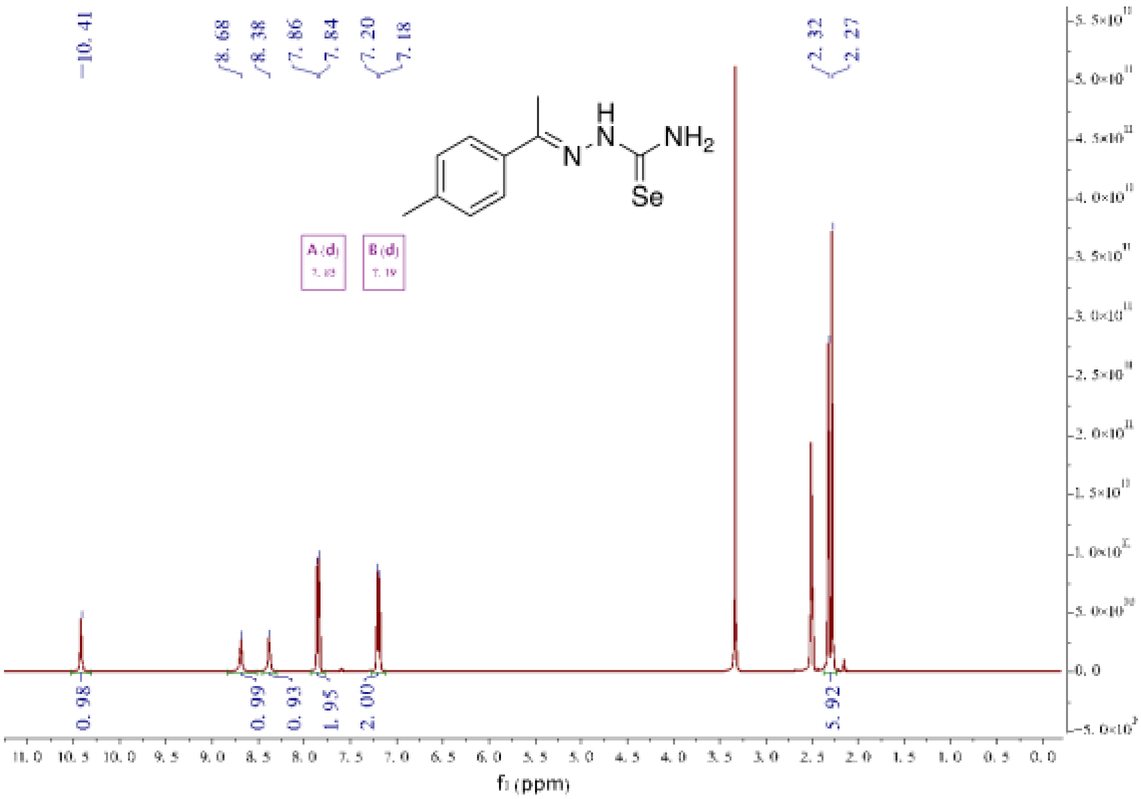

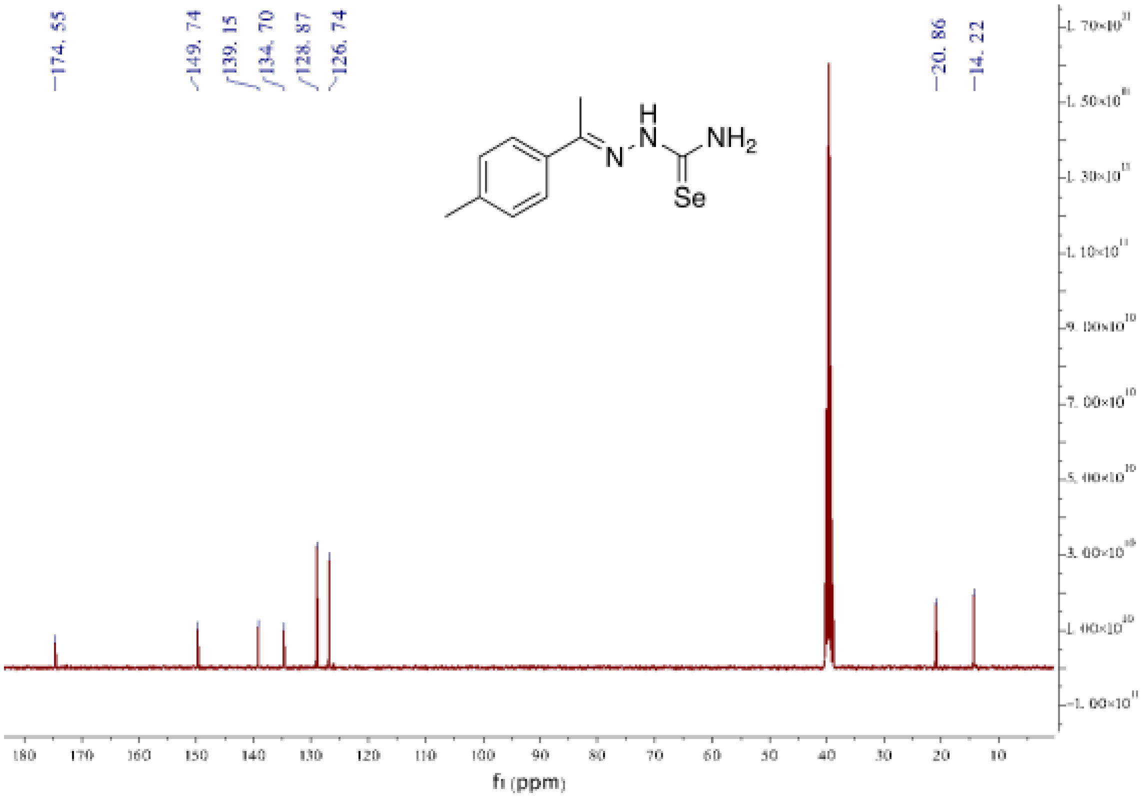

Figure S3. ^{13}C-NMR (up) and ^{77}Se-NMR (down) of compound ***Se*O1**.

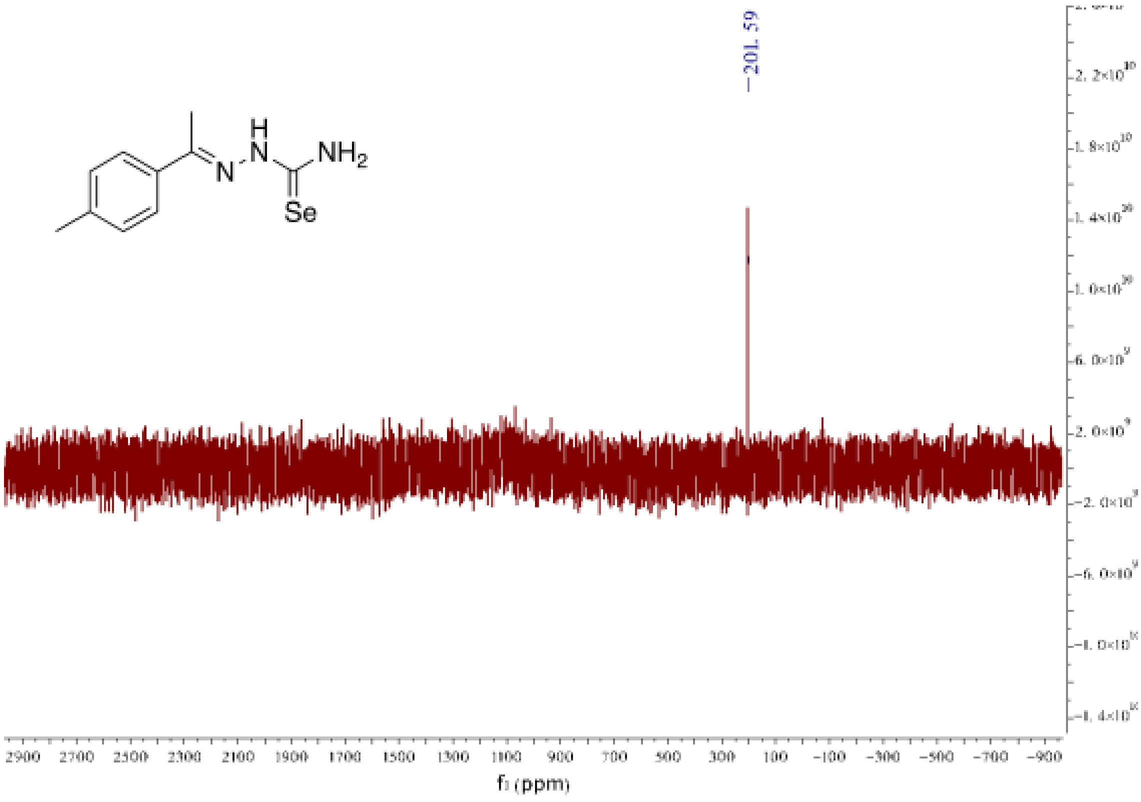

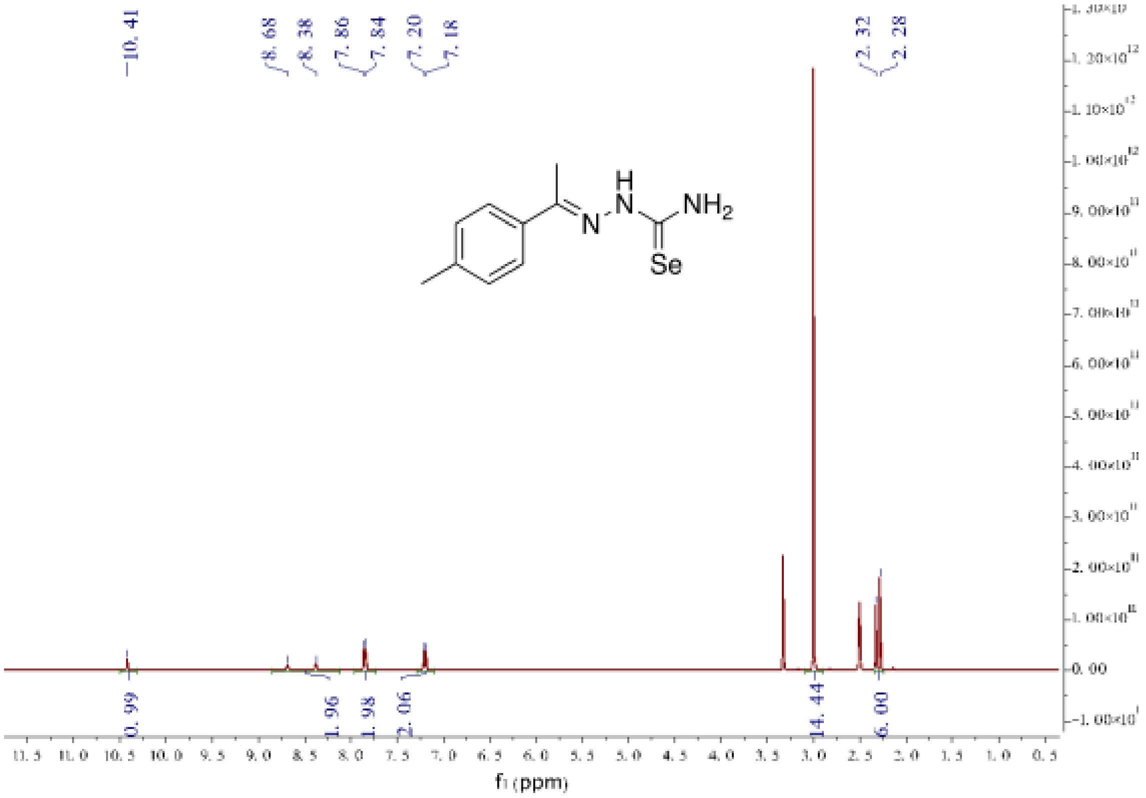

Figure S4. qNMR (up) of compound ***Se*O1** and IR (down) of compound ***Se*C1**.

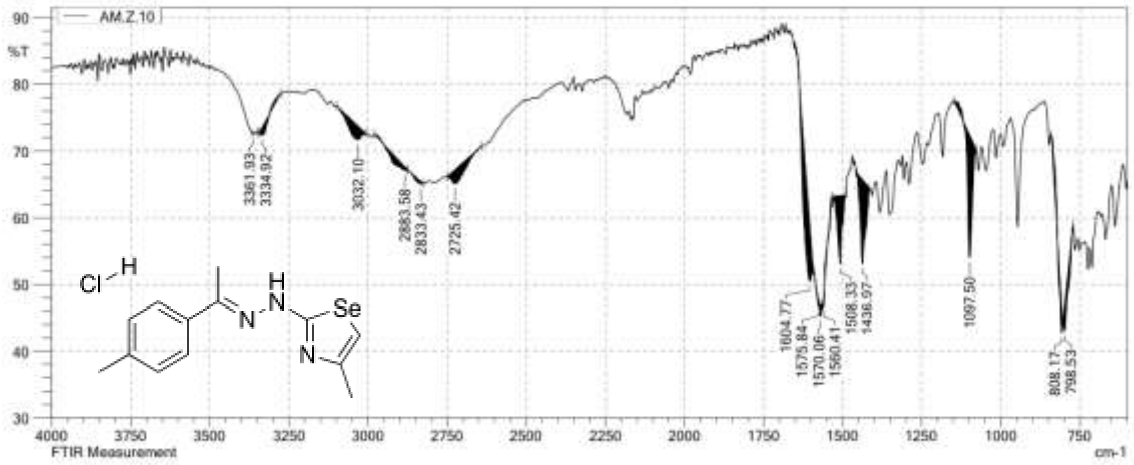

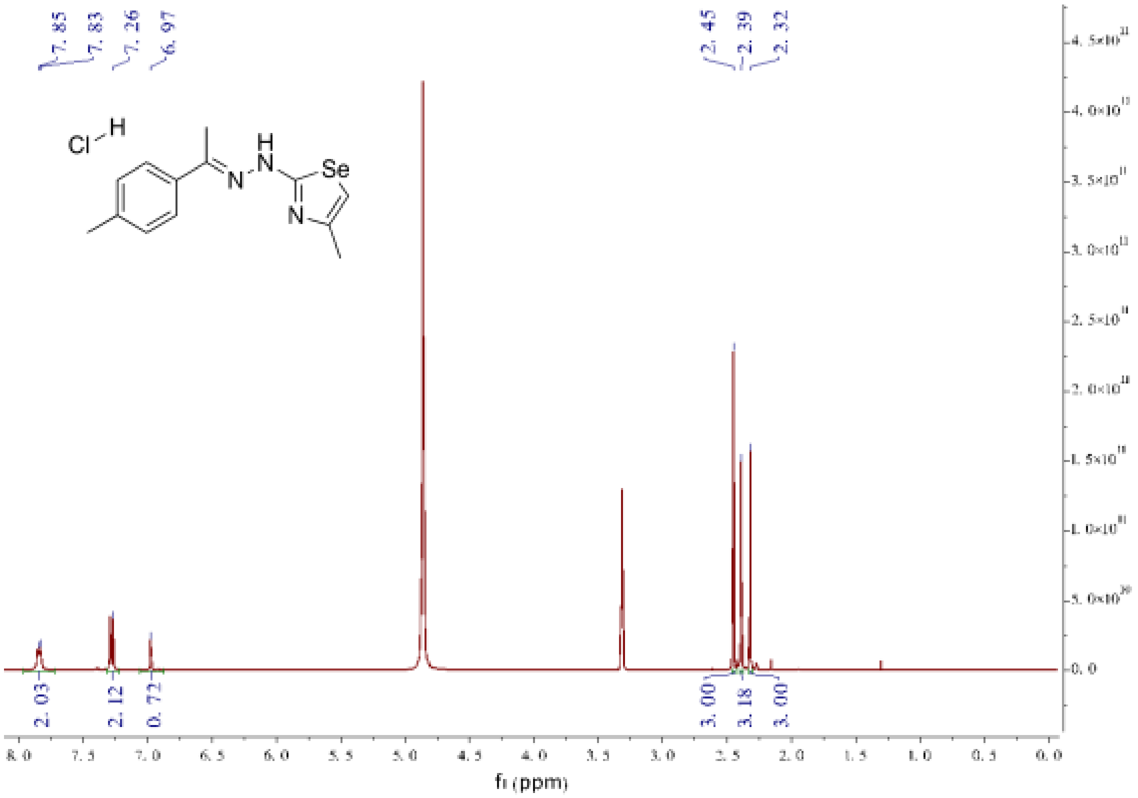

Figure S5. ^{1}H-NMR (up) and ^{13}C-NMR (down) and of compound ***Se*****C1**.

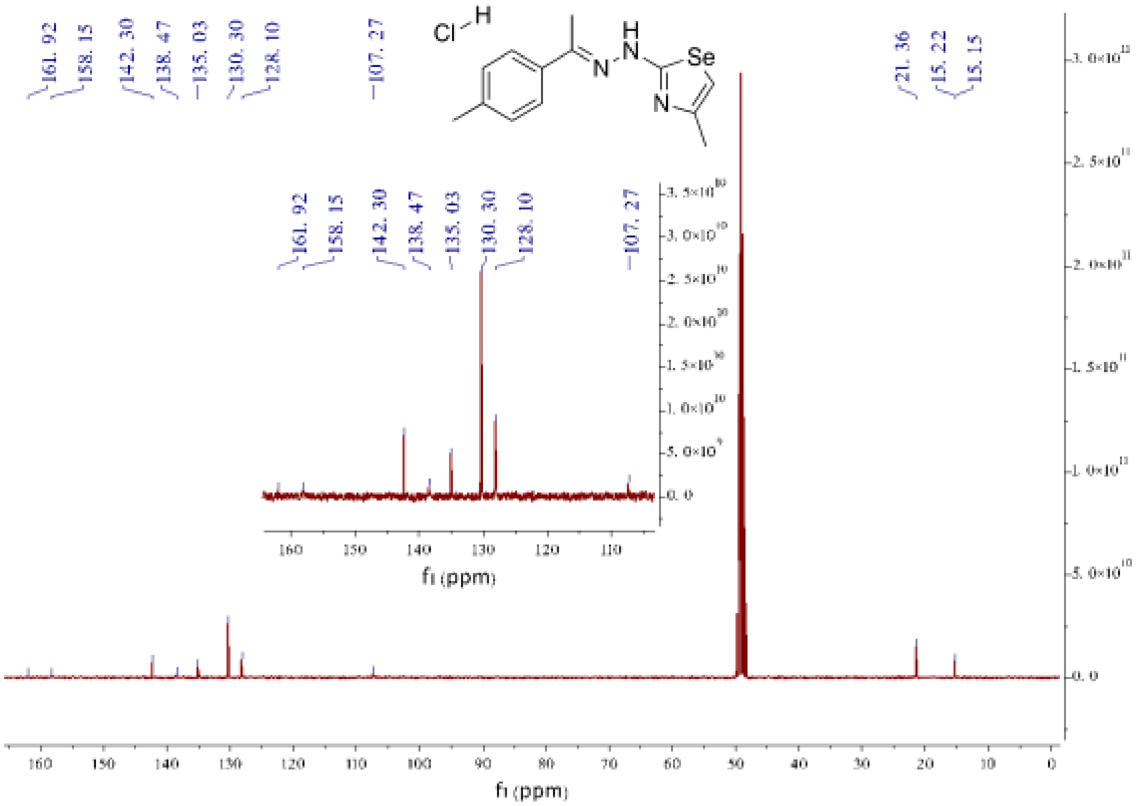

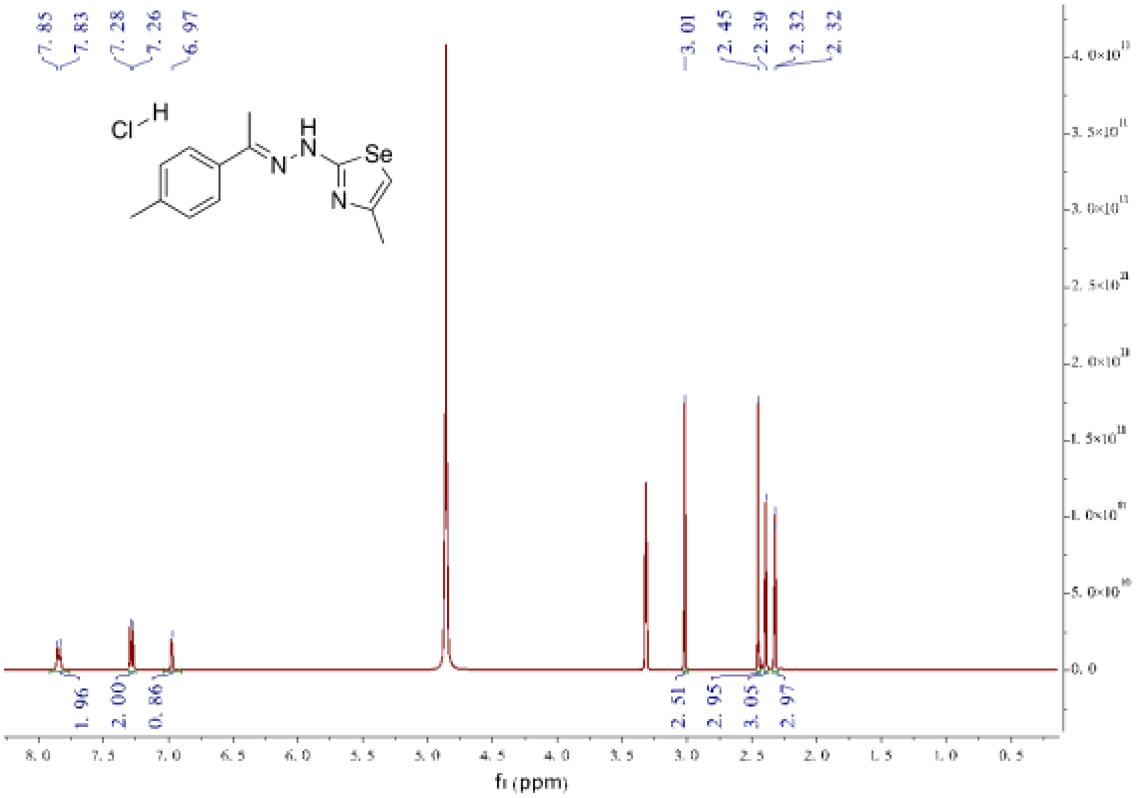

Figure S6. qNMR (up) of compound ***Se*C1** and IR (down) of compound ***S*O1**.

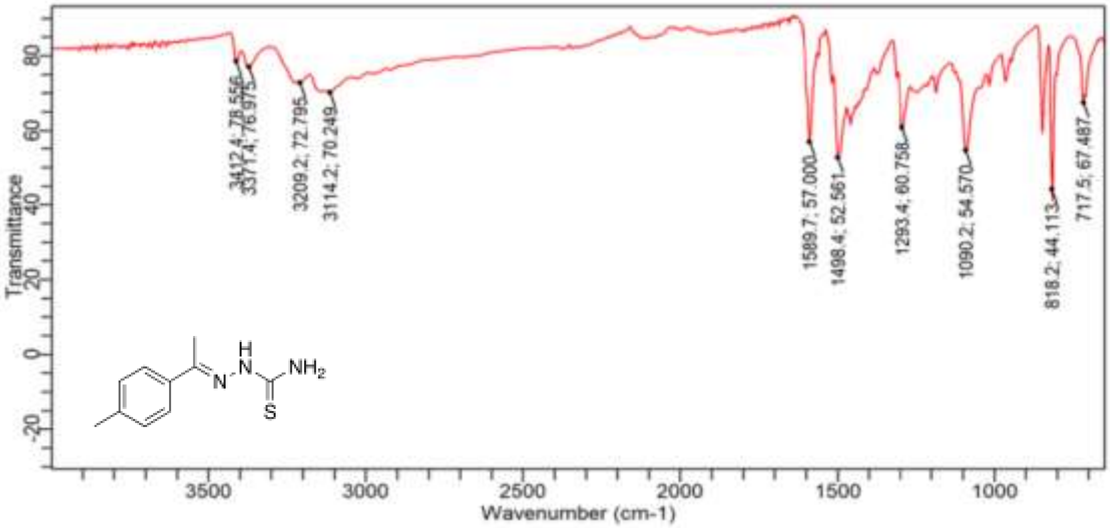

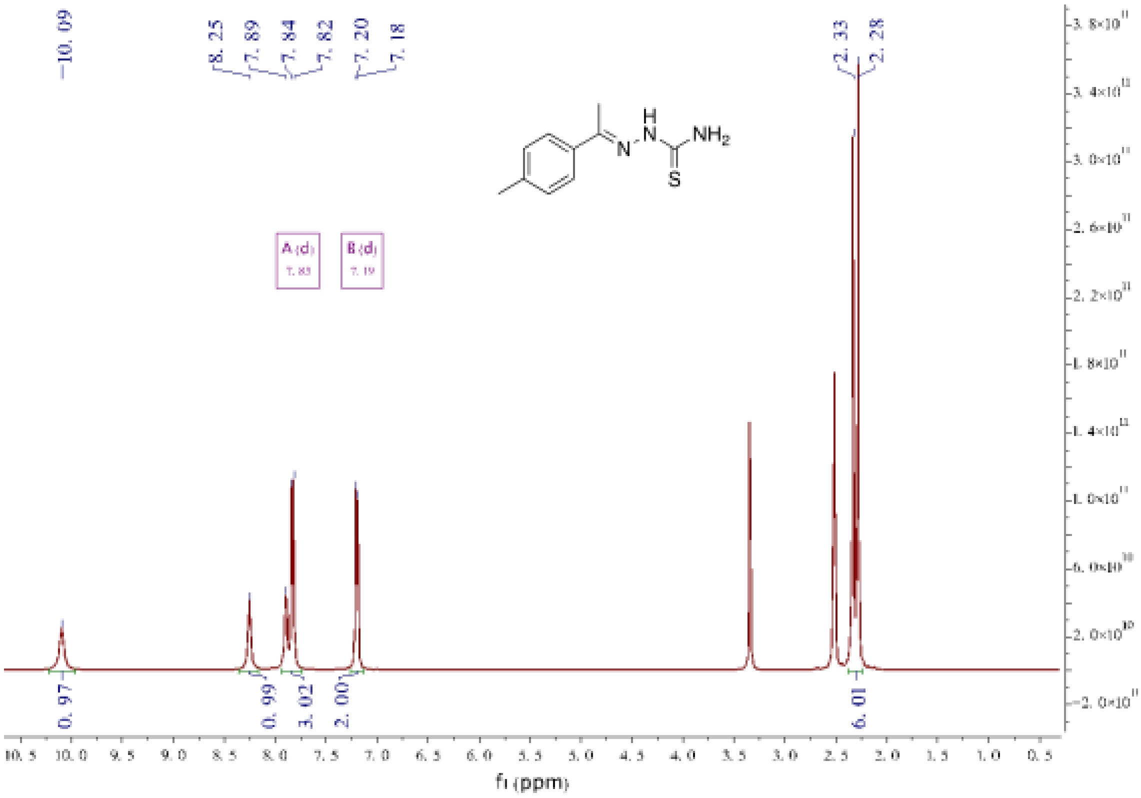

Figure S7. ^{1}H-NMR (up) and ^{13}C-NMR (down) of compound ***SO1***.

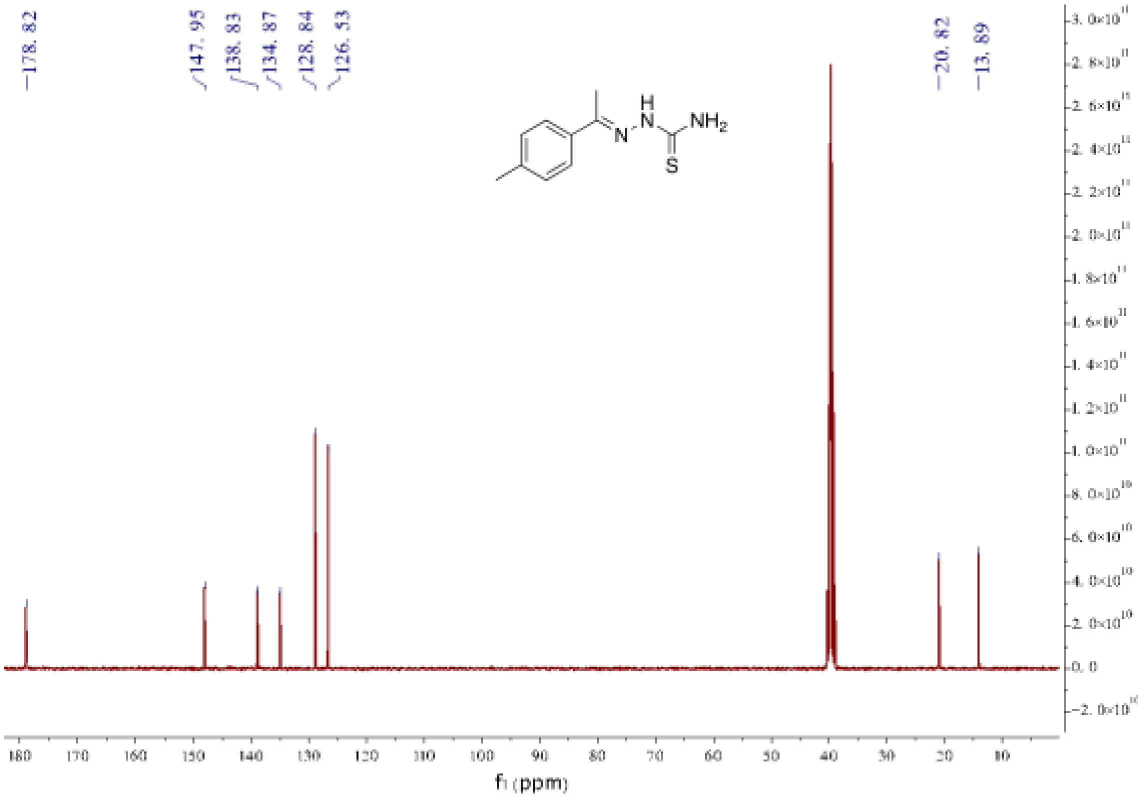

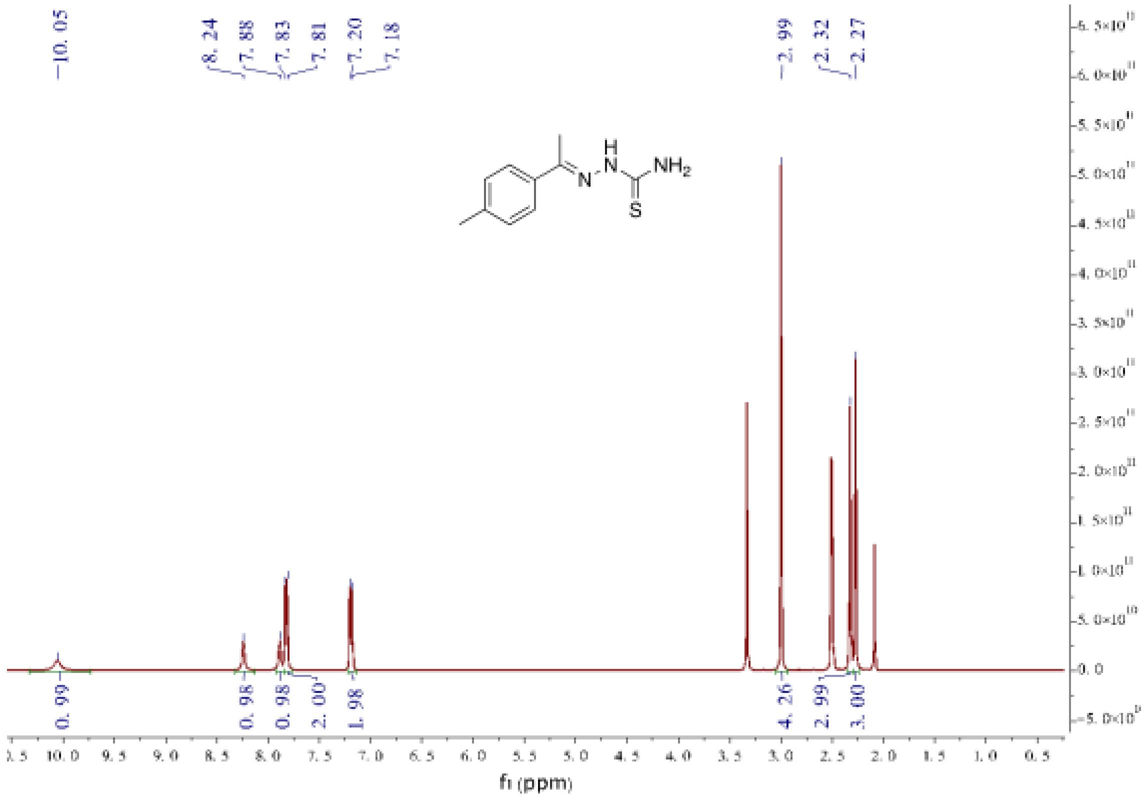

Figure S8. qNMR (up) of compound ***S*O1** and IR (down) of compound ***S*C1**.

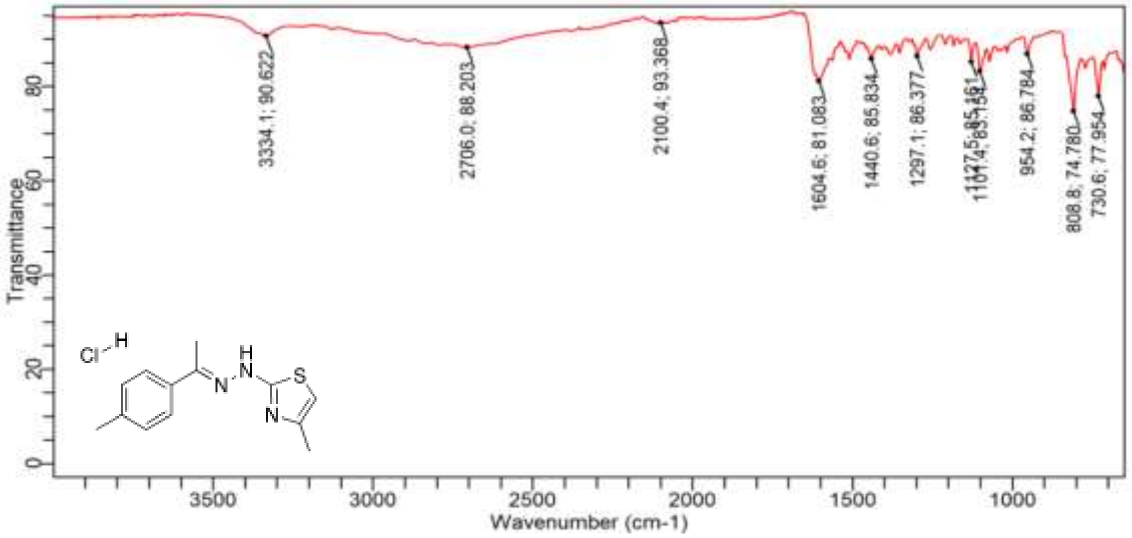

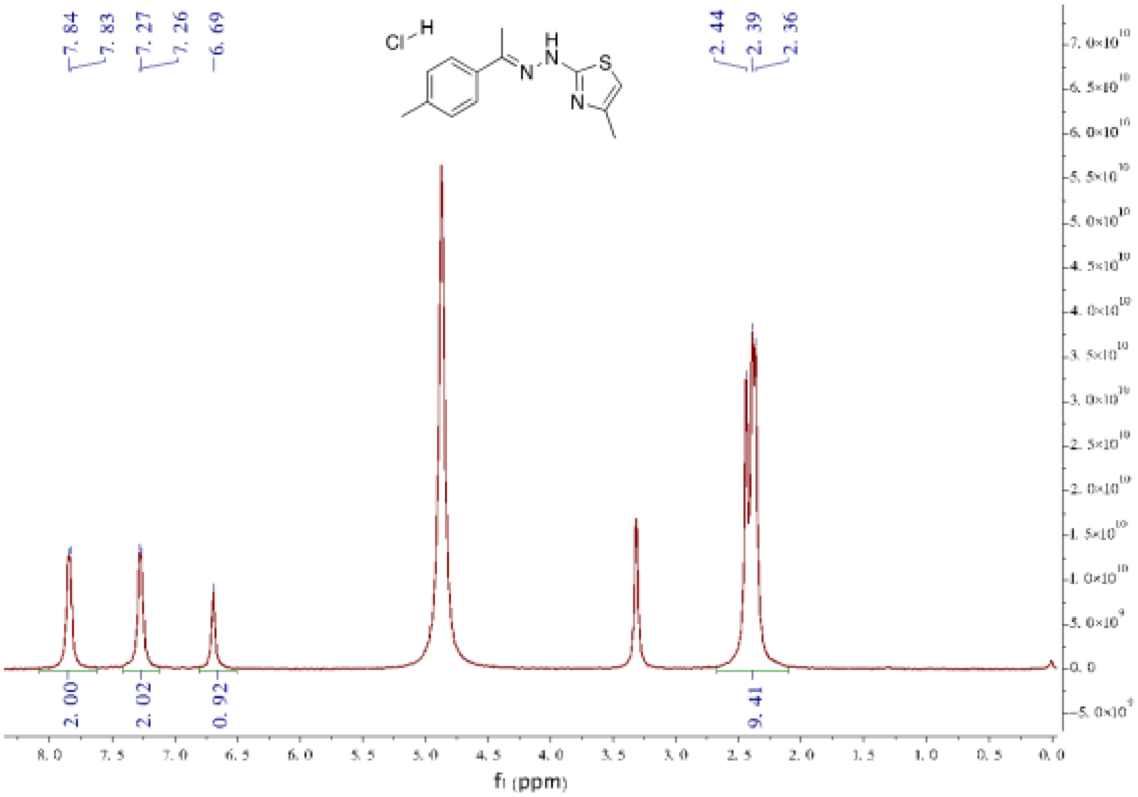

Figure S9. ^{1}H-NMR (up) and ^{13}C-NMR (down) of compound ***S*C1**.

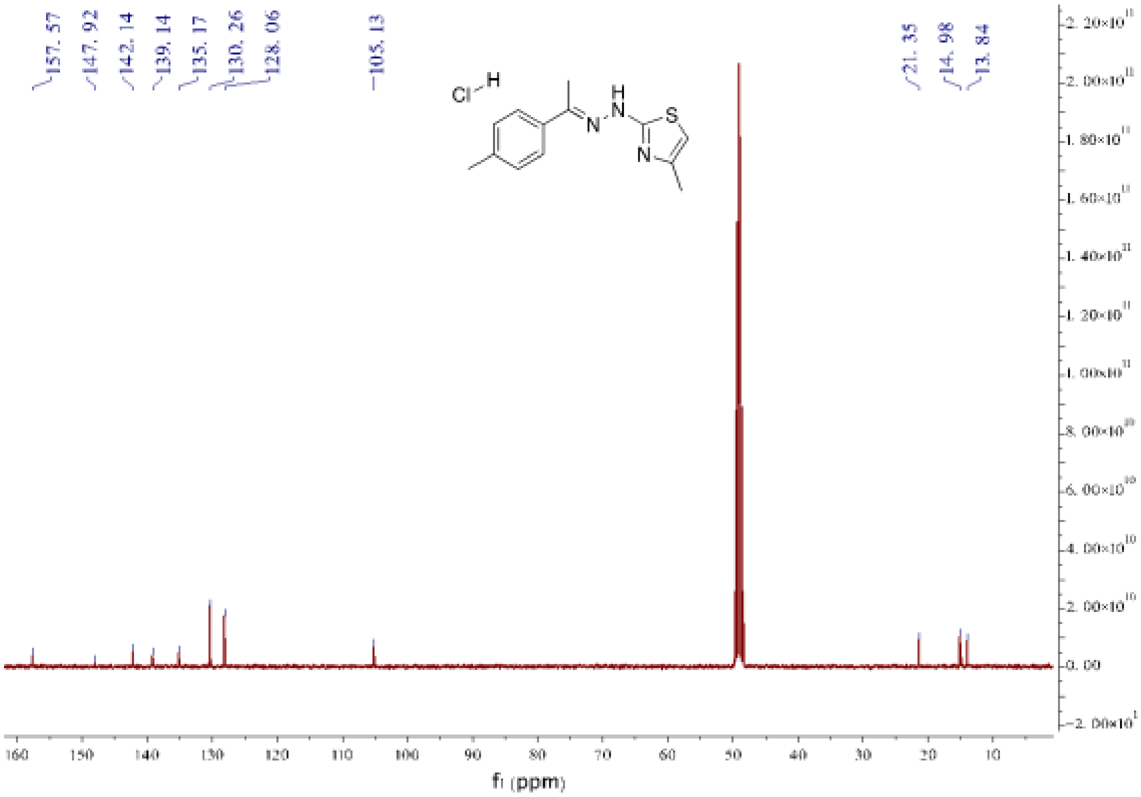

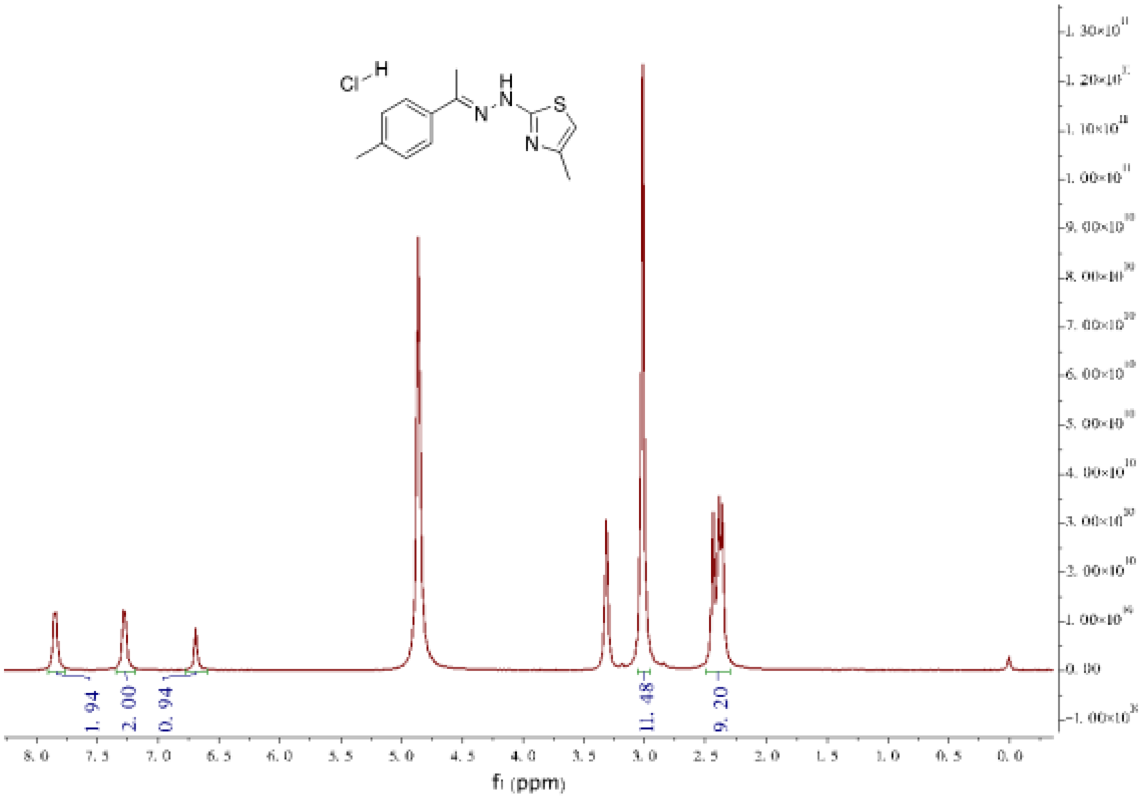

Figure S10. qNMR (up) of compound ***S*C1**and IR of compound ***Se*O2** (down).

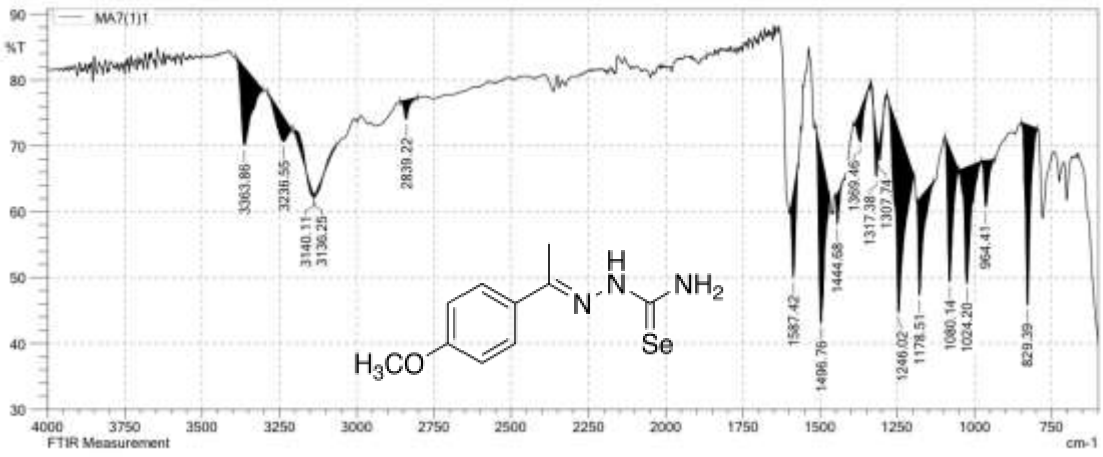

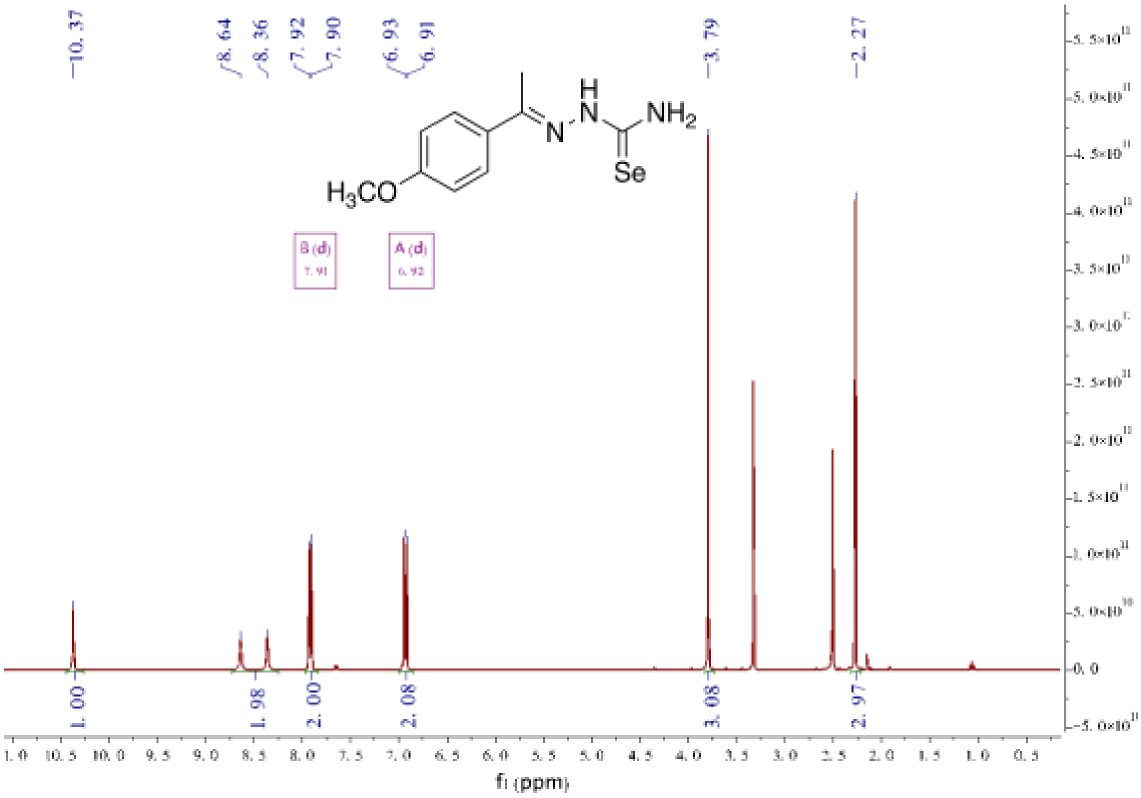

Figure S11. ^{1}H-NMR (up) and ^{13}C-NMR (down) of compound ***Se*O2**.

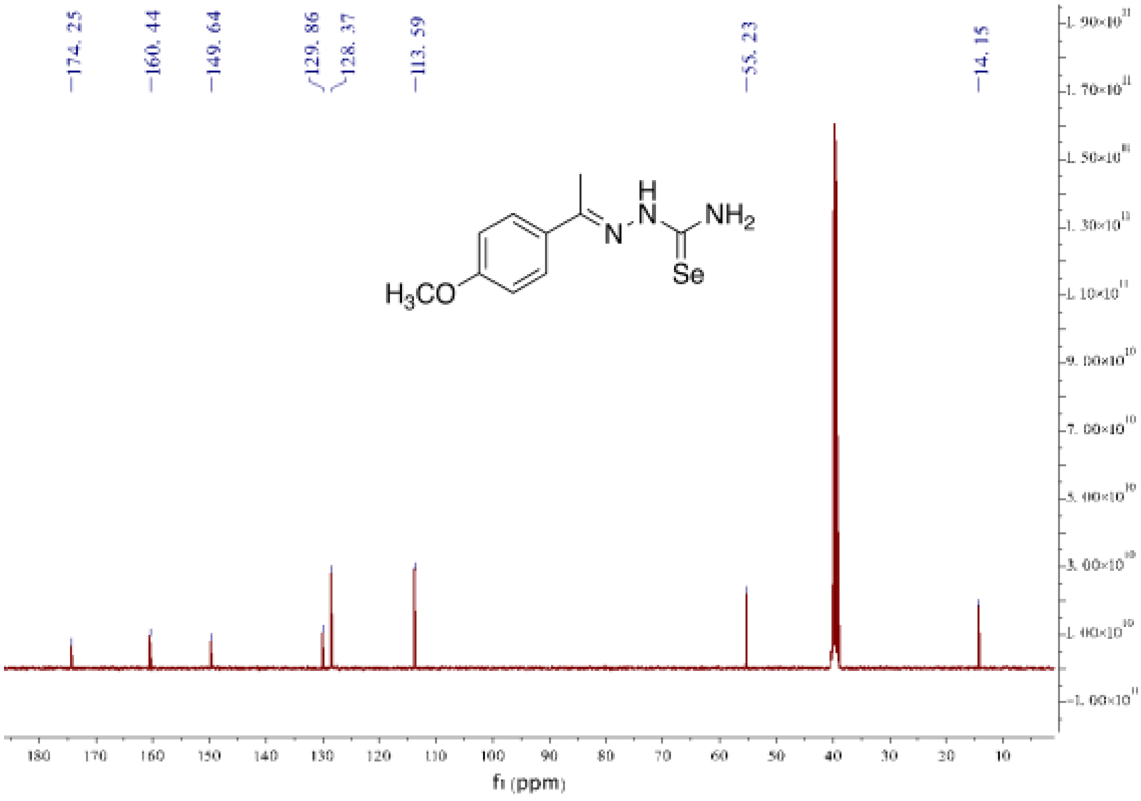

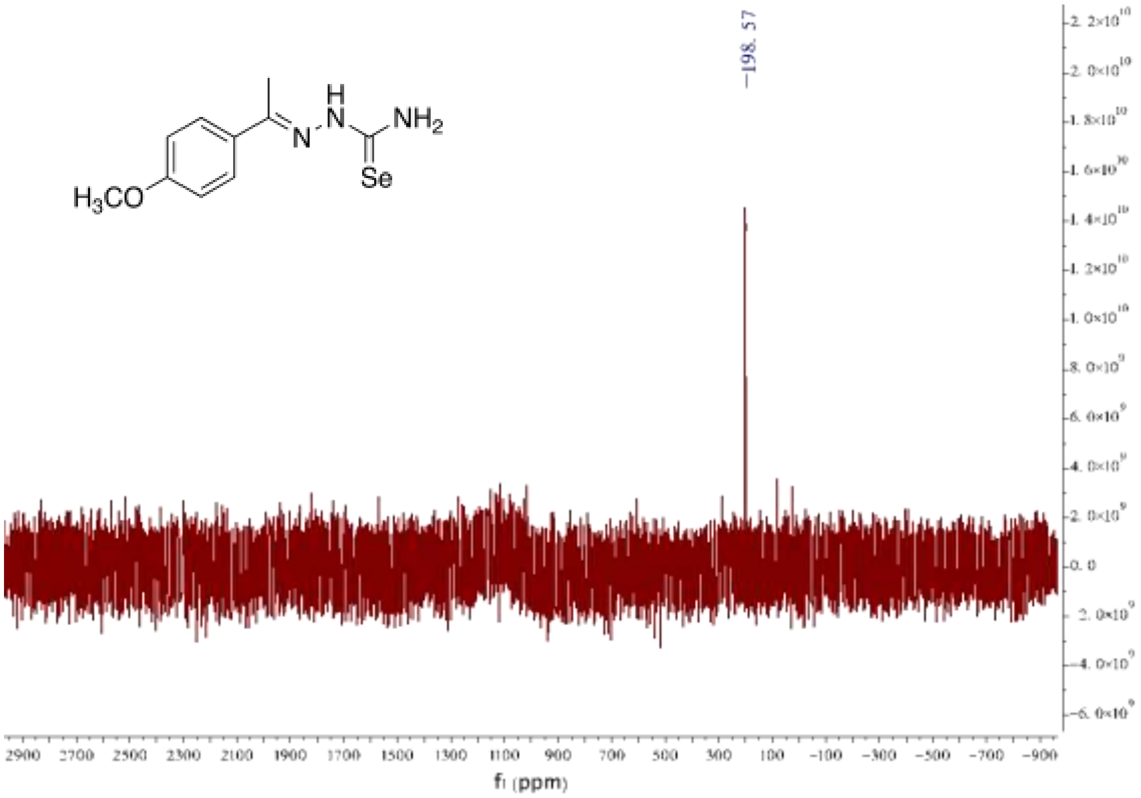

Figure S12. ^{77}Se-NMR (up) and qNMR (down) of compound ***Se*O2**.

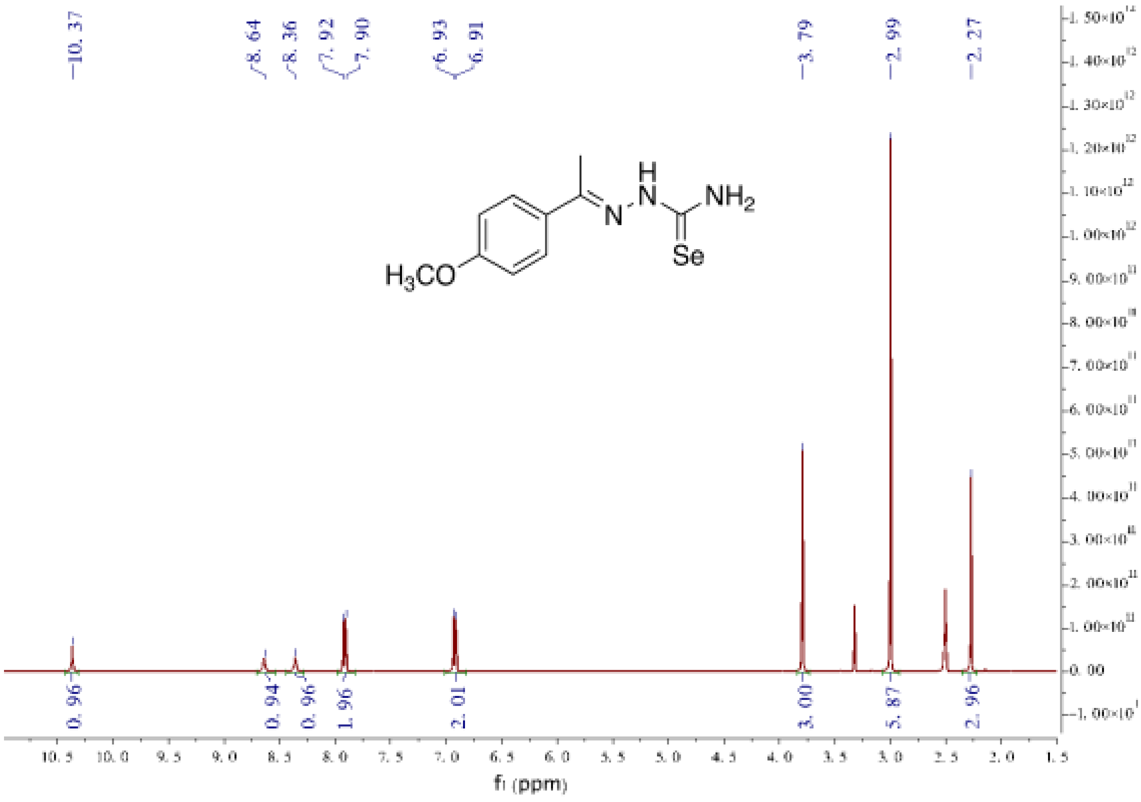

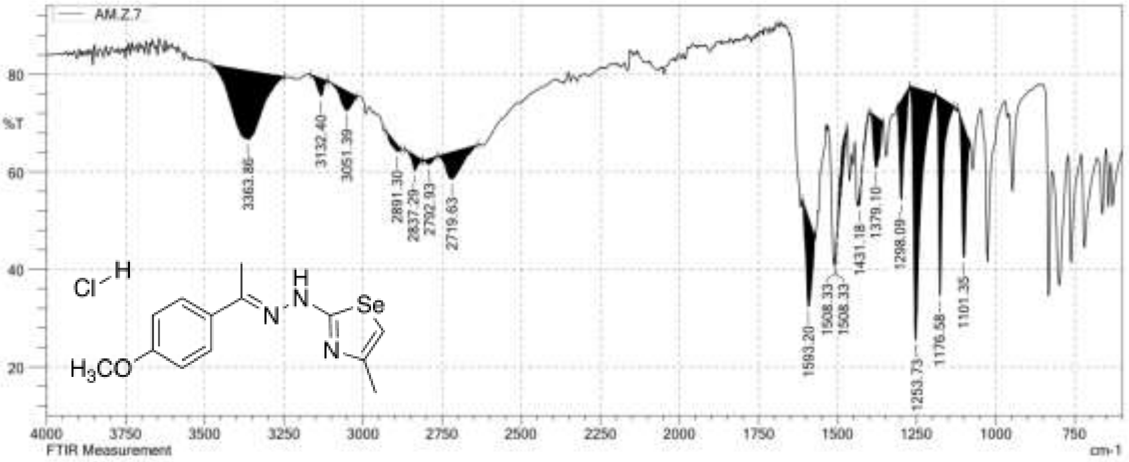

Figure S13. IR (up) and ^{1}H-NMR (down) of compound ***Se*C2**.

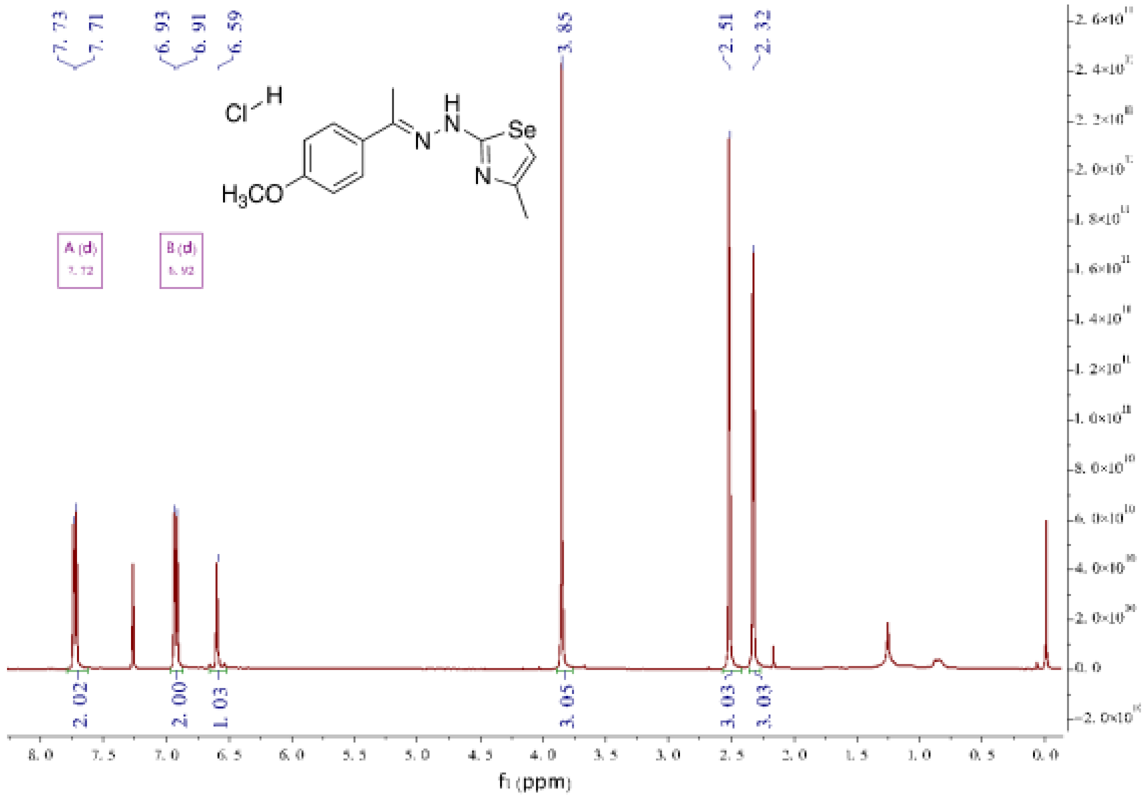

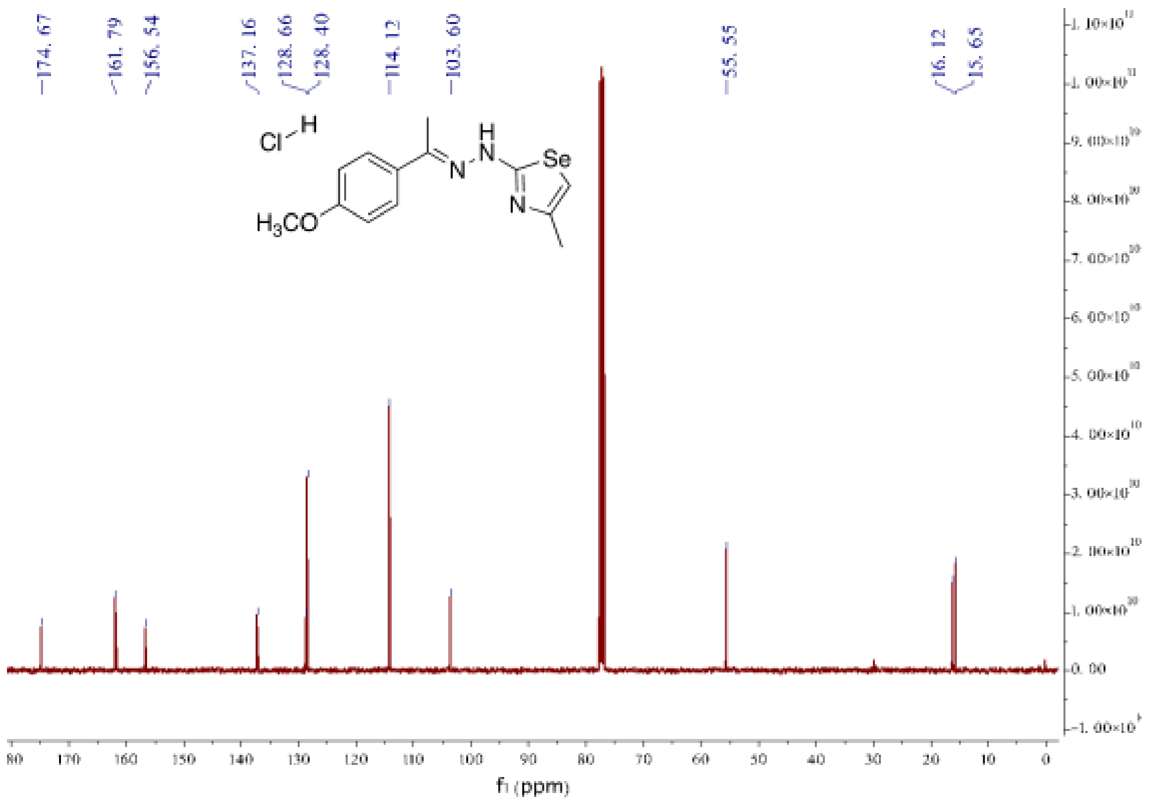

Figure S14. ^{13}C-NMR (up) and qNMR (down) of compound ***Se*C2**.

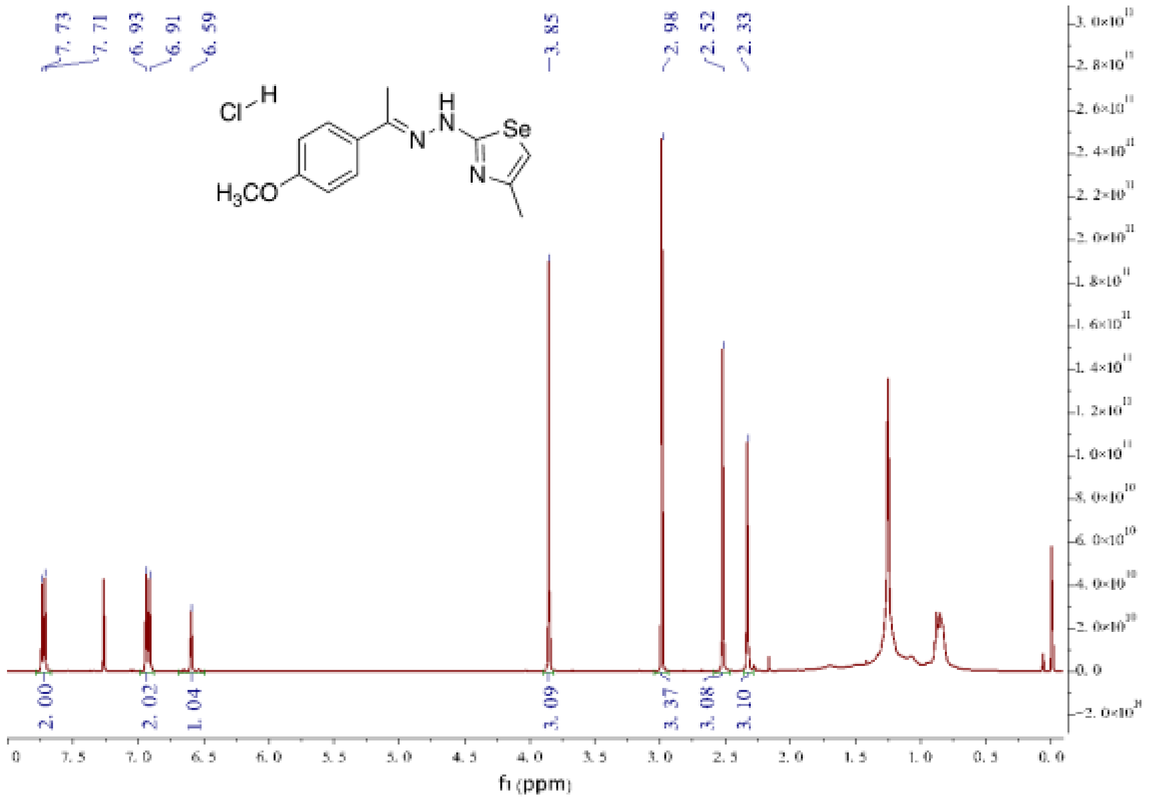

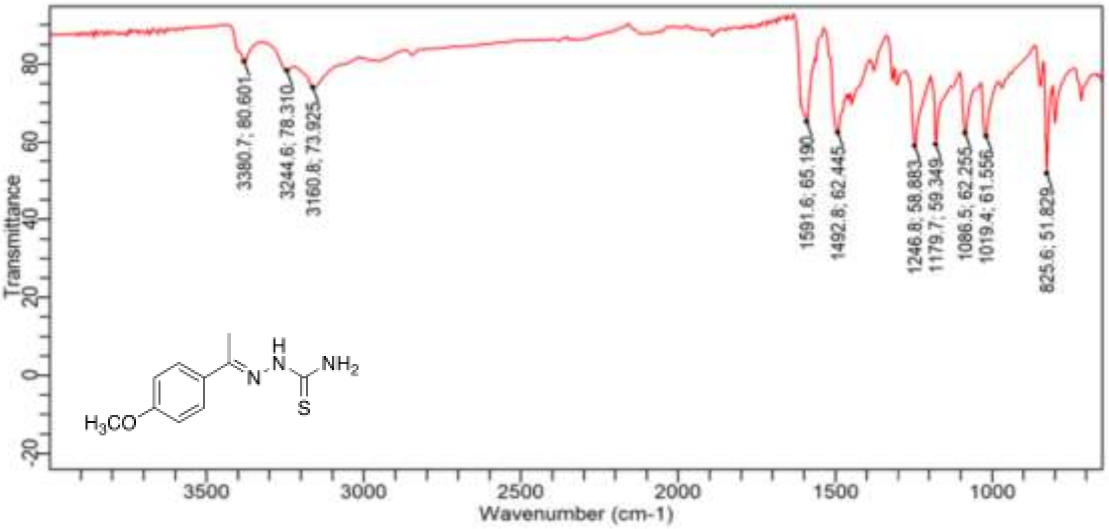

Figure S15. IR (up) and ^{1}H-NMR (down) of compound ***SO*2**.

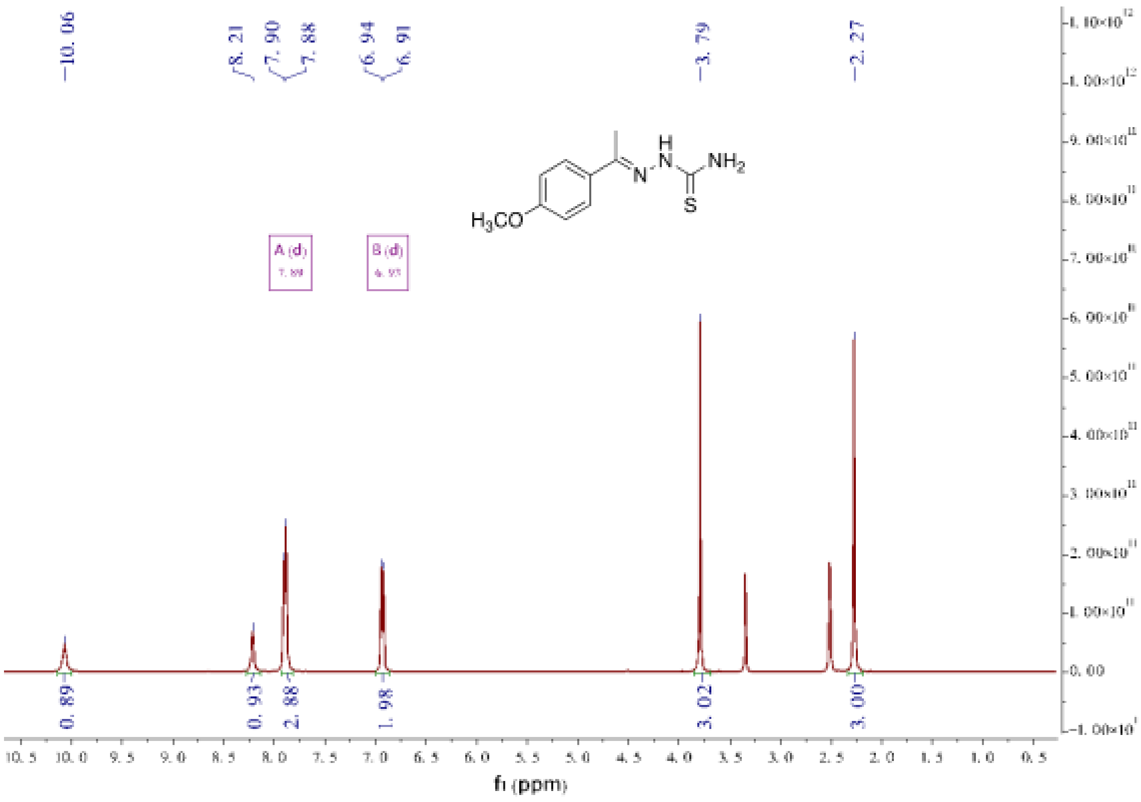

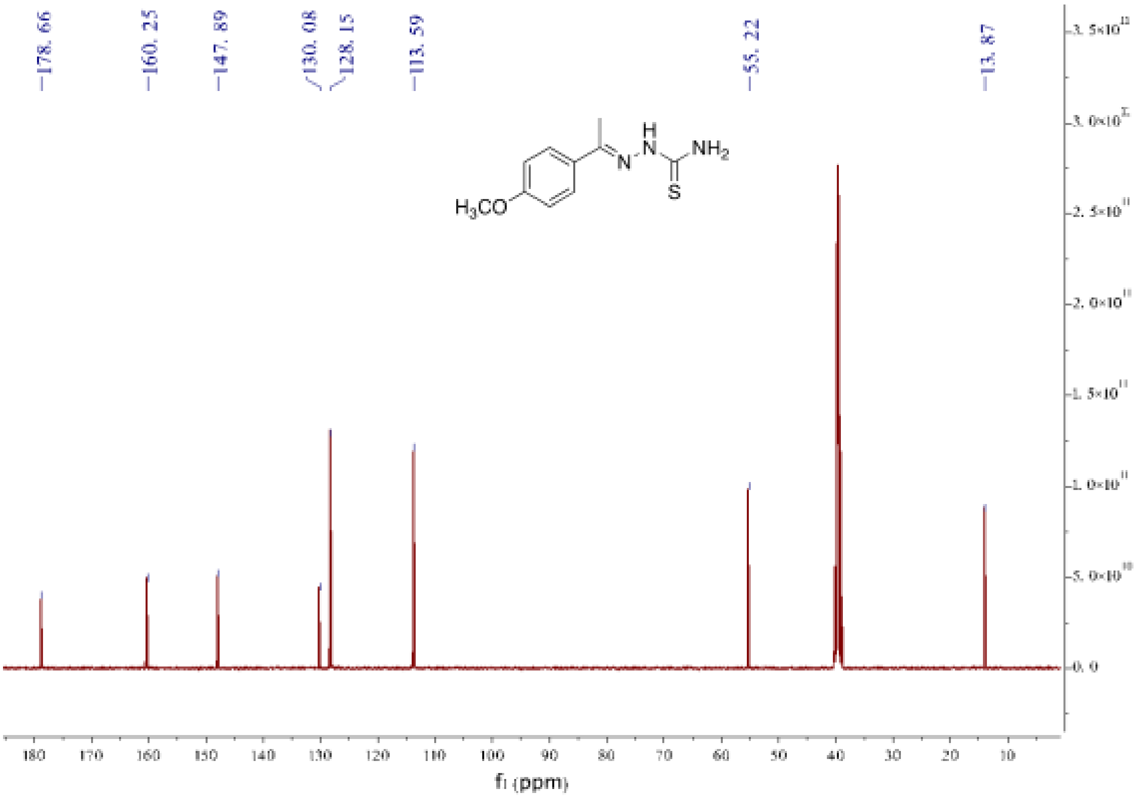

Figure S16. ^{13}C-NMR (up) and qNMR (down) of compound **SO2**.

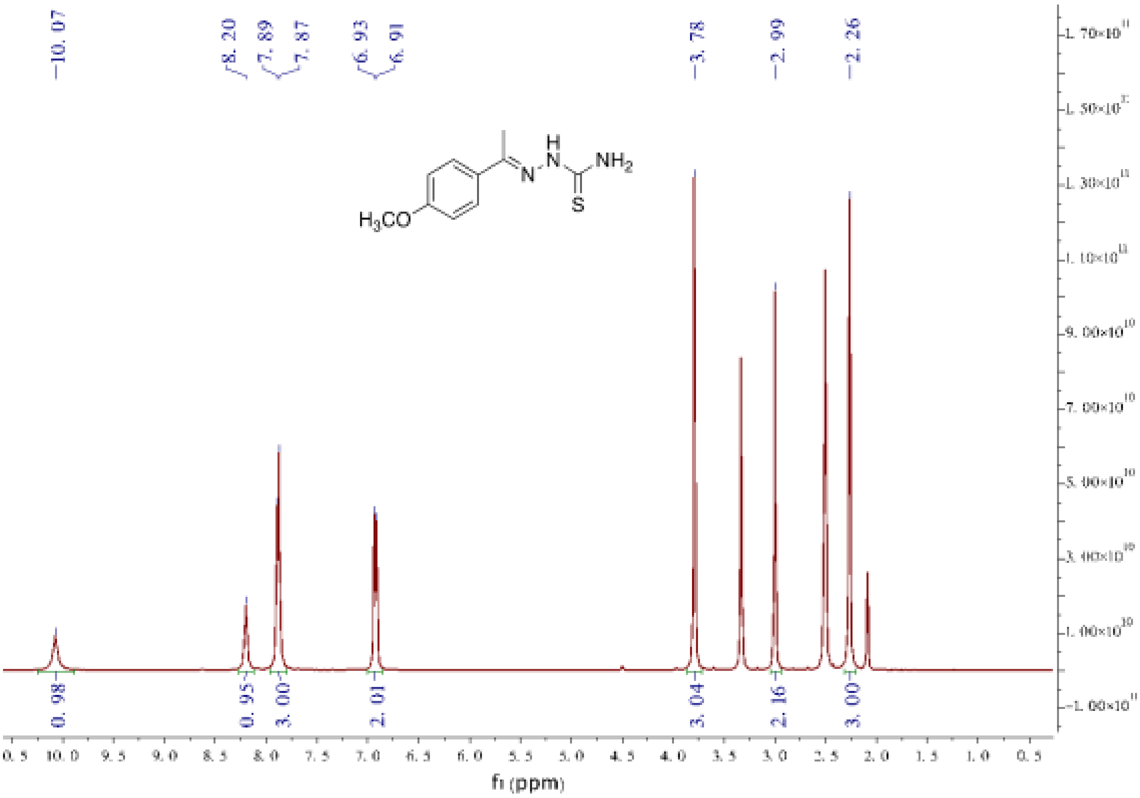

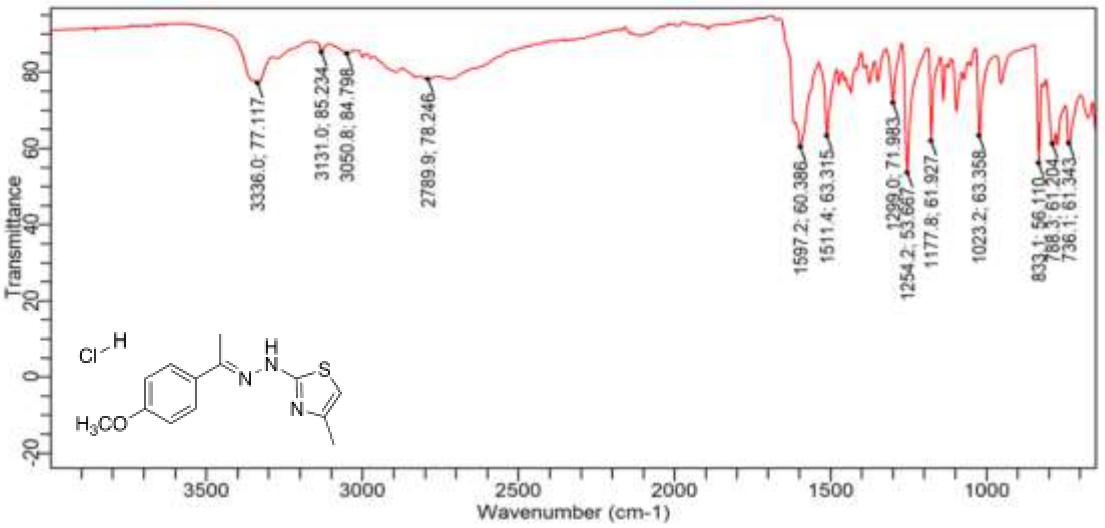

Figure S17. IR (up) and ^{1}H-NMR (down) of compound ***S*C2**.

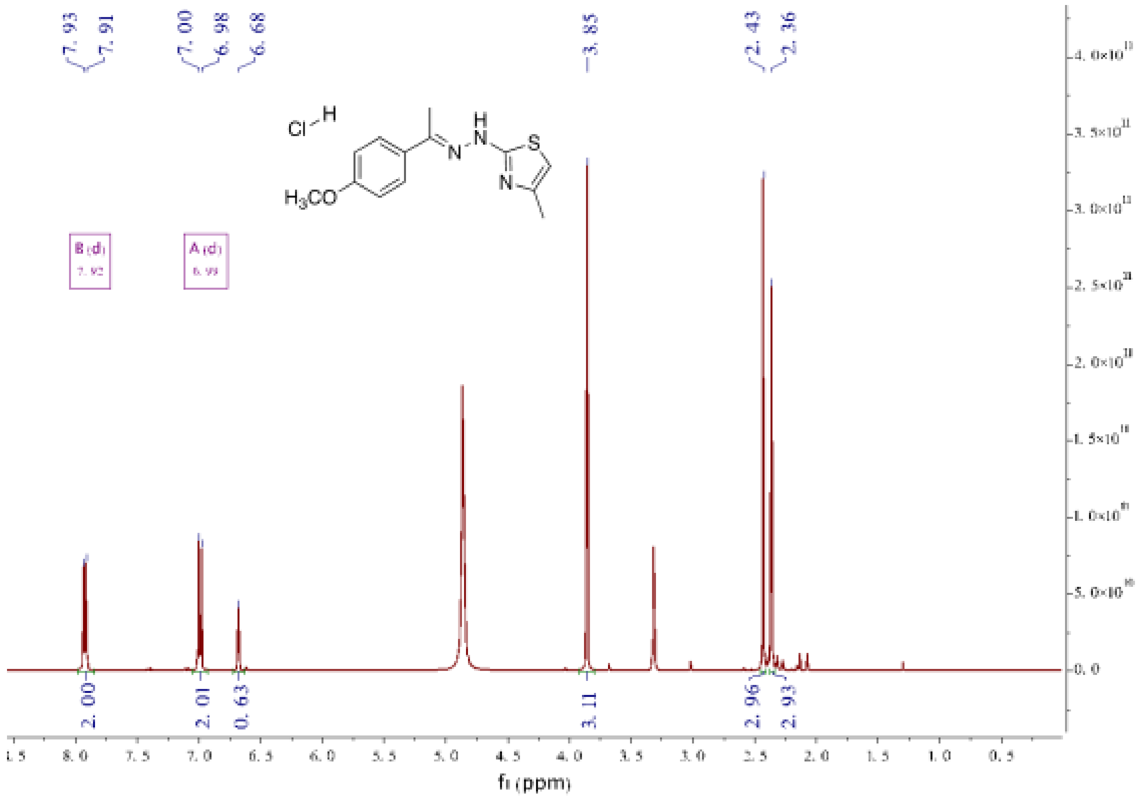

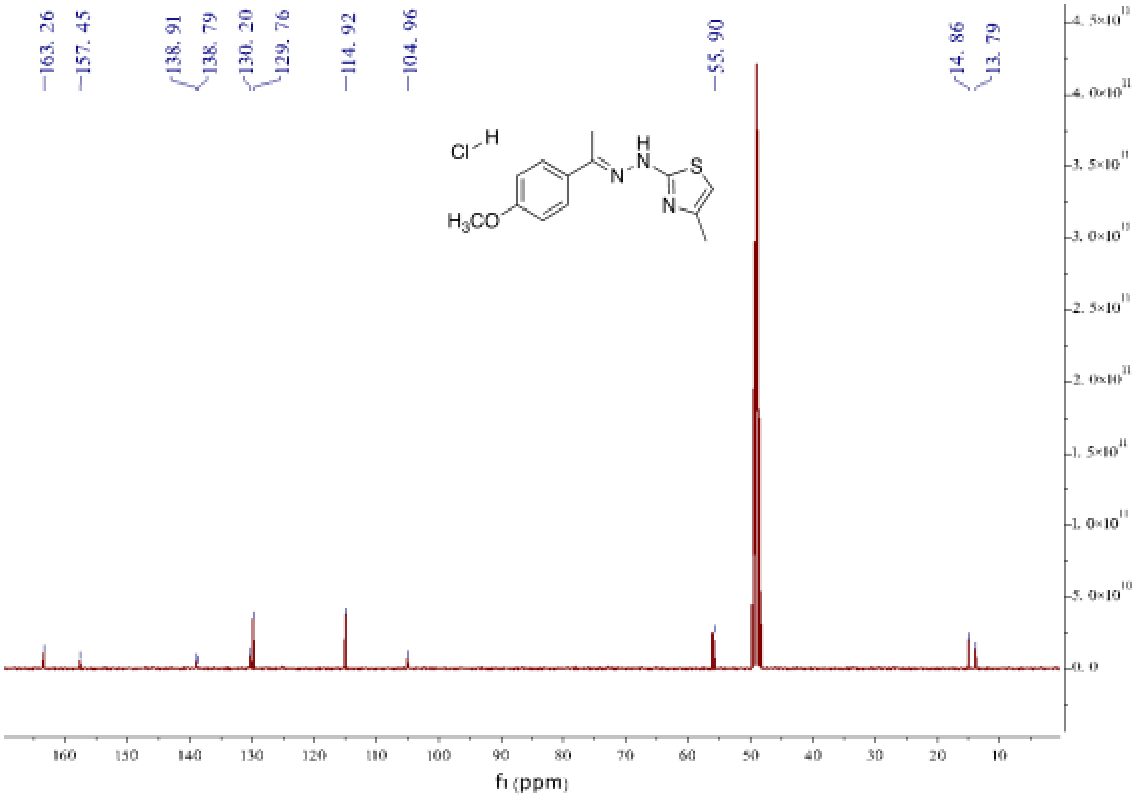

Figure S18. ^{13}C-NMR (up) and qNMR (down) of compound ***S*****C2**.

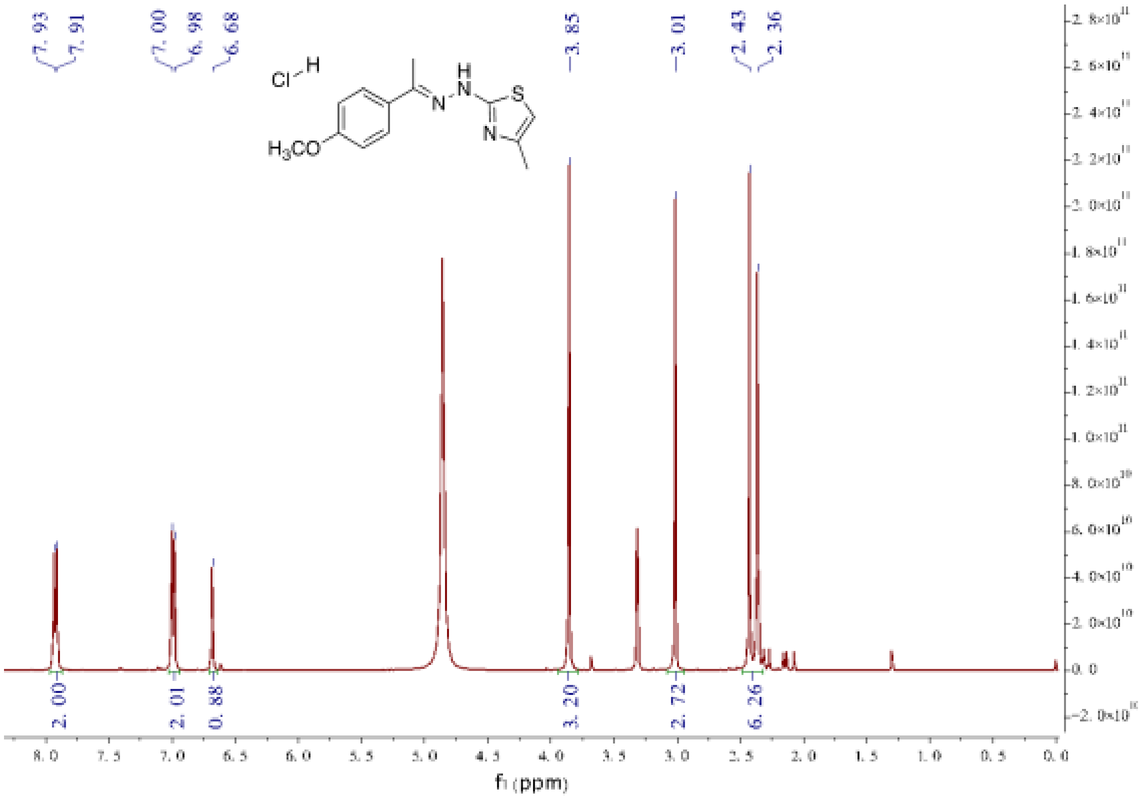

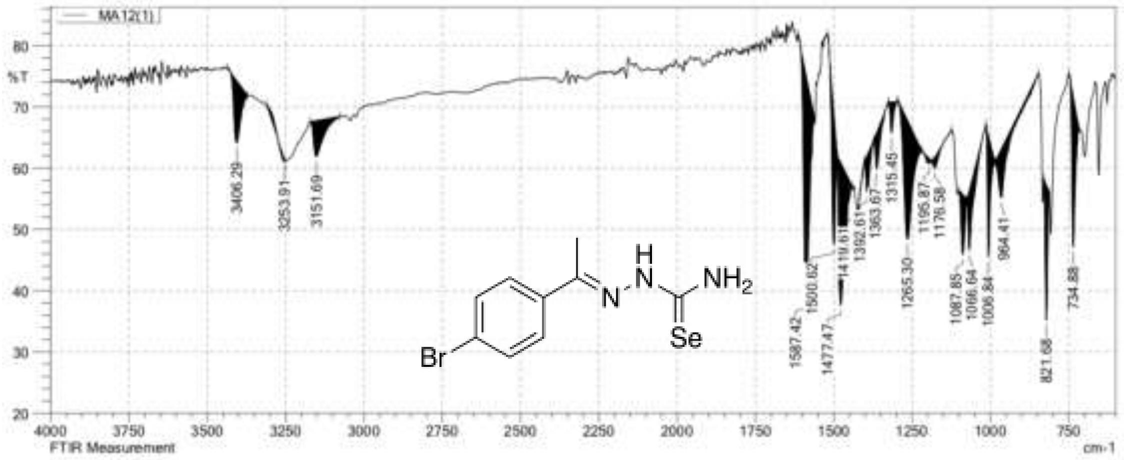

Figure S19. IR (up) and ^{1}H-NMR (down) of compound ***Se*O3**

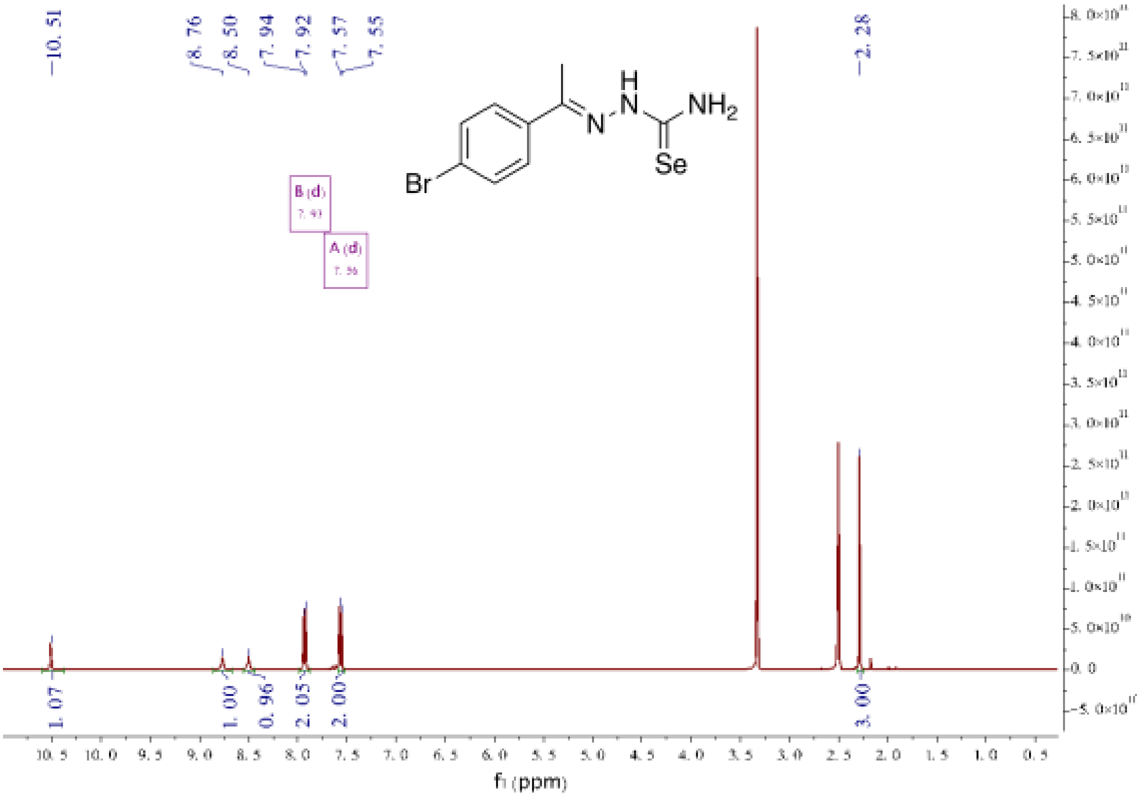

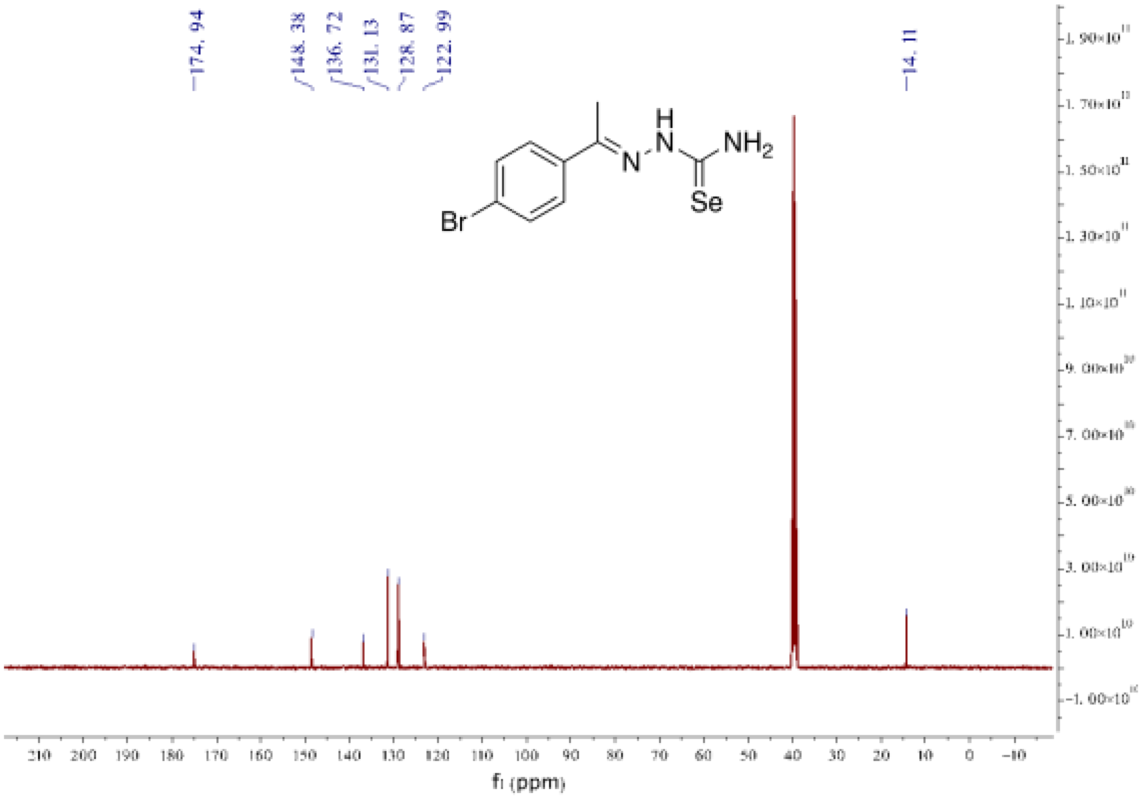

Figure S20. ^{13}C-NMR (up) and ^{77}Se-NMR (down) of compound ***Se*O3**.

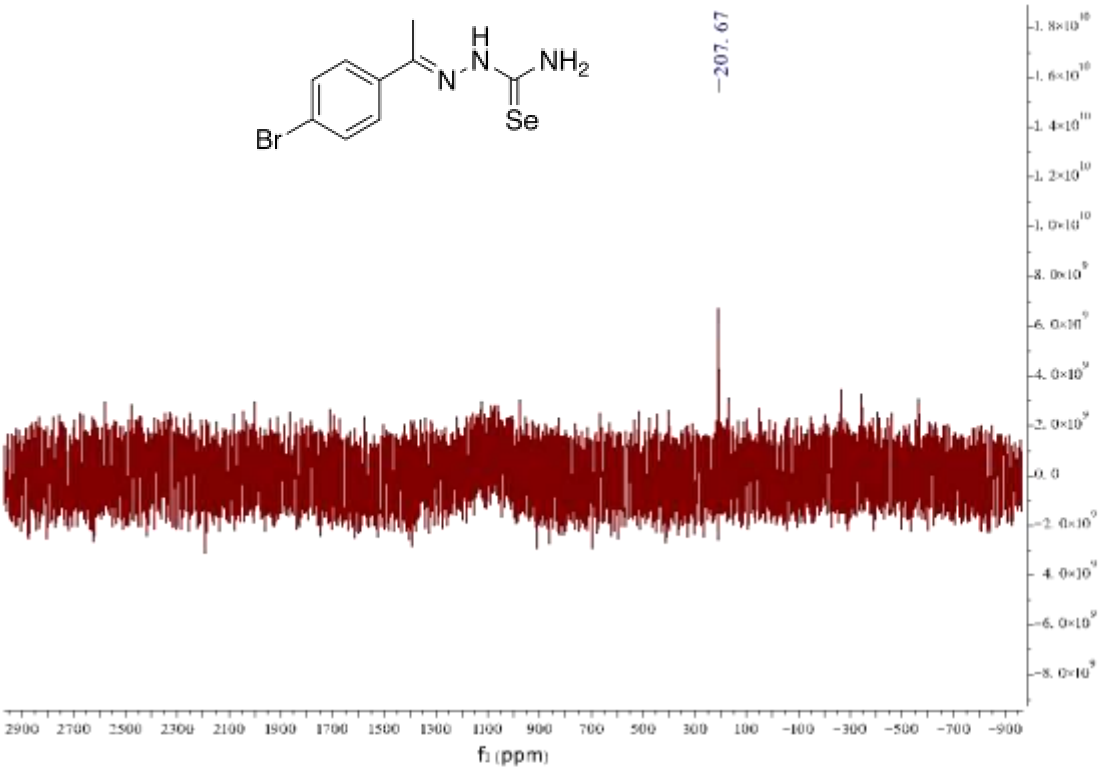

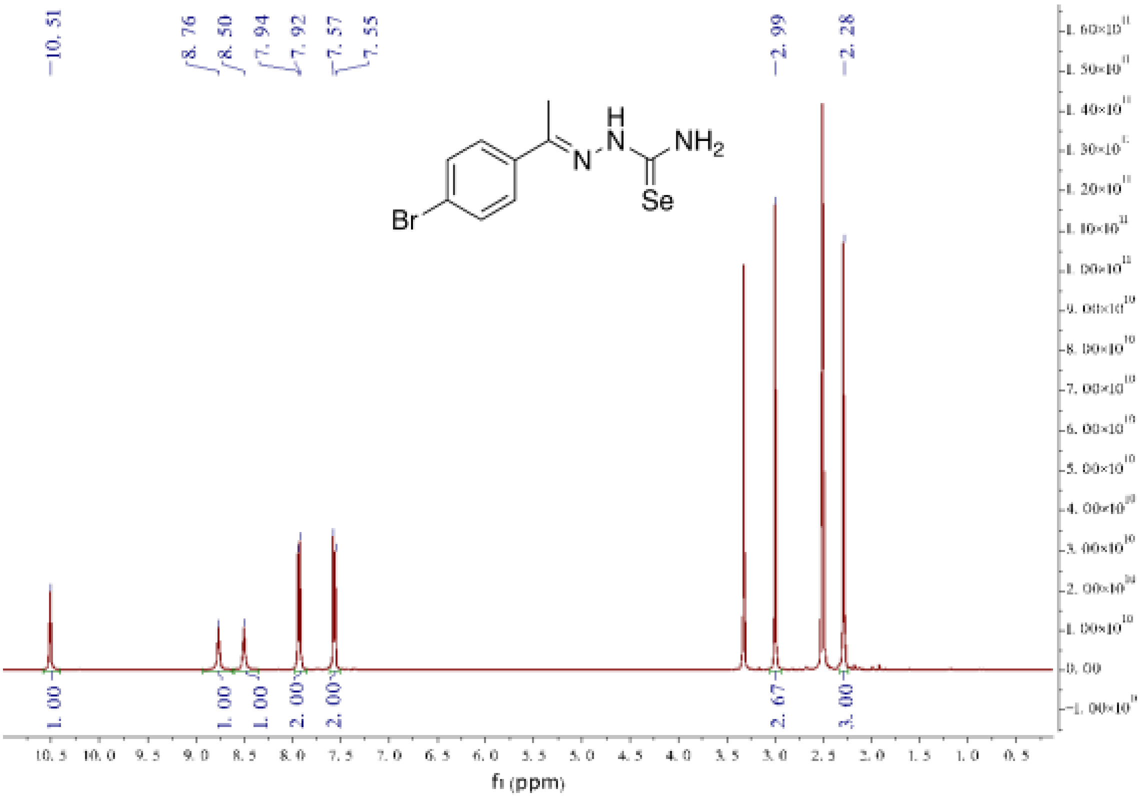

Figure S21. qNMR (up) of compound ***Se*O3** and IR (down) of compound ***Se*C3.**

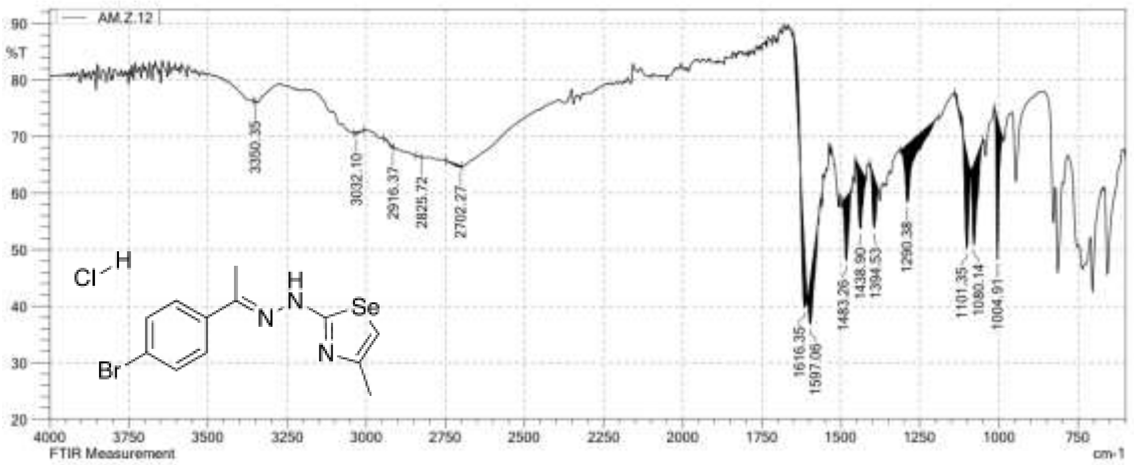

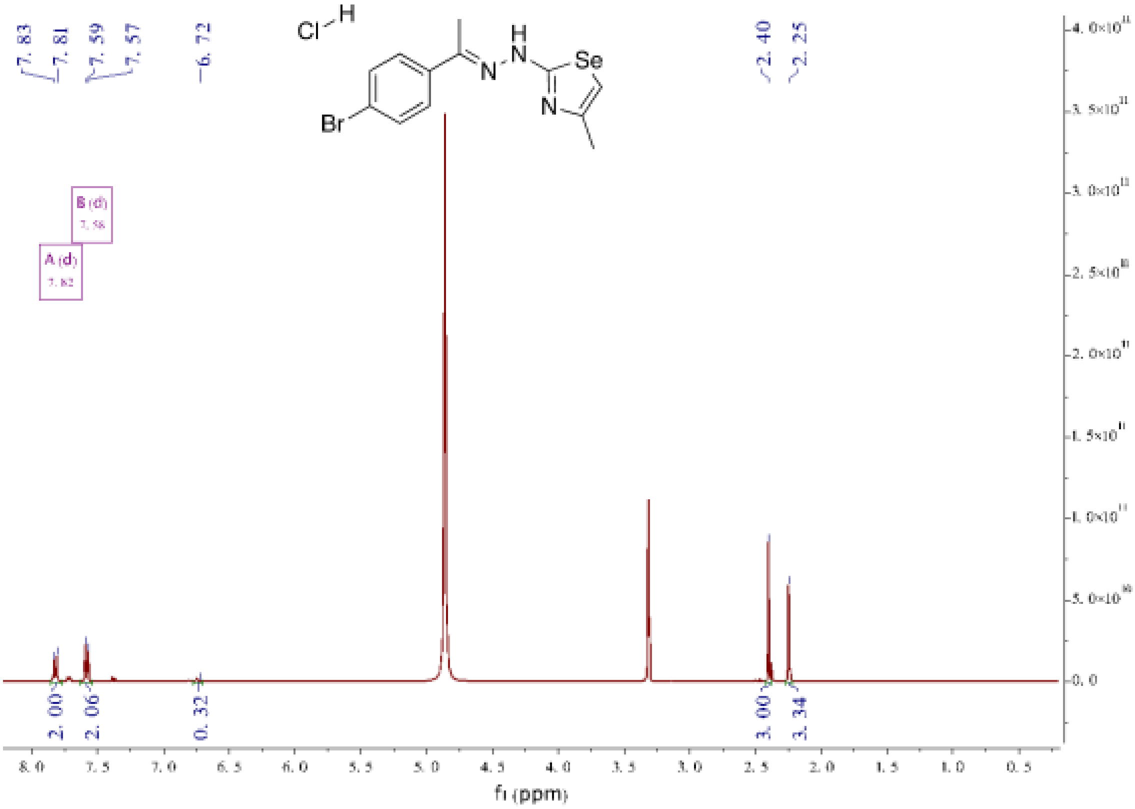

Figure S22. ^{1}H-NMR (up) and ^{13}C-NMR (down) of compound ***Se*C3**.

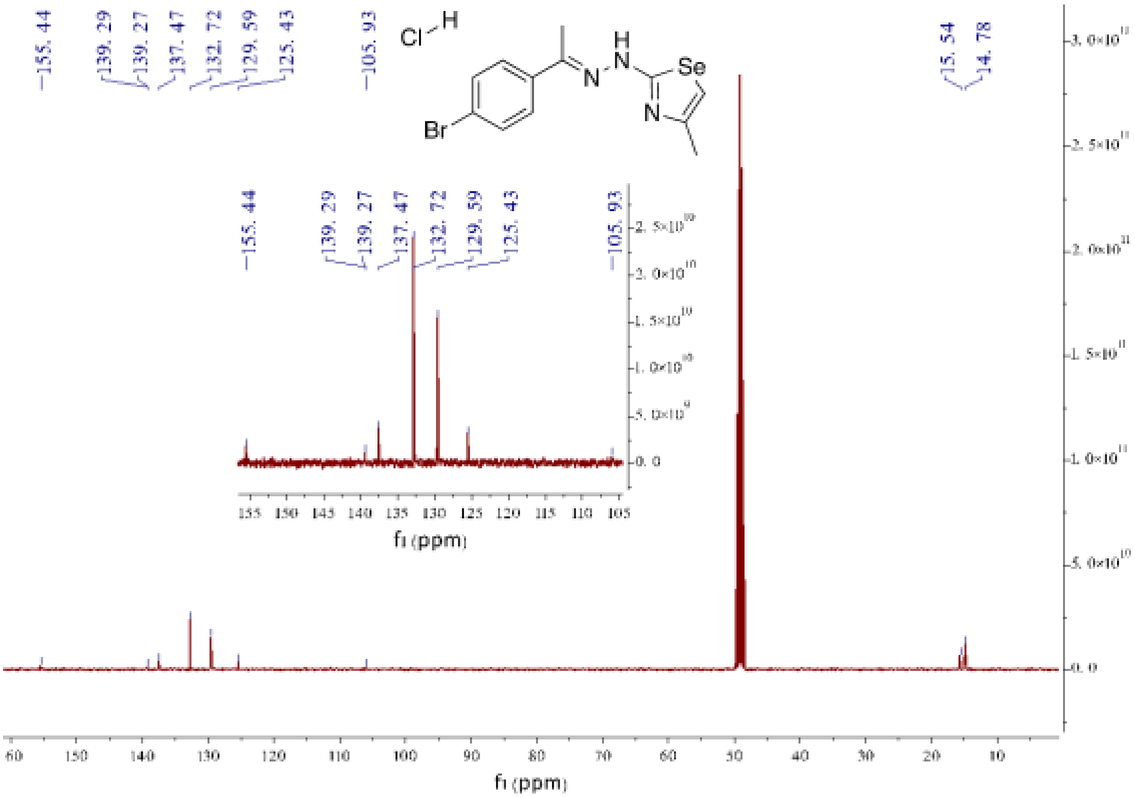

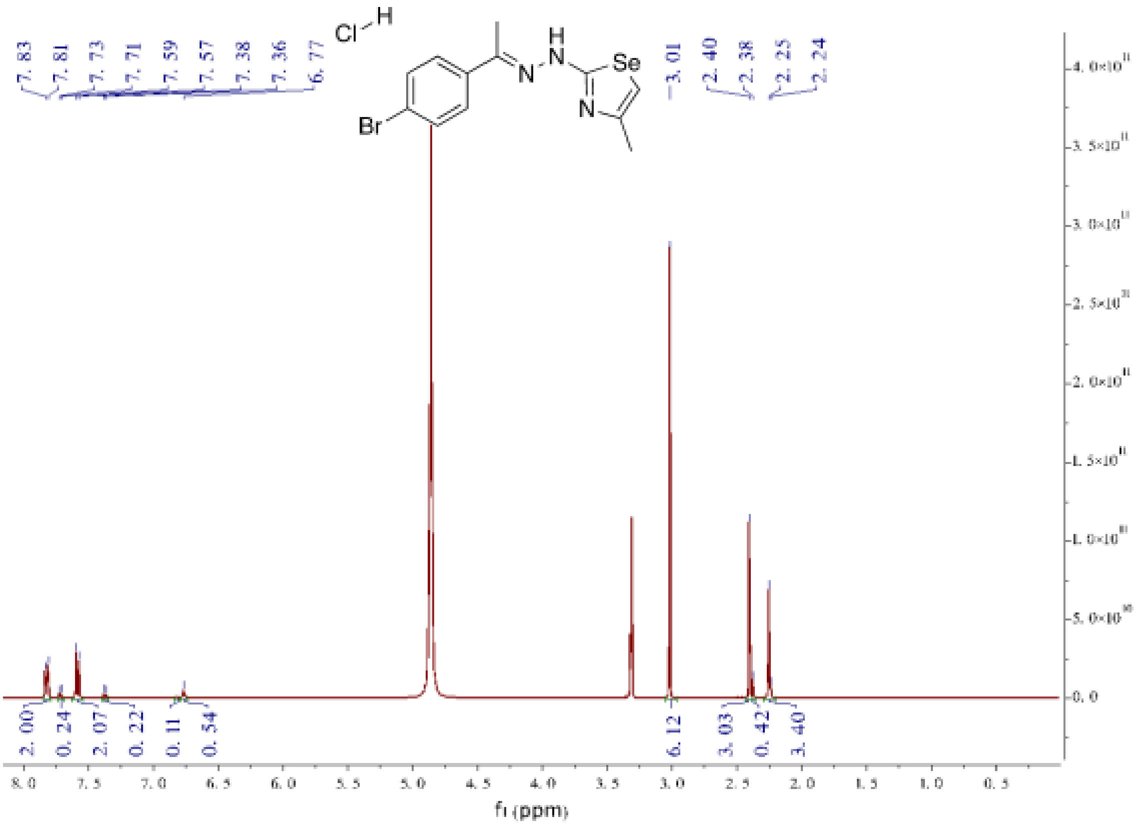

Figure S23. qNMR (up) of compound ***Se*C3** and IR (down) of compound **SO3**.

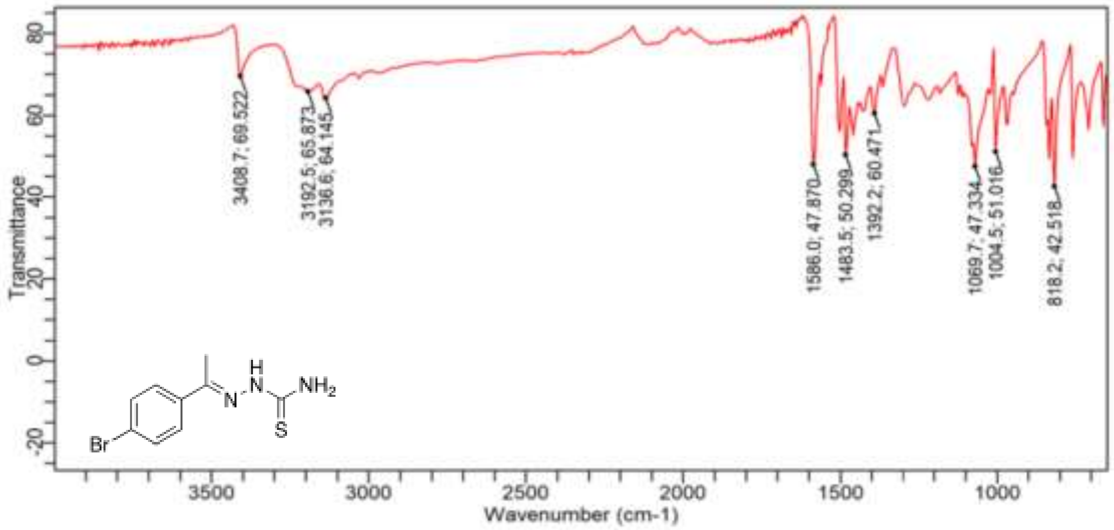

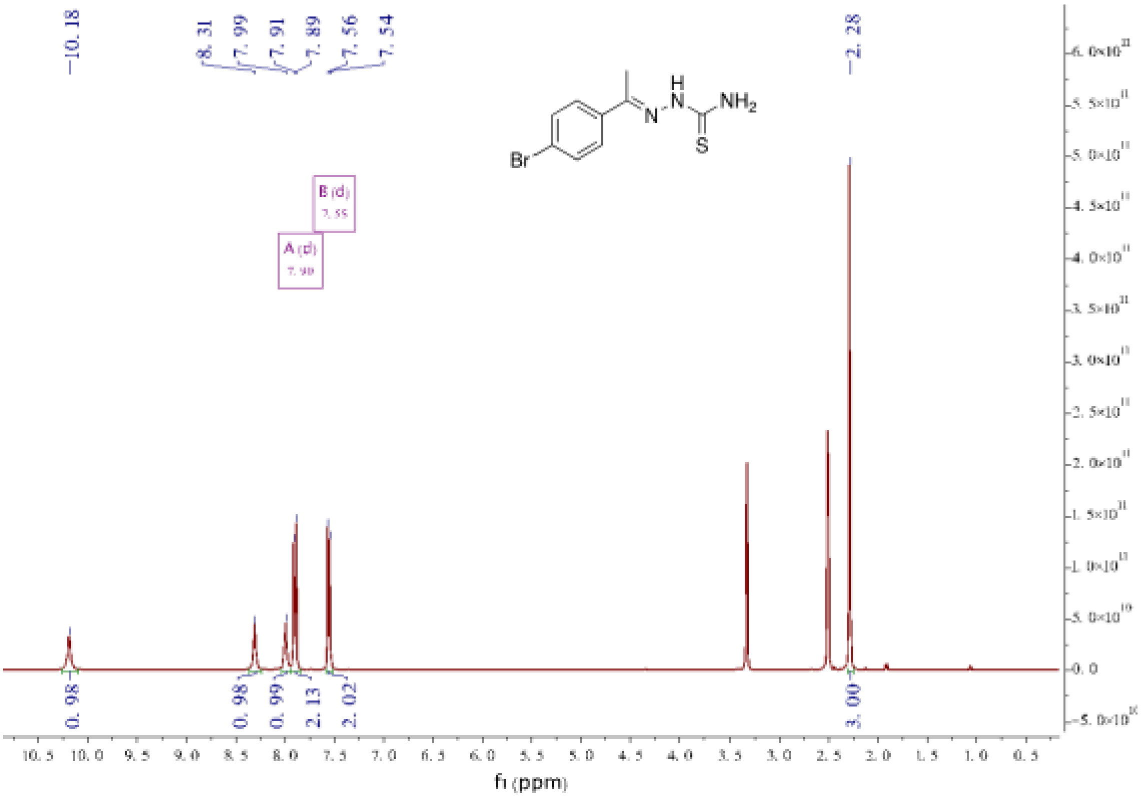

Figure S24. ^{1}H-NMR (up) and ^{13}C-NMR (down) of compound **SO3**.

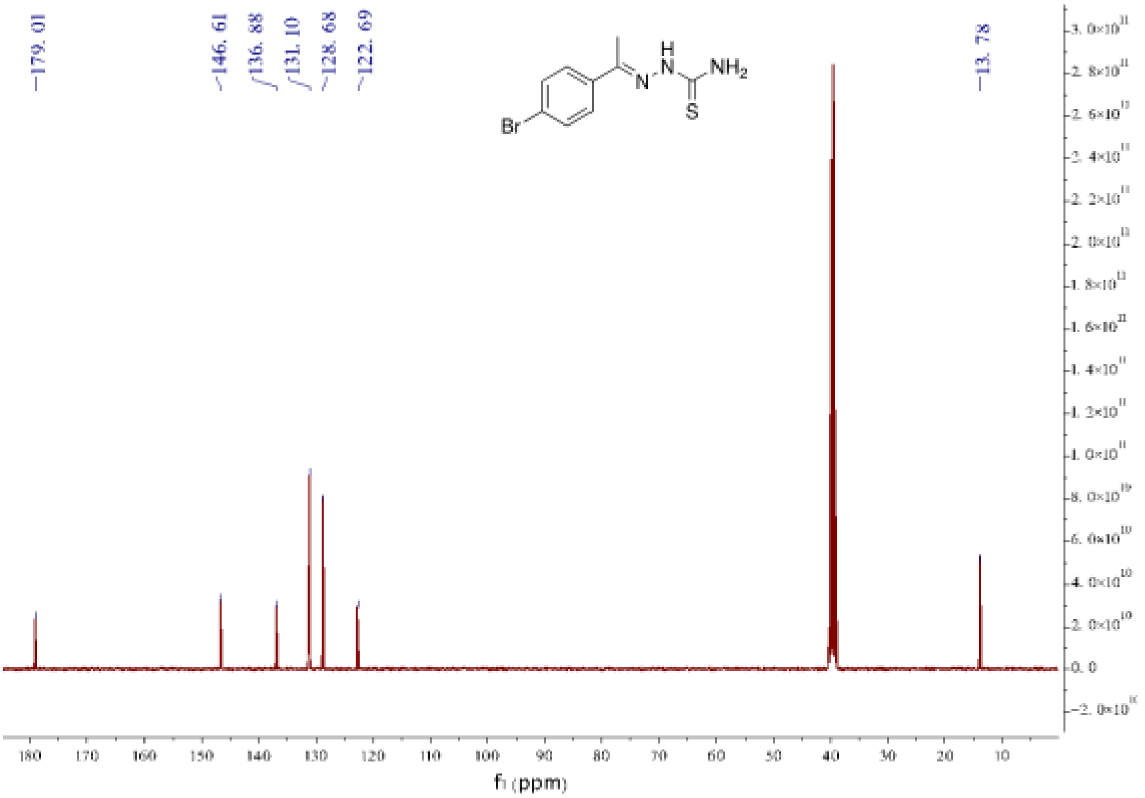

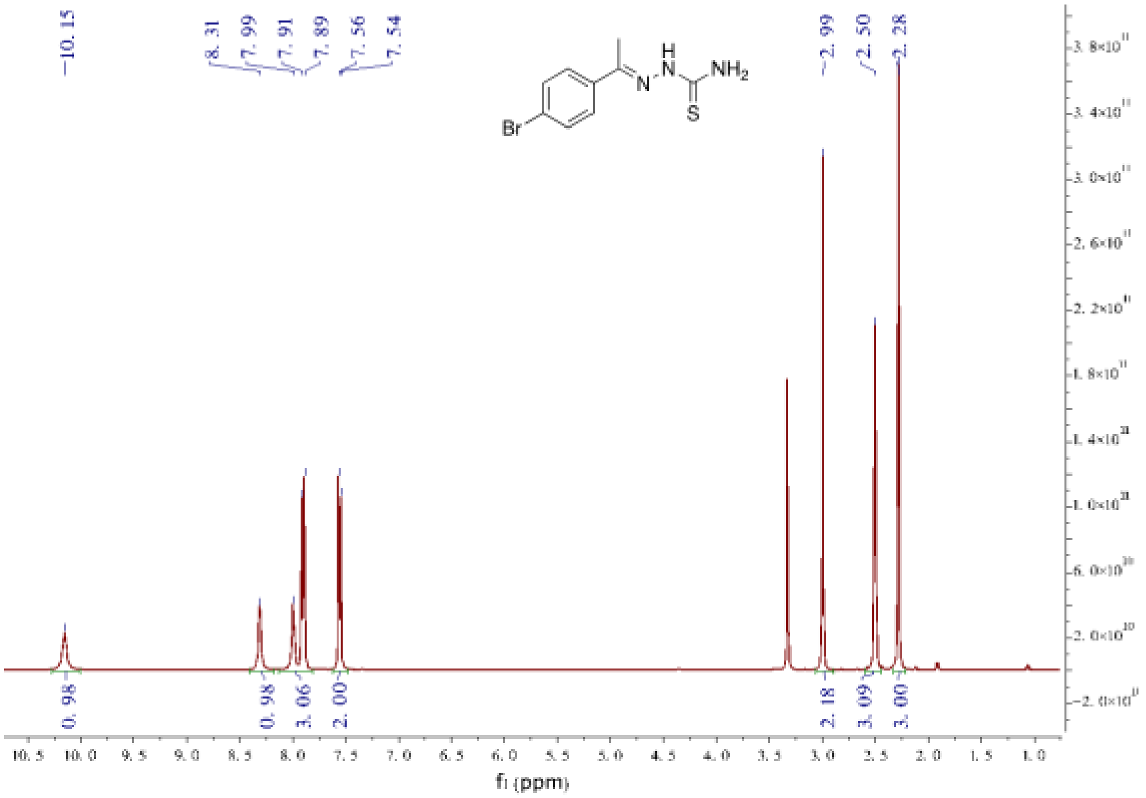

Figure S25. qNMR (up) of compound **SO3** and IR (down) of compound ***S*C3**.

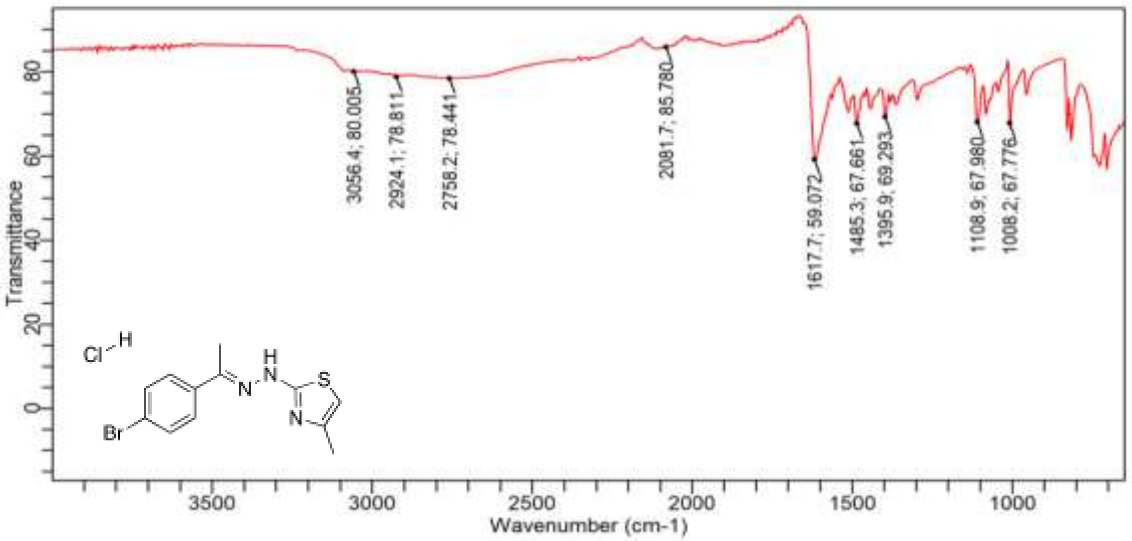

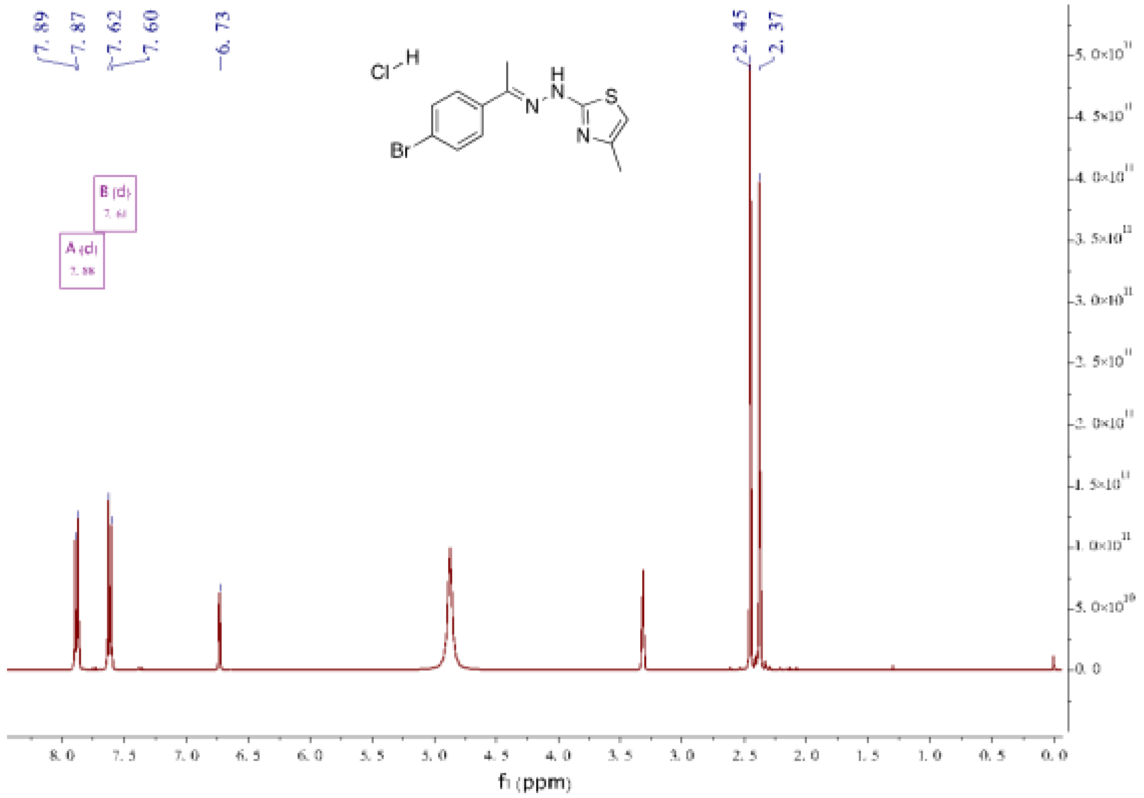

Figure S26. ^{1}H-NMR (up) and ^{13}C-NMR (down) of compound ***S*C3**.

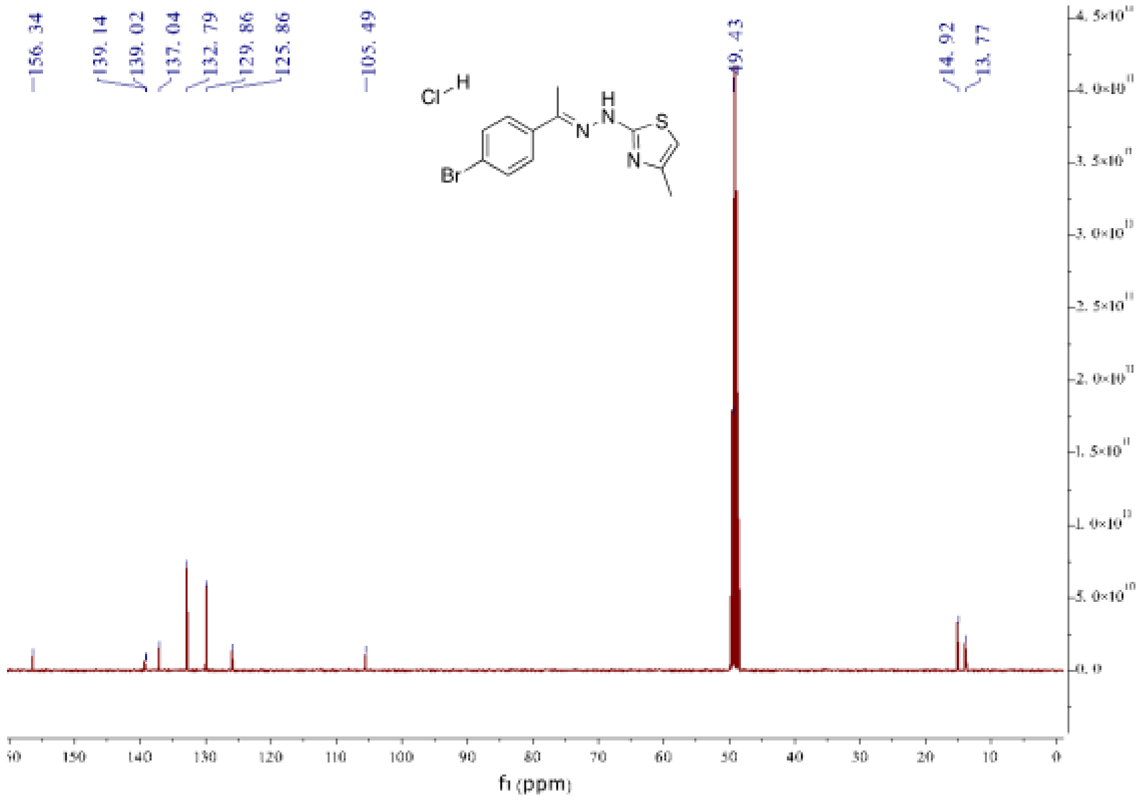

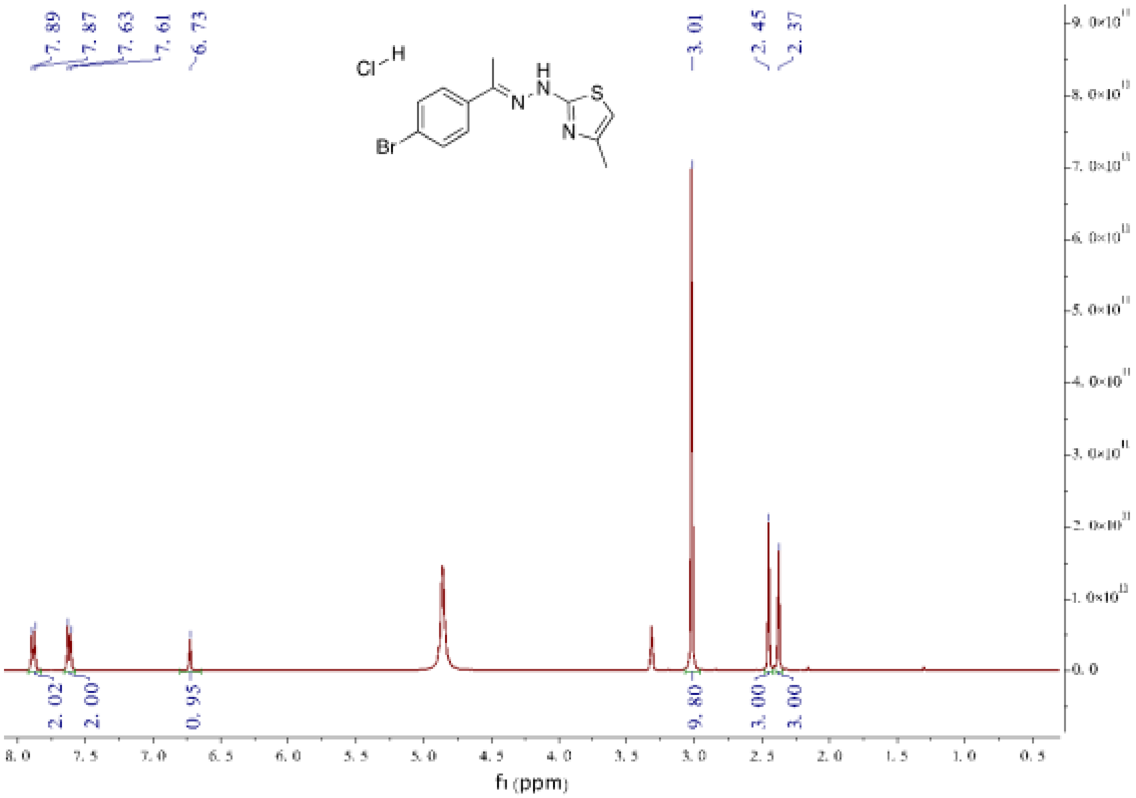

Figure S27. qNMR (up) of compound ***S*C3** and IR (down) of compound ***Se*O4**.

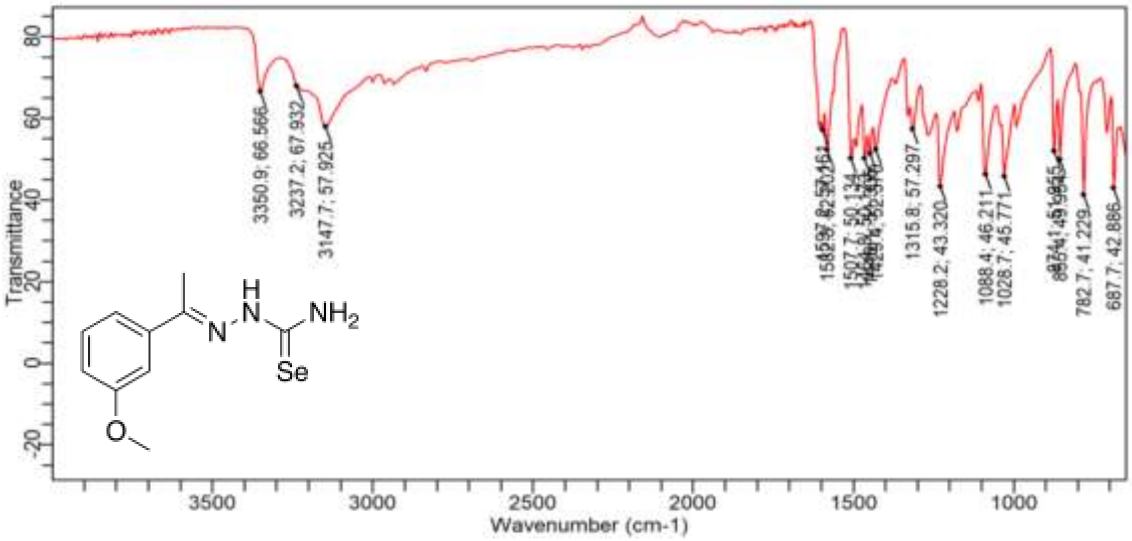

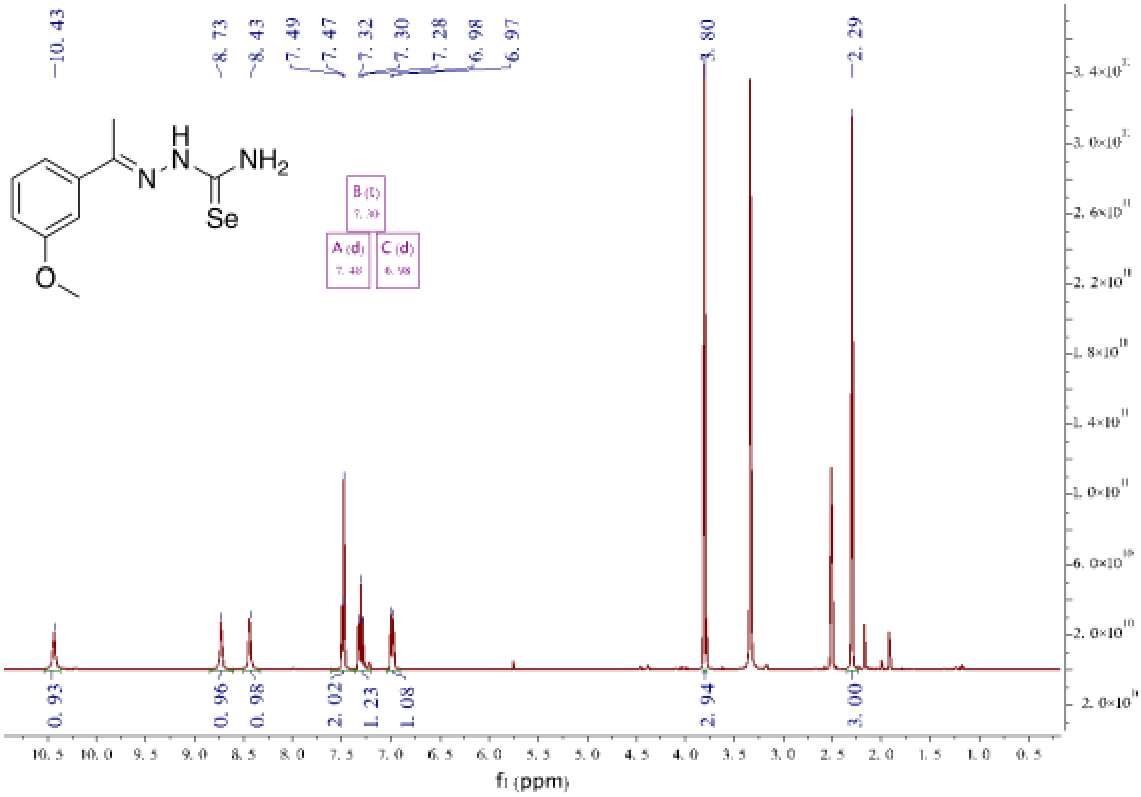

Figure S28. ^{1}H-NMR (up) and ^{13}C-NMR (down) of compound ***Se*O4**.

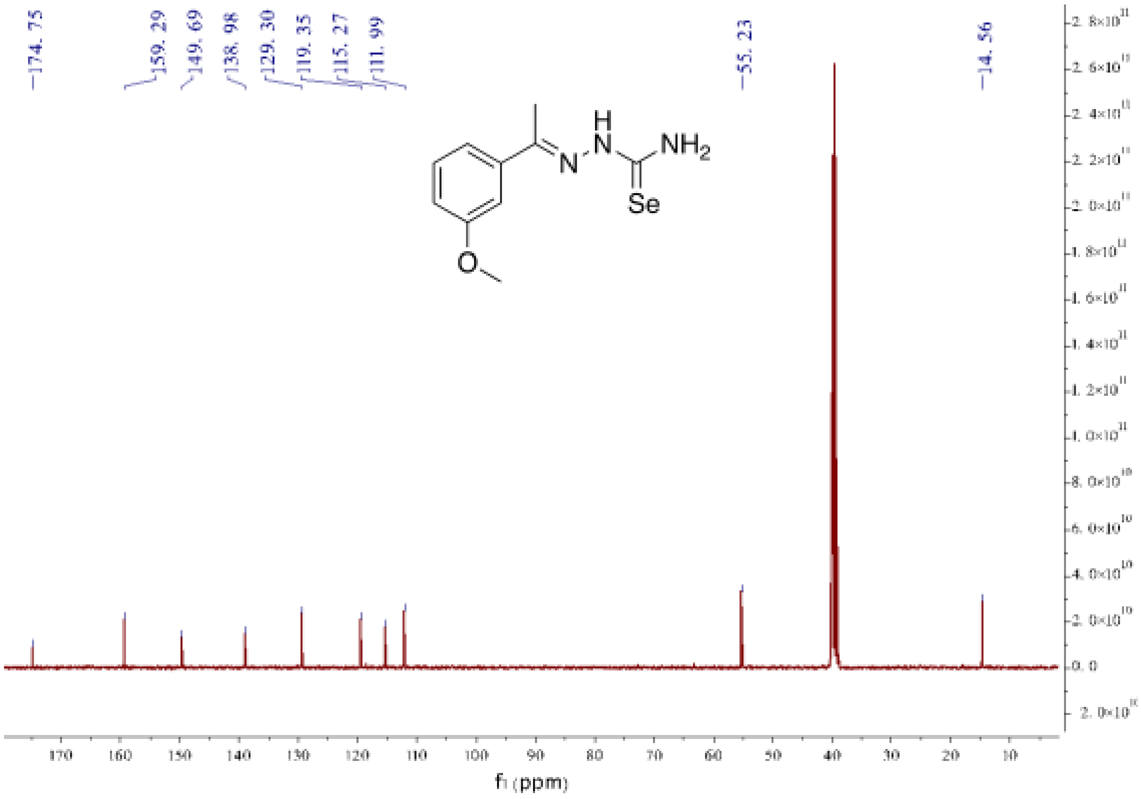

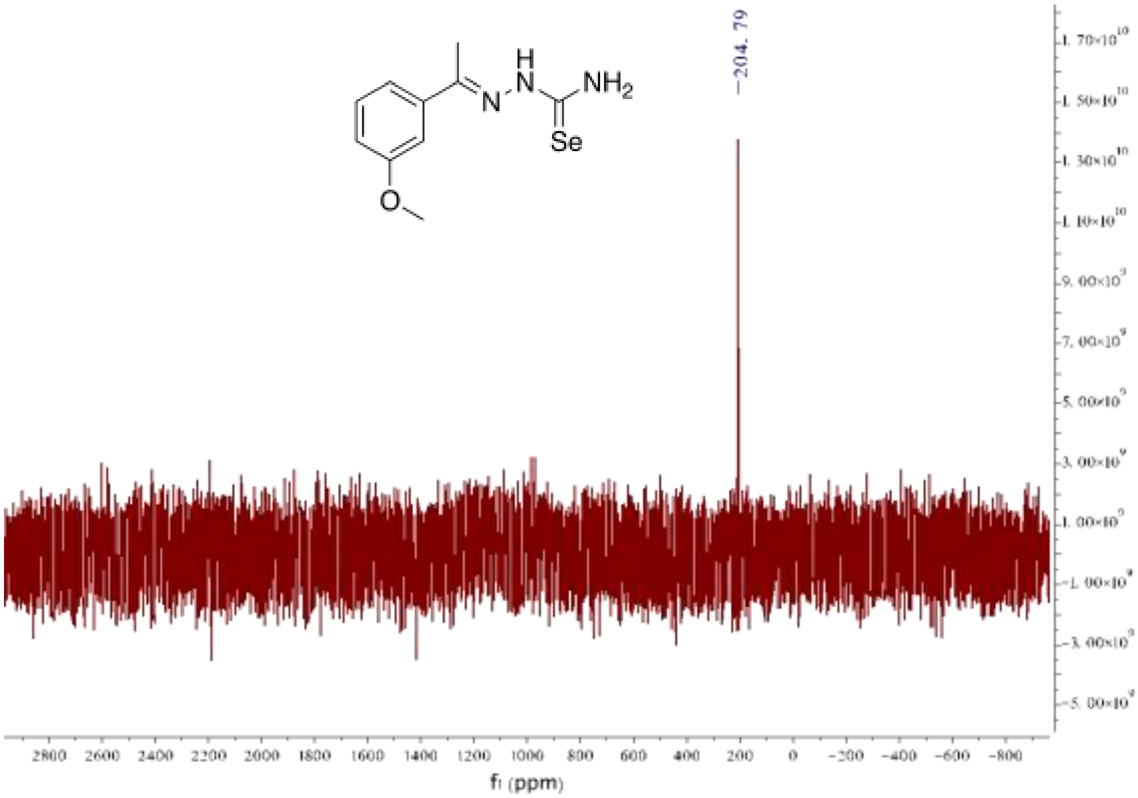

Figure S29. ^{77}Se-NMR (up) and qNMR (down) of compound ***Se*O4**.

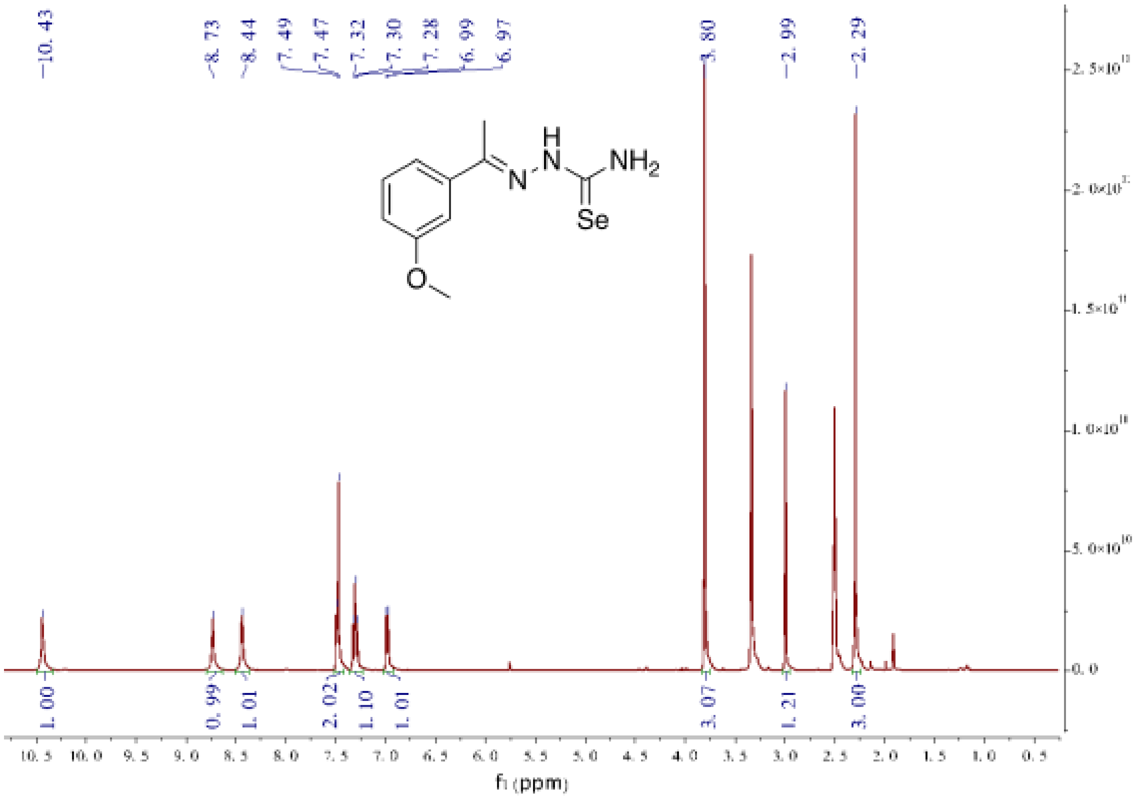

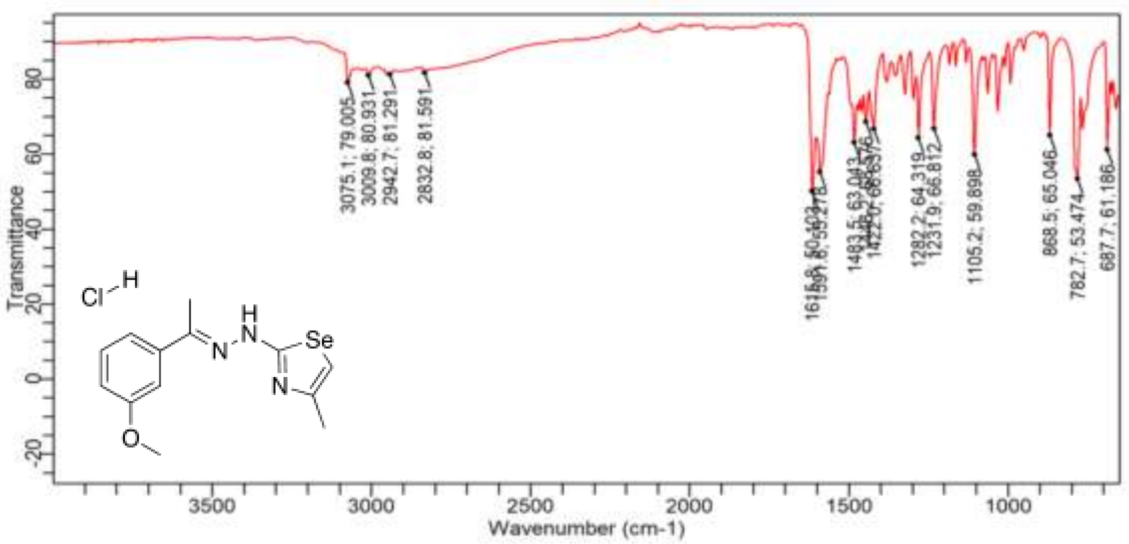

Figure S30. IR (up) and ^{1}H-NMR (down) of compound ***Se*C4**.

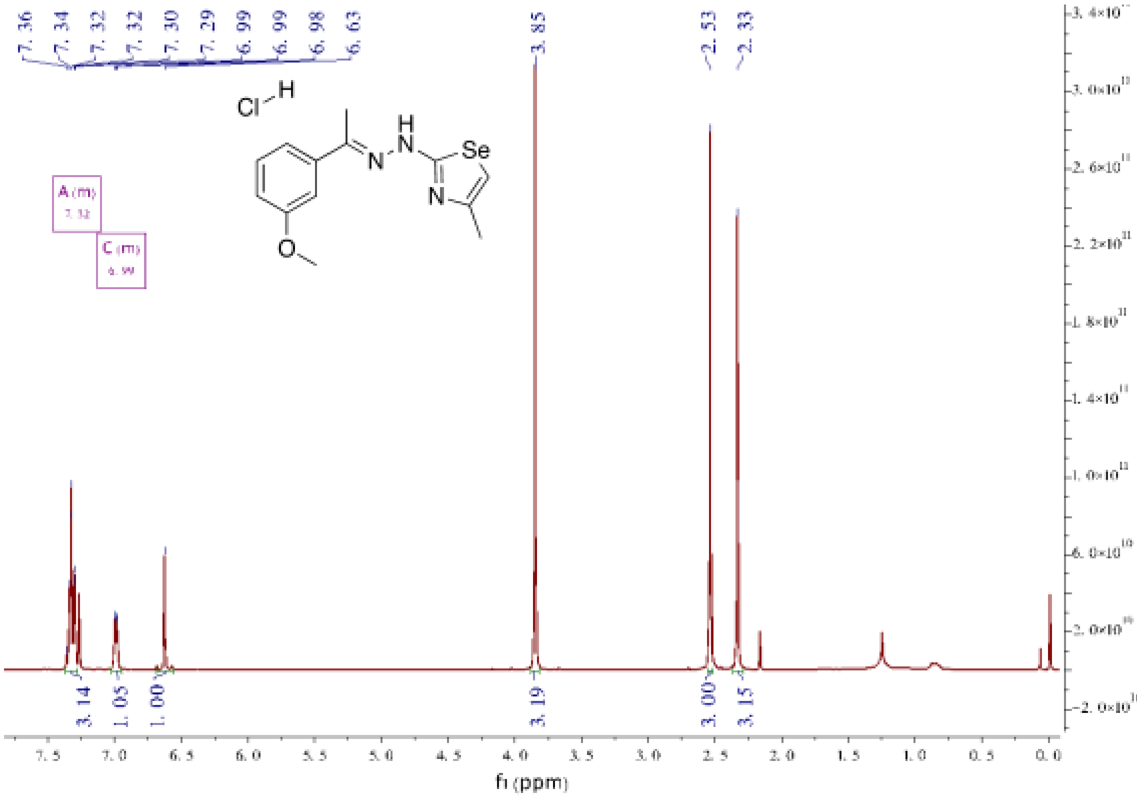

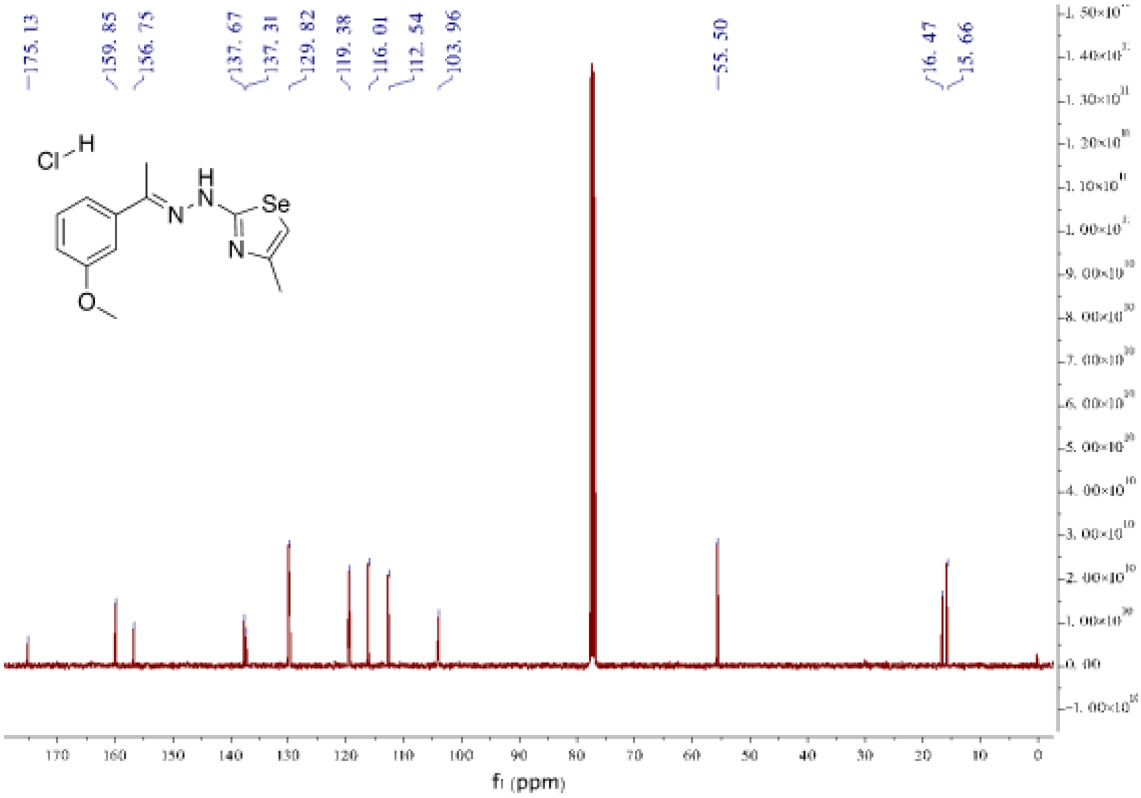

Figure S31. ^{13}C-NMR (up) and qNMR (down) of compound ***Se*C4**.

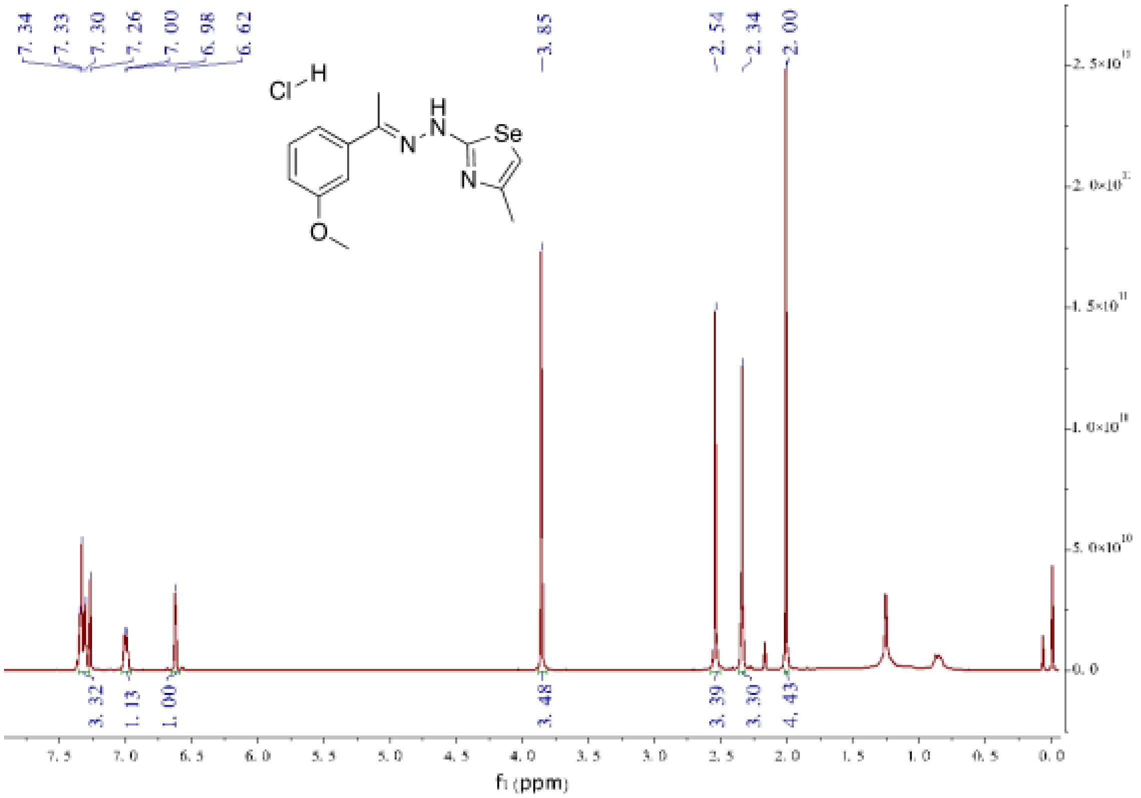

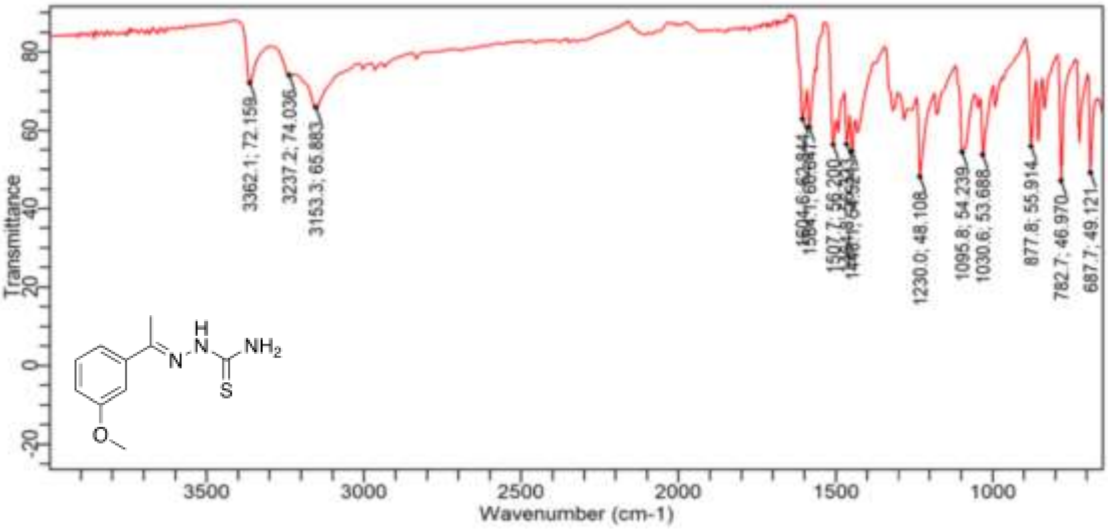

Figure S32. IR (up) and ^{1}H-NMR (down) of compound **SO4.**

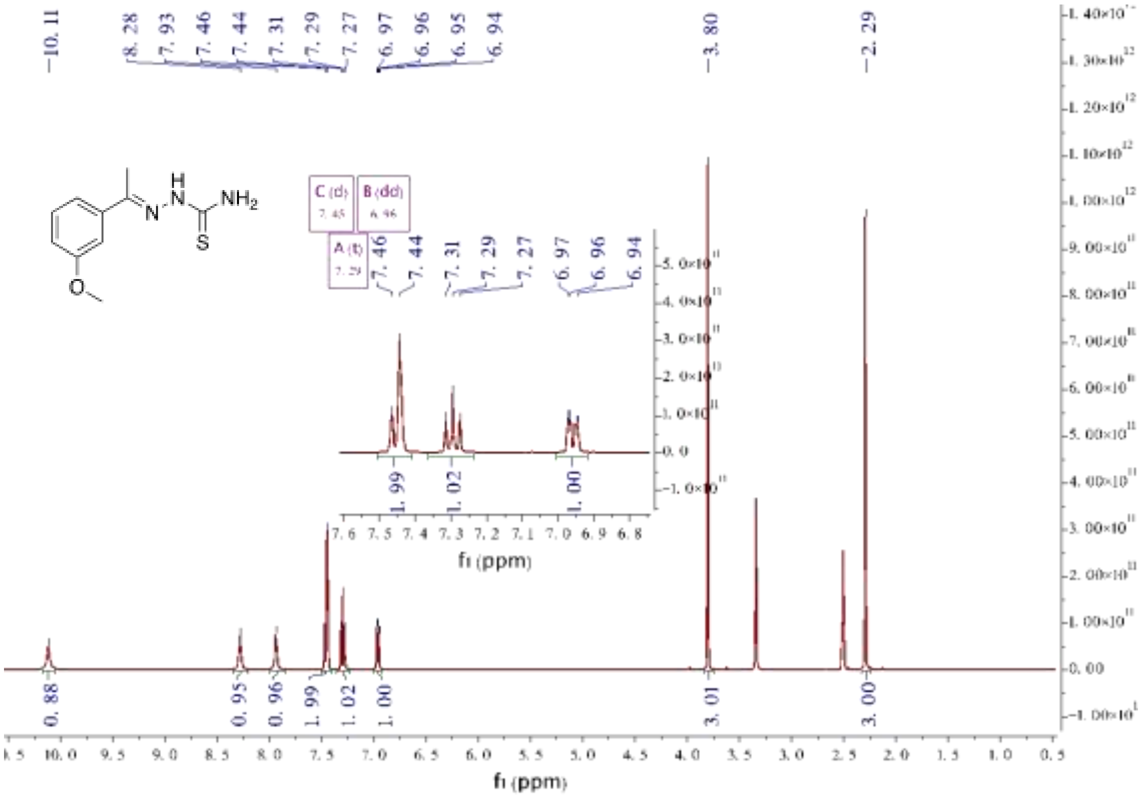

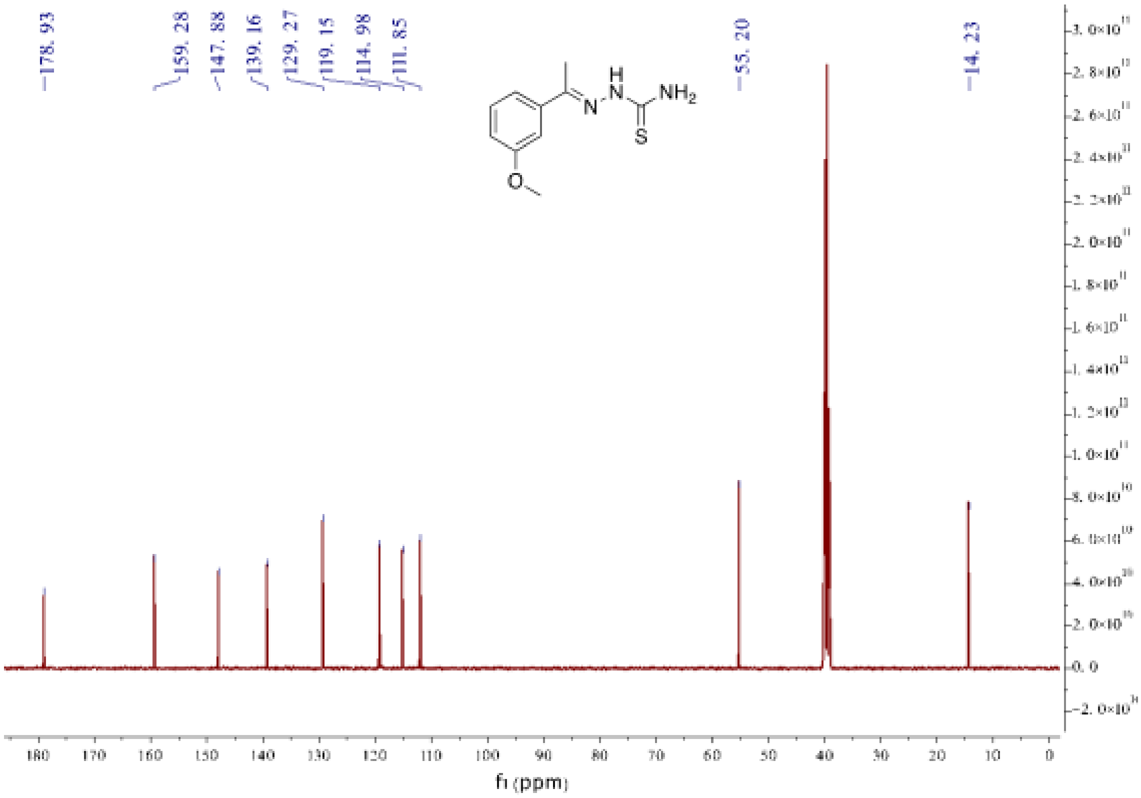

Figure S33. ^{13}C-NMR (up) and qNMR (down) of compound **SO4**.

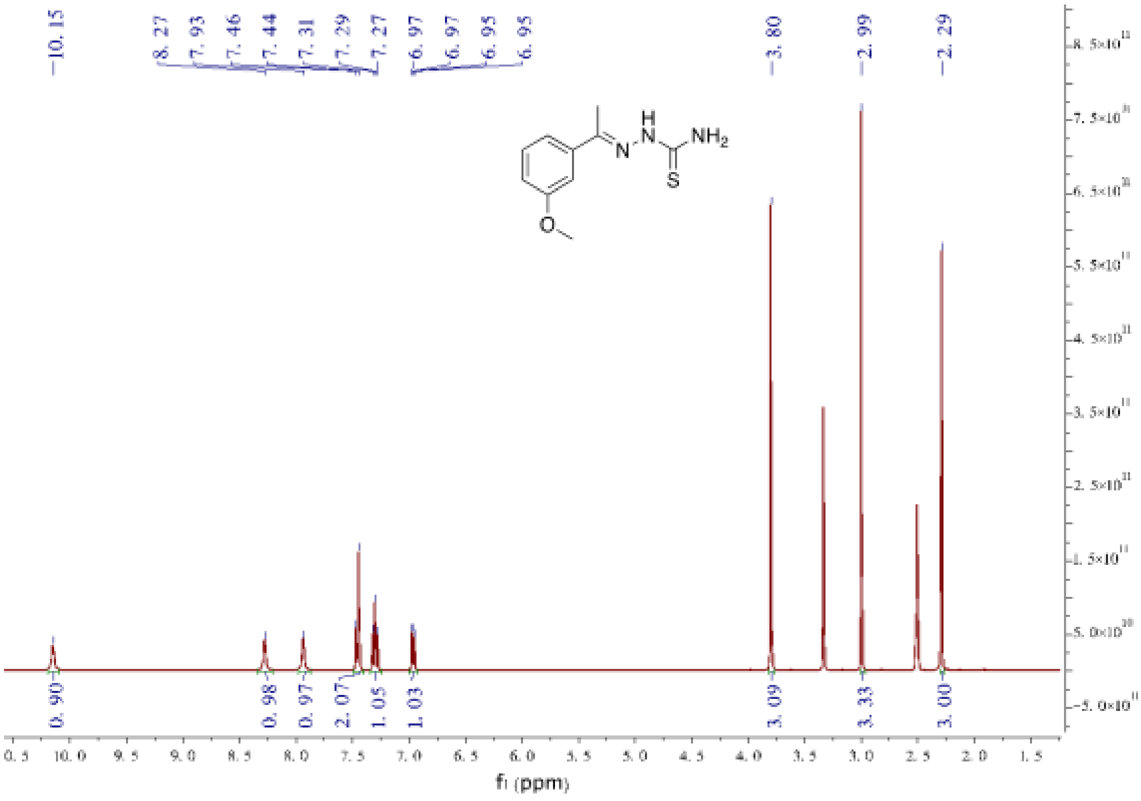

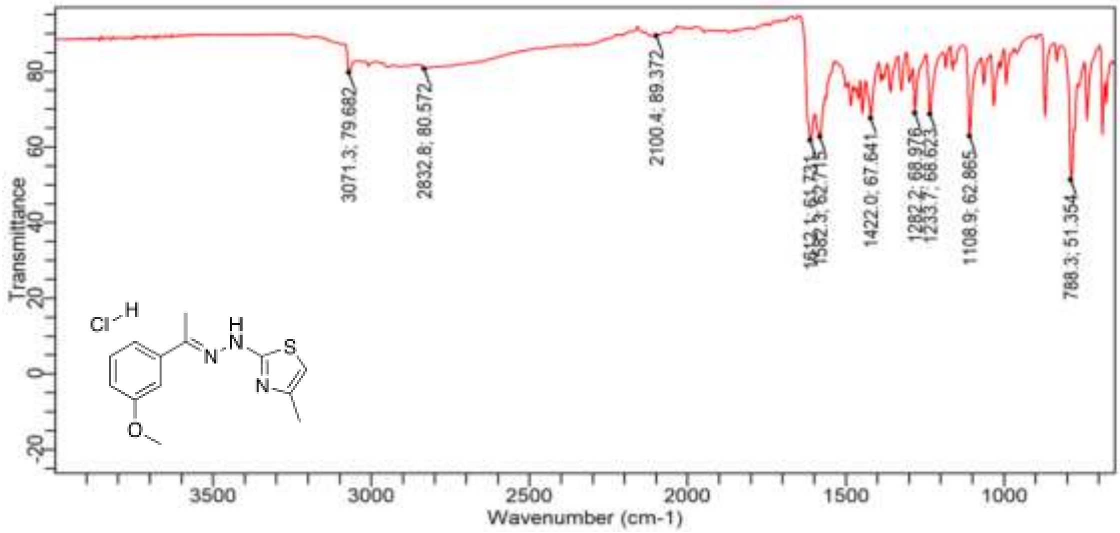

Figure S34. IR (up) and ^{1}H-NMR (down) of compound ***S*C4**.

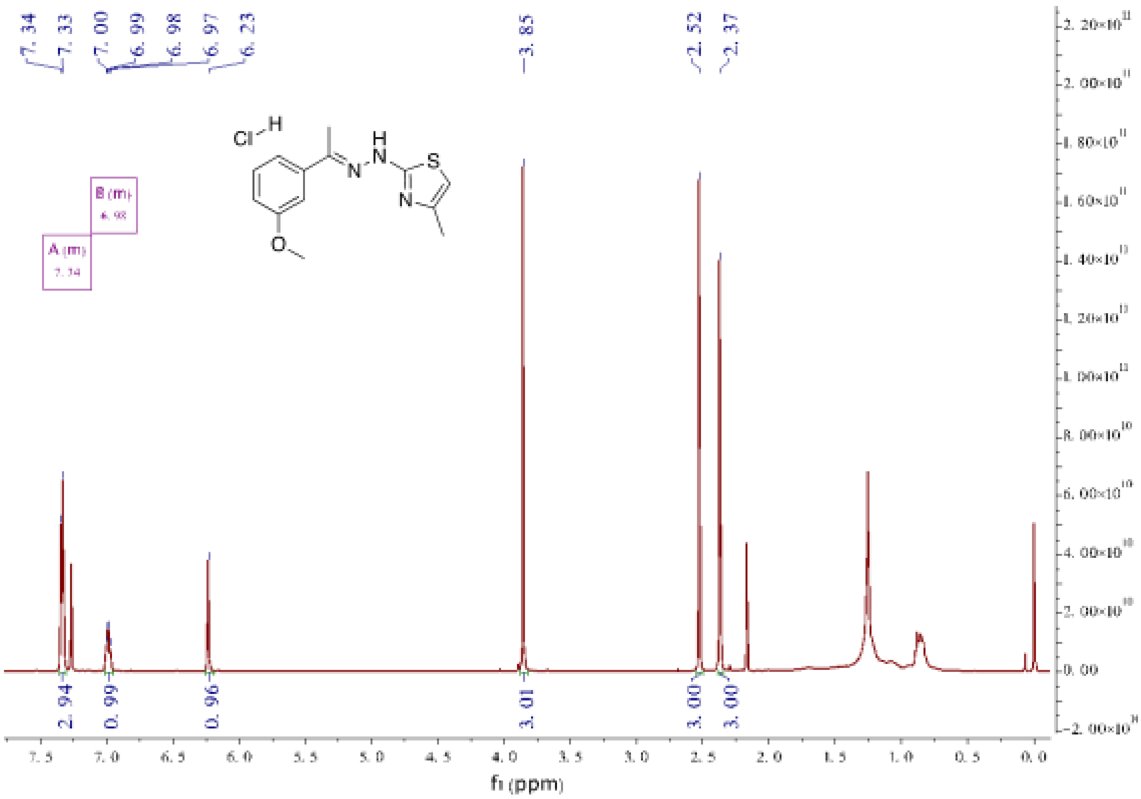

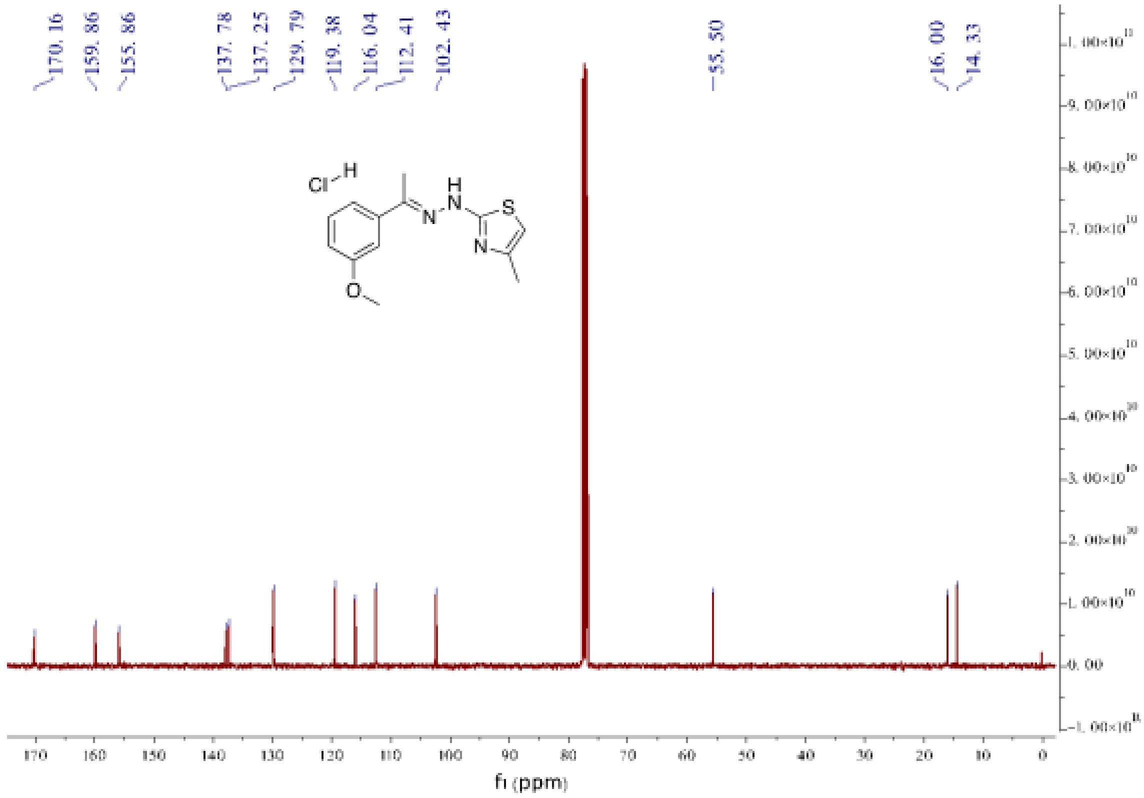

Figure S35. ^{13}C-NMR (up) and qNMR (down) of compound ***S*C4**.

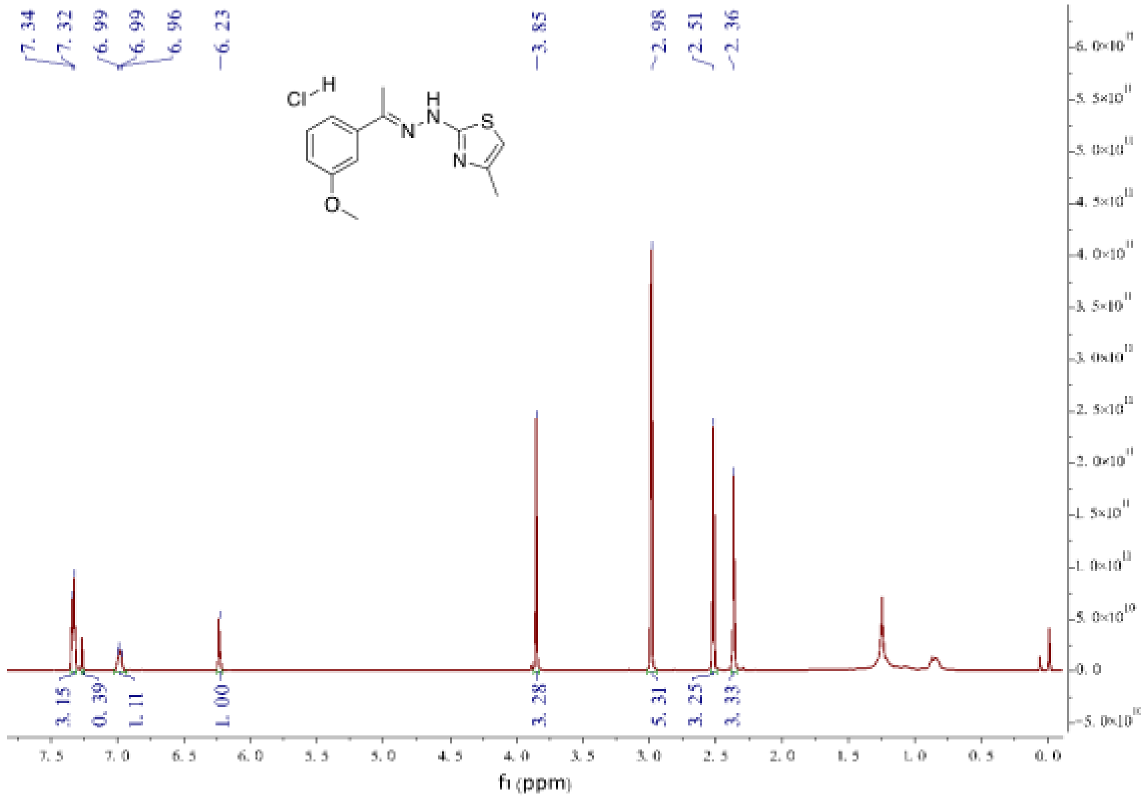

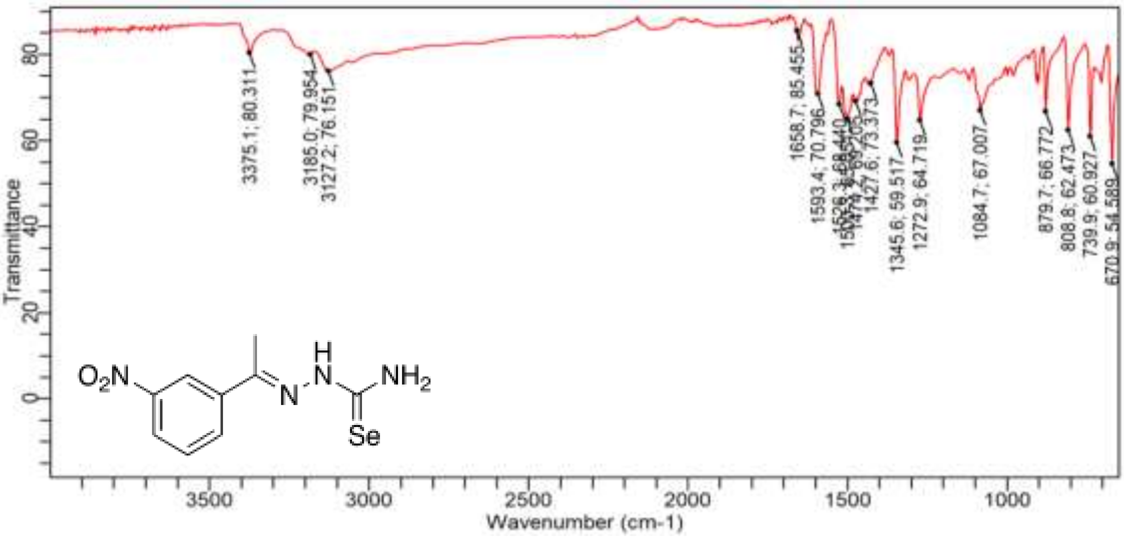

Figure S36. IR (up) of compound ***Se*O5** and ^{1}H-NMR (down) of compound ***Se*O5**.

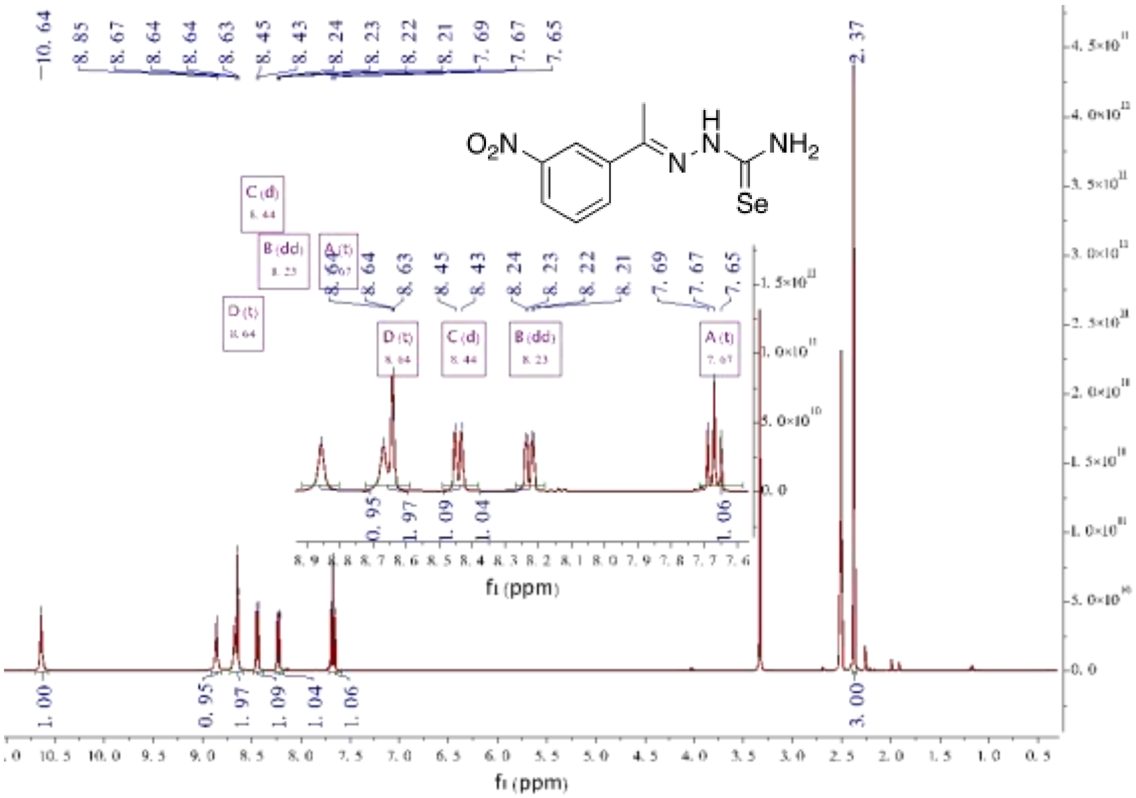

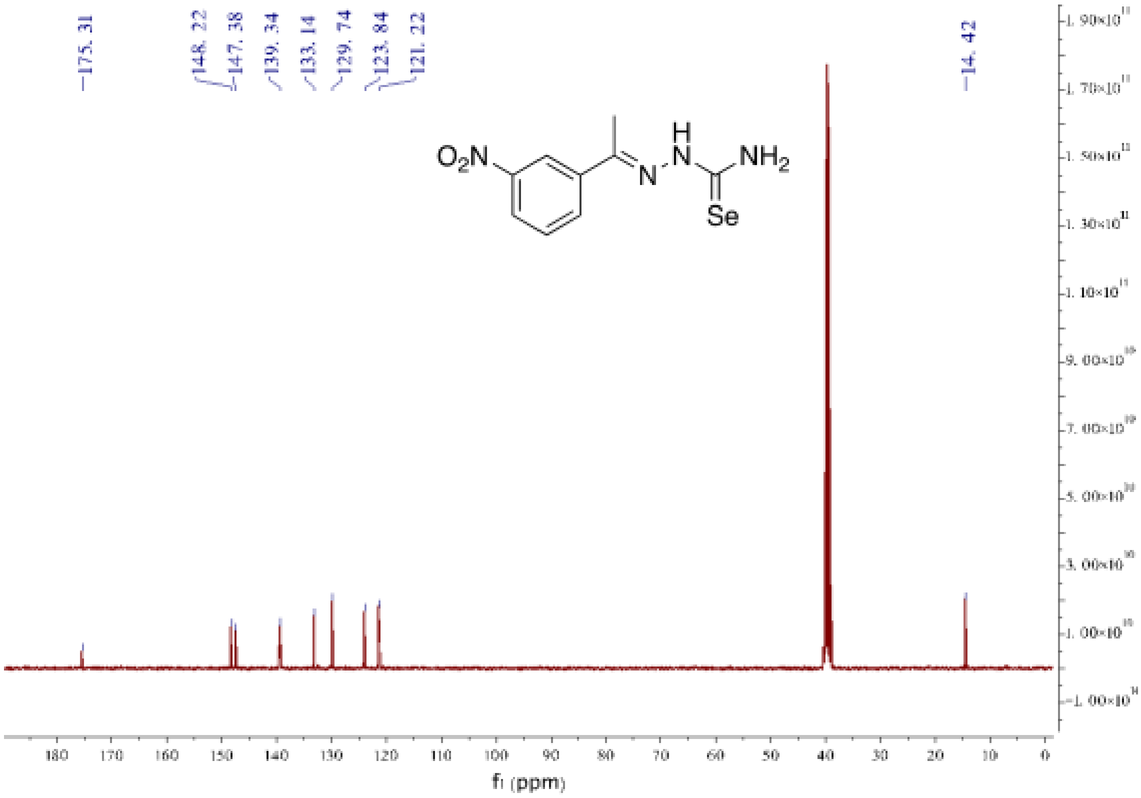

Figure S37. ^{13}C-NMR (up) and ^{77}Se-NMR (down) of compound ***Se*O5**.

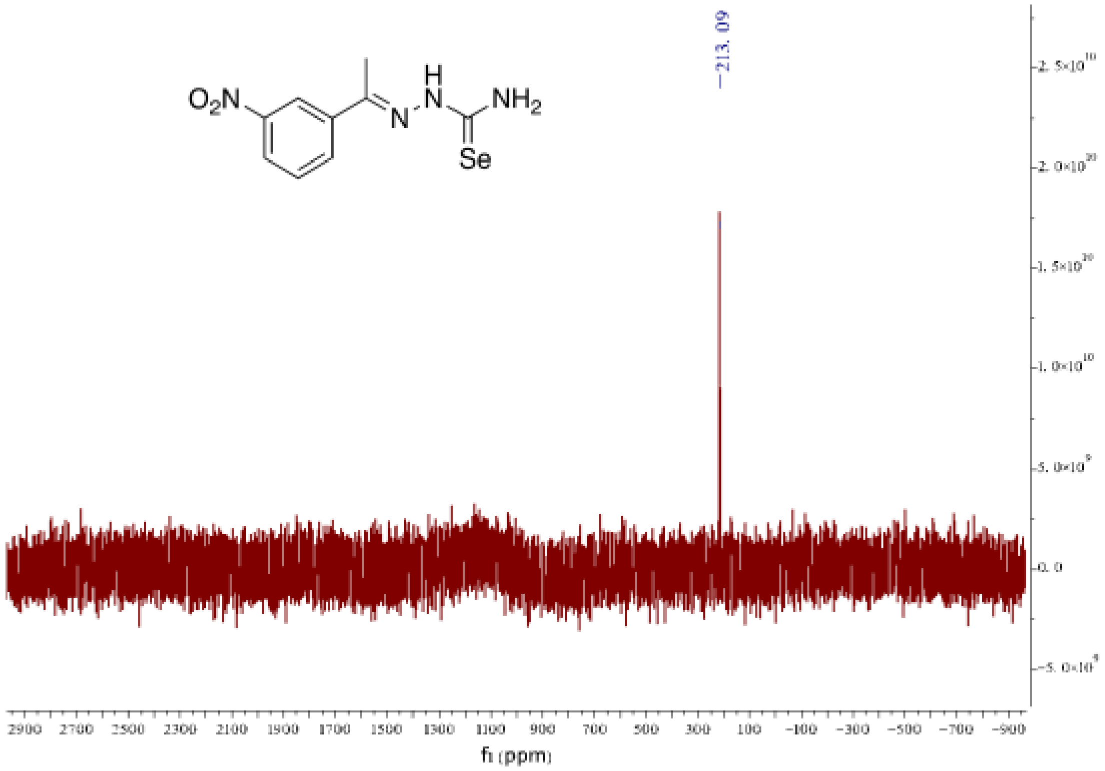

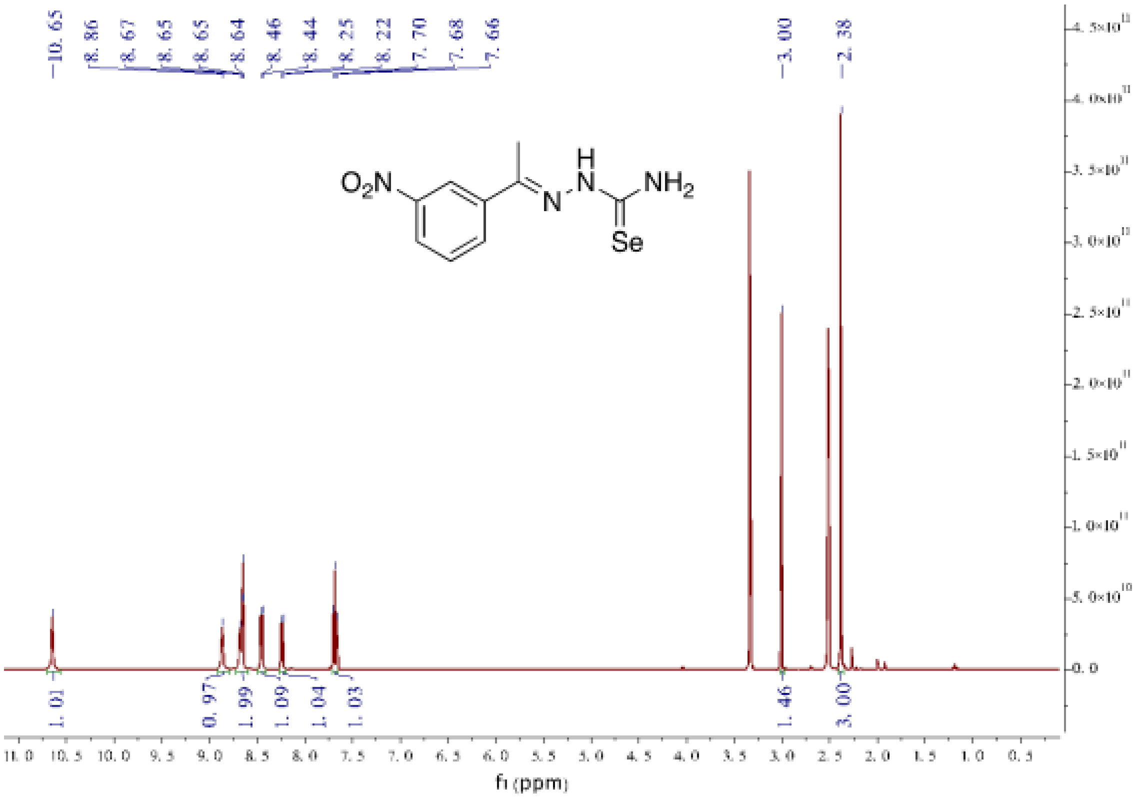

Figure S38. qNMR (up) of compound ***Se*O5** and IR (up) of compound ***Se*C5**.

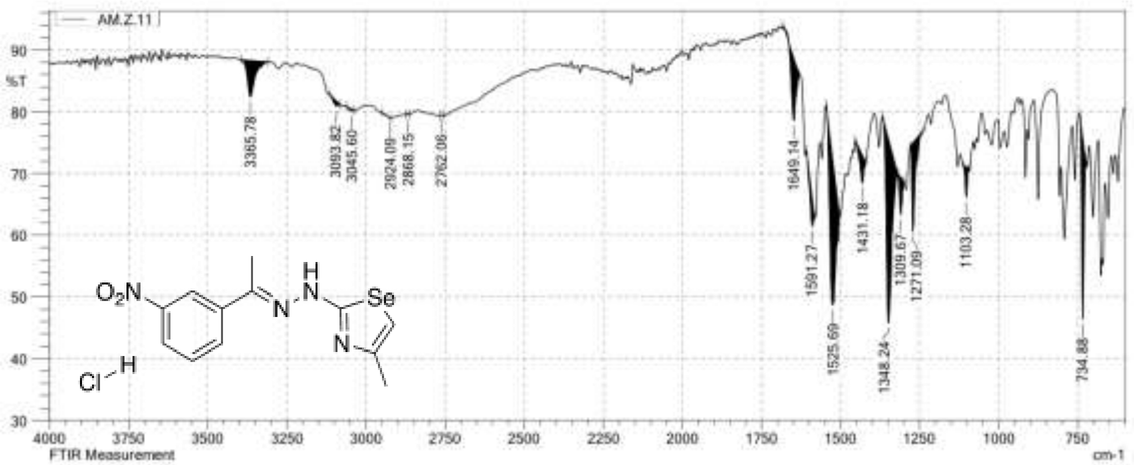

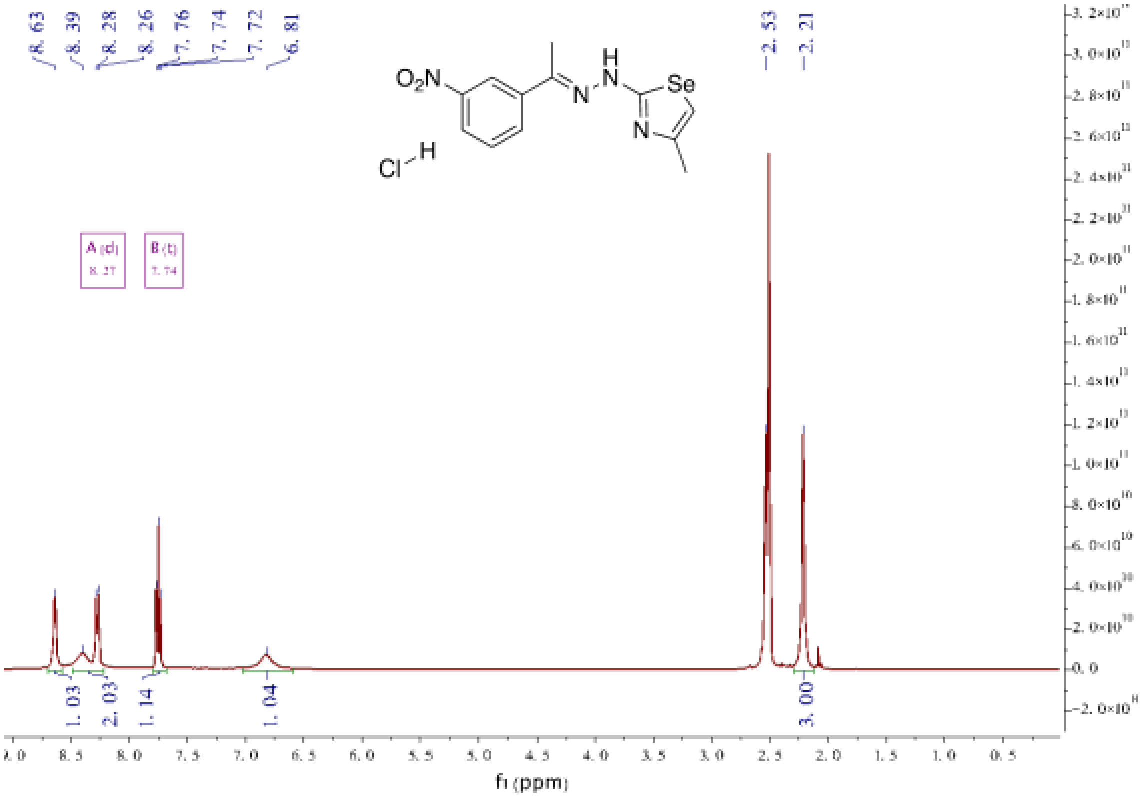

Figure S39. ^{1}H-NMR (up) and ^{13}C-NMR (down) of compound ***Se*C5**.

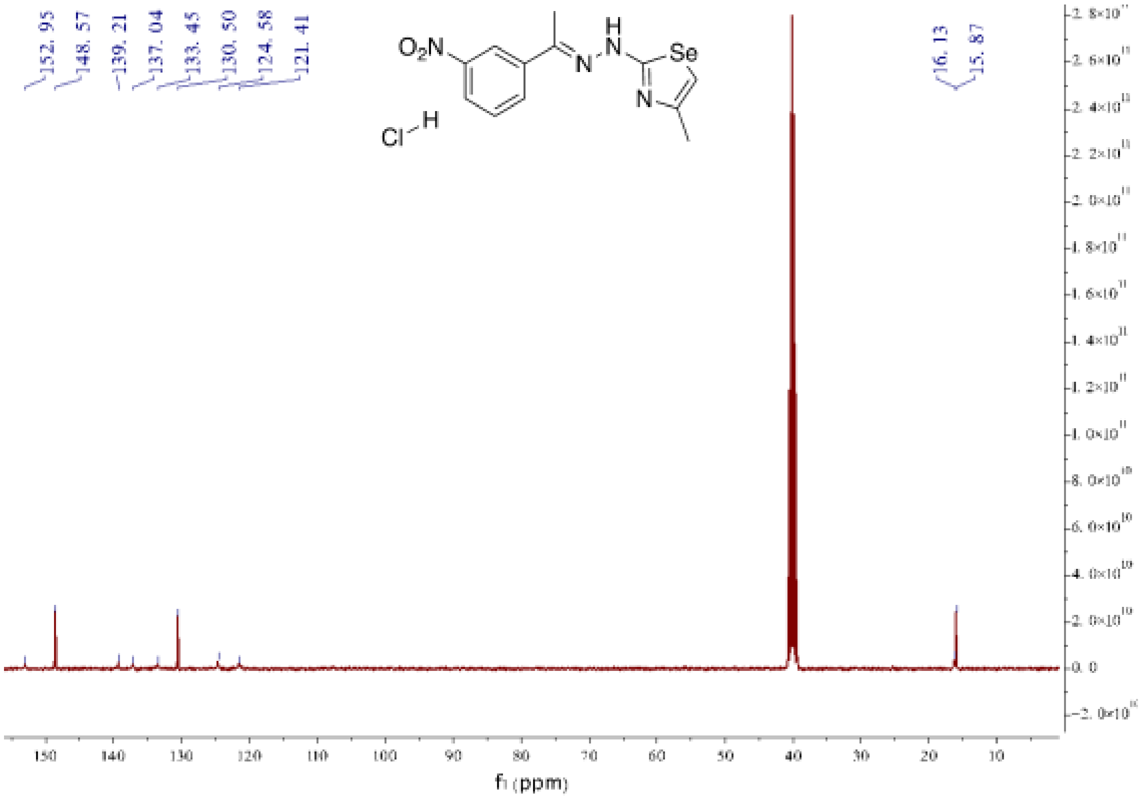

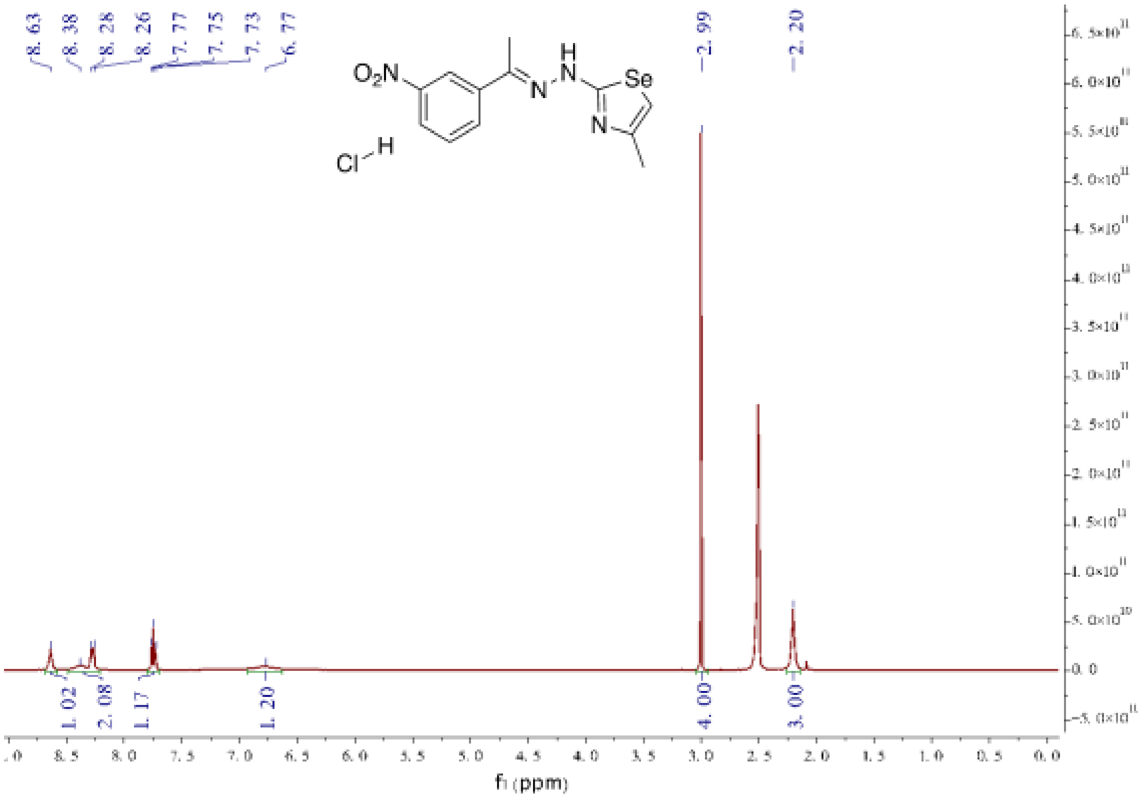

Figure S40. qNMR (up) of compound ***Se*C5** and IR (down) compound ***S*O5**.

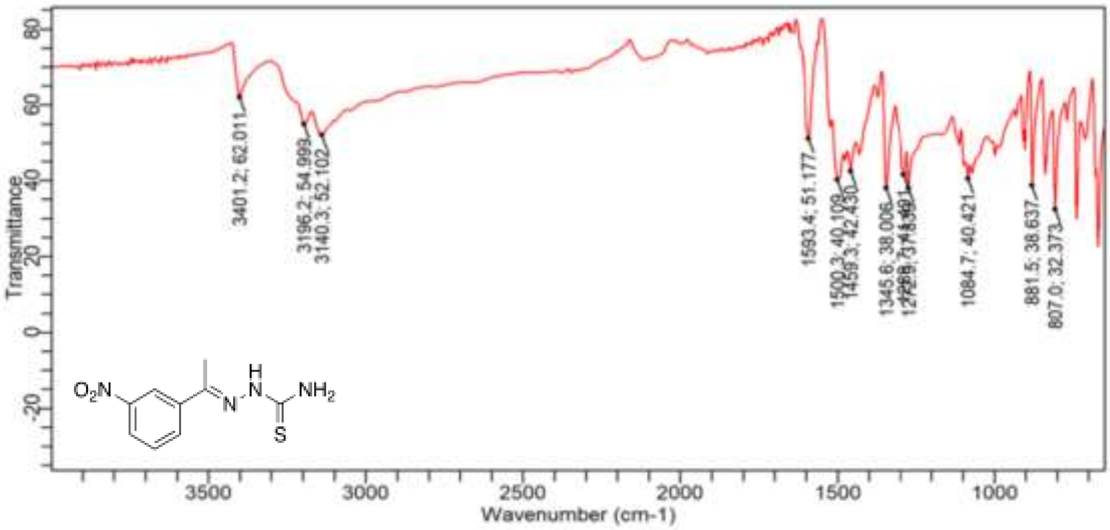

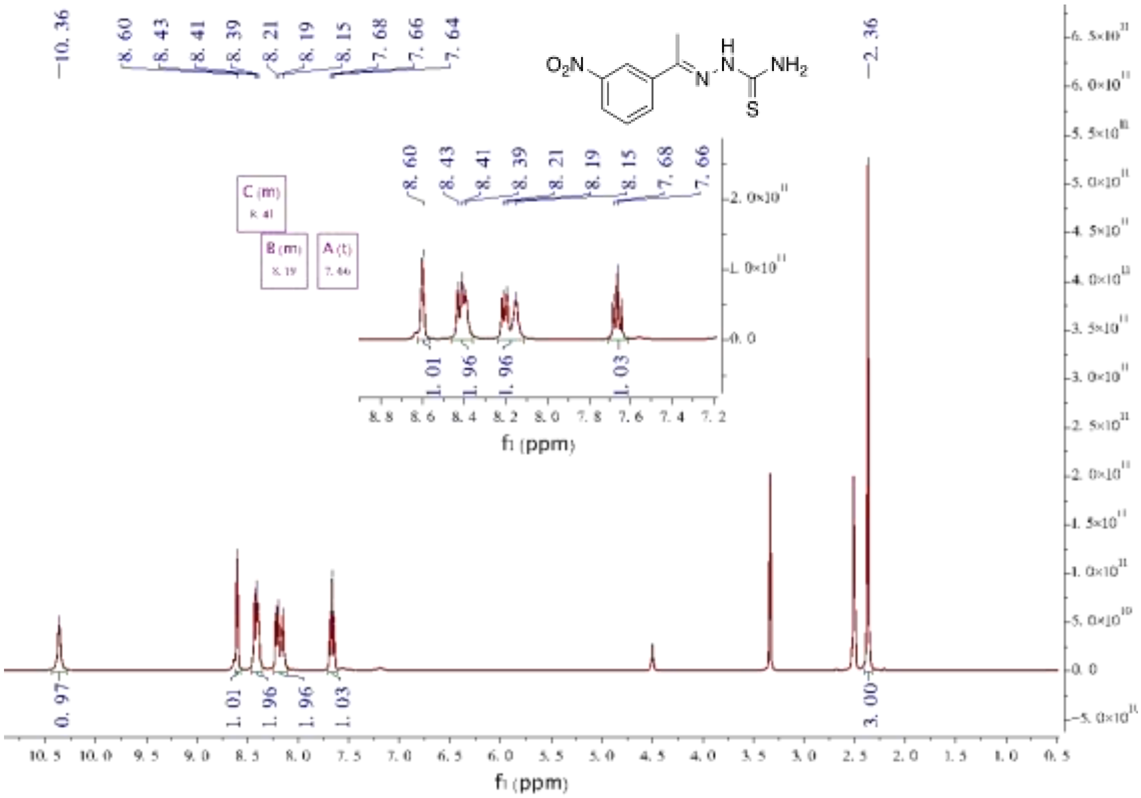

Figure S41. ^{1}H-NMR (up) and ^{13}C-NMR (down) of compound **SO5**.

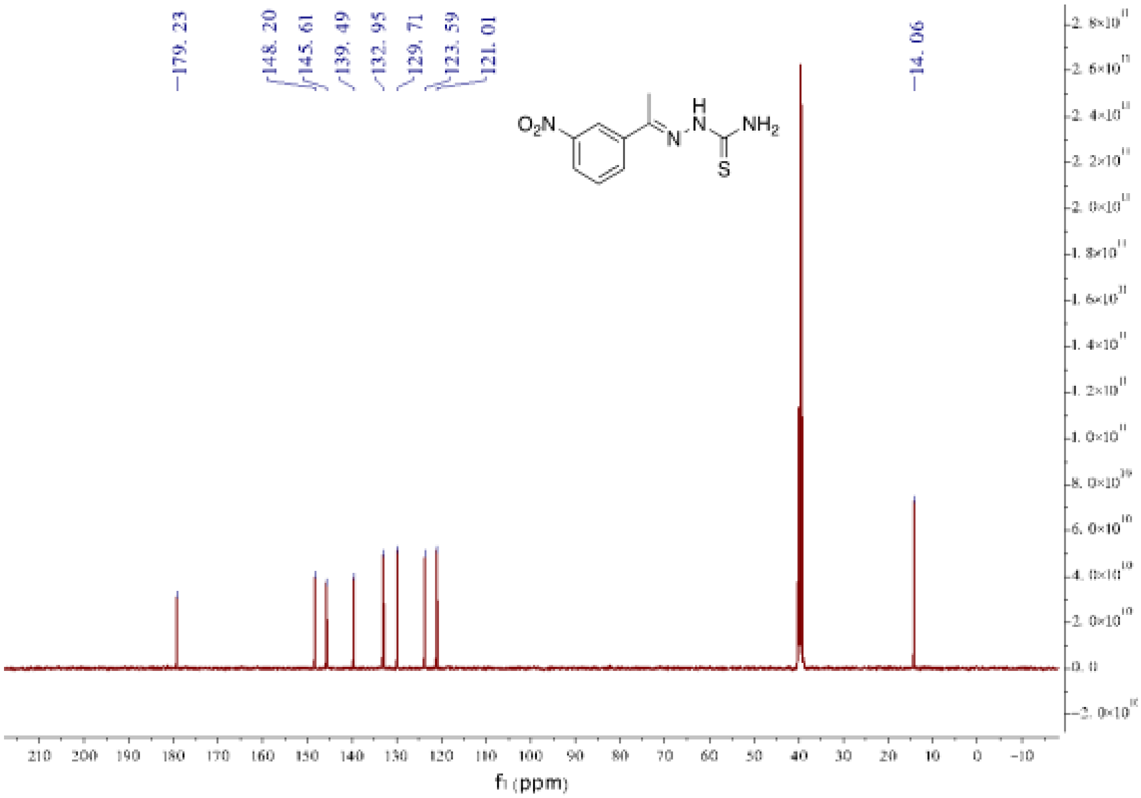

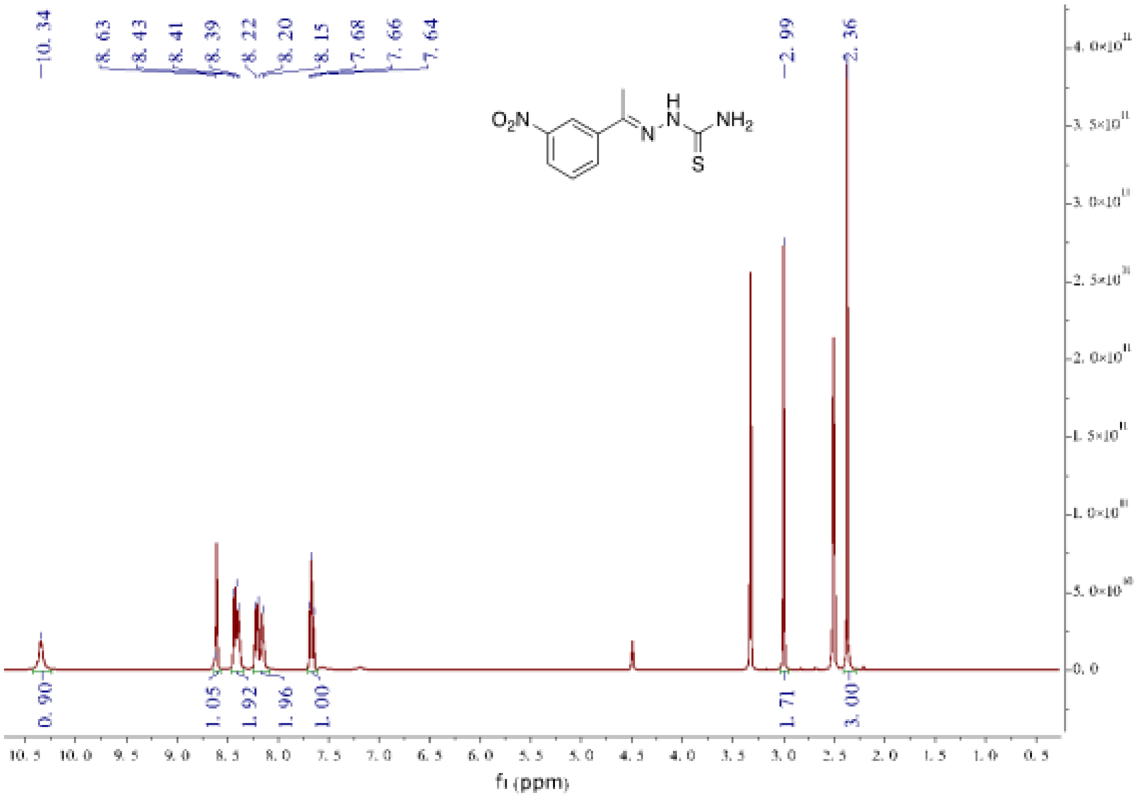

Figure S42. qNMR (down) of compound **SO5** and IR (down) of compound ***S*C5**.

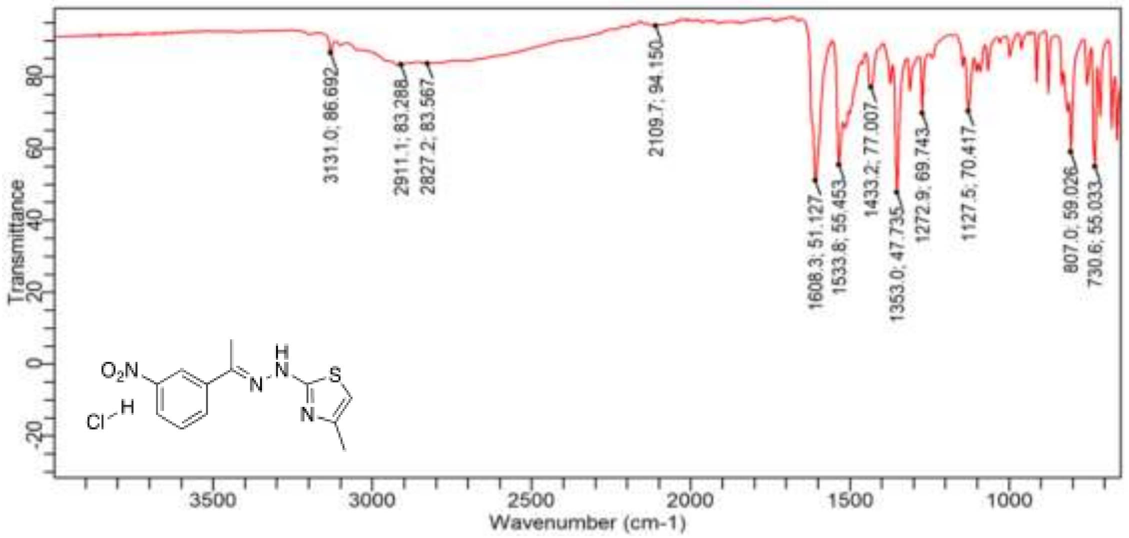

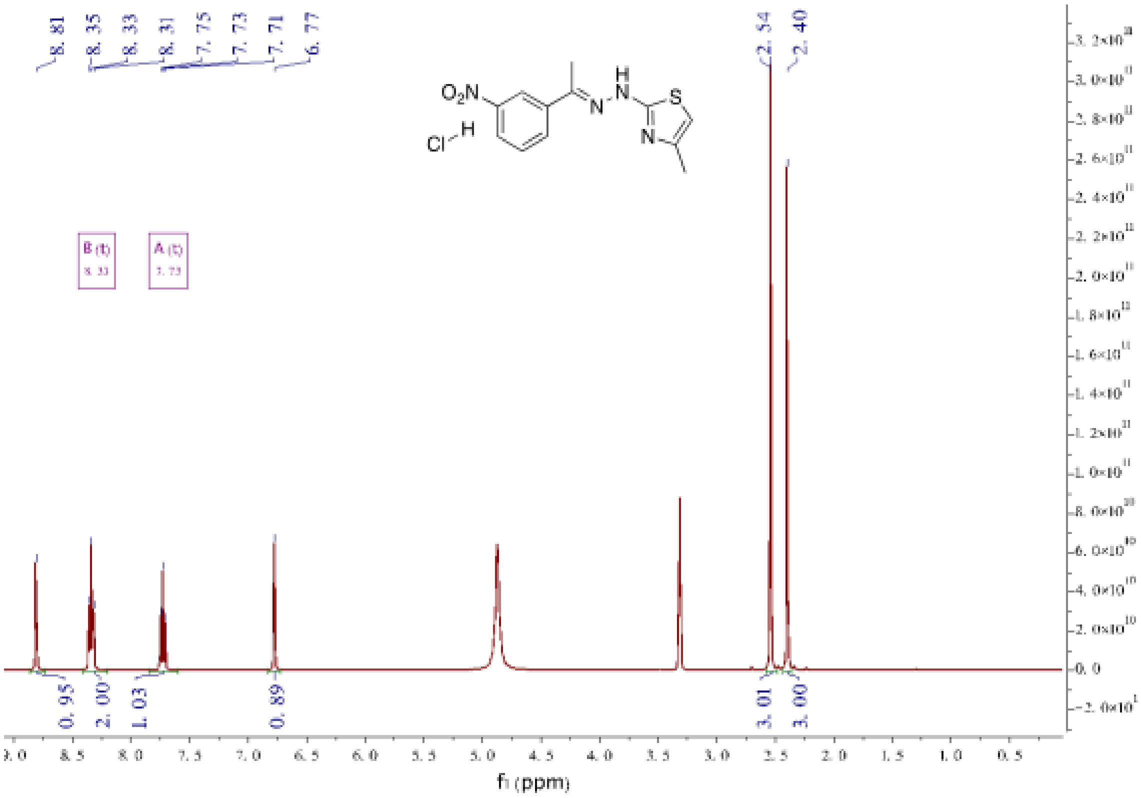

Figure S43. ^{1}H-NMR (down) and ^{13}C-NMR (up) of compound ***S*C5**.

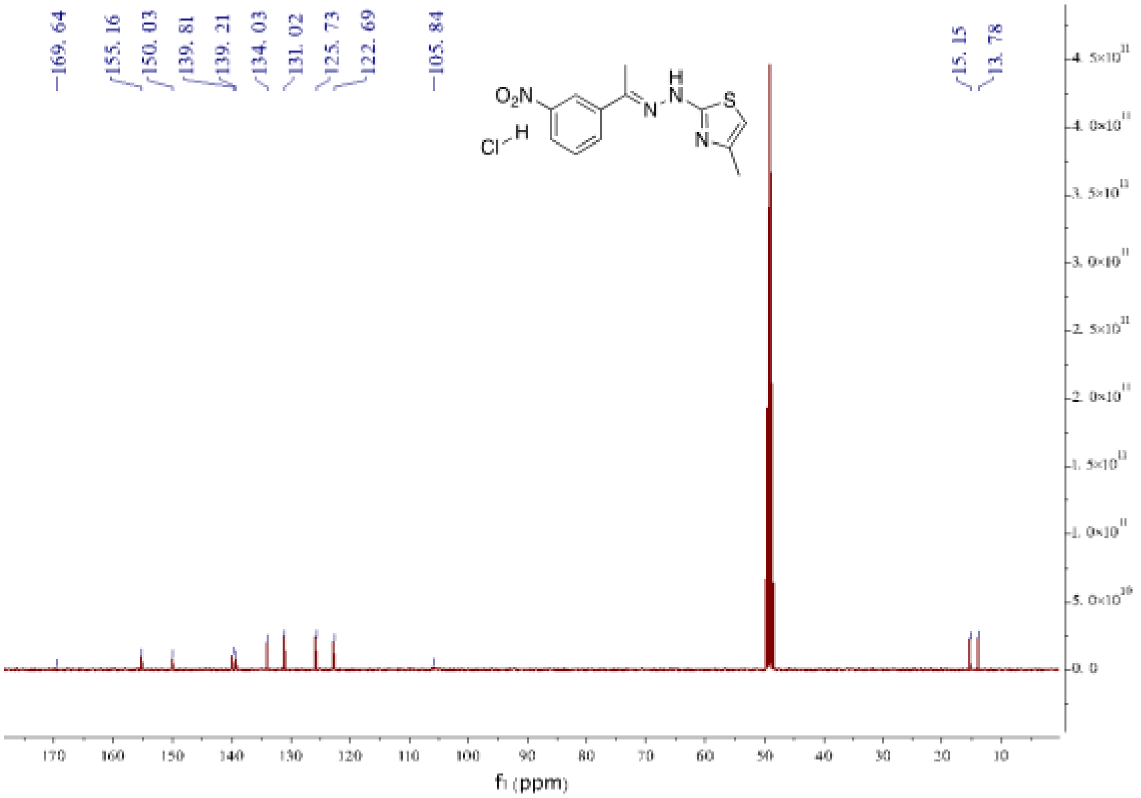

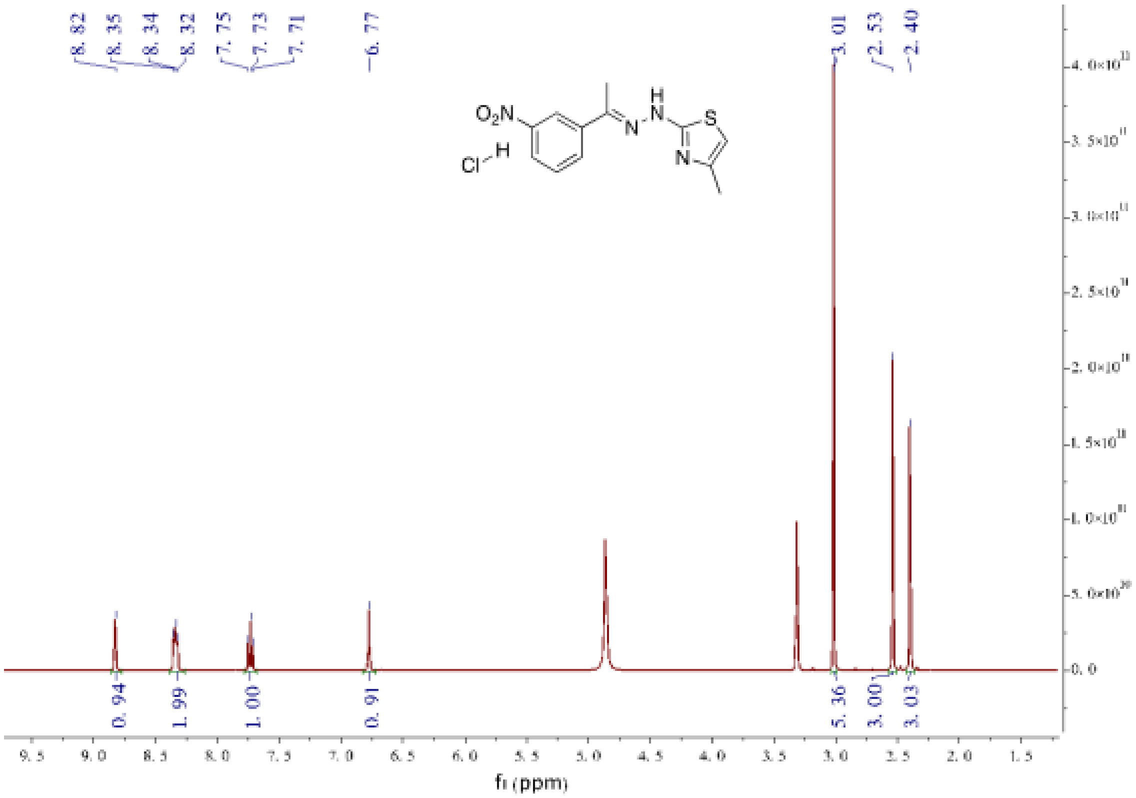

Figure S44. qNMR (up) of compound ***S*C5** and IR (down) of compound ***Se*O6**.

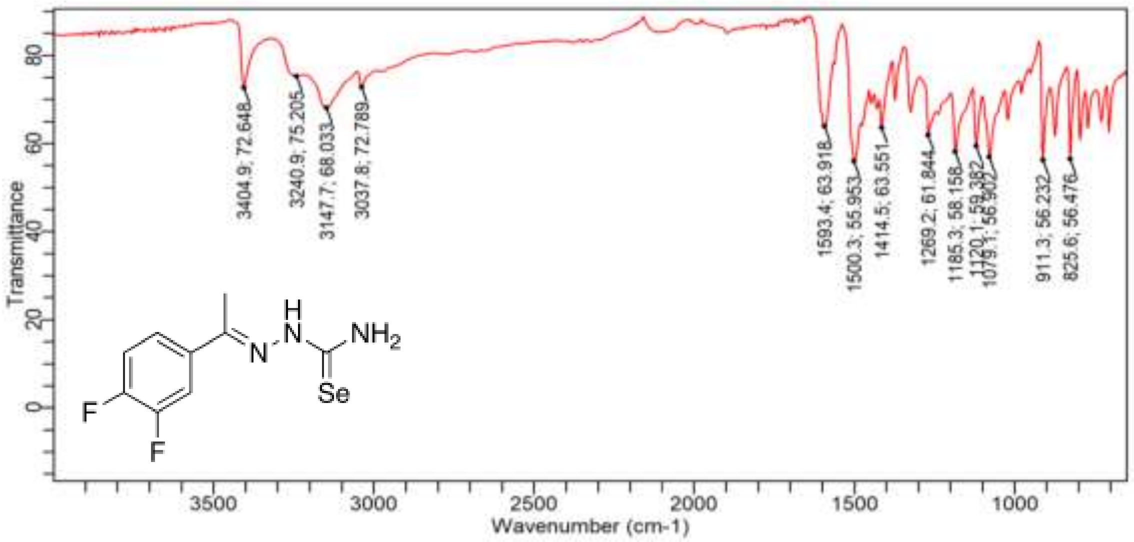

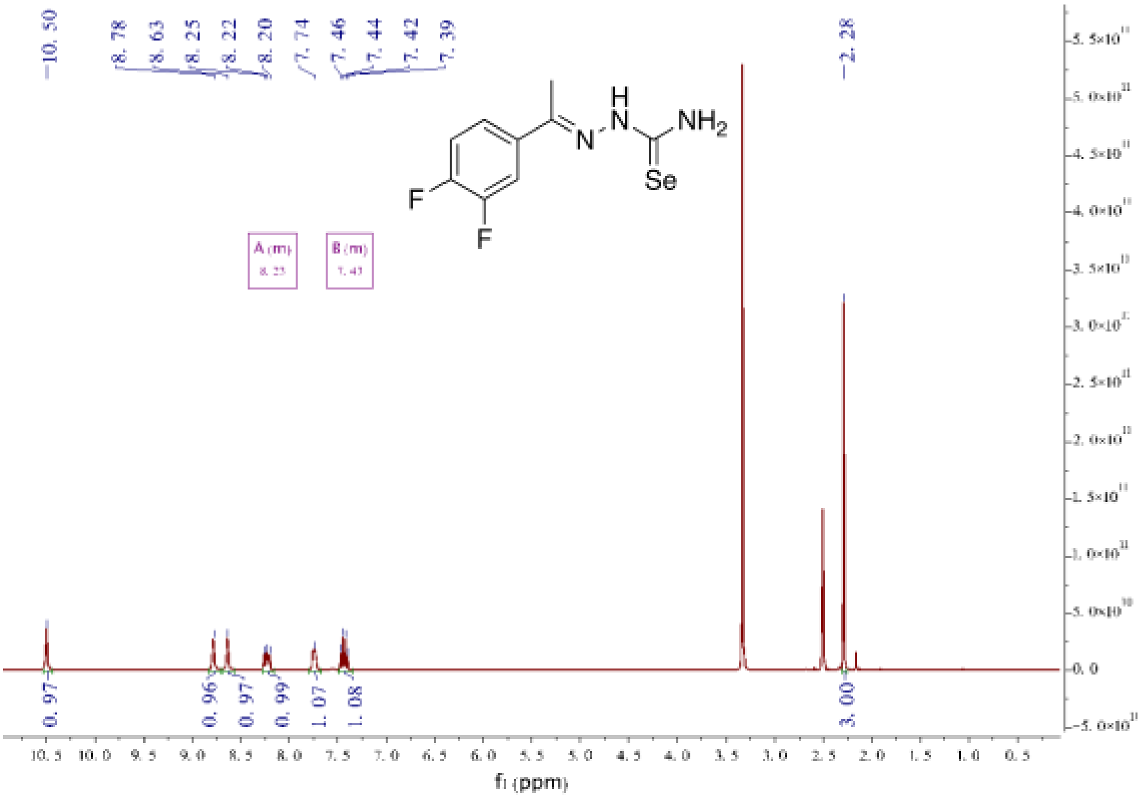

Figure S45. ^{1}H-NMR (up) and ^{13}C-NMR (down) of compound ***Se*O6**.

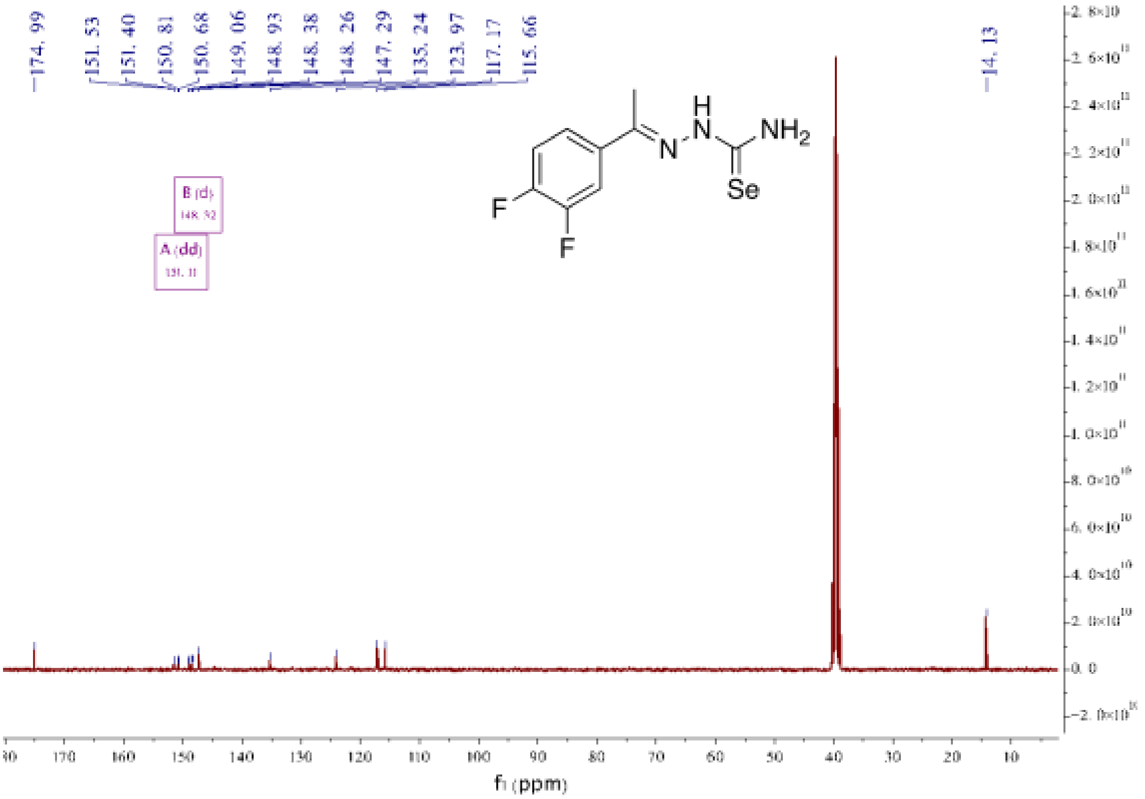

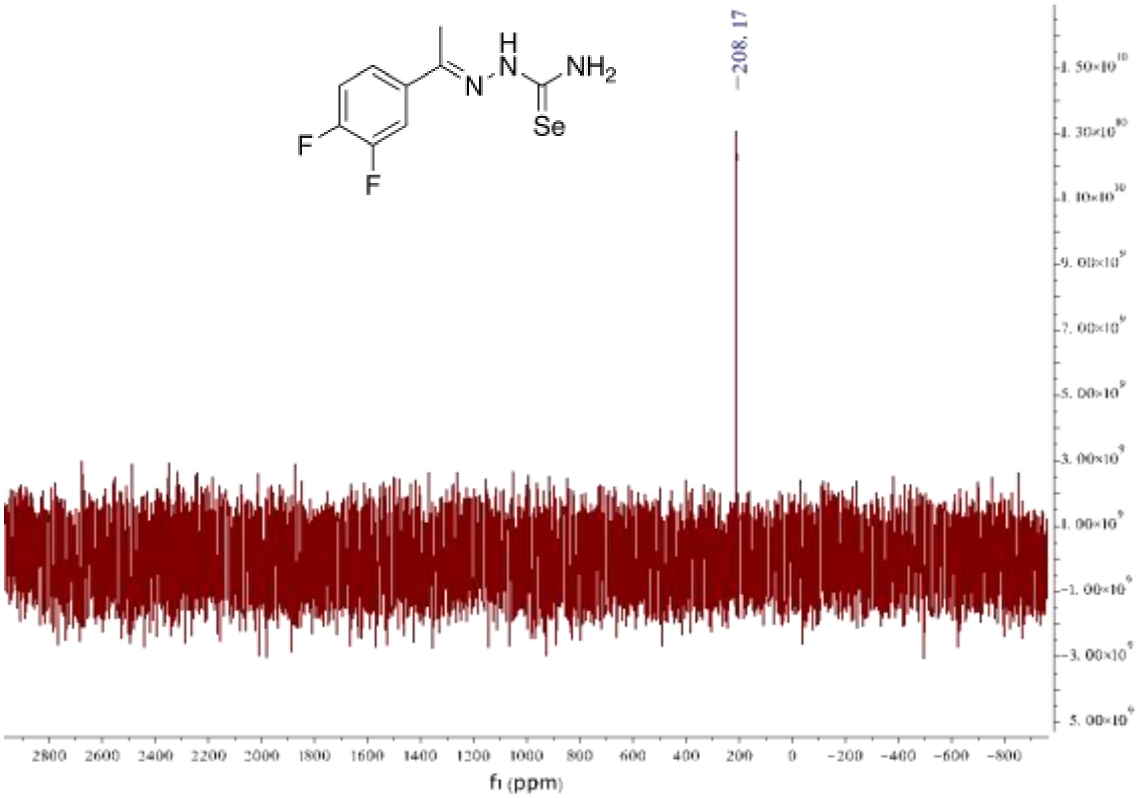

Figure S46. ^{77}Se-NMR (up) and qNMR (down) of compound ***Se*O6**.

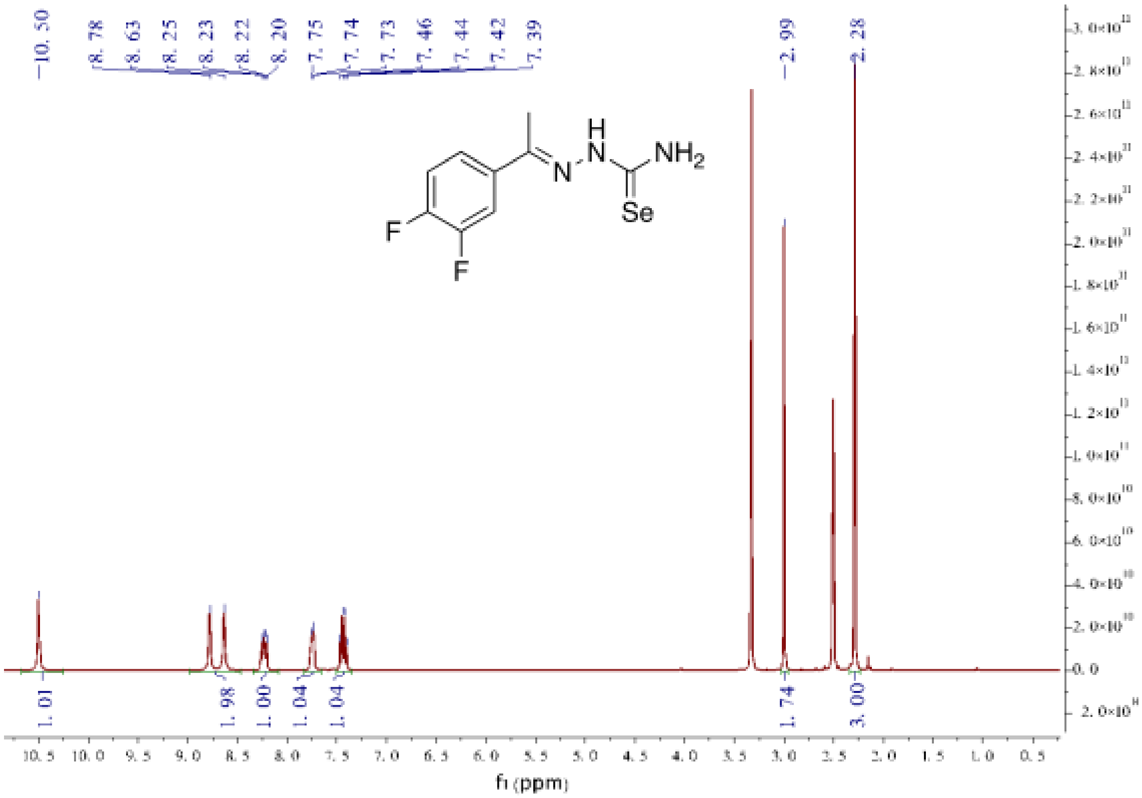

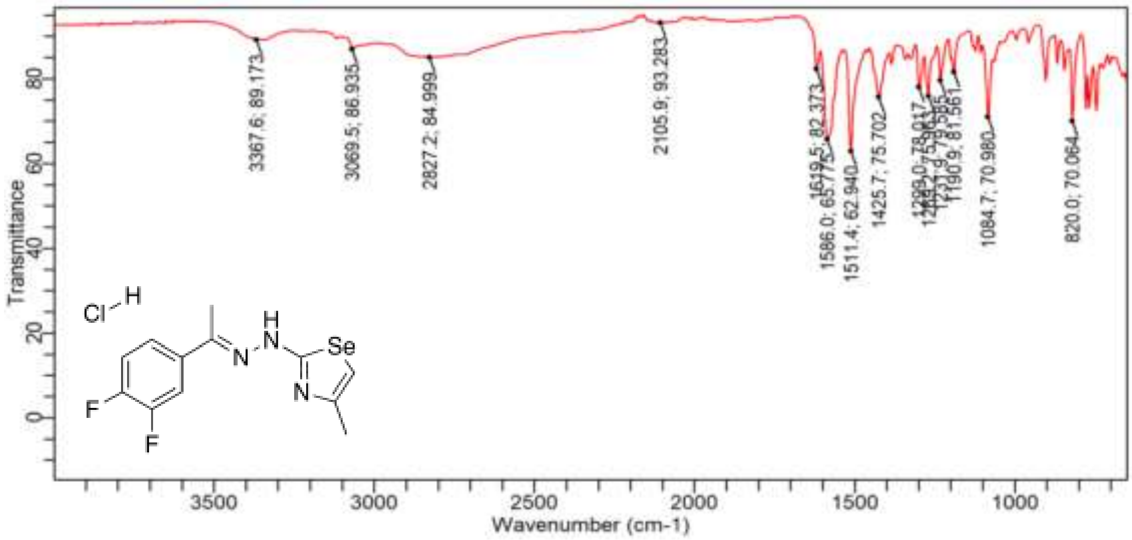

Figure S47. IR (up) and ^{1}H-NMR (down) of compound ***Se*****C6**.

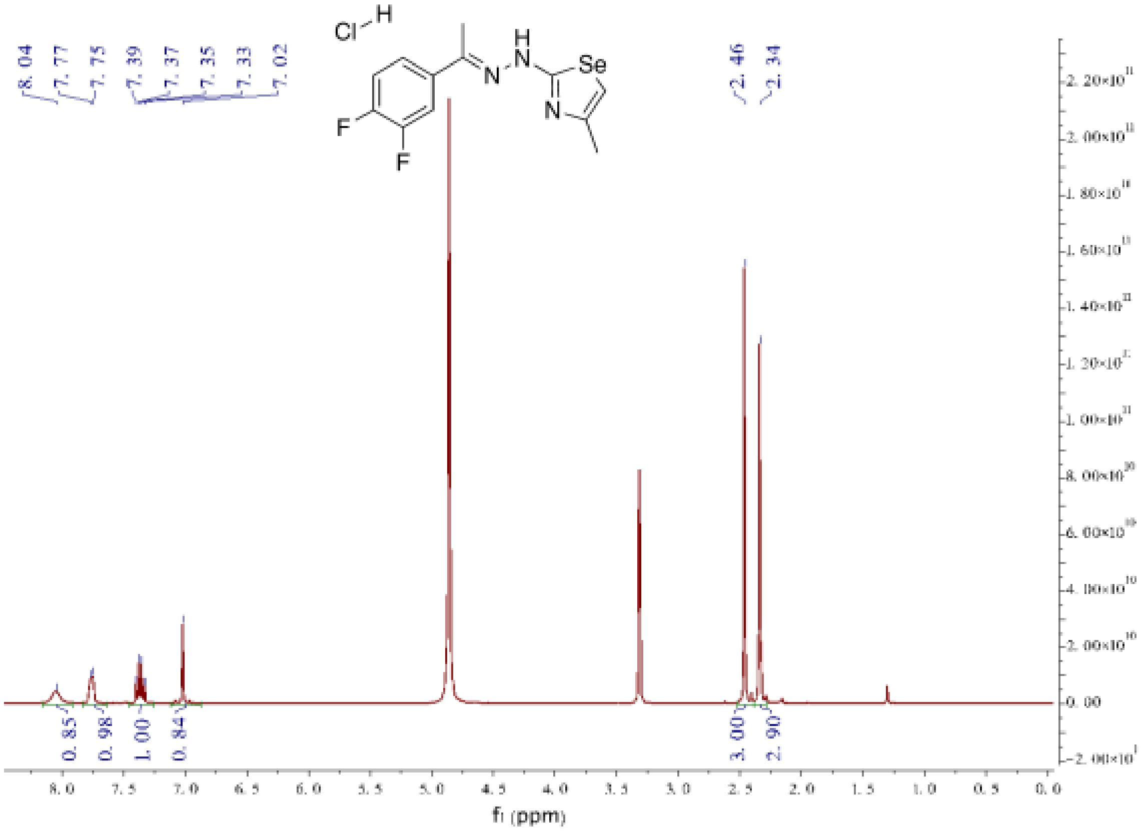

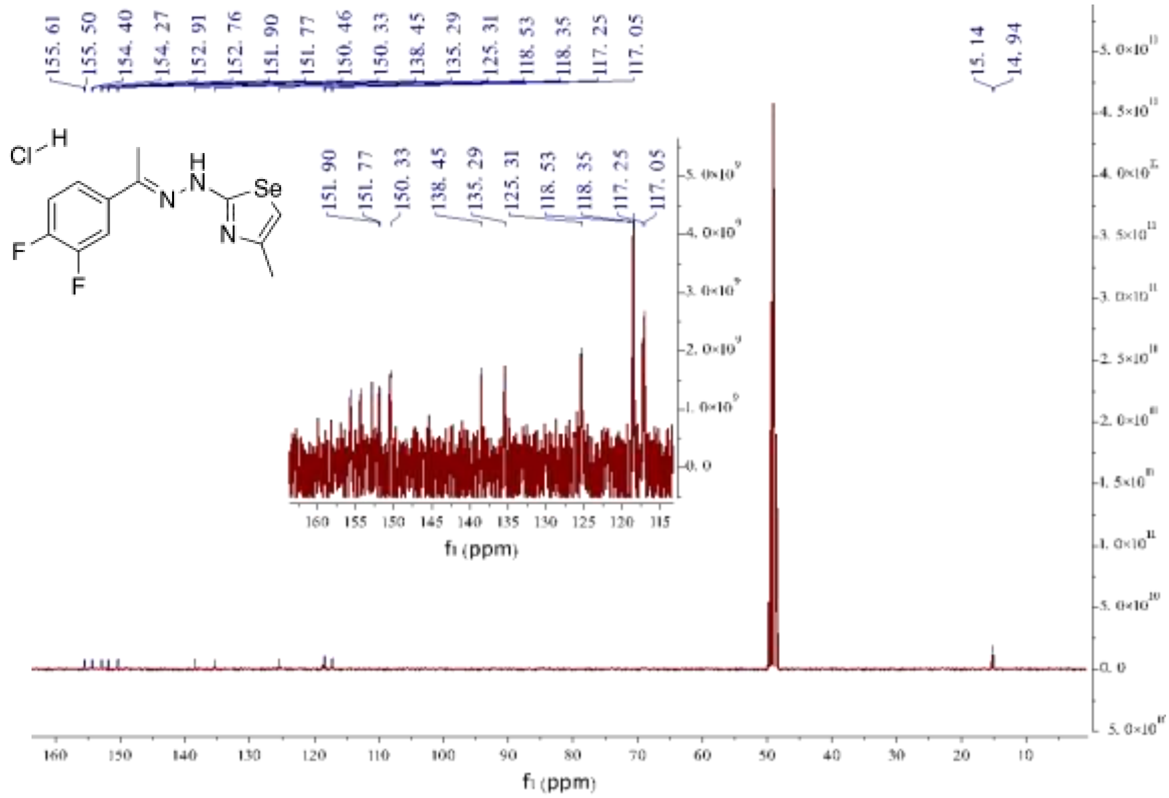

Figure S48. ^{13}C-NMR (up) and qNMR (down) of compound ***Se*C6**.

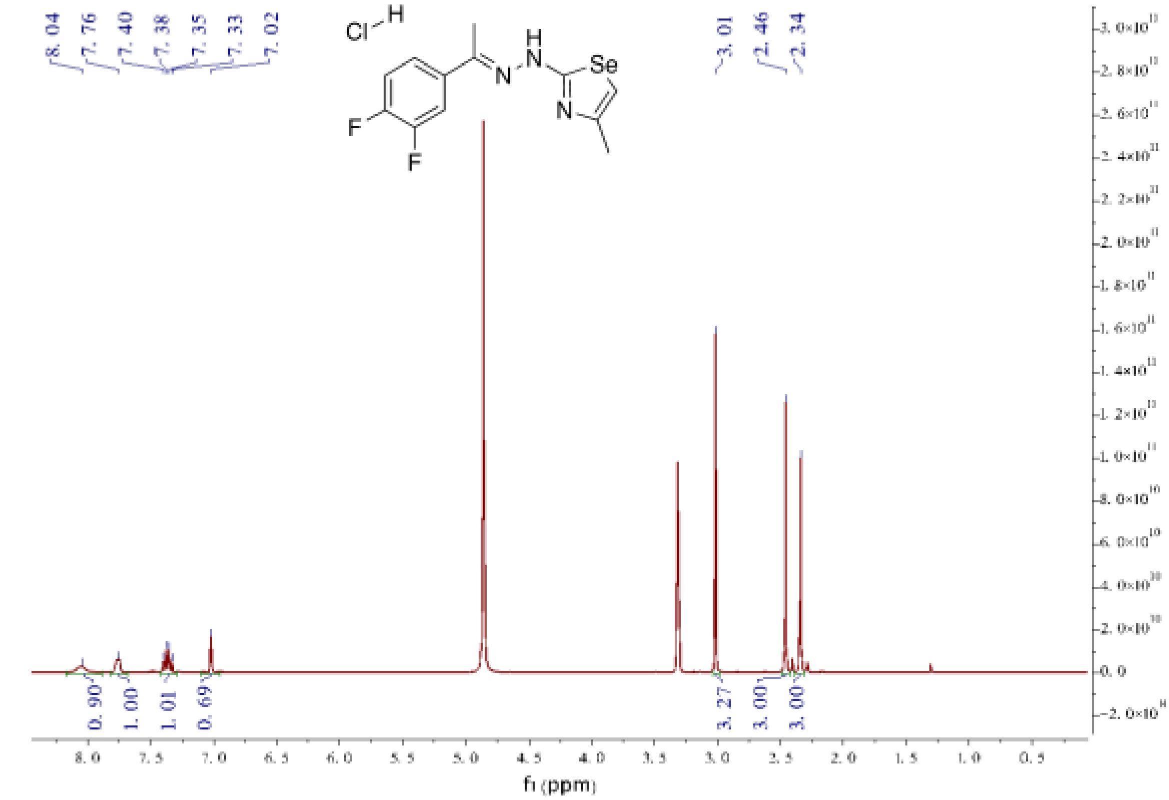

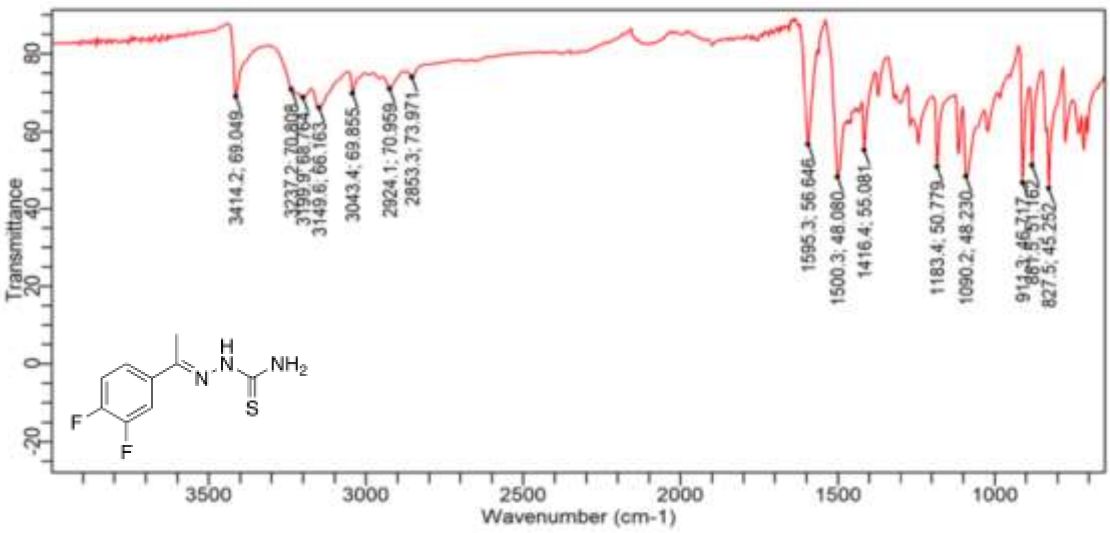

Figure S49. IR (up) and ^{1}H-NMR (down) of compound **SO6**.

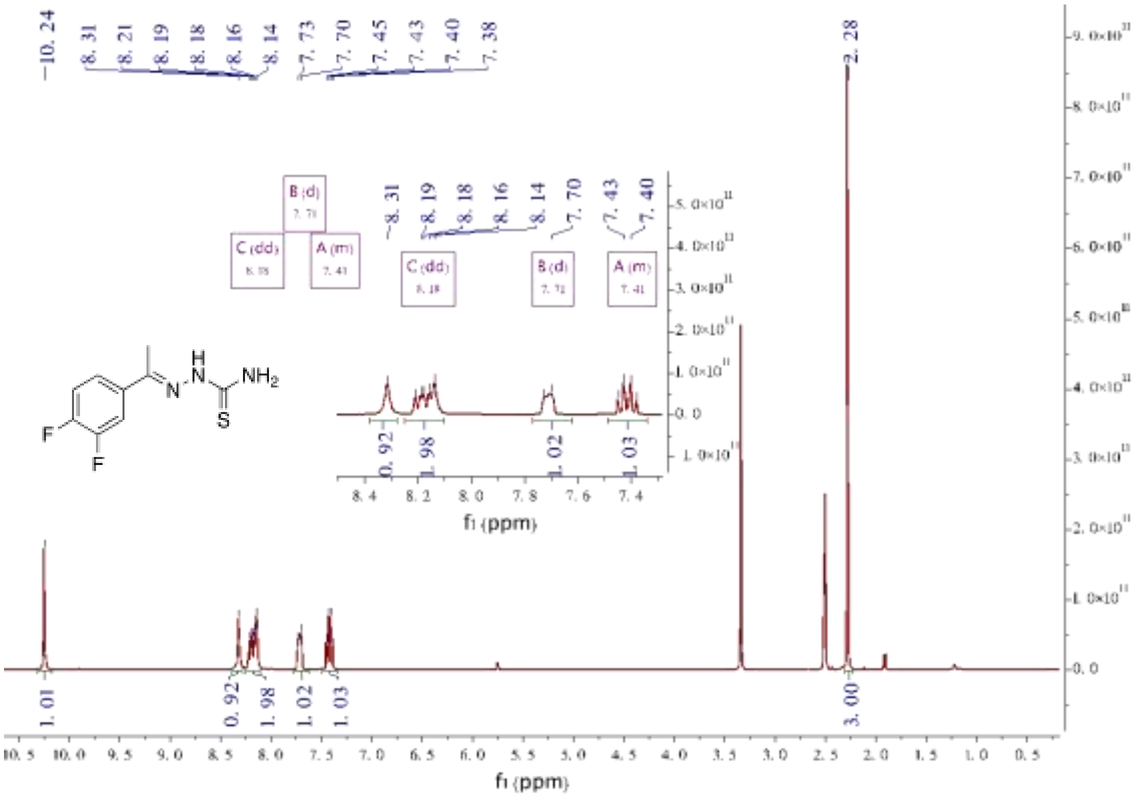

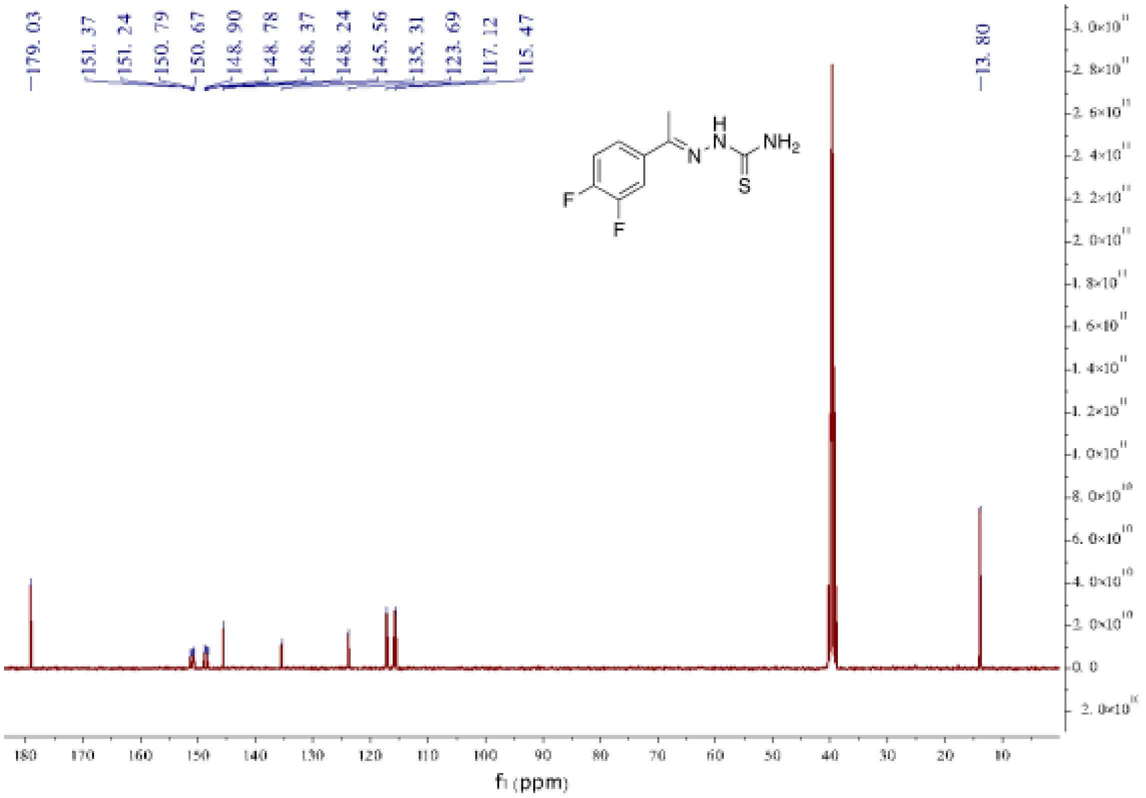

Figure S50. ^{13}C-NMR (up) and qNMR (down) of compound **SO6**.

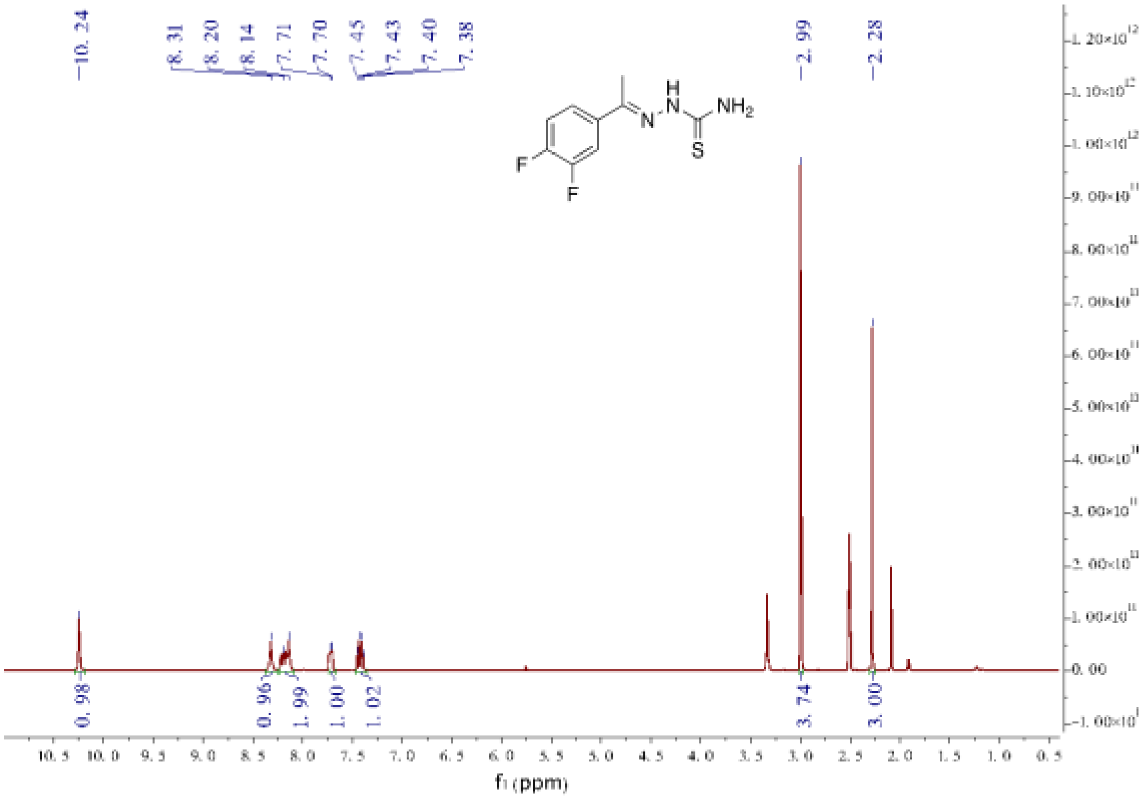

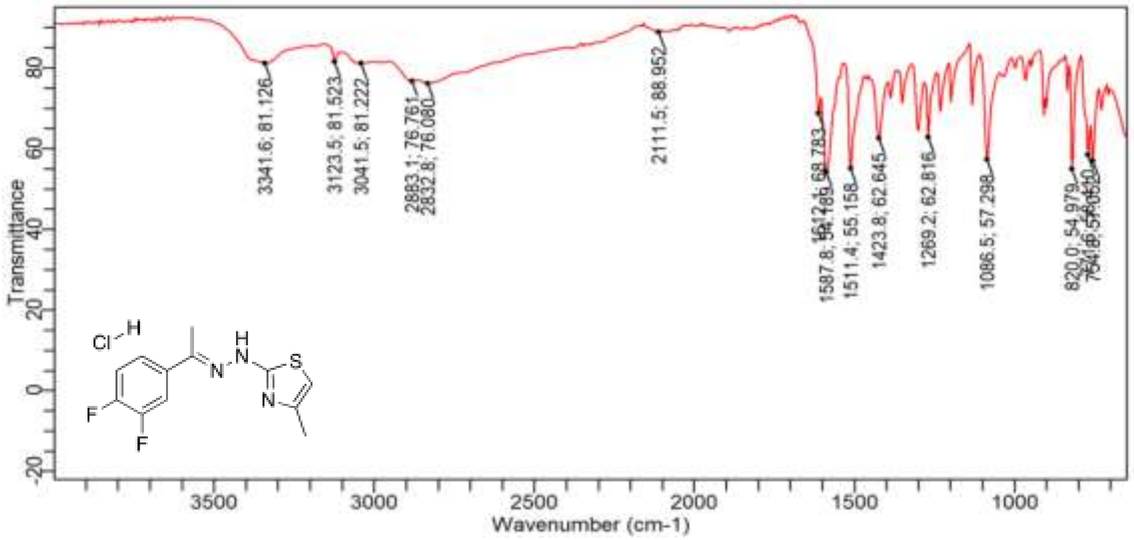

Figure S51. IR (up) and ^{1}H-NMR (down) of compound ***S*C6**.

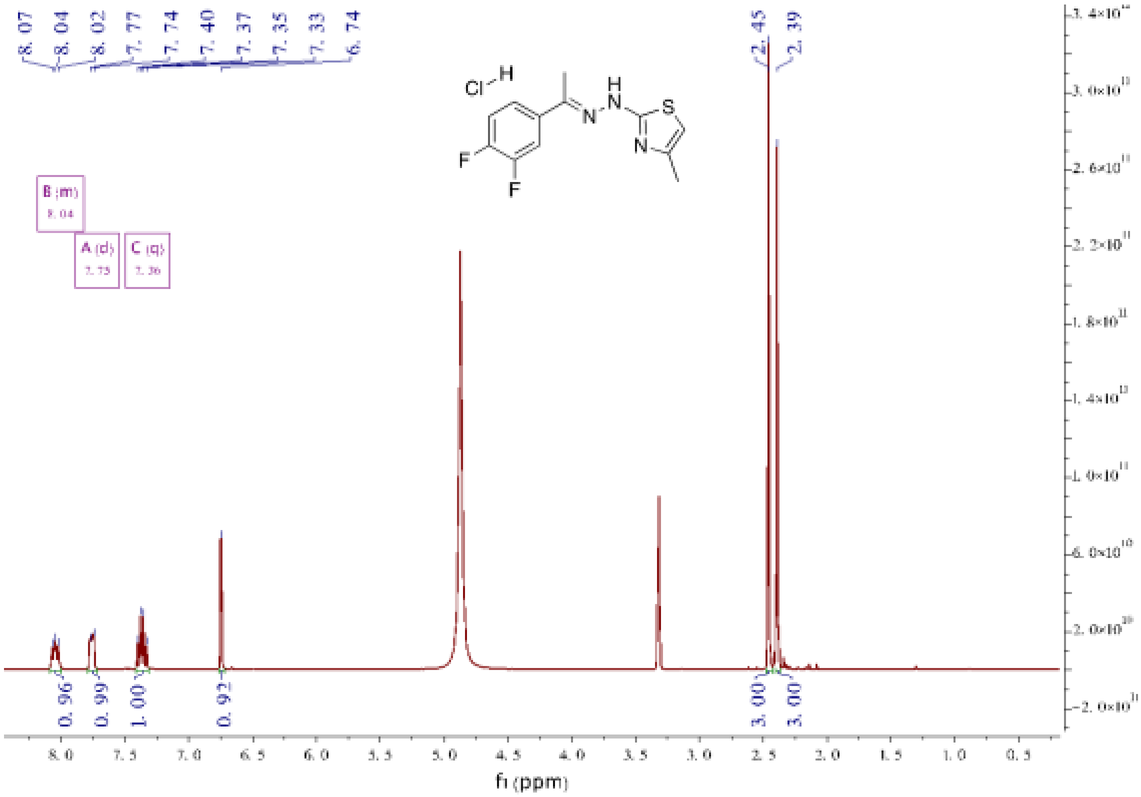

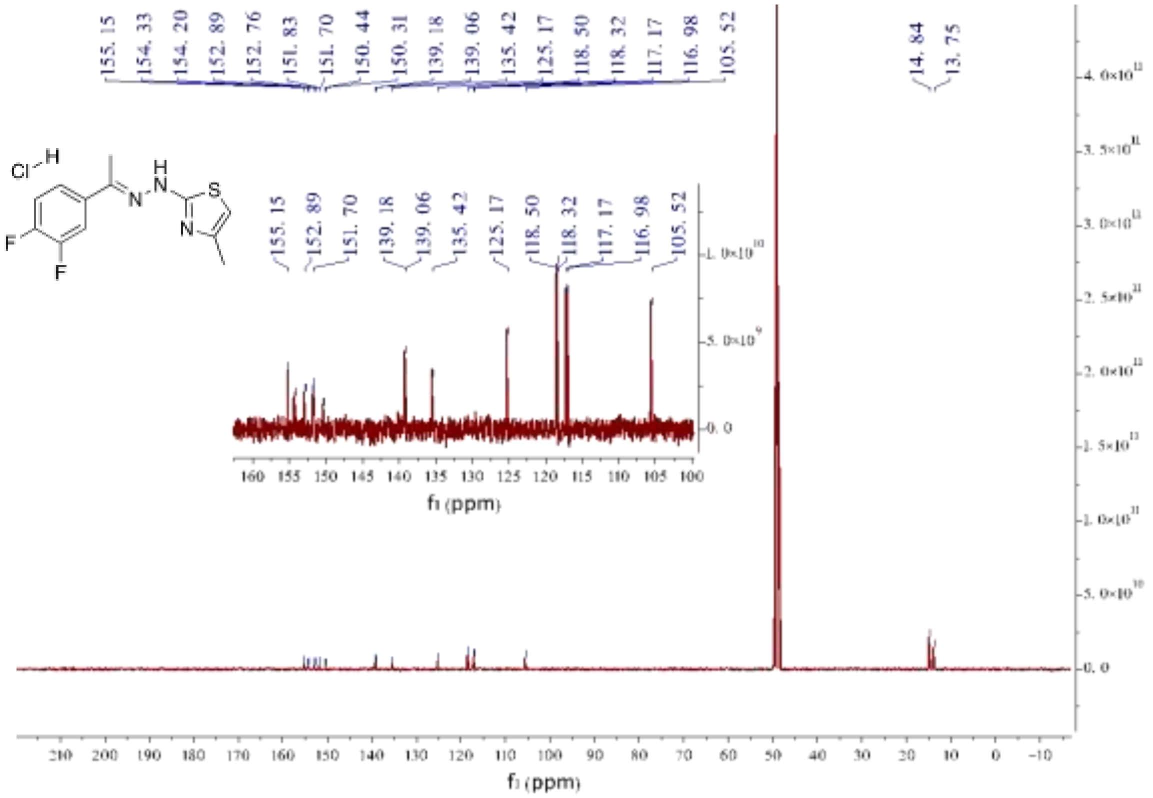

Figure S52. ^{13}C-NMR (up) and qNMR (down) of compound ***S*C6**.

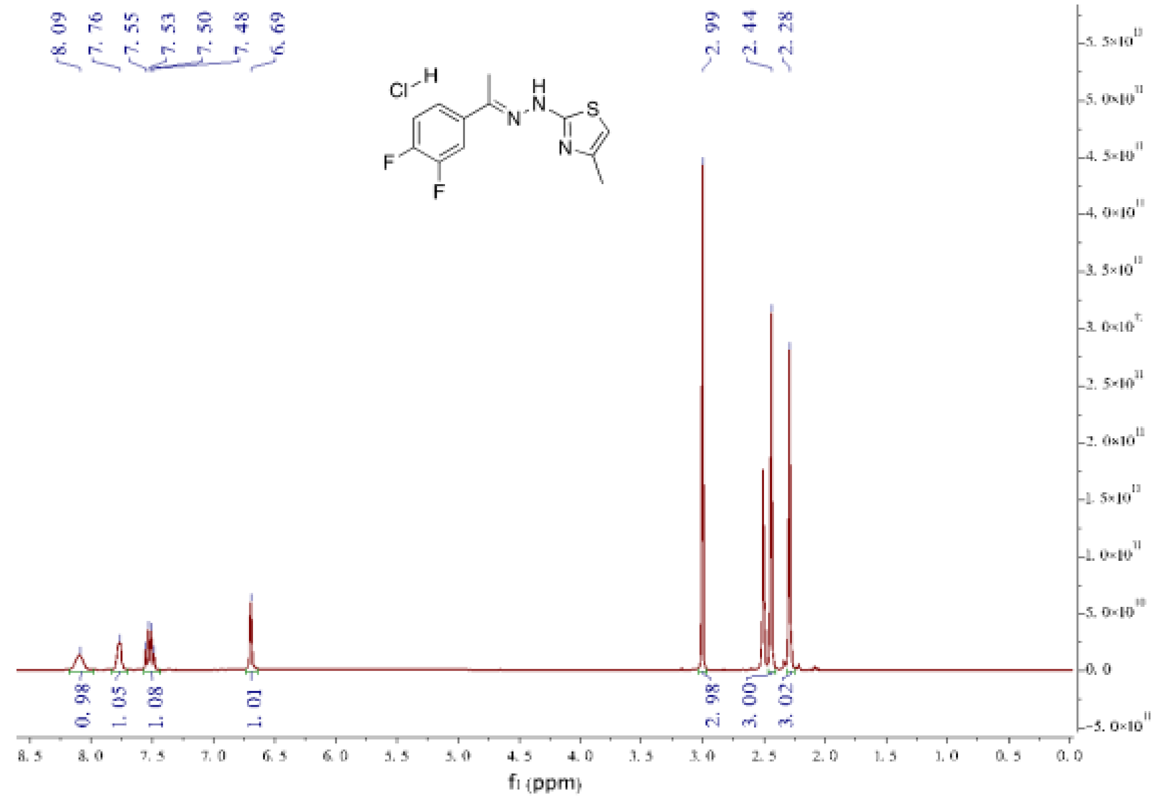

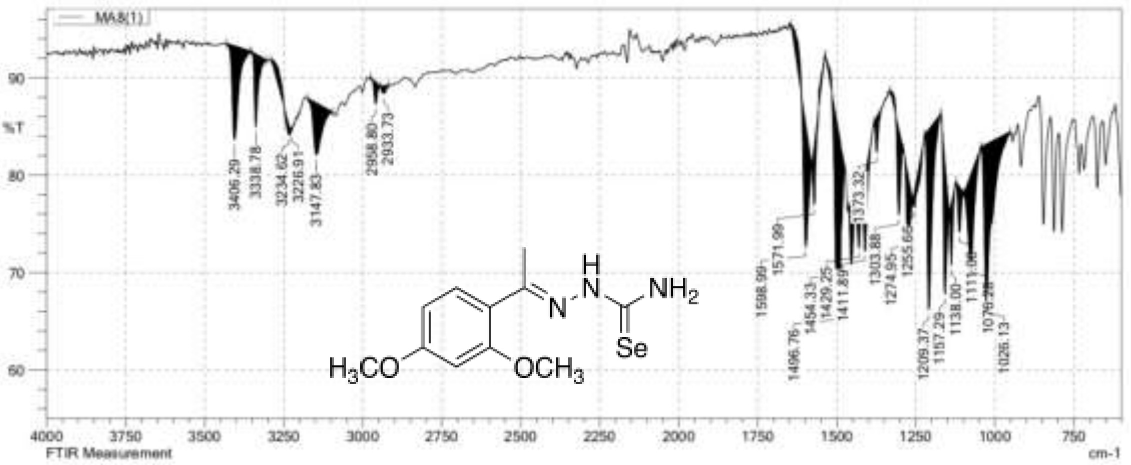

Figure S53. IR (up) and ^{1}H-NMR (down) of compound ***Se*O7**.

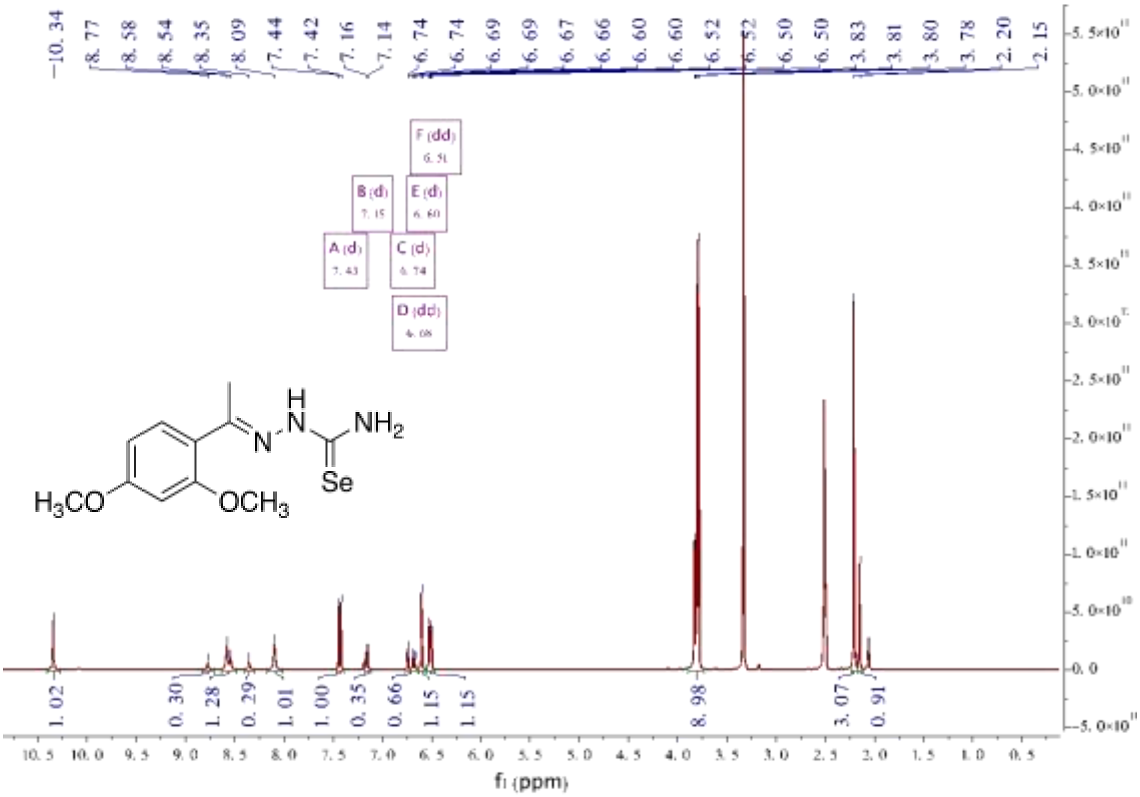

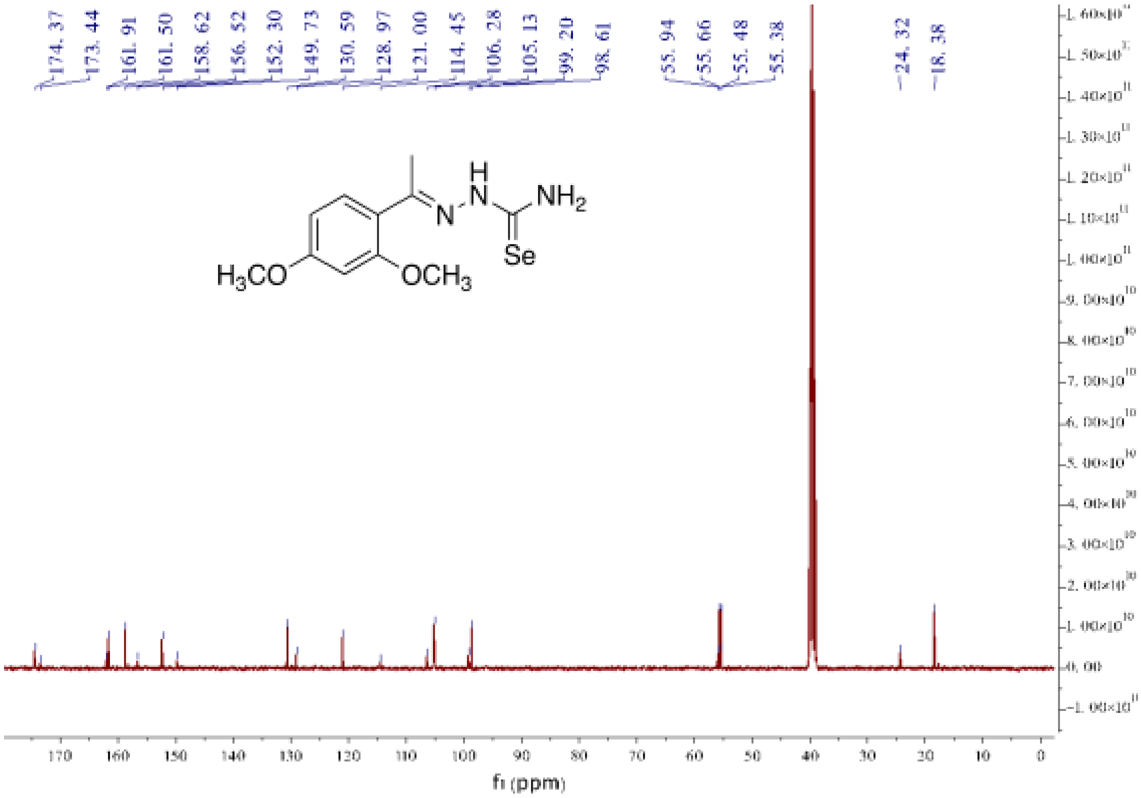

Figure S54. ^{13}C-NMR (up) and ^{77}Se-NMR (down) of compound ***Se*O7**.

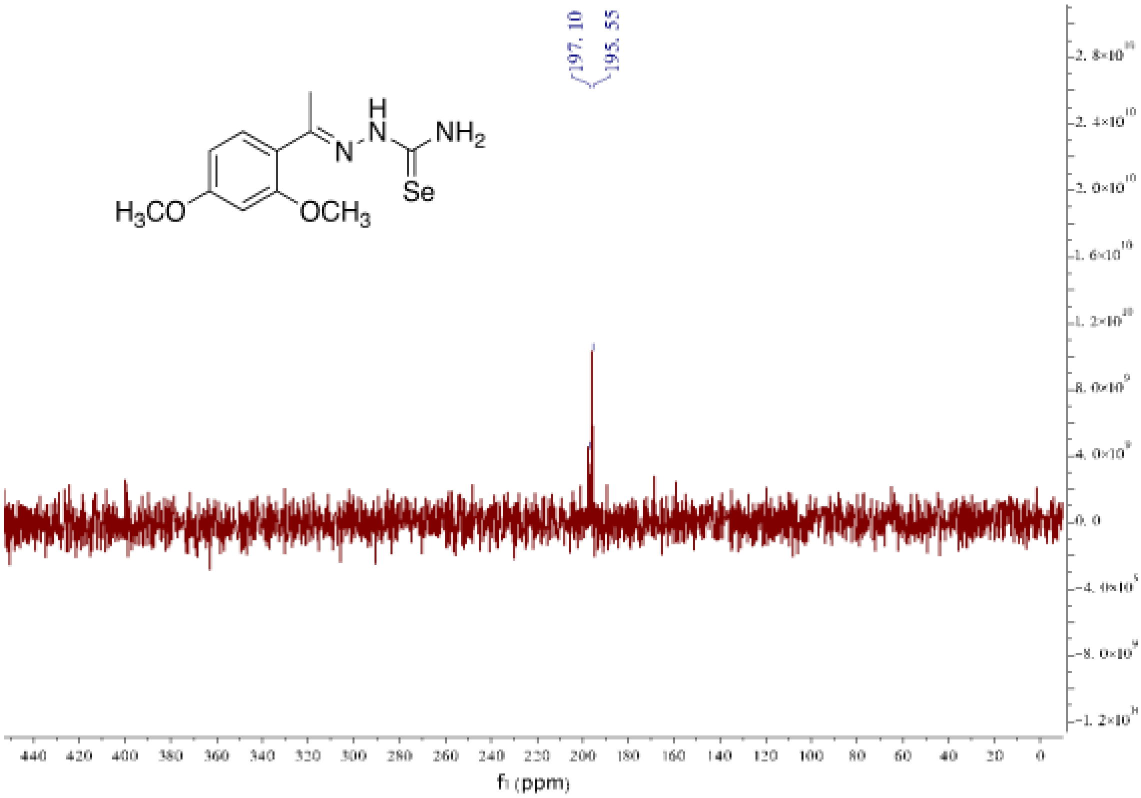

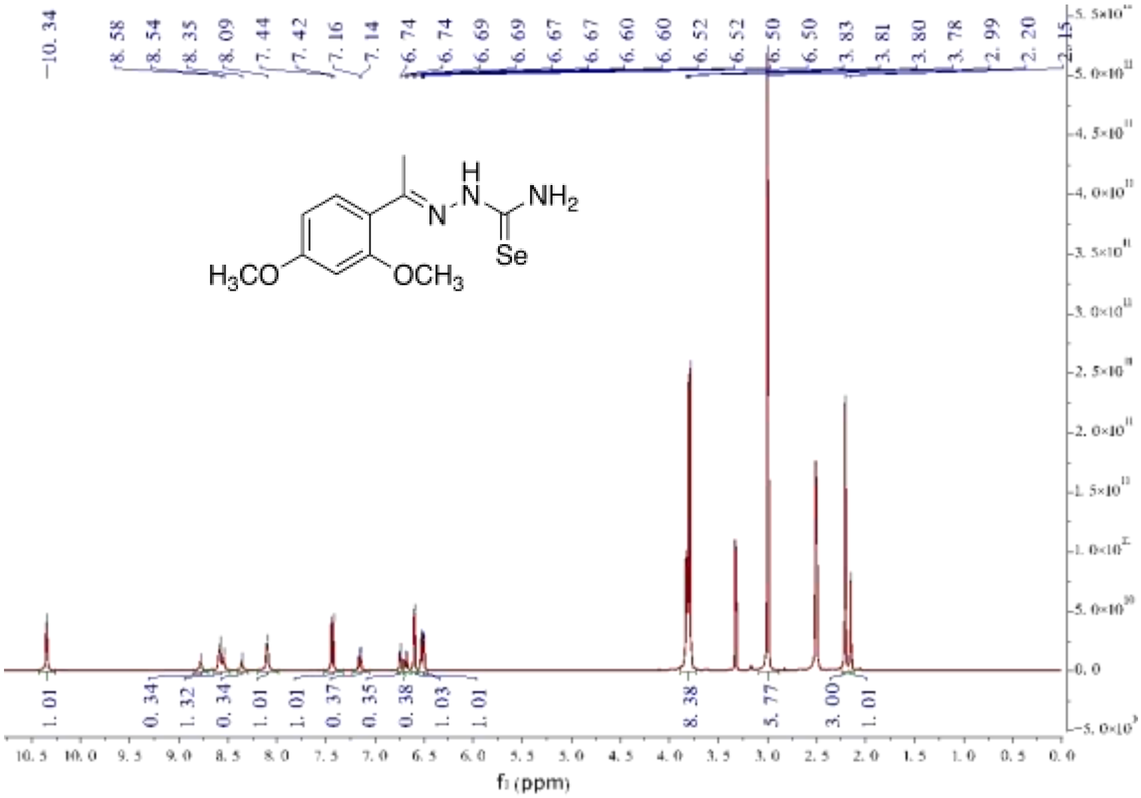

Figure S55. qNMR (up) of compound ***Se*O7** and IR (down) of compound ***Se*C7**.

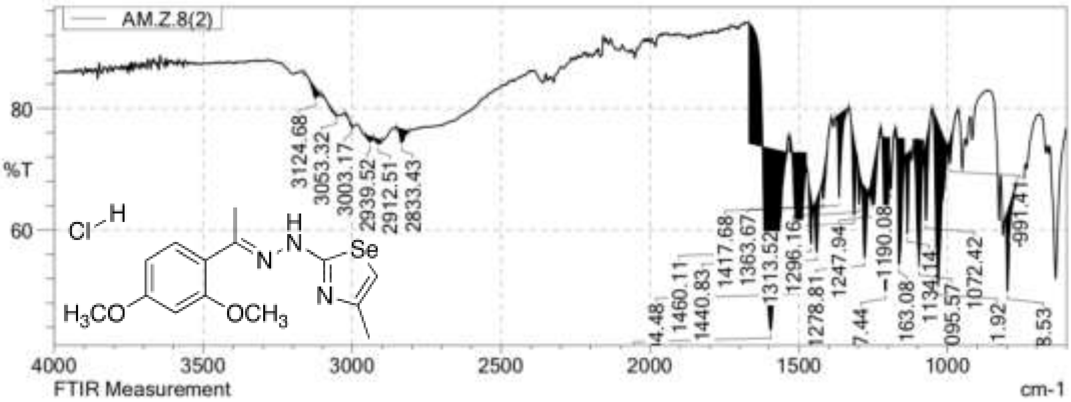

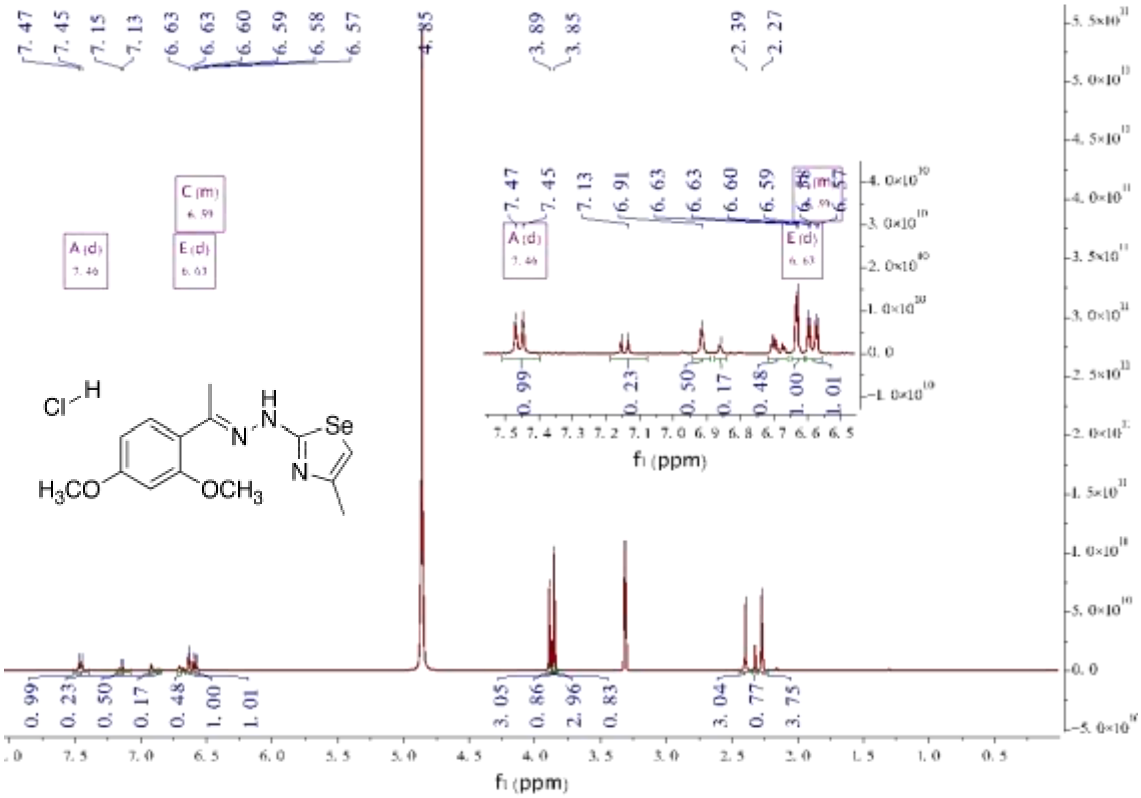

Figure S56. ^{1}H-NMR (up) and ^{13}C-NMR (down) of compound ***Se*C7**.

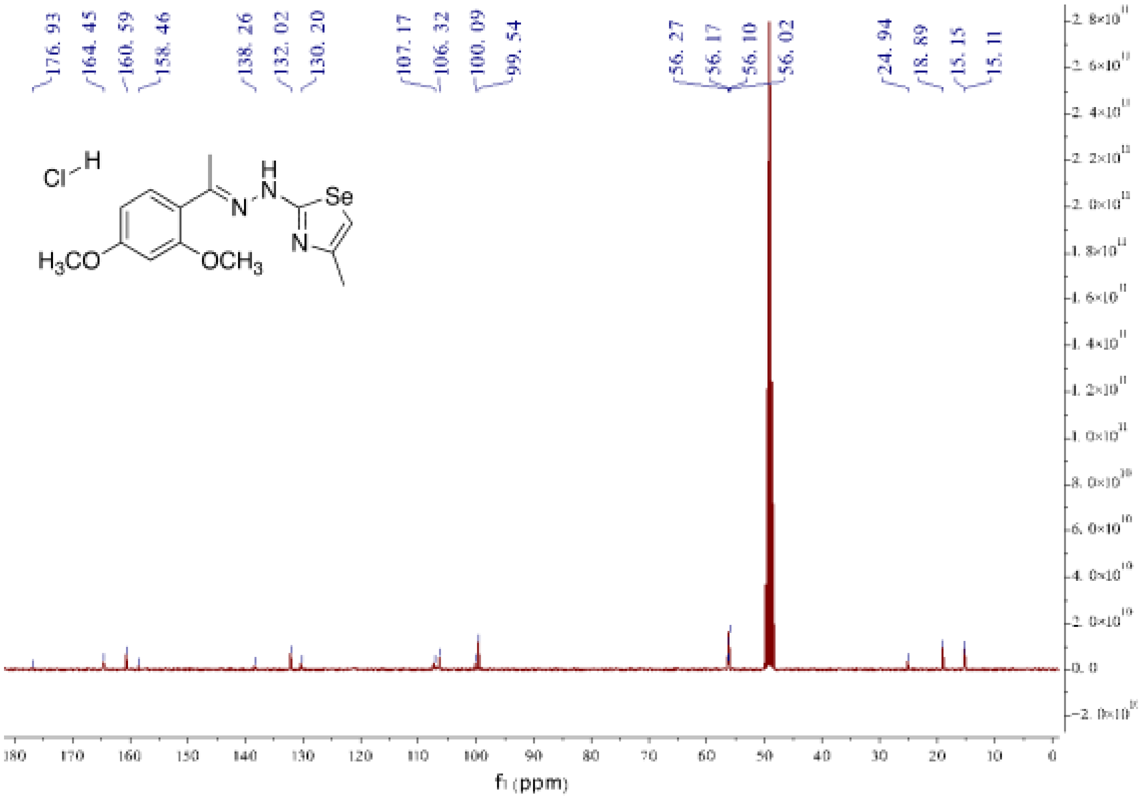

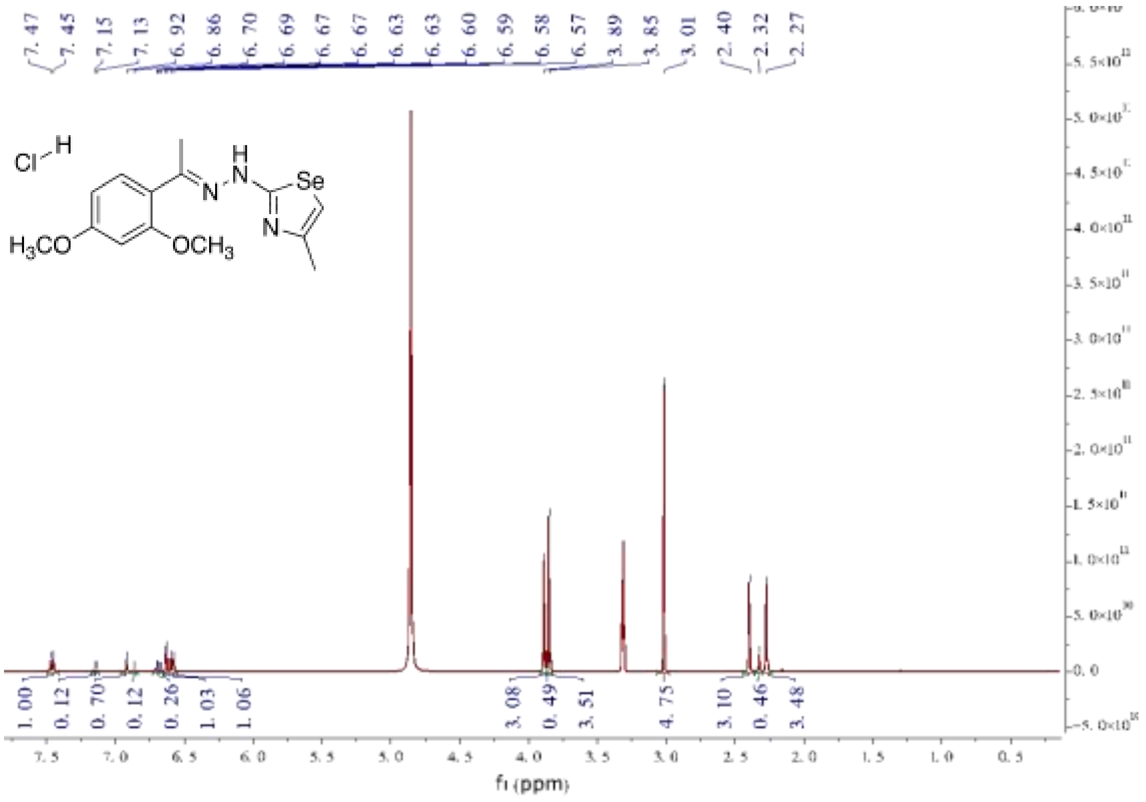

Figure S57. qNMR (down) of compound ***Se*C7** and IR (down) of compound **SO7.**

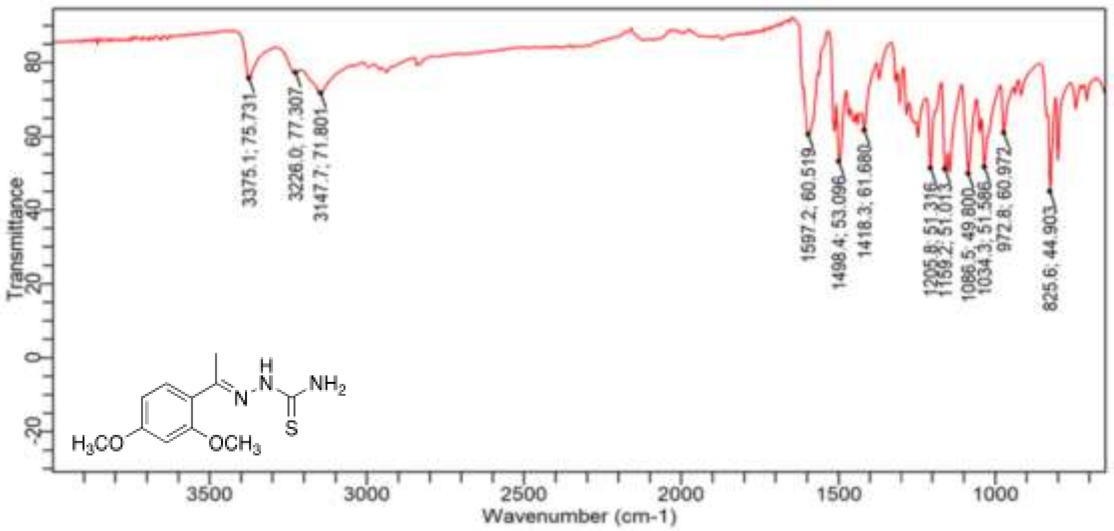

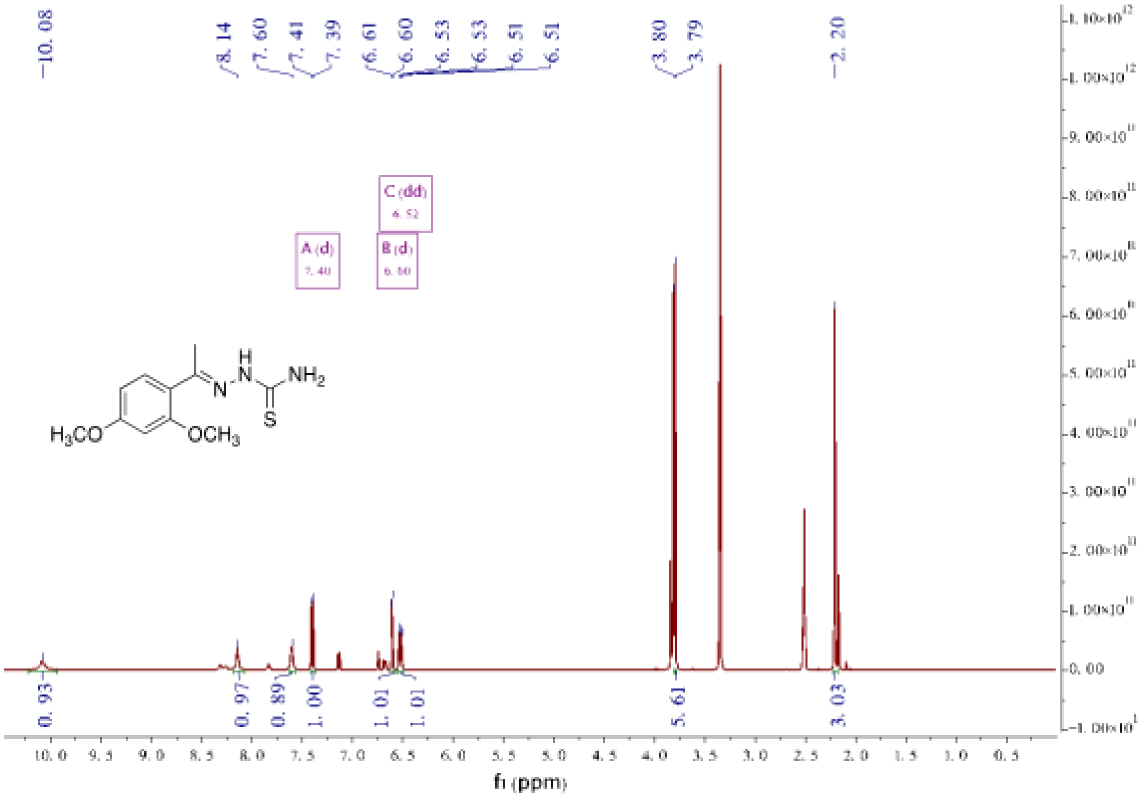

Figure S58. ^{1}H-NMR (up) and ^{13}C-NMR (down) of compound **SO7**.

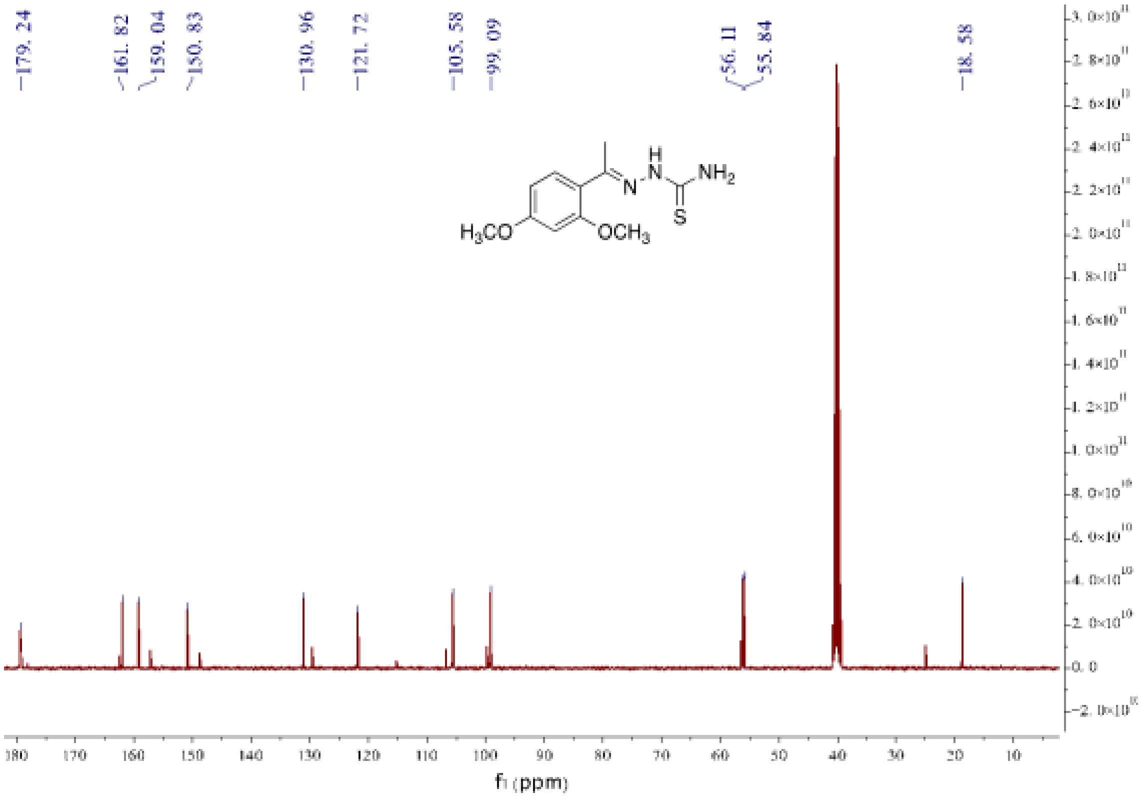

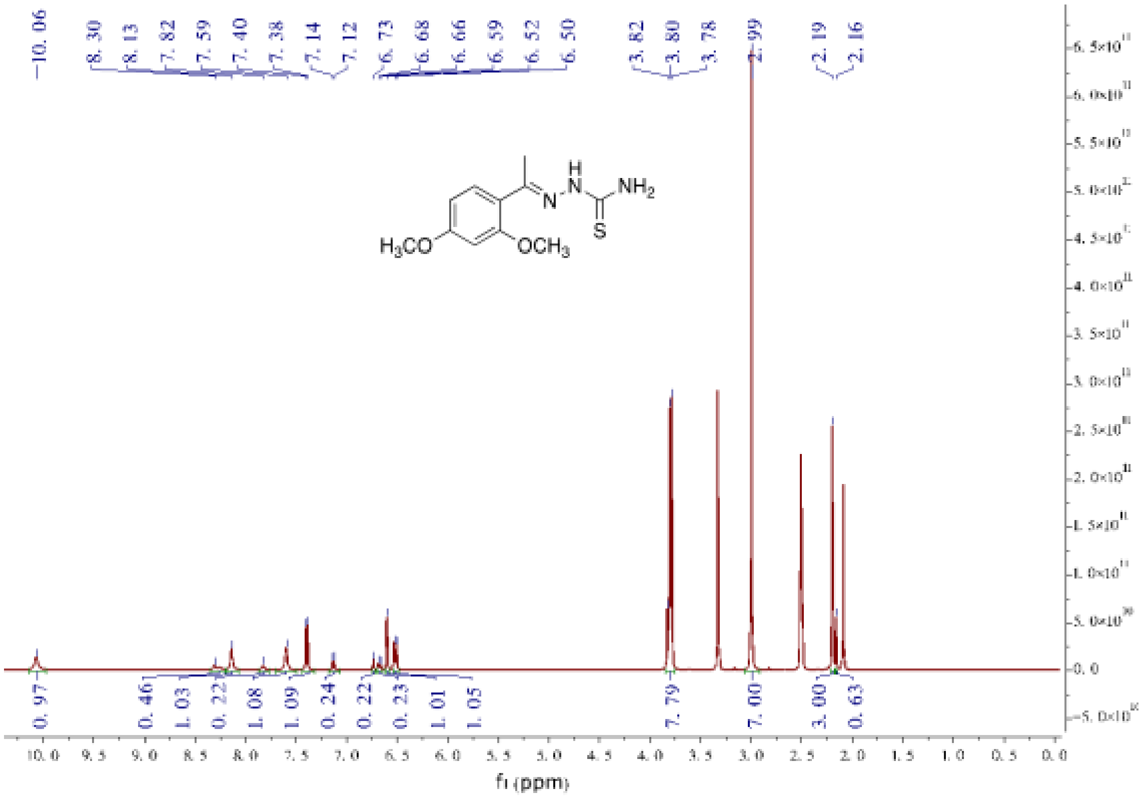

Figure S59. qNMR (up) of compound **SO7** and IR (up) of compound ***S*C7**.

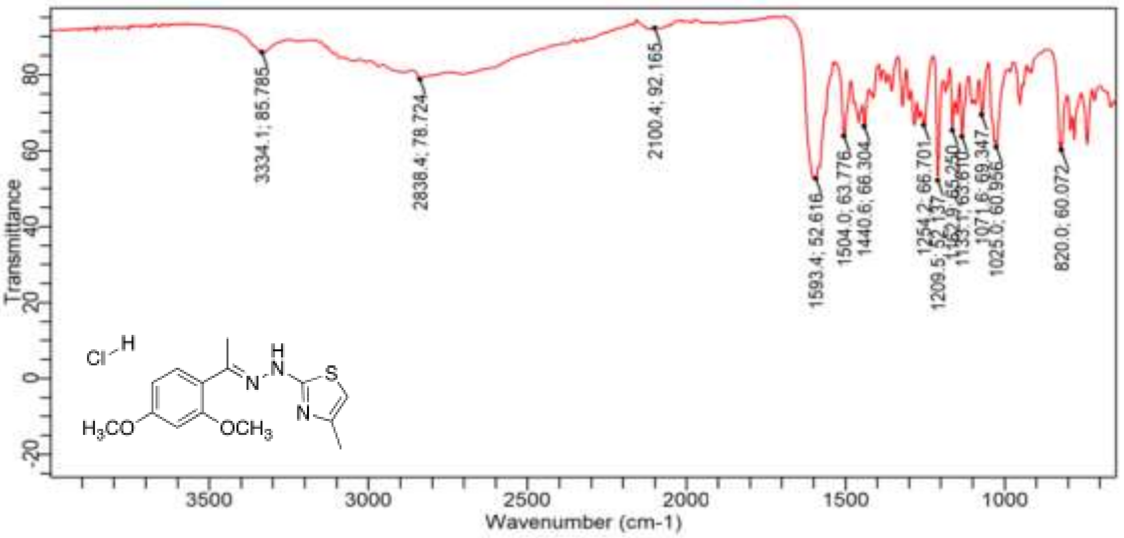

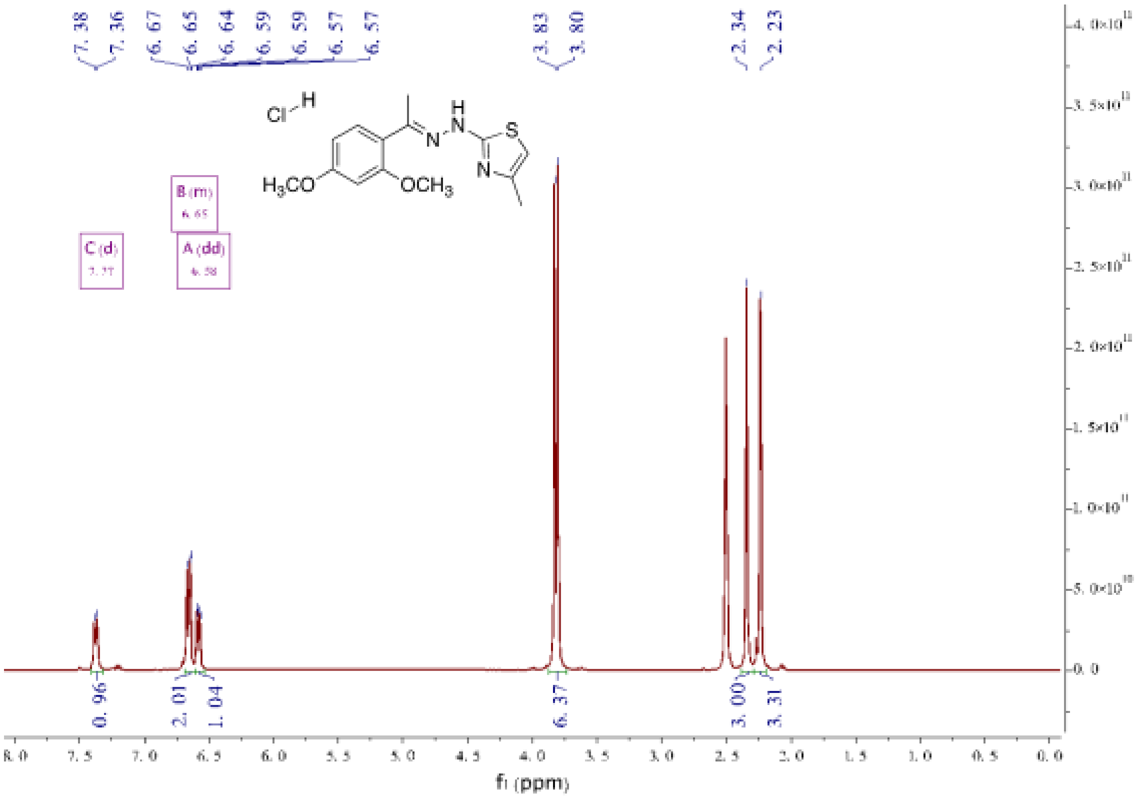

Figure S60. ^{1}H-NMR (up) and ^{13}C-NMR (down) of compound ***S*C7**.

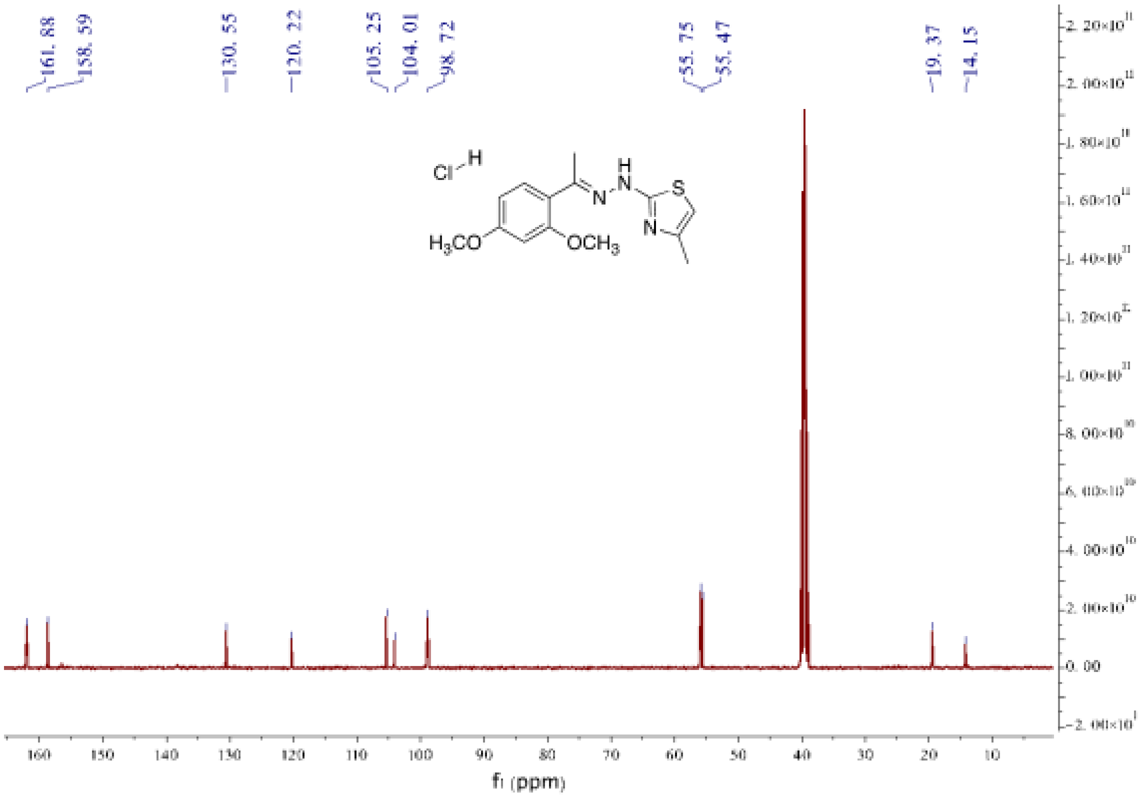

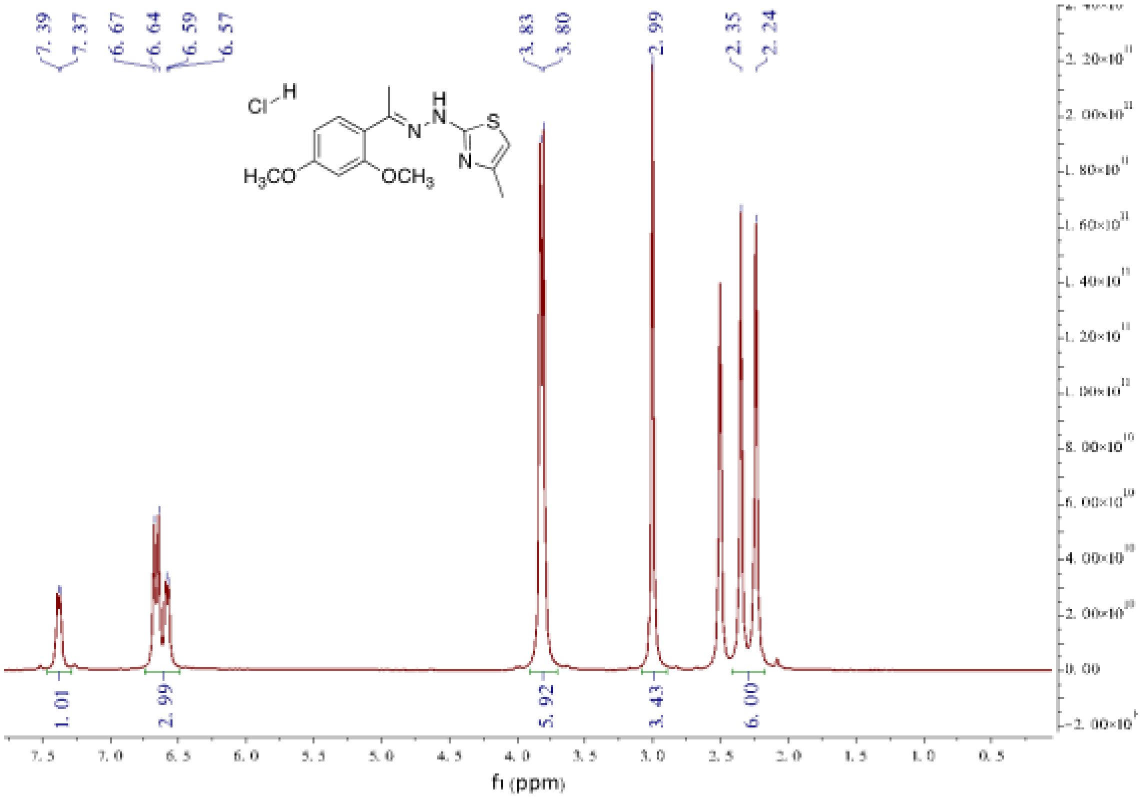

Figure S61. qNMR (up) of compound ***S*C7** and IR (down) of compound ***Se*O8**.

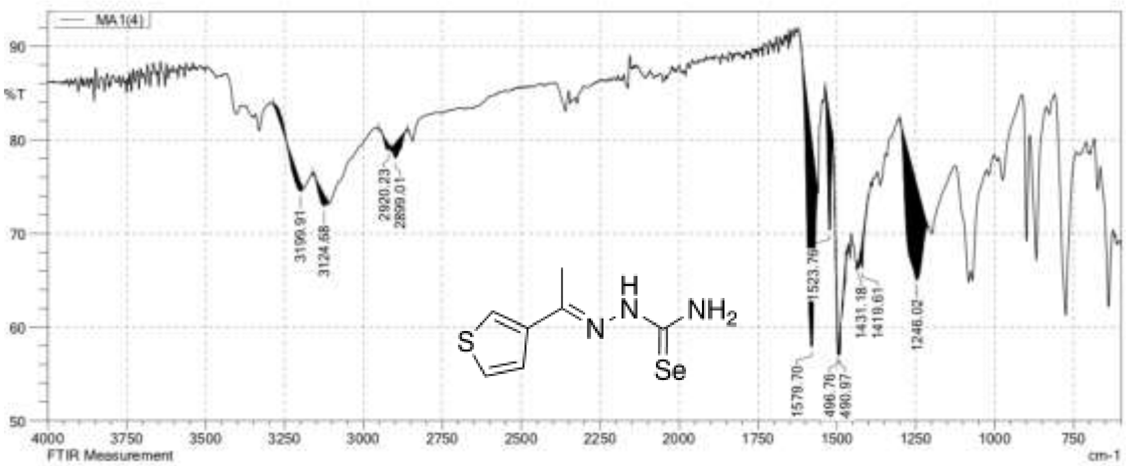

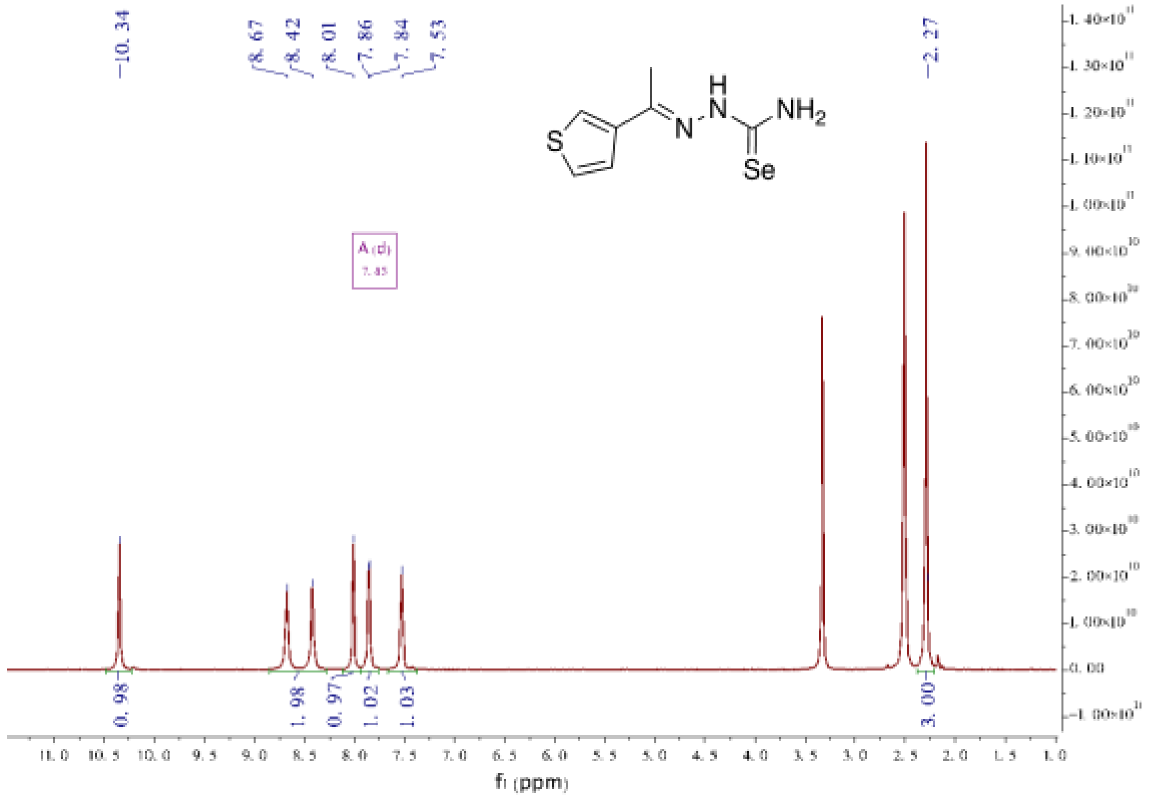

Figure S62. ^{1}H-NMR (up) and ^{13}C-NMR (down) of compound ***Se*****O8.**

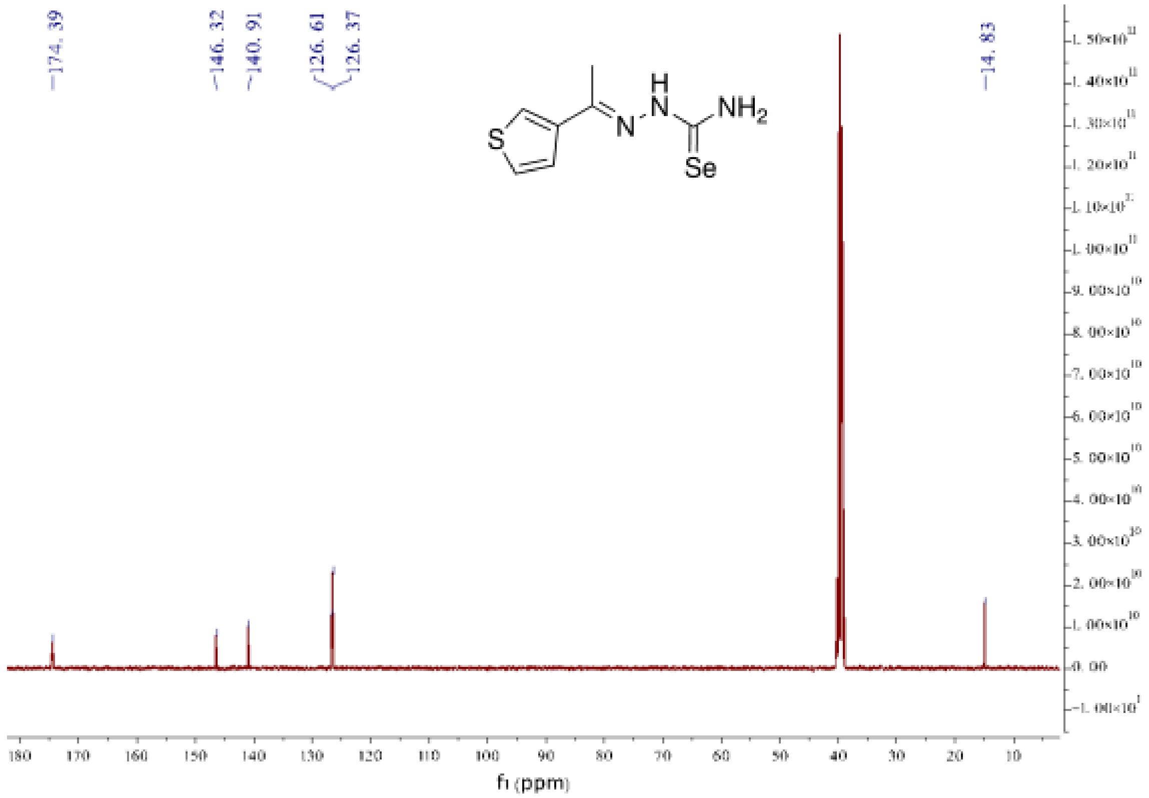

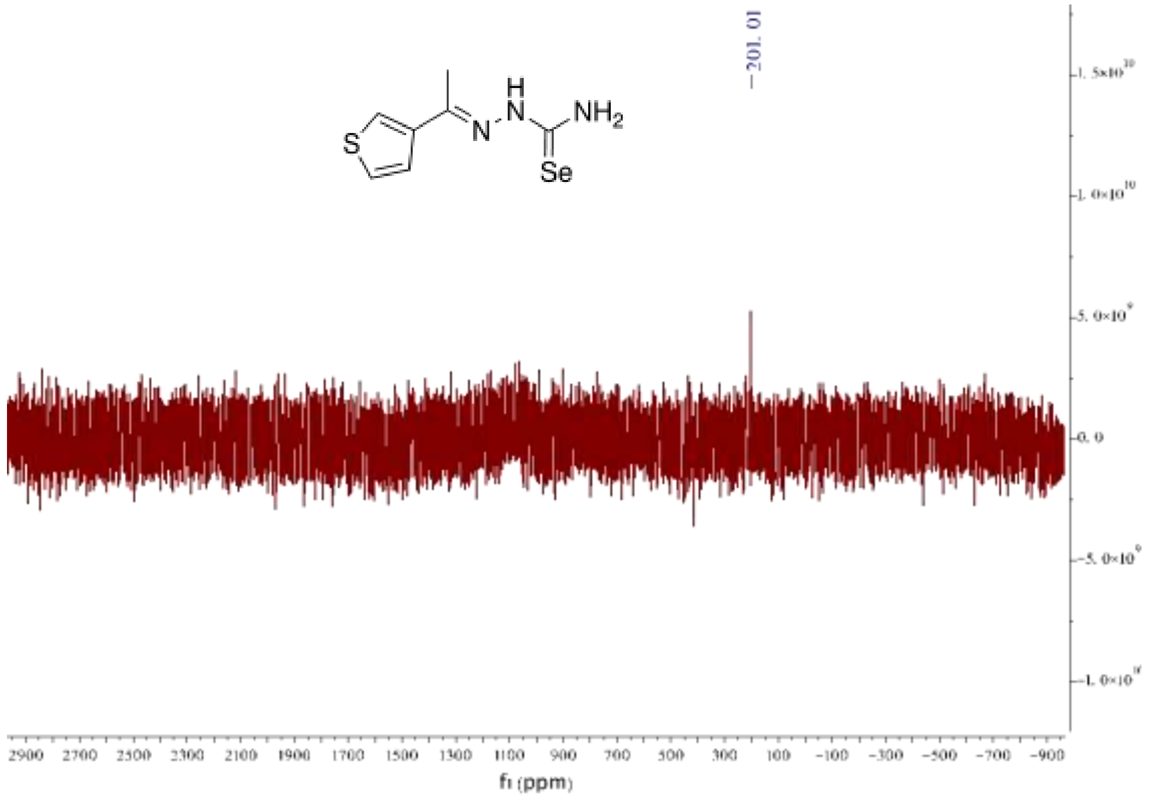

Figure S63. ^{77}Se-NMR (up) and qNMR (down) of compound ***Se*O8**.

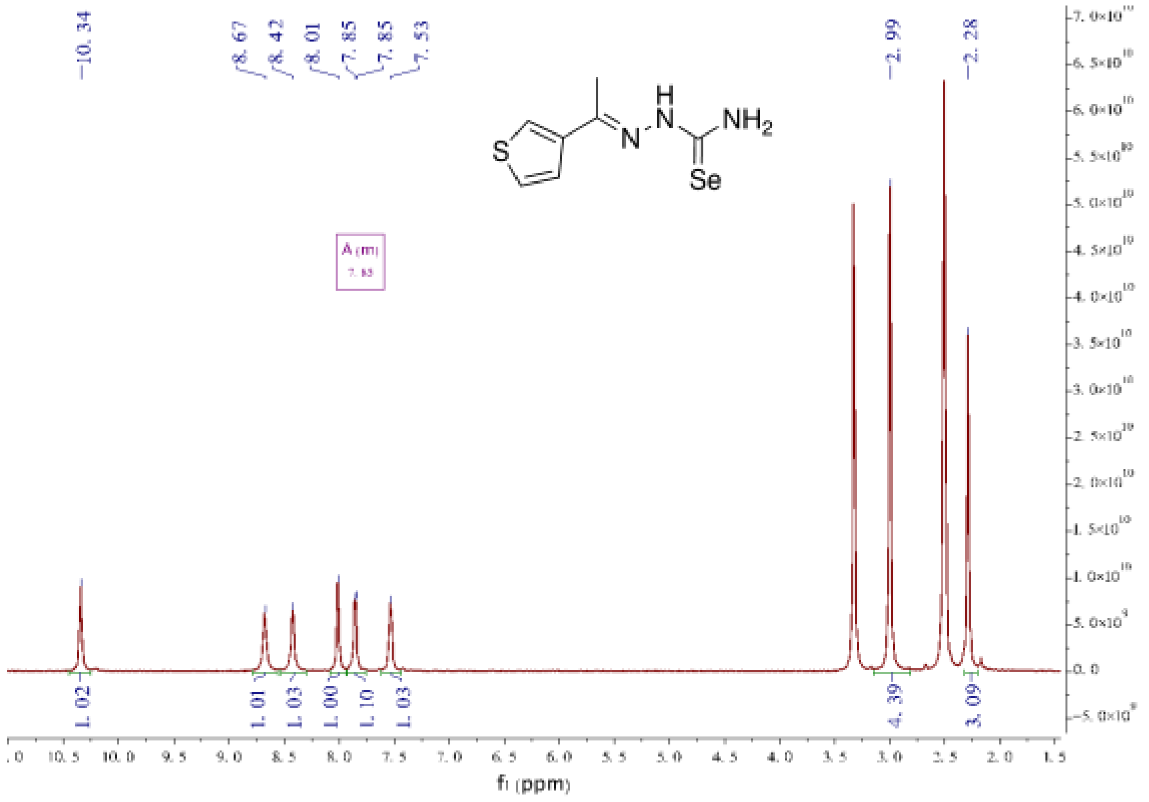

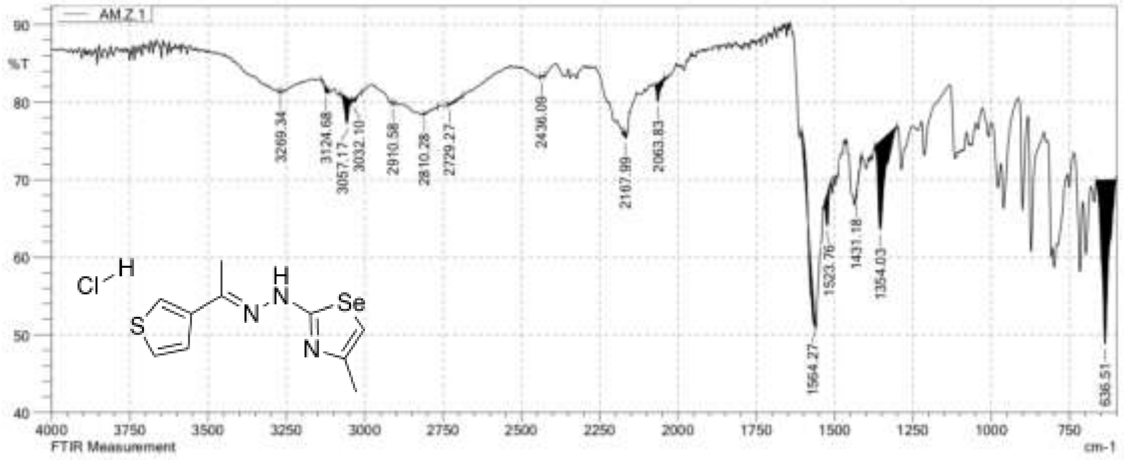

Figure S64. IR (up) and ^{1}H-NMR (down) of compound ***Se*C8**.

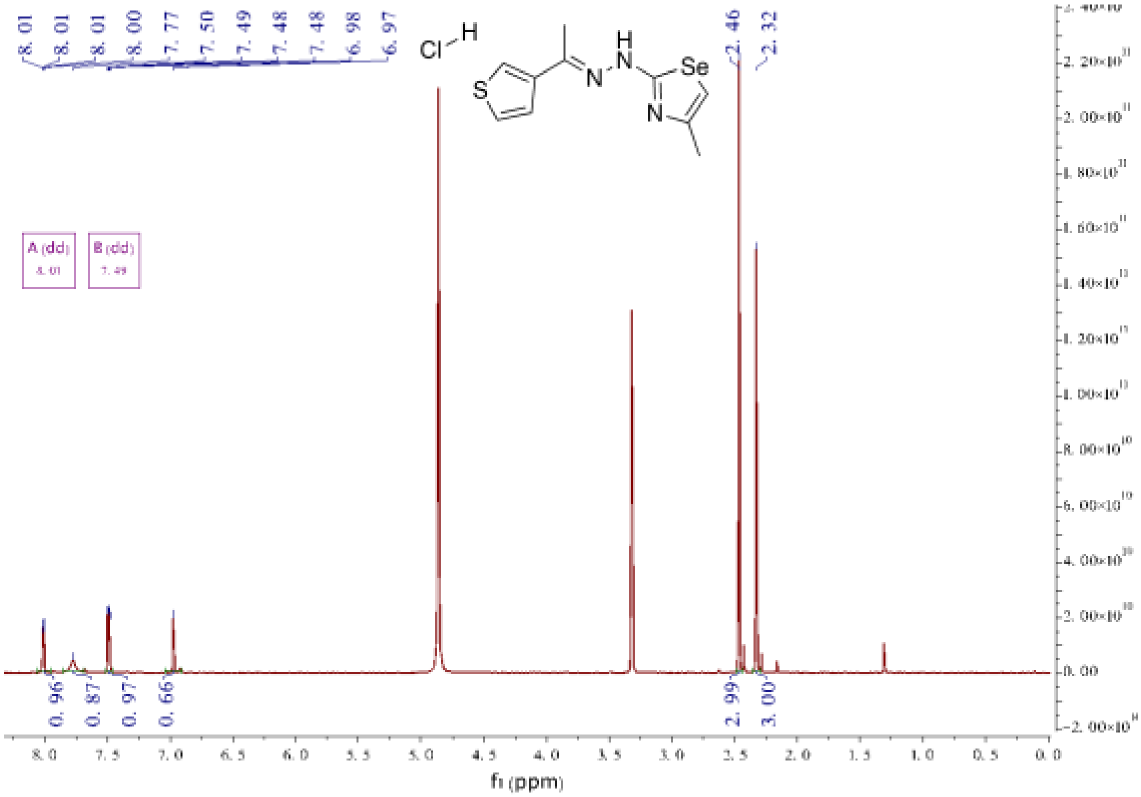

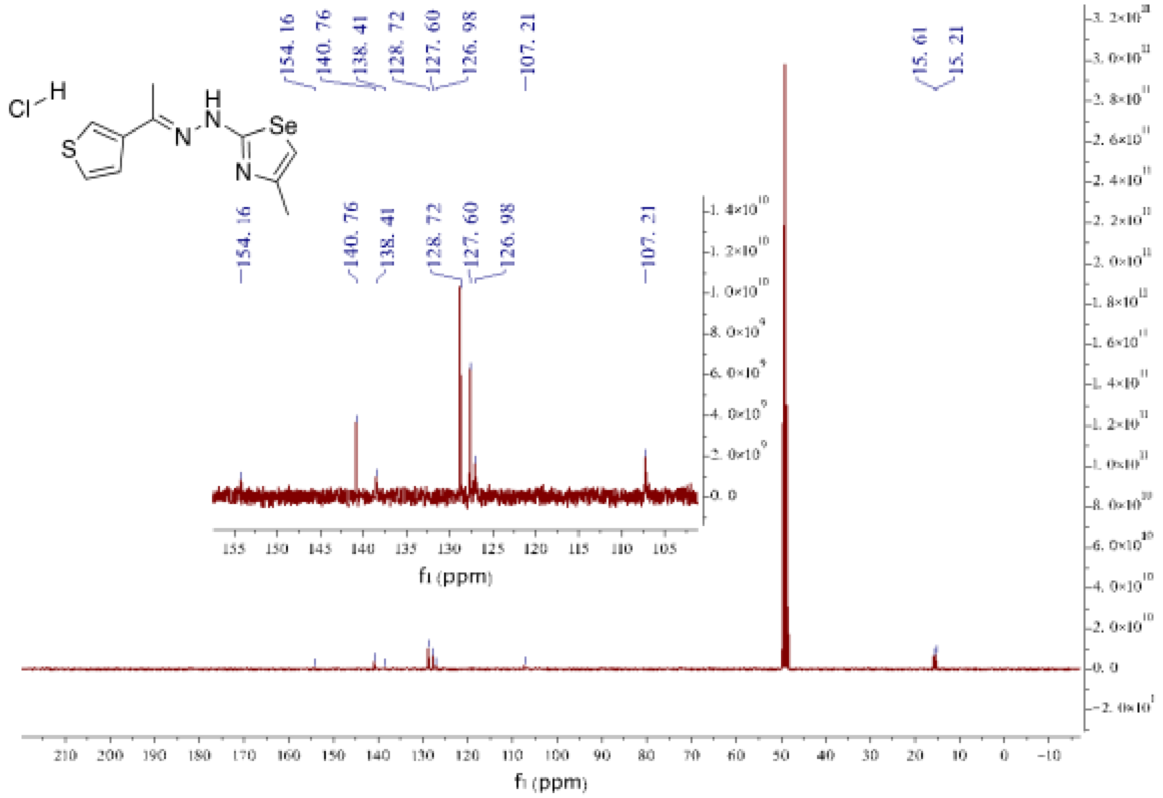

Figure S65. ^{13}C-NMR (up) and qNMR (down) of compound ***Se*C8**.

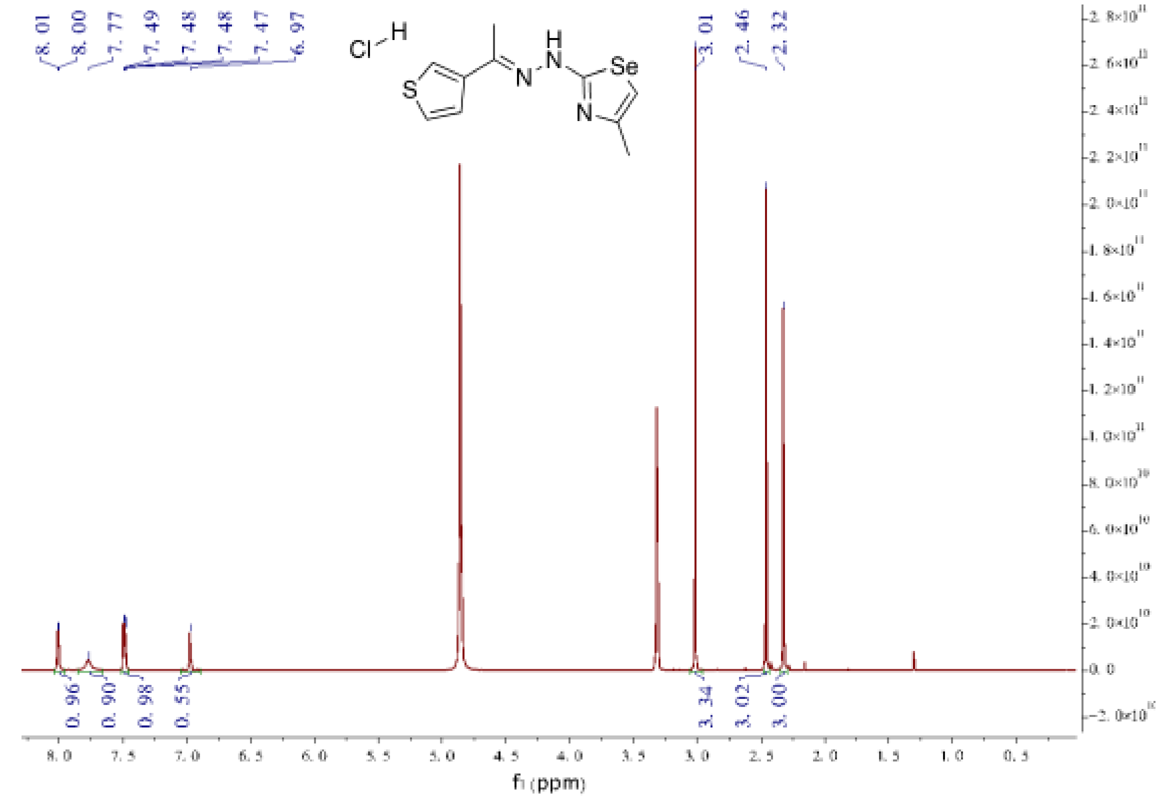

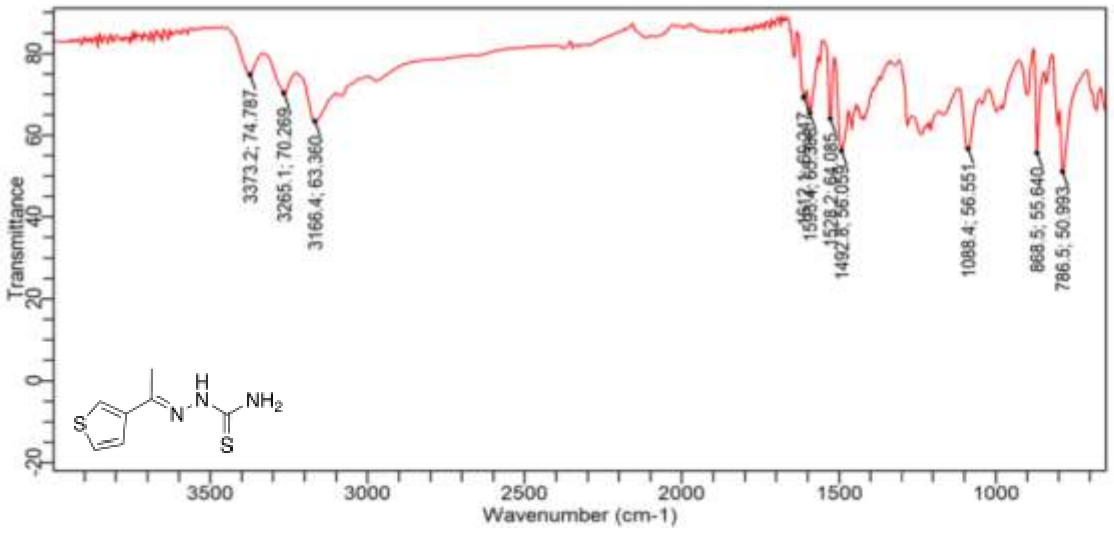

Figure S66. IR (up) and ^{1}H-NMR (down) of compound **SO8**.

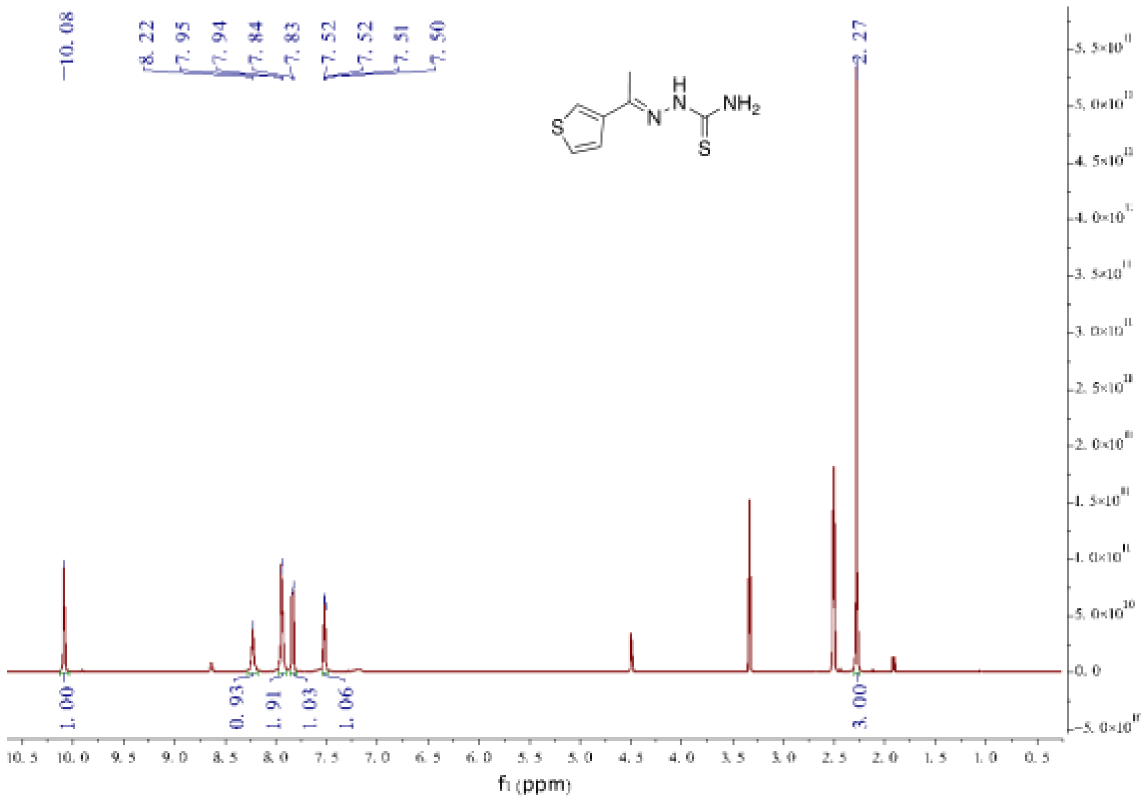

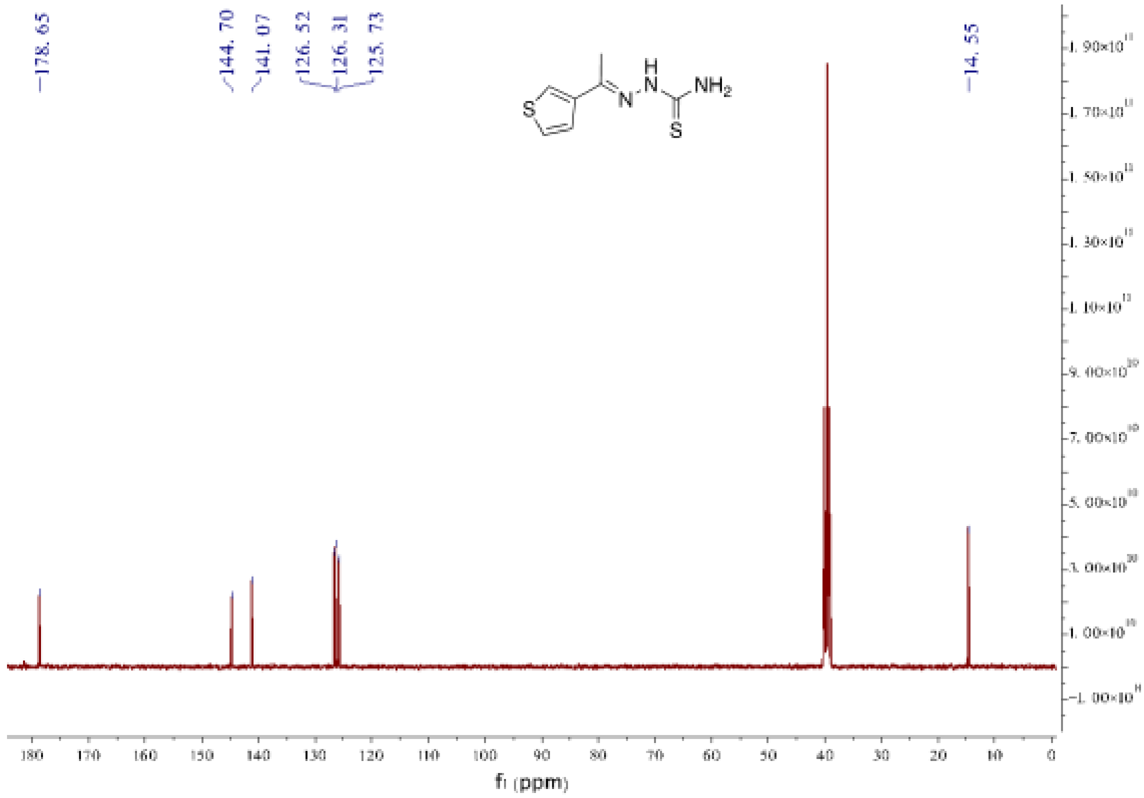

Figure S67. ^{13}C-NMR (up) and qNMR (down) of compound **SO8**.

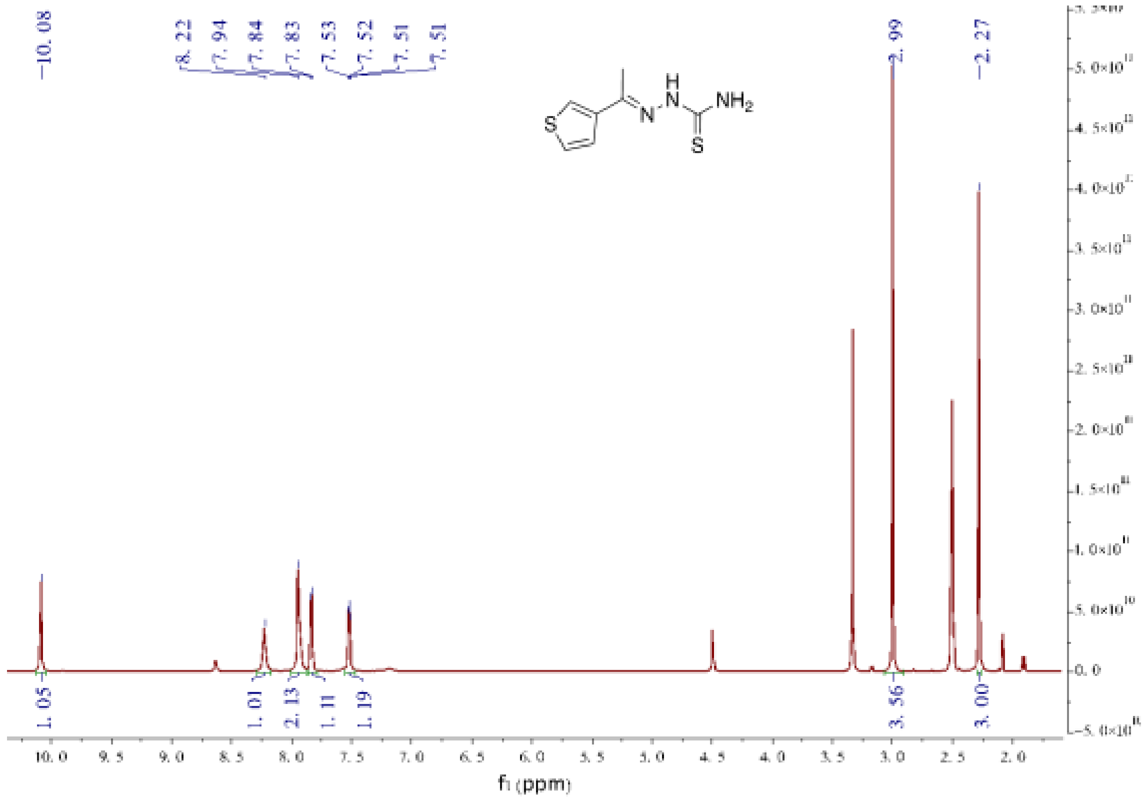

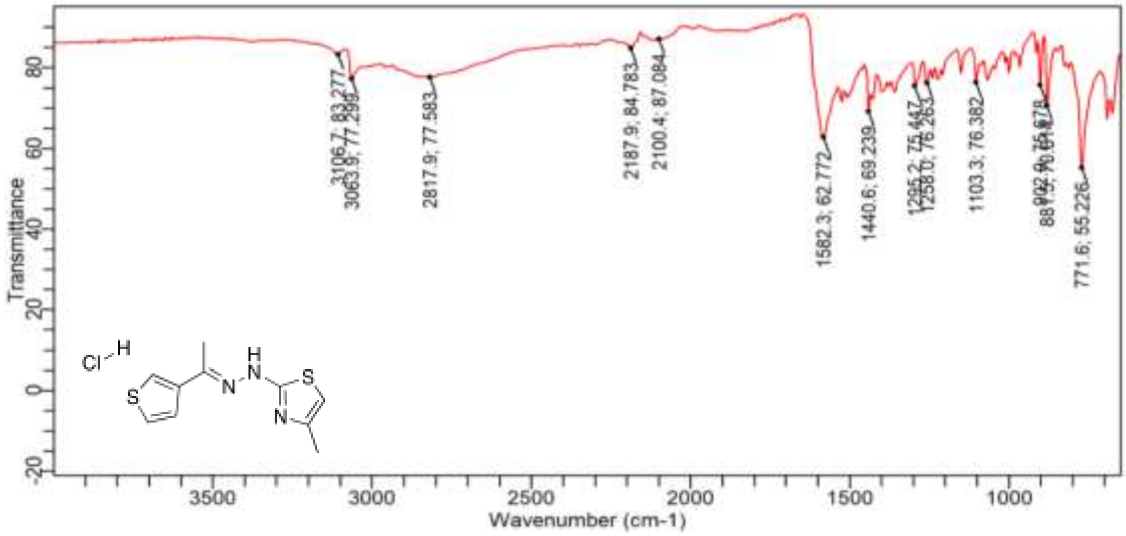

Figure S68. IR (up) and ^{1}H-NMR (down) of compound ***S*C8**.

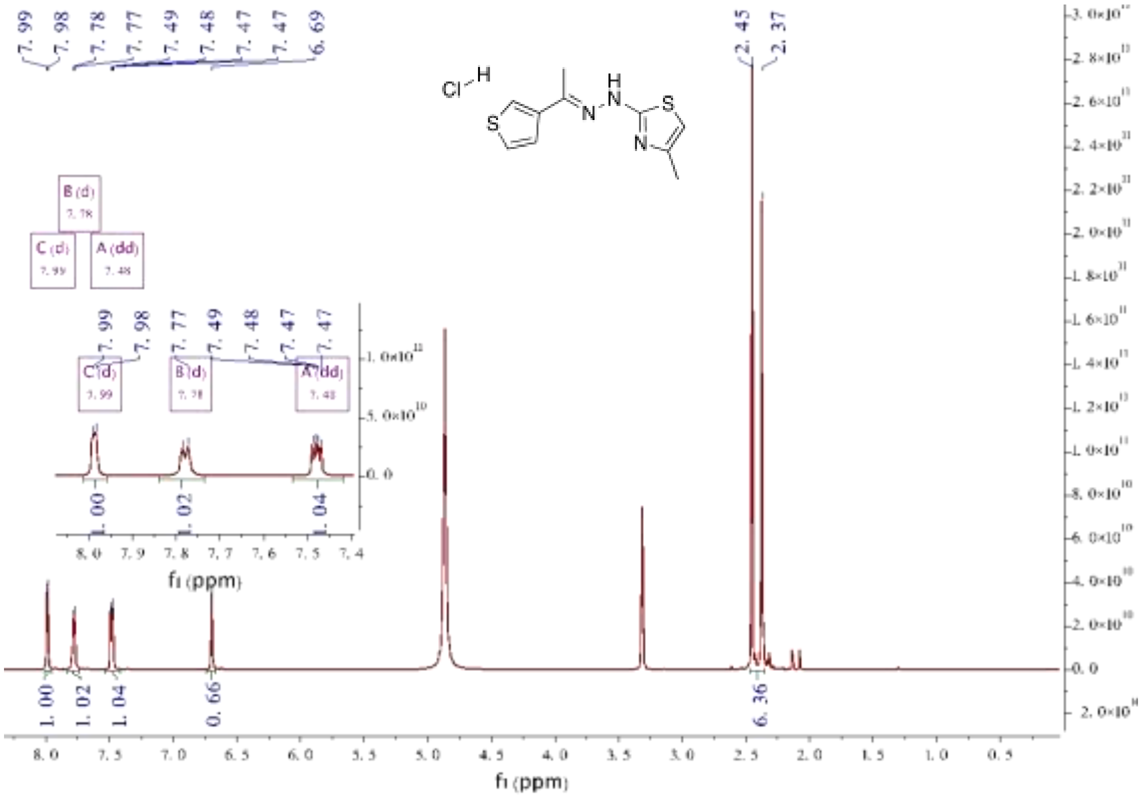

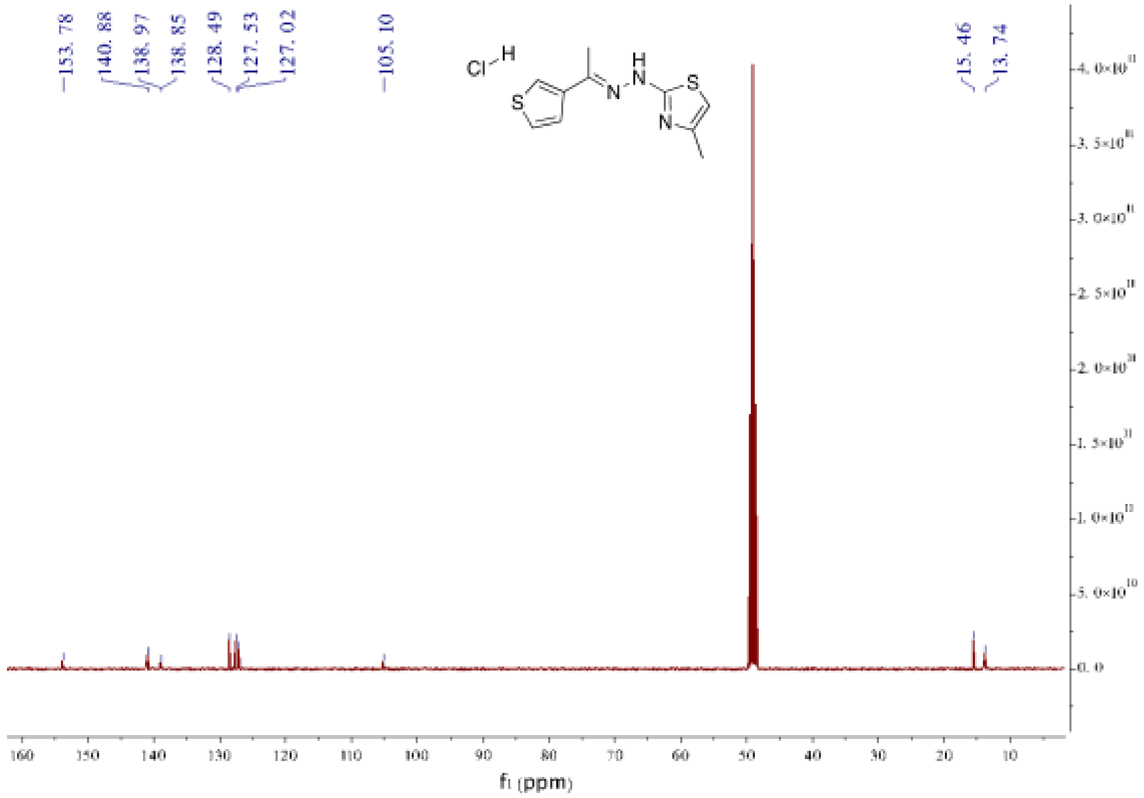

Figure S69. ^{13}C-NMR (up) and qNMR (down) of compound ***S*C8**.

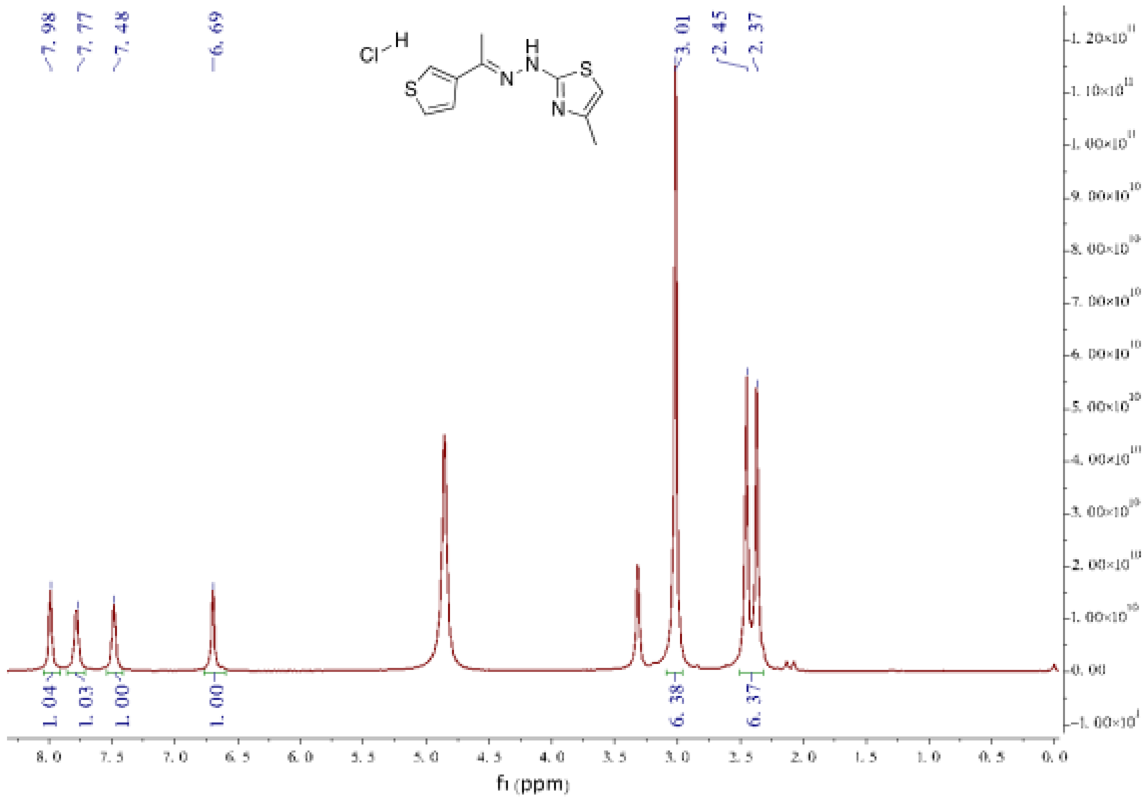

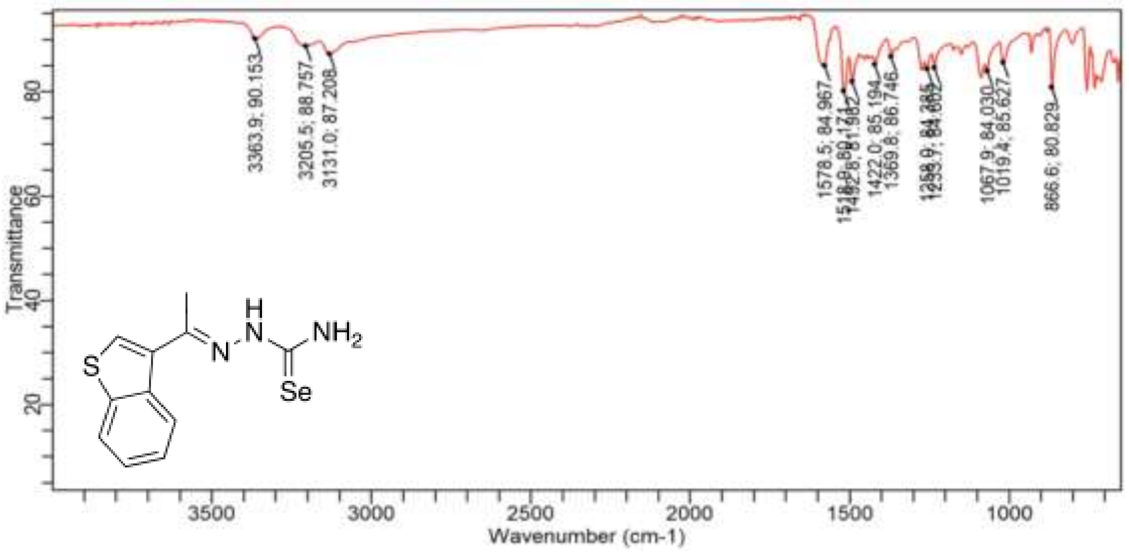

Figure S70. IR (up) and ^{1}H-NMR (down) of compound ***Se*O9**.

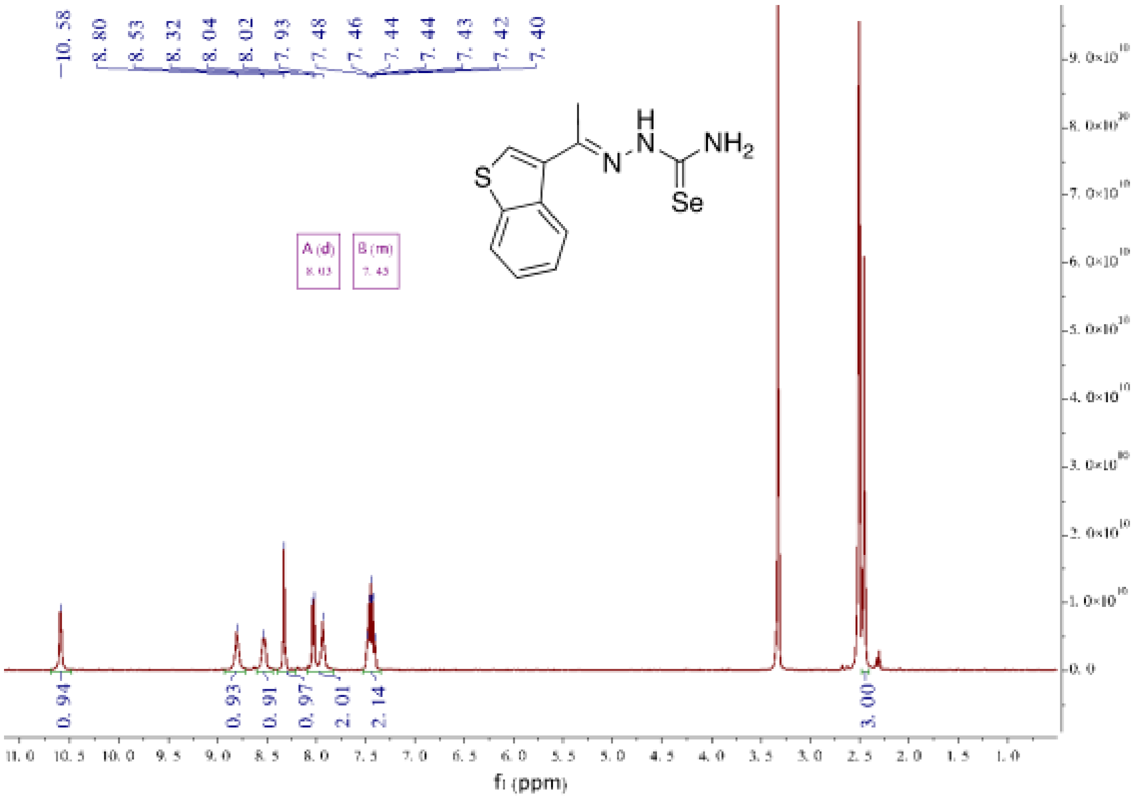

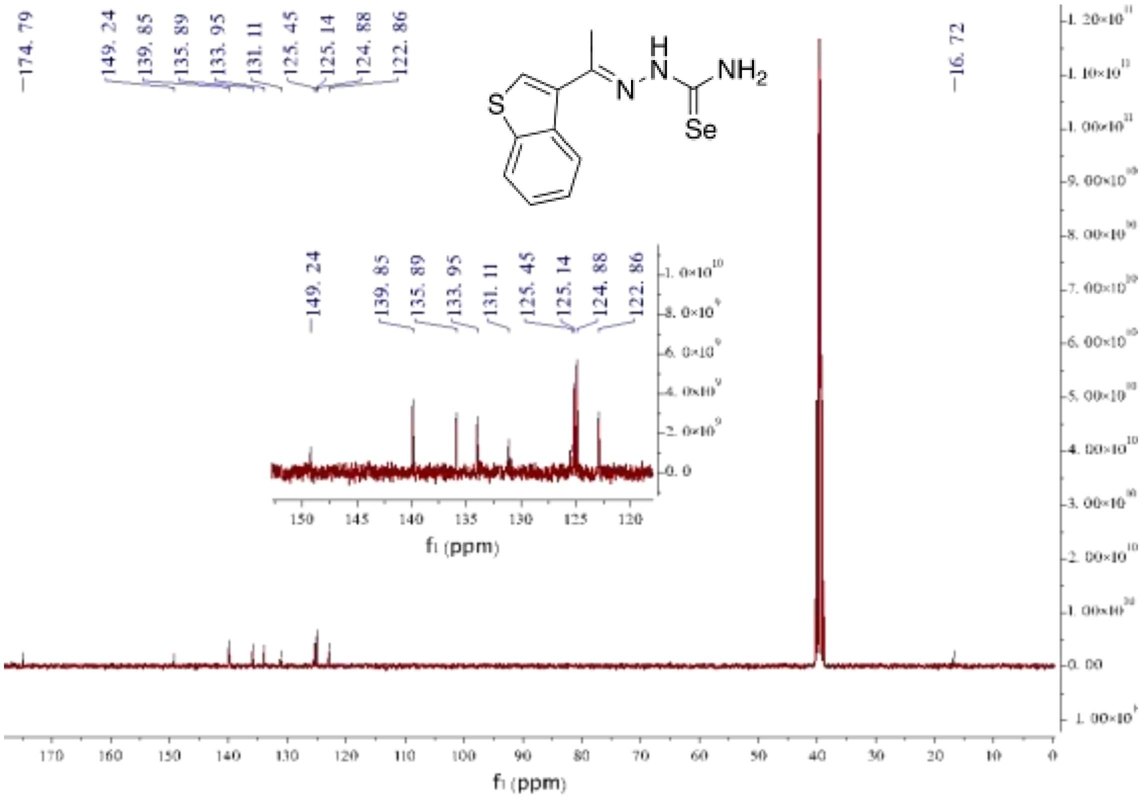

Figure S71. ^{13}C-NMR (up) and ^{77}Se-NMR (down) of compound ***Se*O9**.

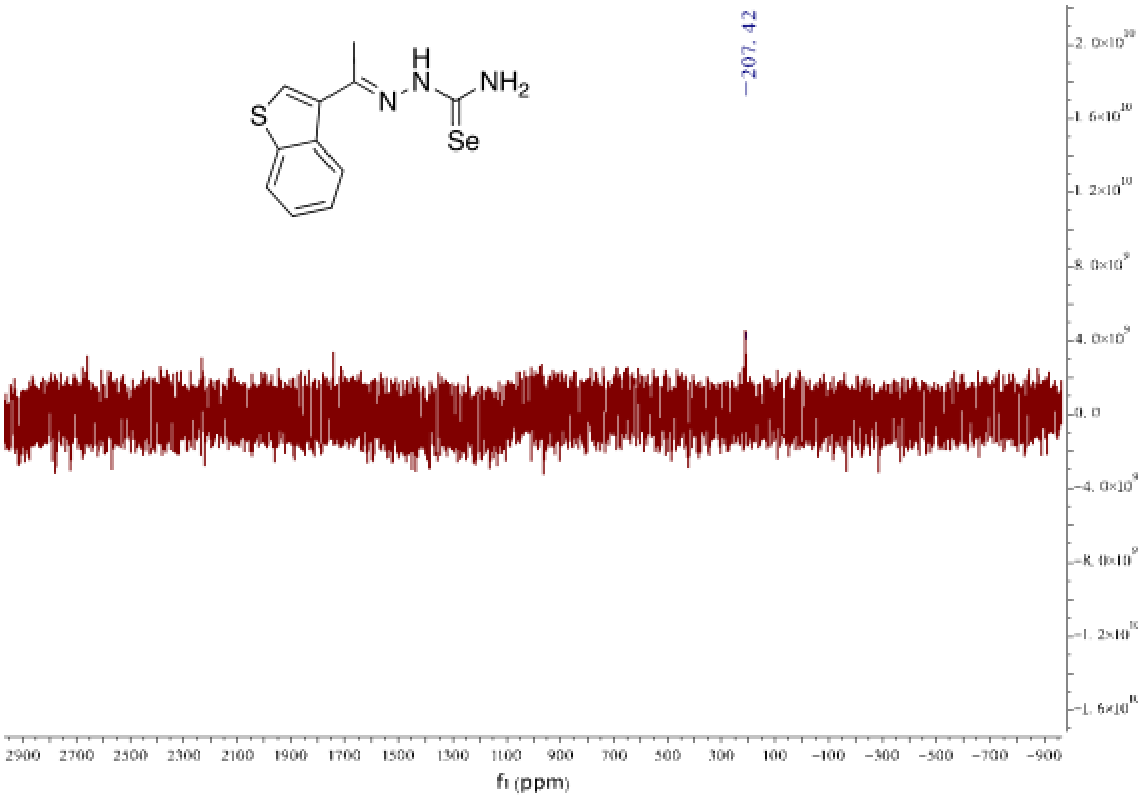

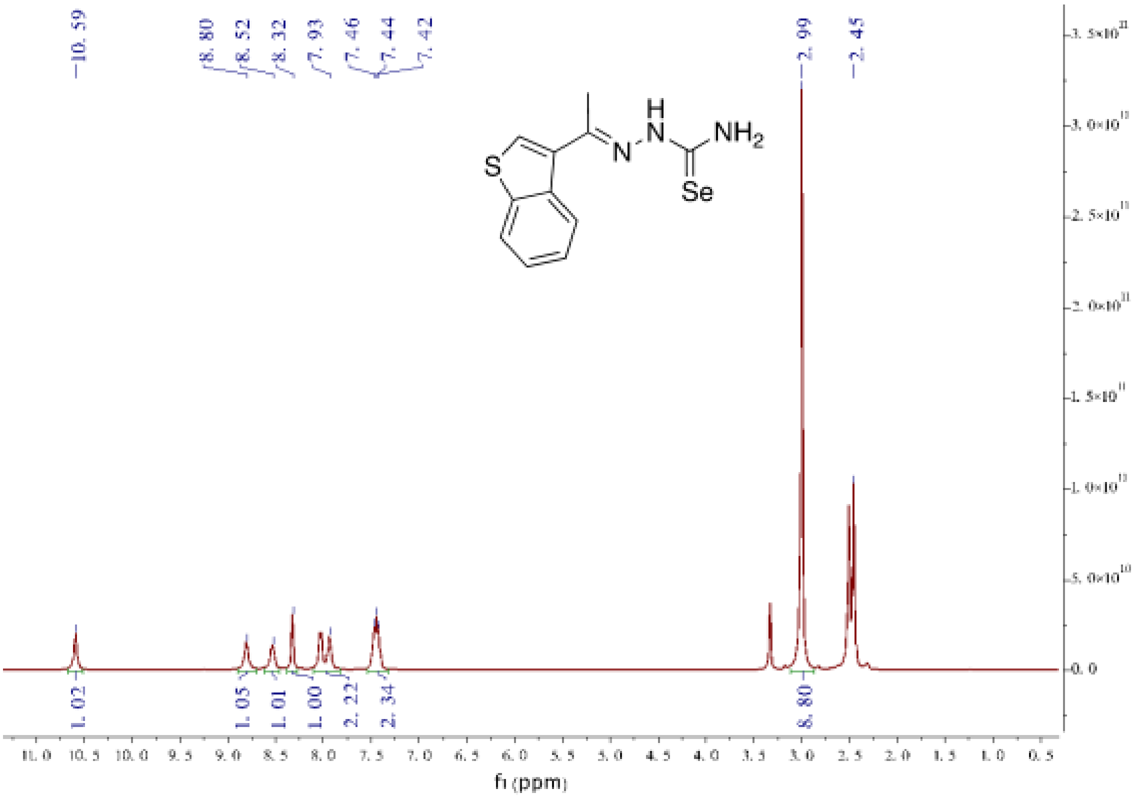

Figure S72. qNMR (up) of compound ***Se*O9** and IR (down) of compound ***Se*C9**.

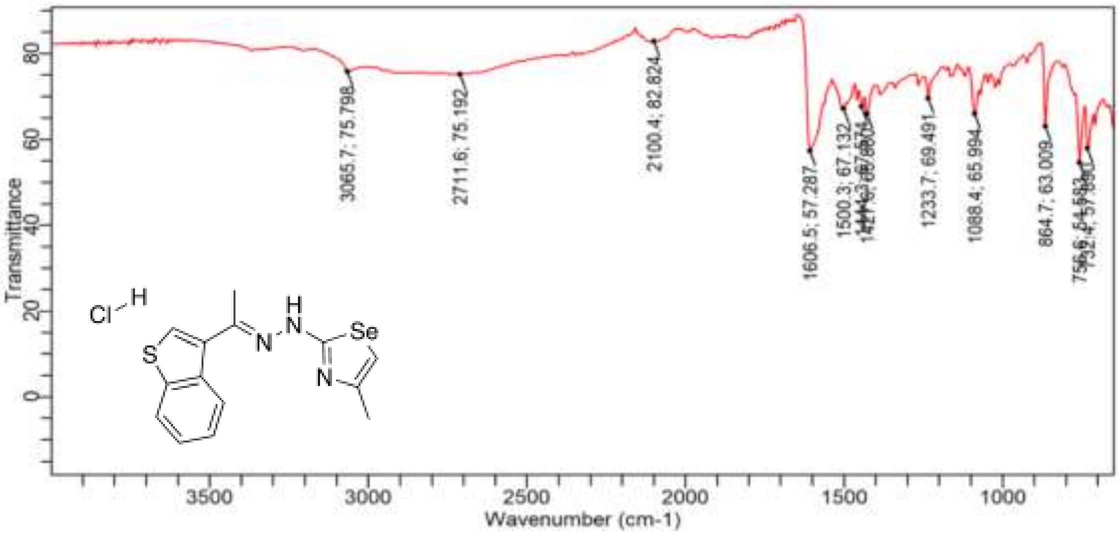

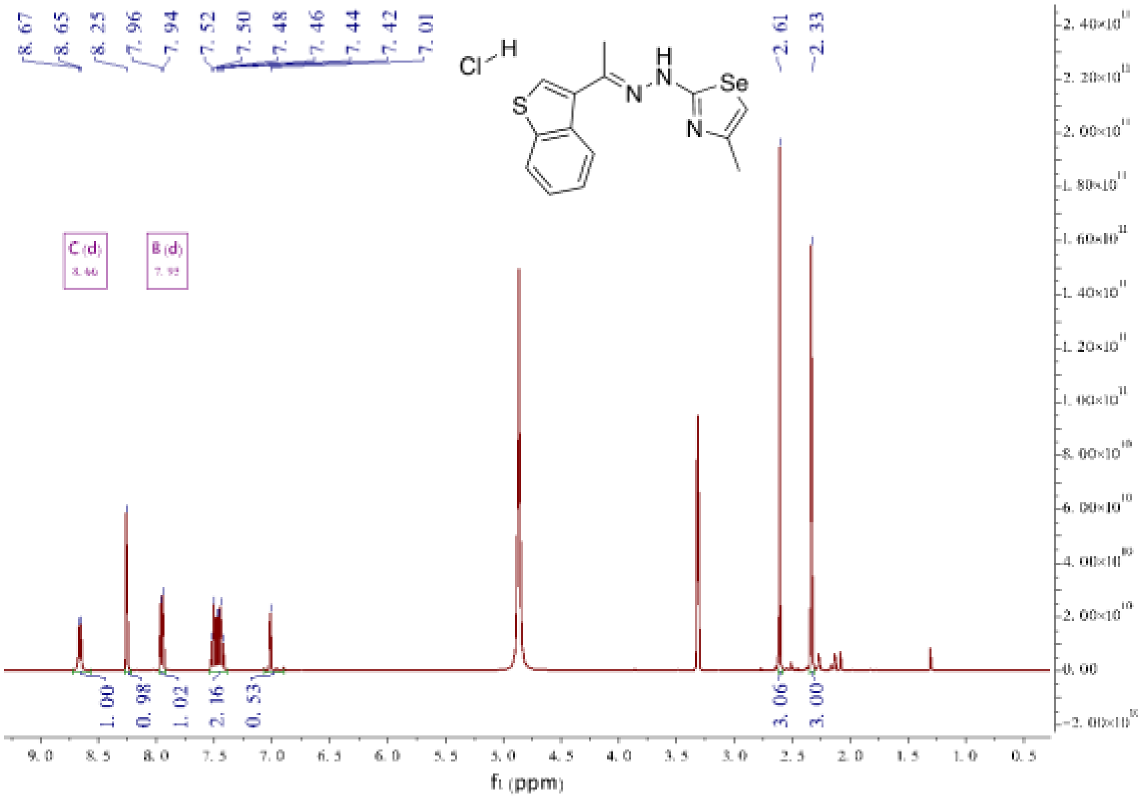

Figure S73. ^{1}H-NMR (up) and ^{13}C-NMR (down) of compound ***Se*C9**.

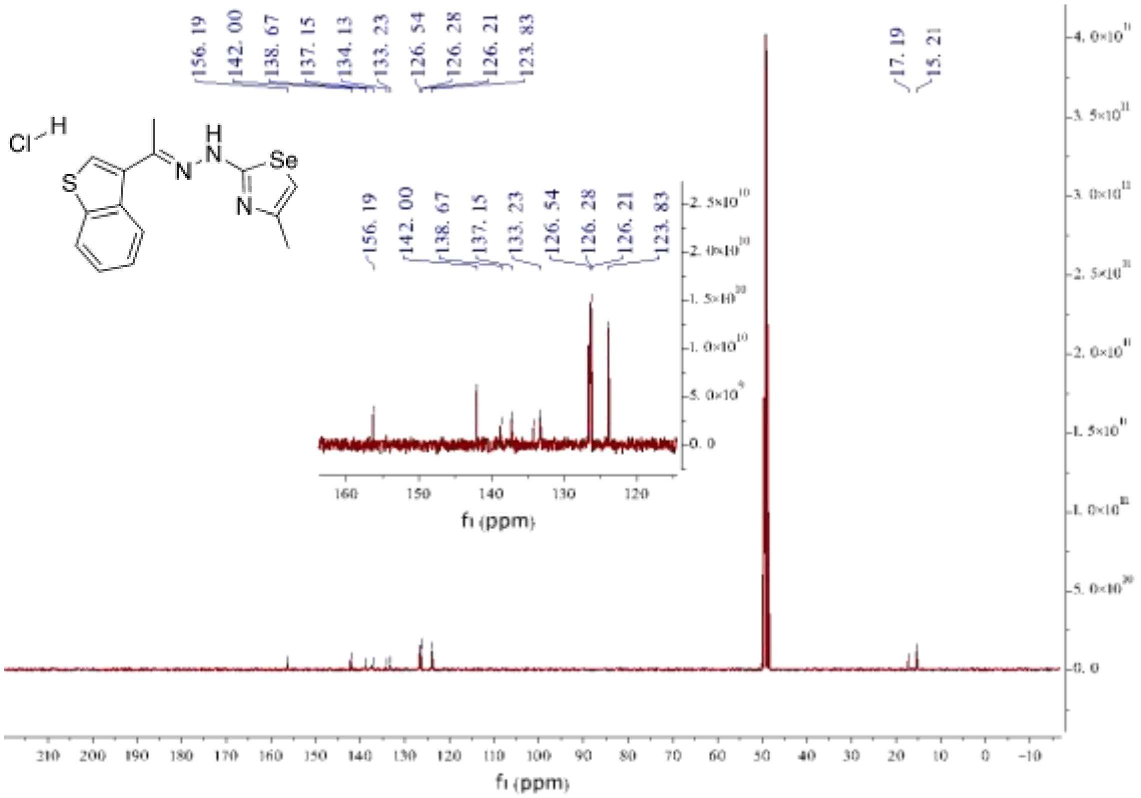

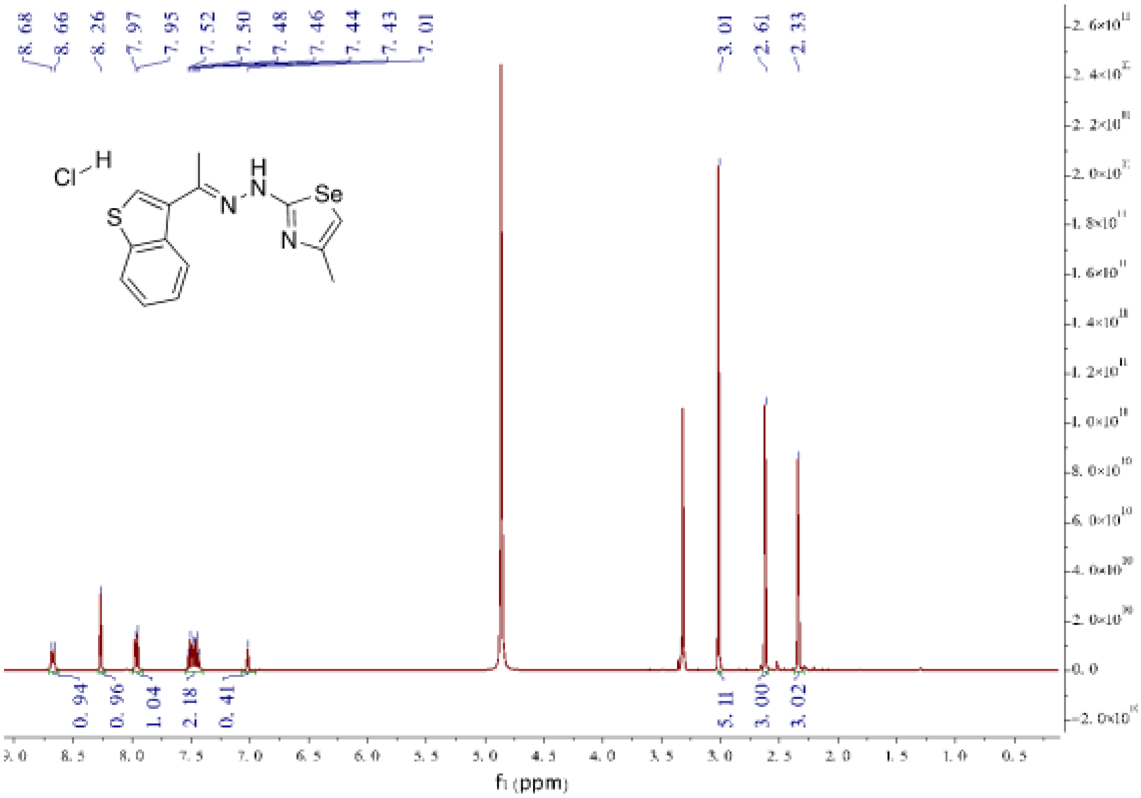

Figure S74. qNMR (up) of compound ***Se*C9** and IR (down) of compound **SO9**.

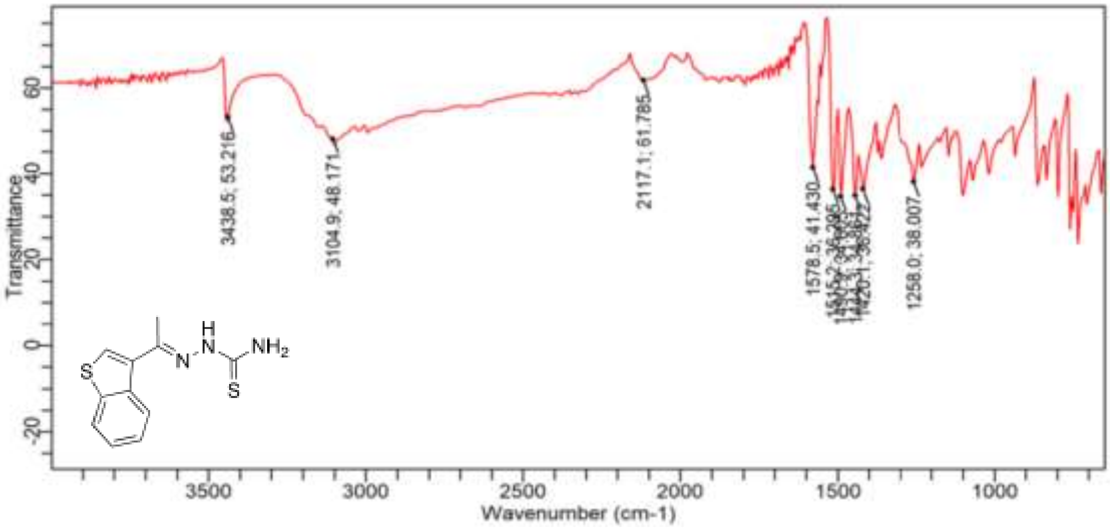

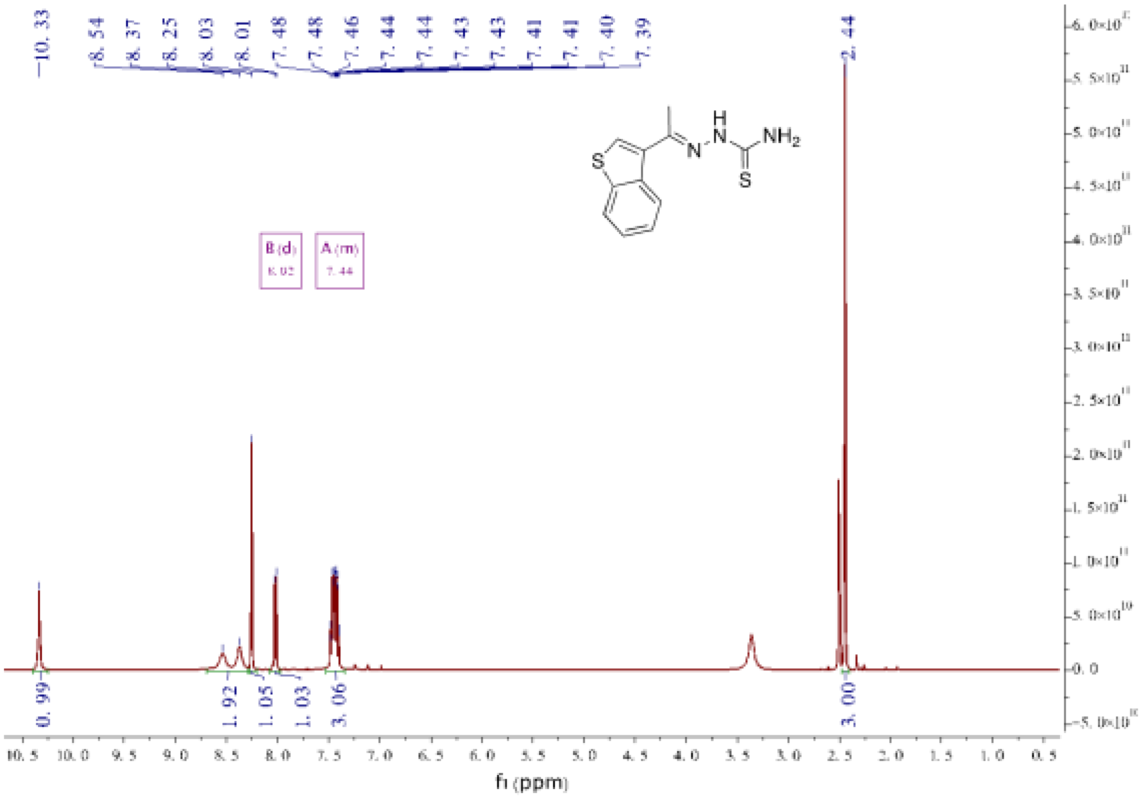

Figure S75. ^{1}H-NMR (up) and ^{13}C-NMR (down) of compound **SO9**.

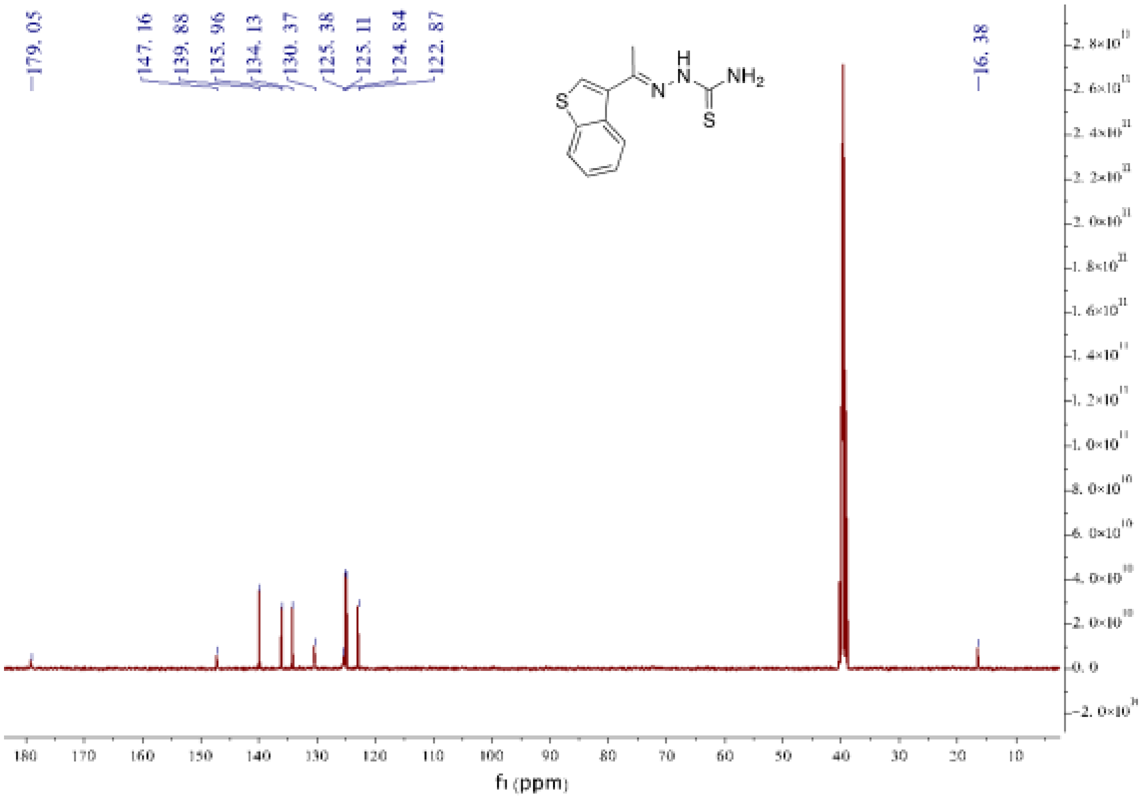

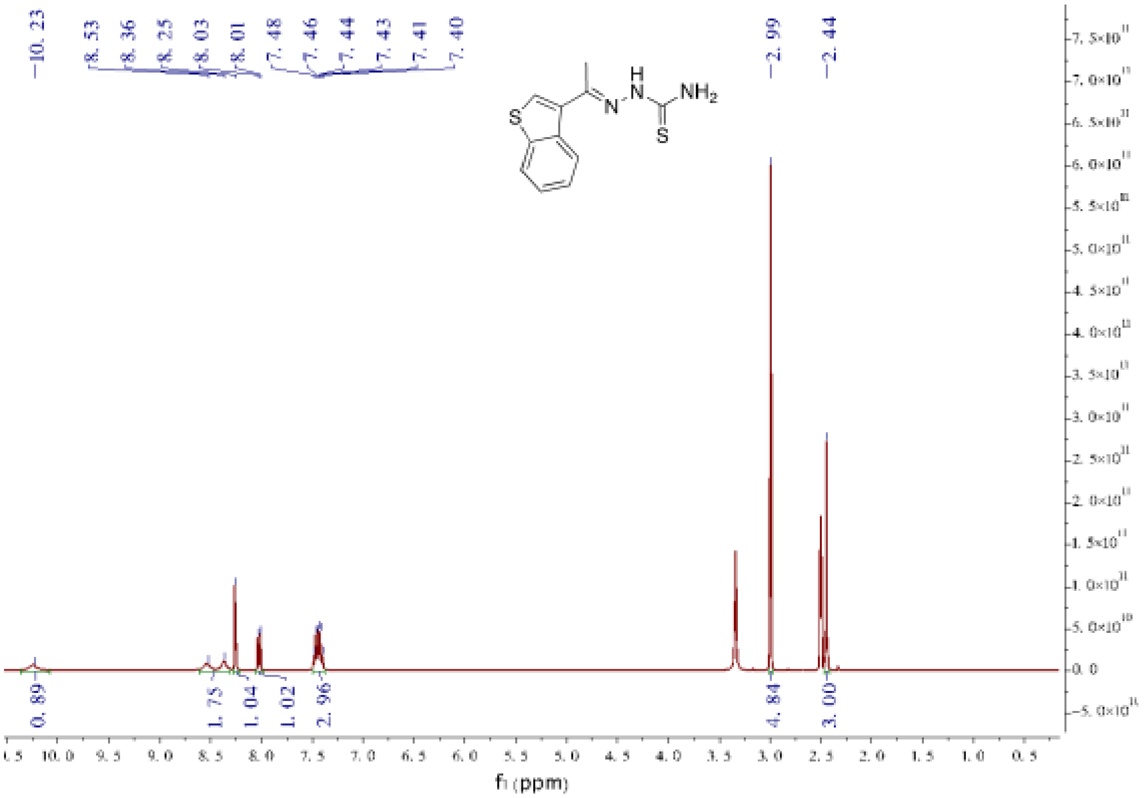

Figure S76. qNMR (up) of compound **SO9** and IR (down) of compound ***S*C9**.

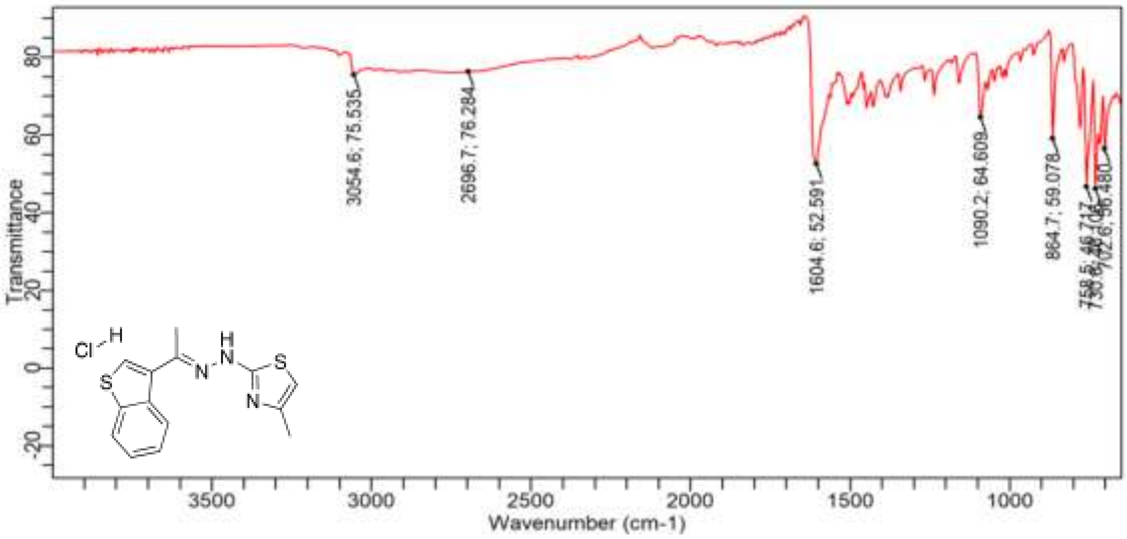

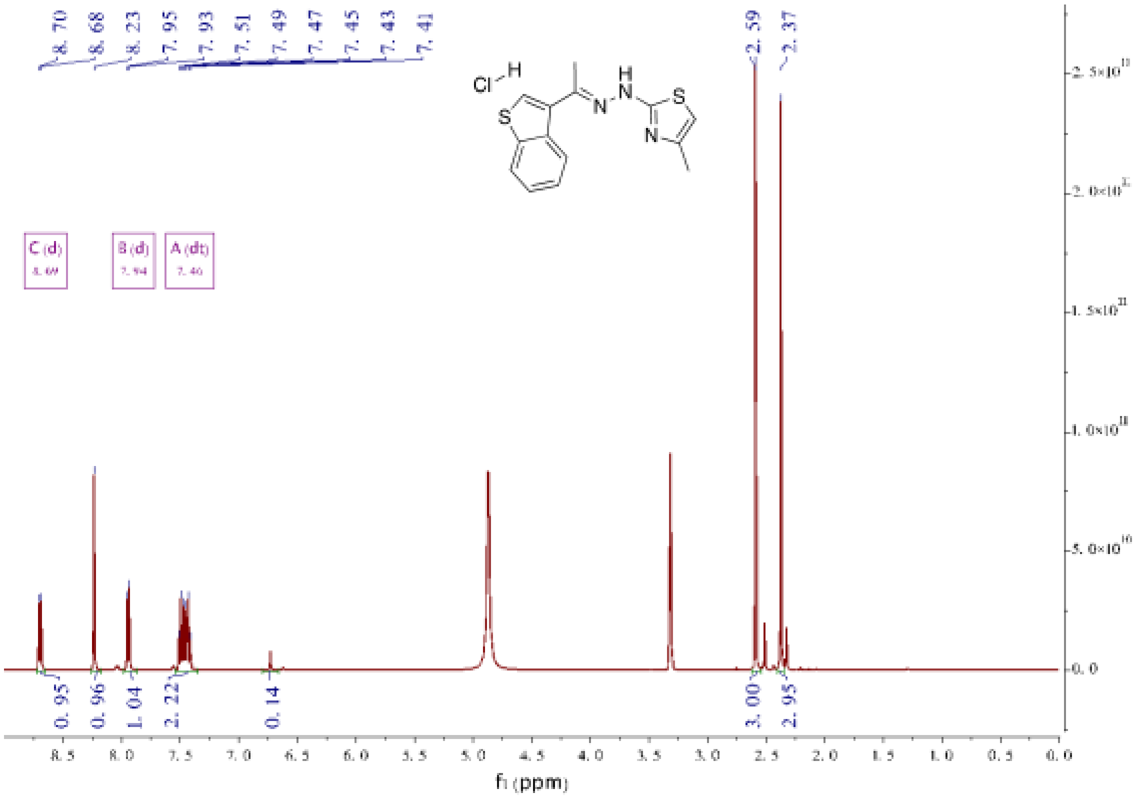

Figure S77. ^{1}H-NMR (up) and ^{13}C-NMR (down) of compound ***SC*9**.

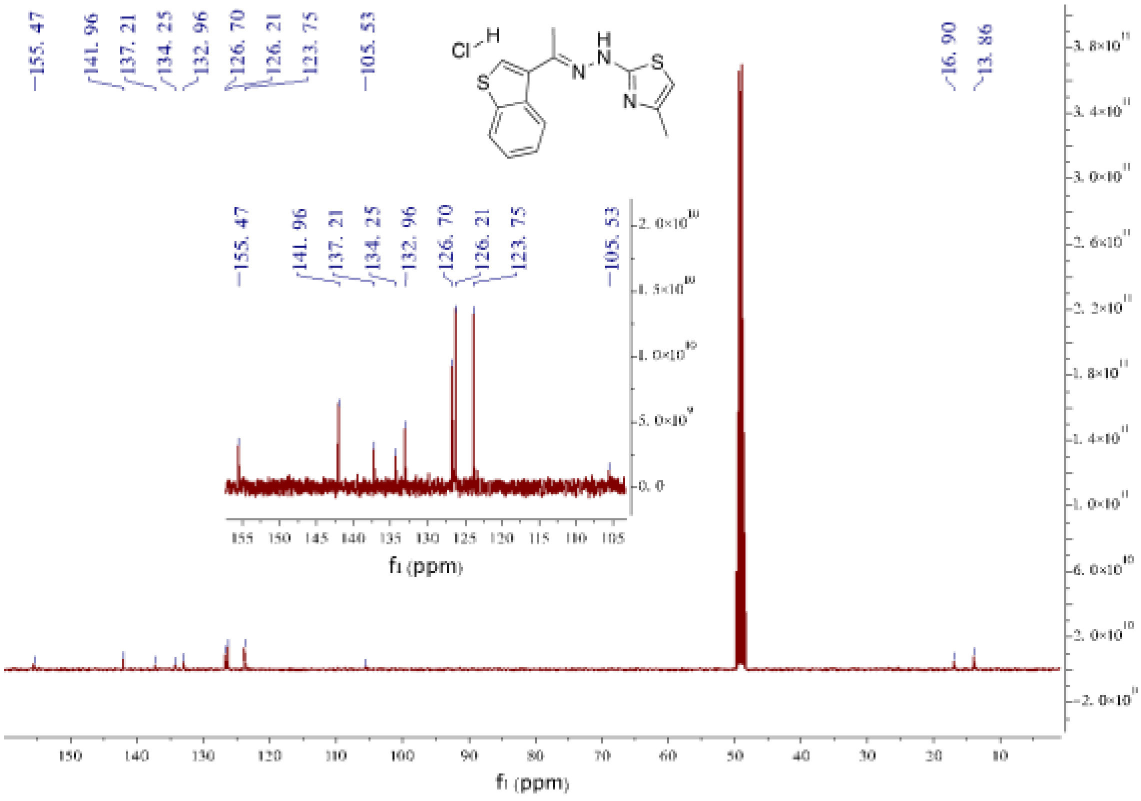

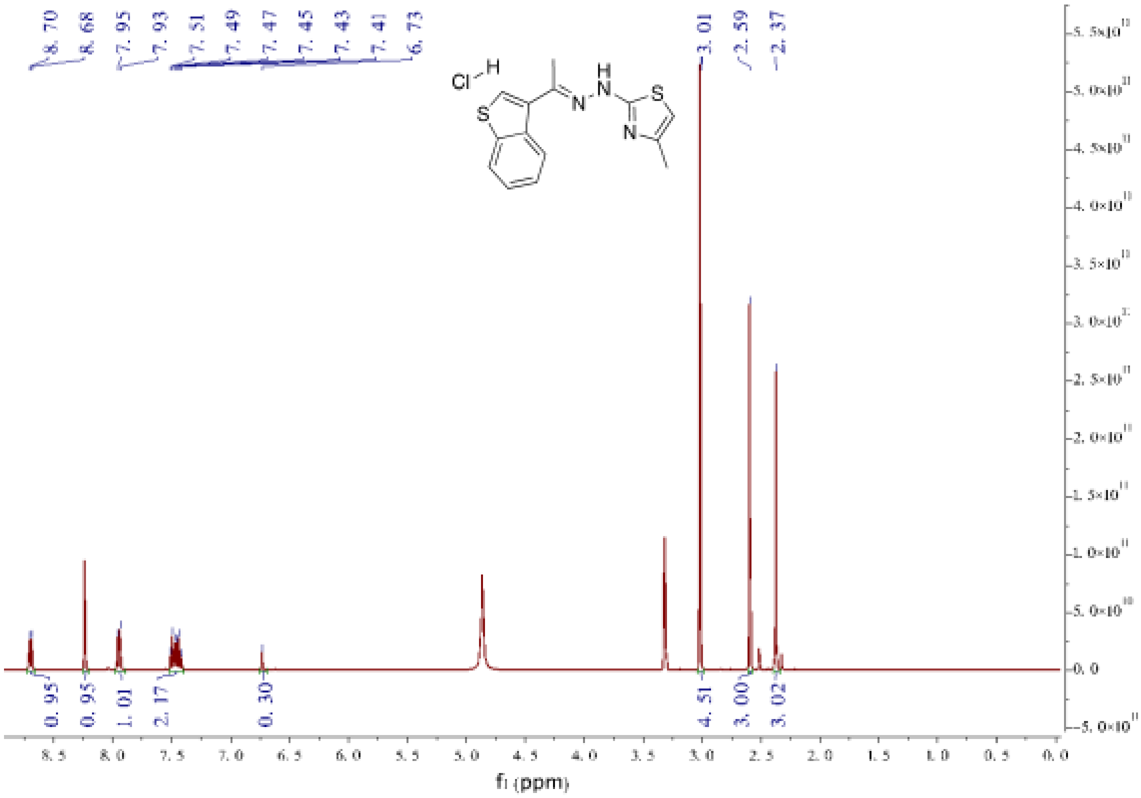

Figure S78. qNMR (up) of compound ***S*C9** and IR (down) of compound ***Se*O10**.

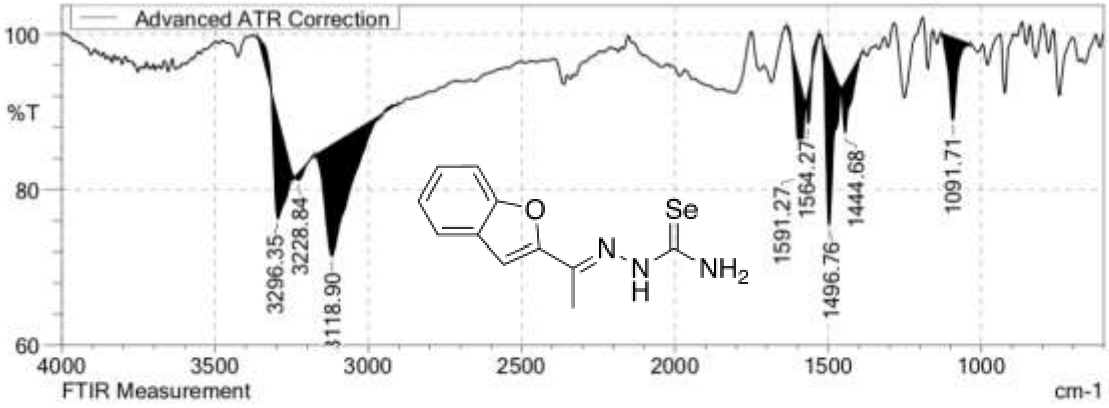

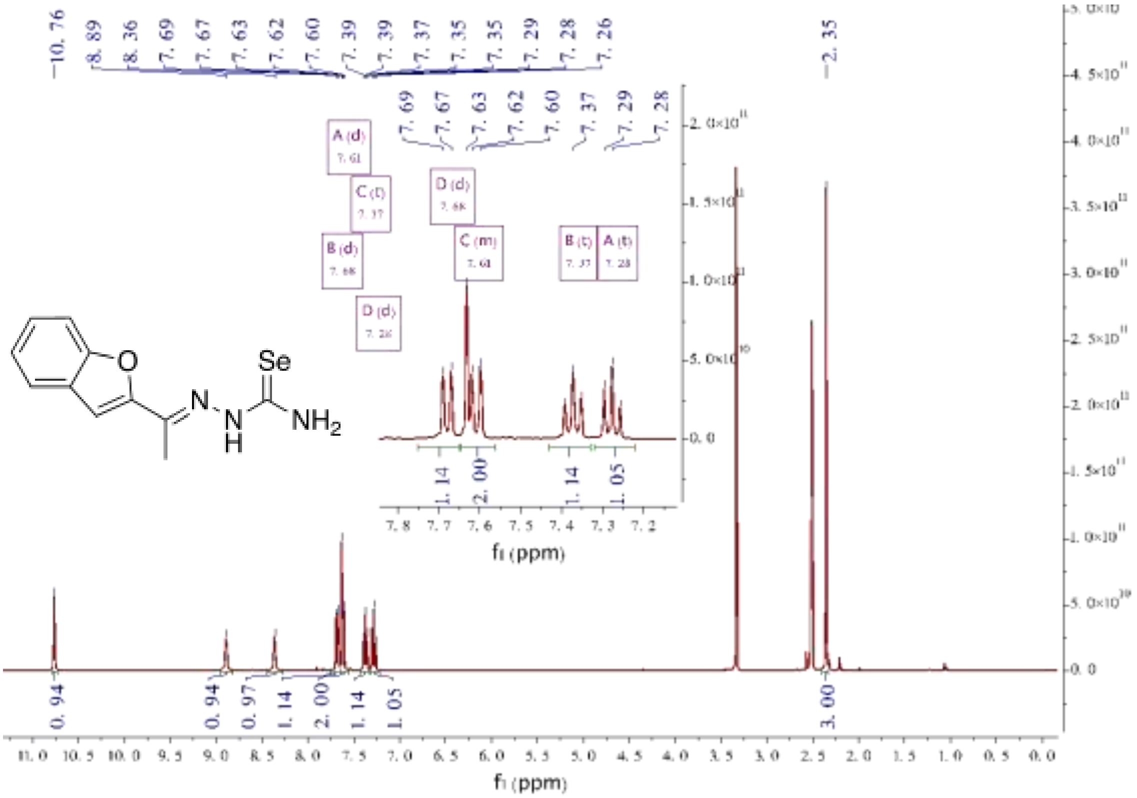

Figure S79. ^{1}H-NMR (up) and ^{13}C-NMR (down) of compound ***Se*O10**.

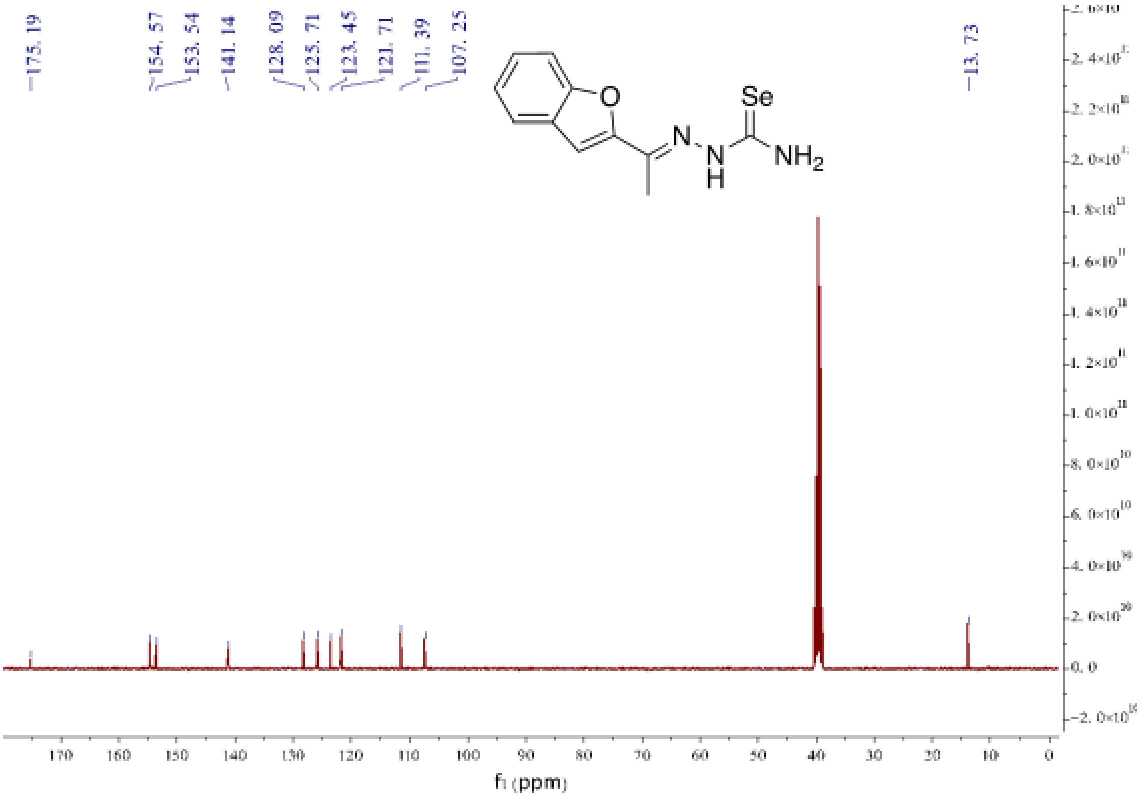

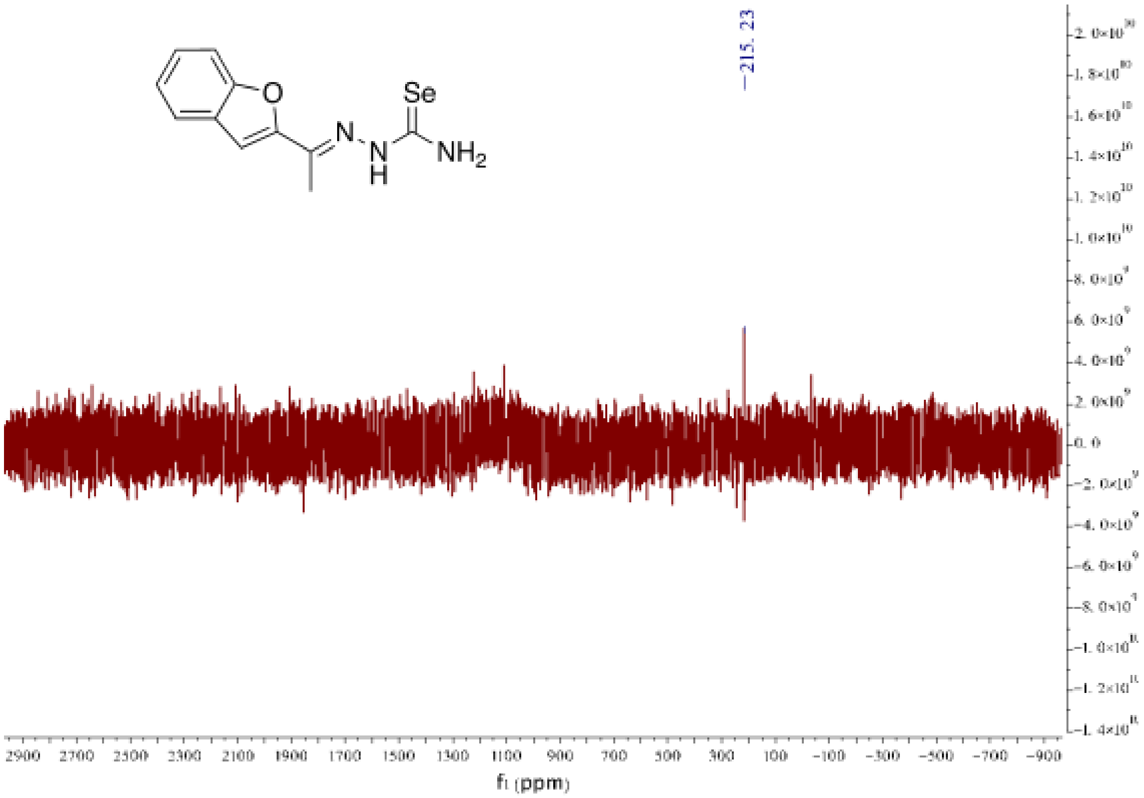

Figure S80. ^{77}Se-NMR (up) and qNMR (down) of compound ***Se*O10**.

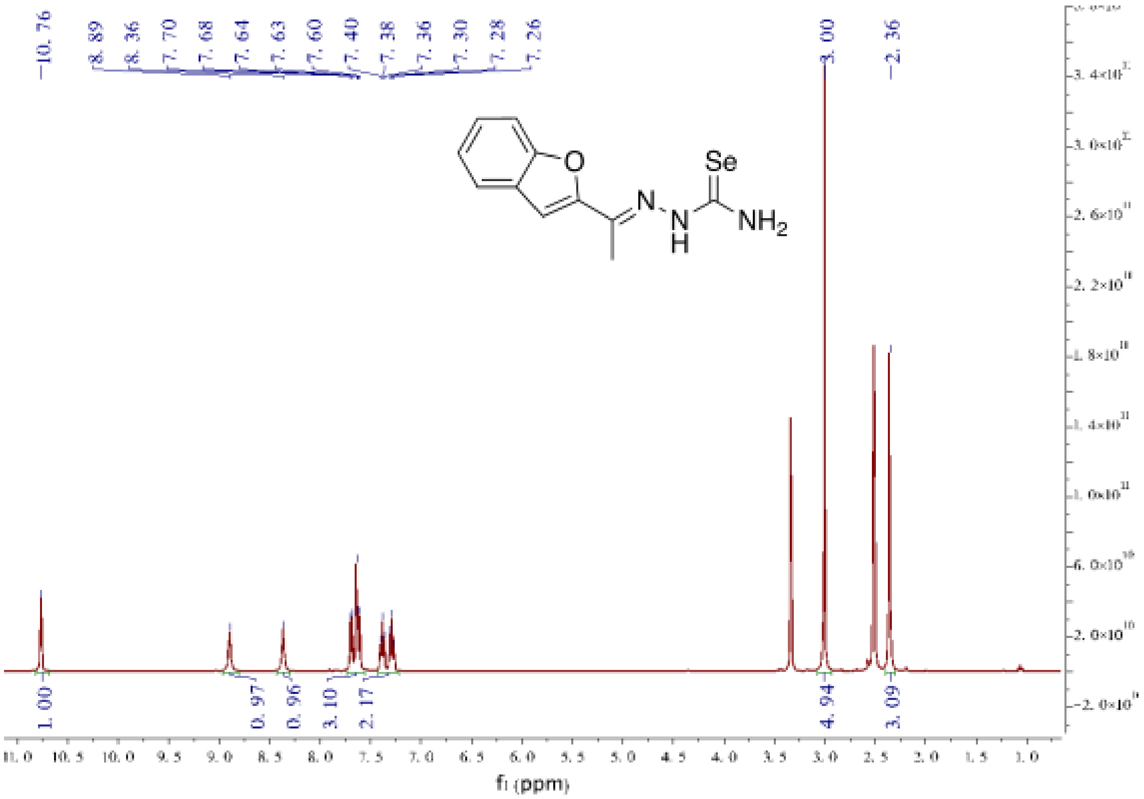

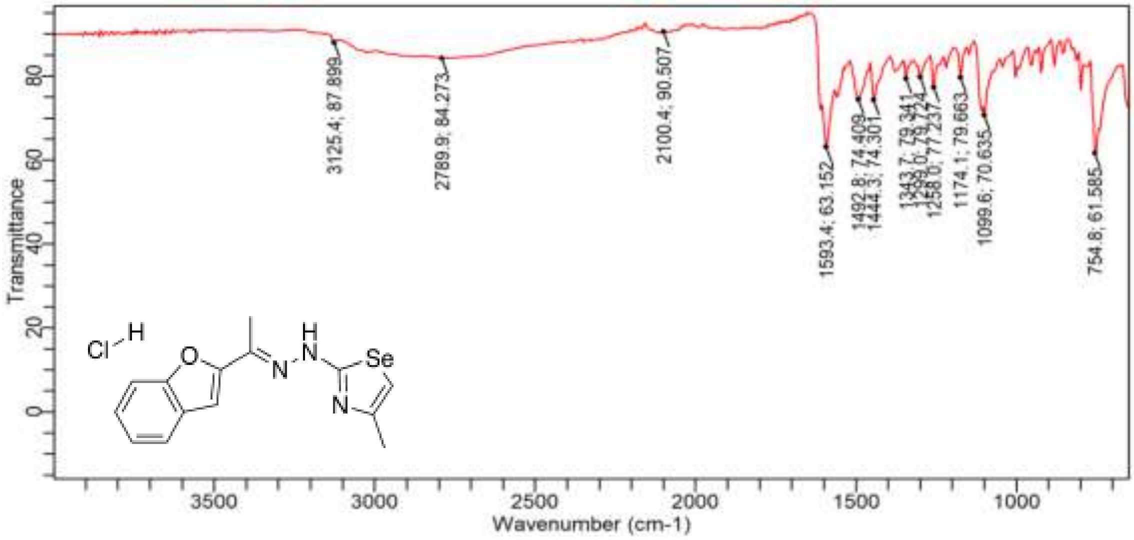

Figure S81. IR (up) and ^{1}H-NMR (down) of compound ***Se*C10**.

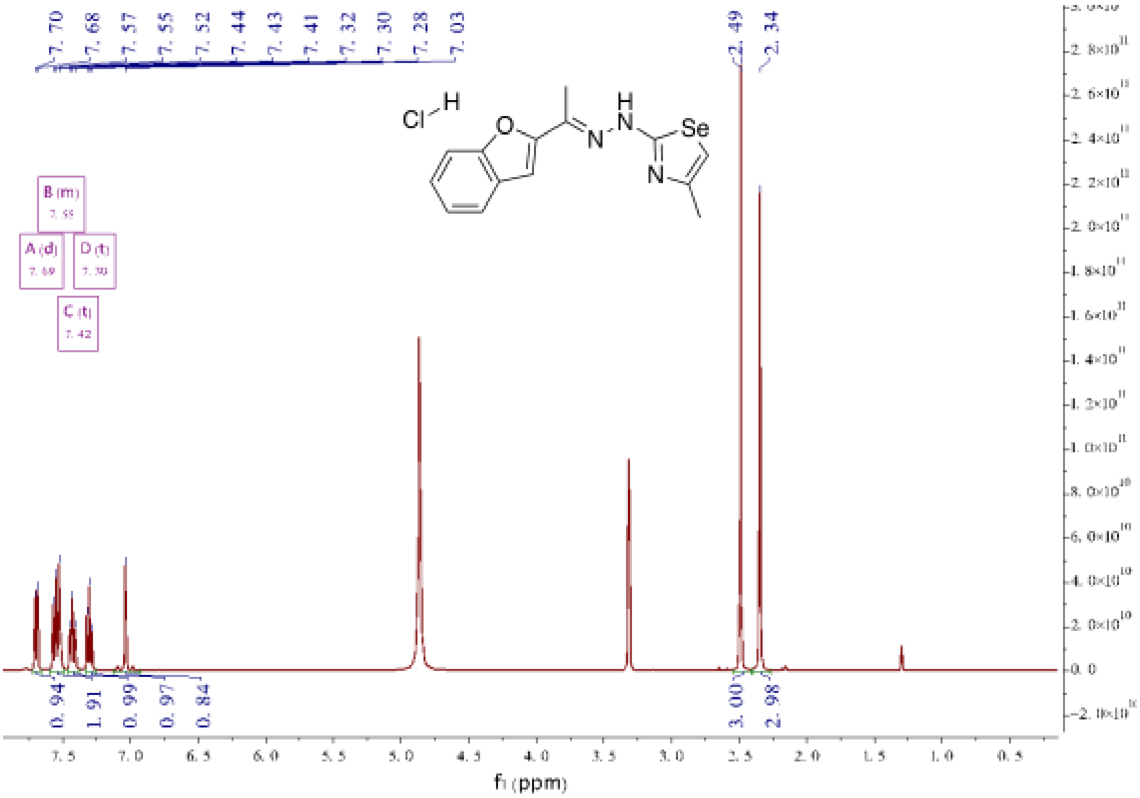

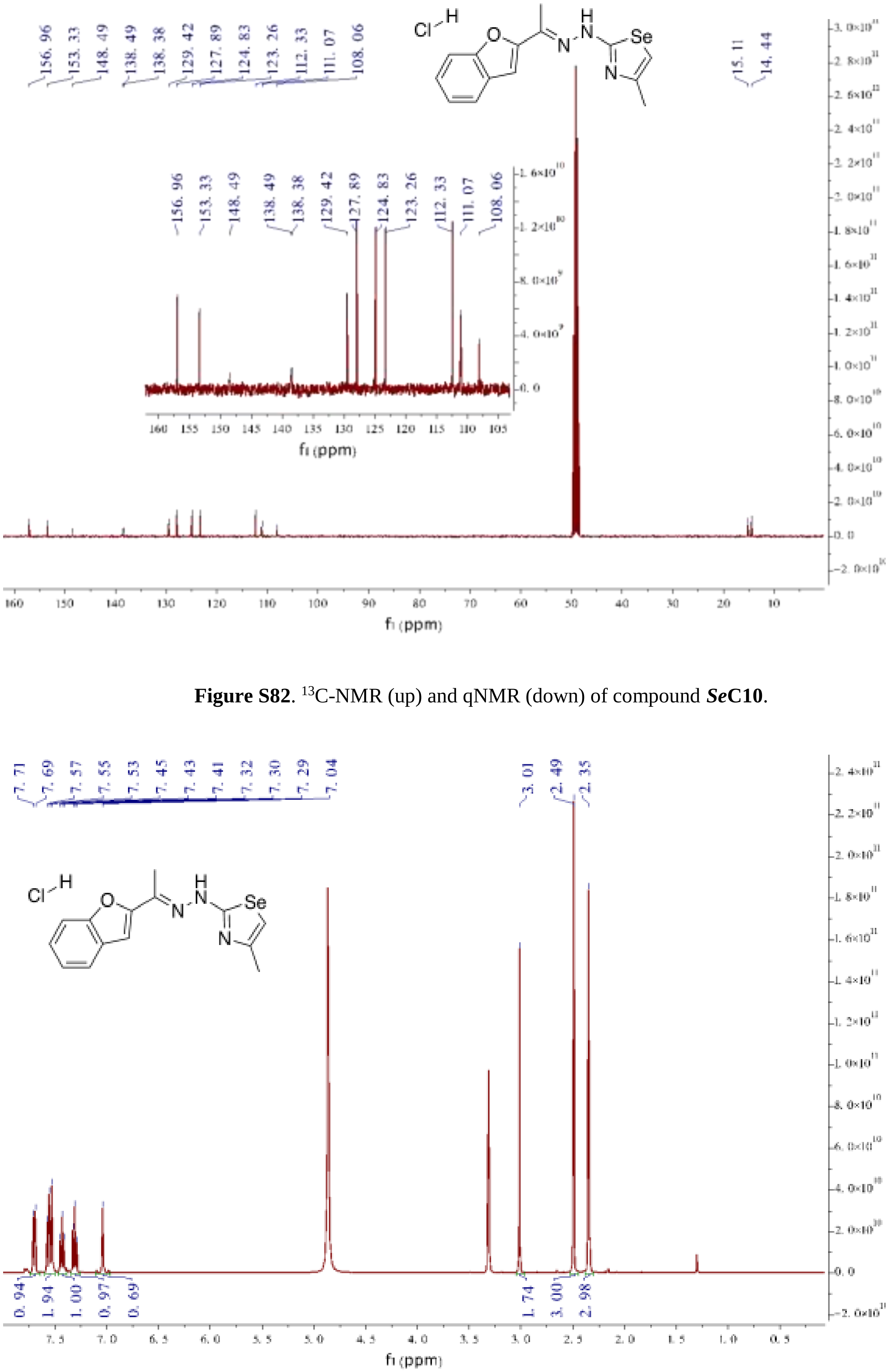

Figure S82. ^{13}C-NMR (up) and qNMR (down) of compound ***Se*C10**.

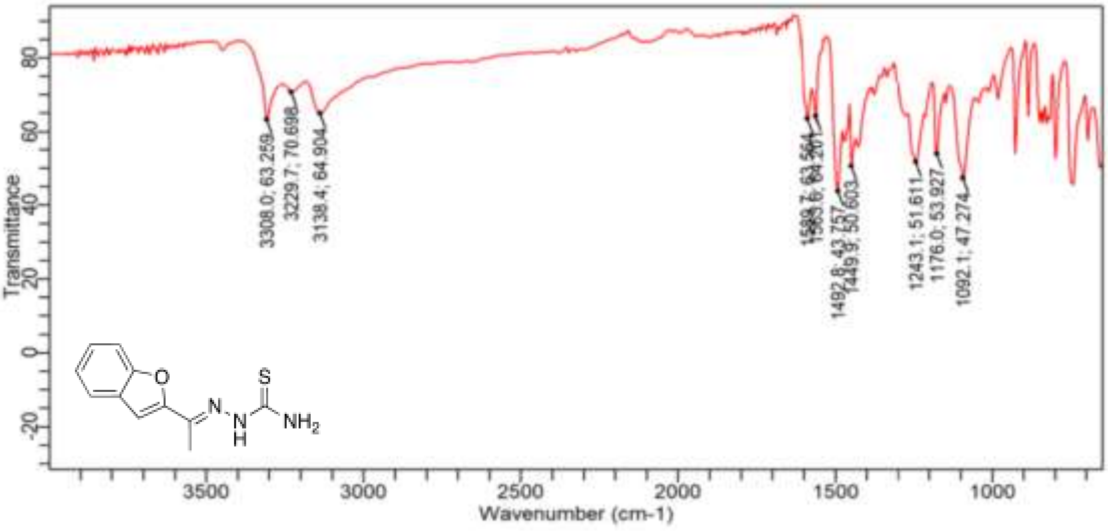

Figure S83. IR (up) and ^{1}H-NMR (down) of compound **SO10**.

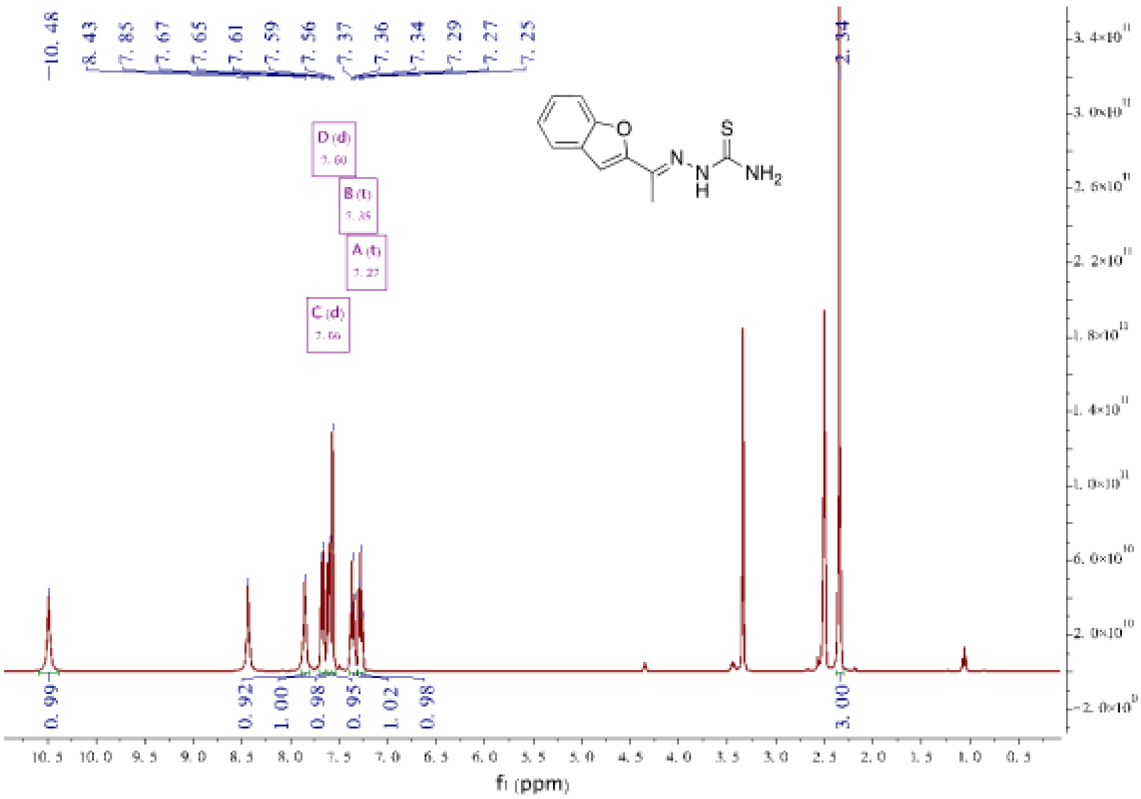

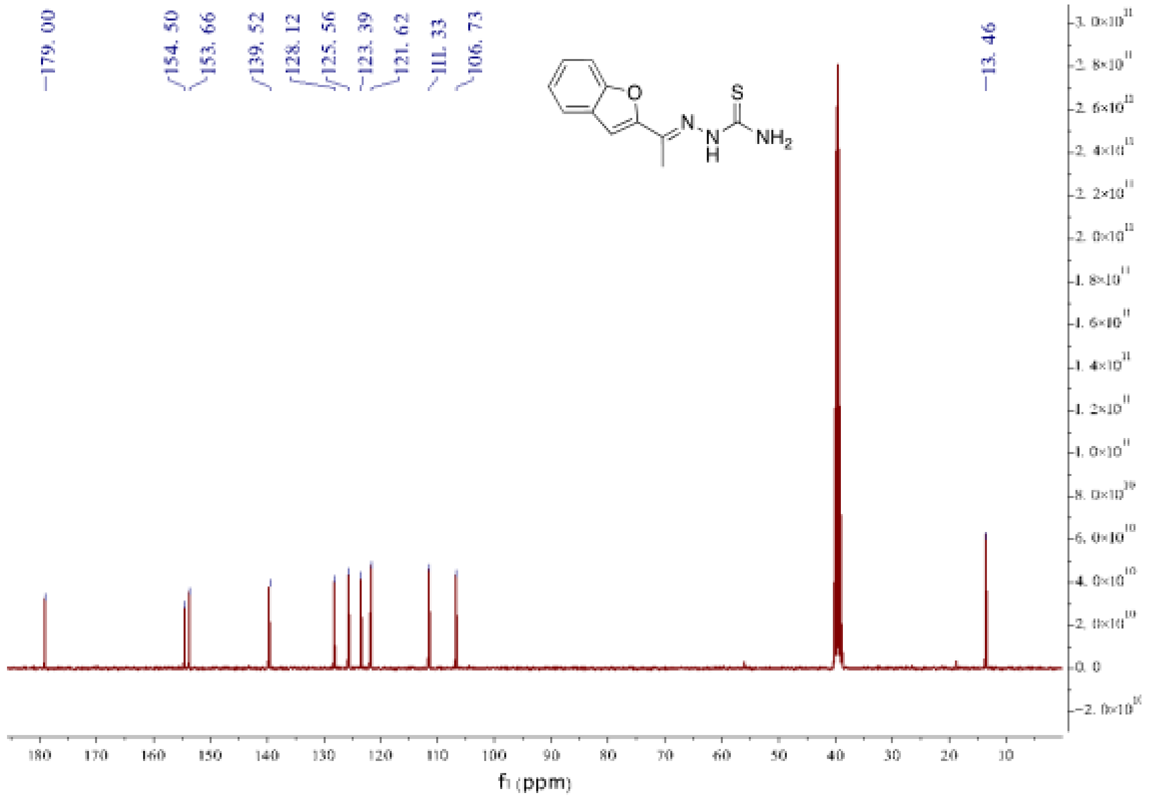

Figure S84. ^{13}C-NMR (up) and qNMR (down) of compound **SO10**.

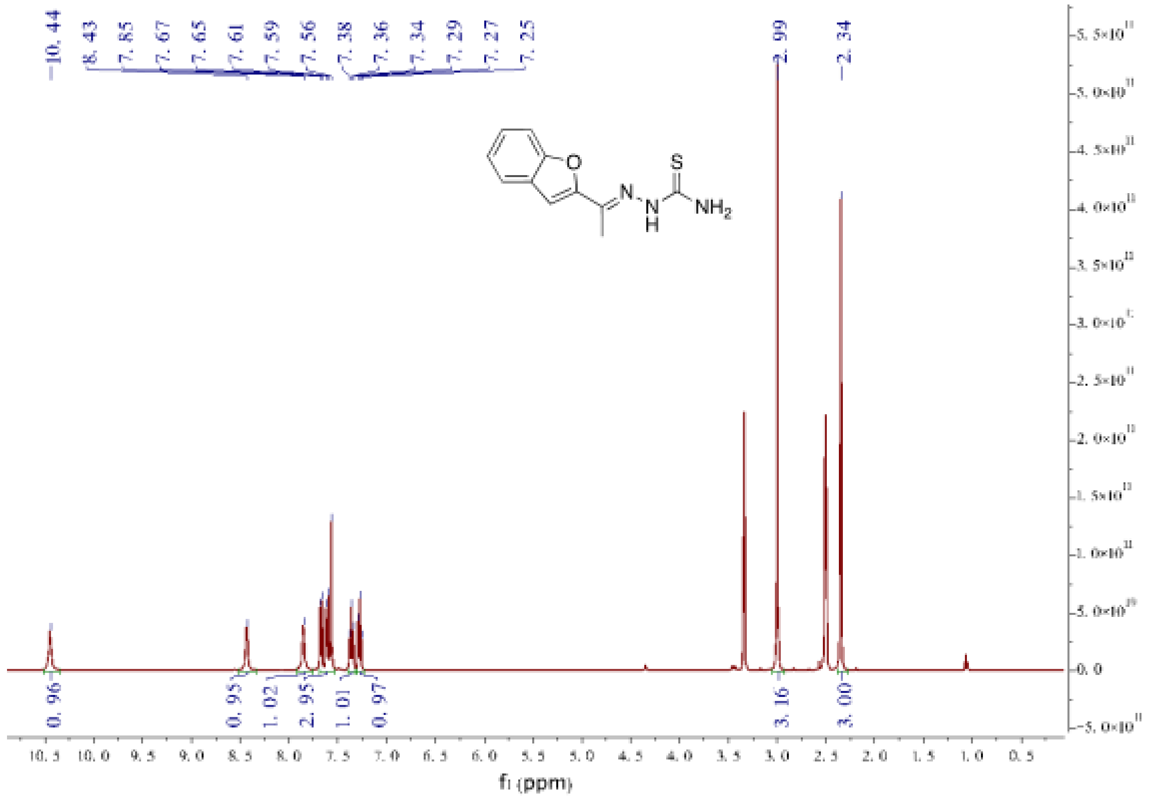

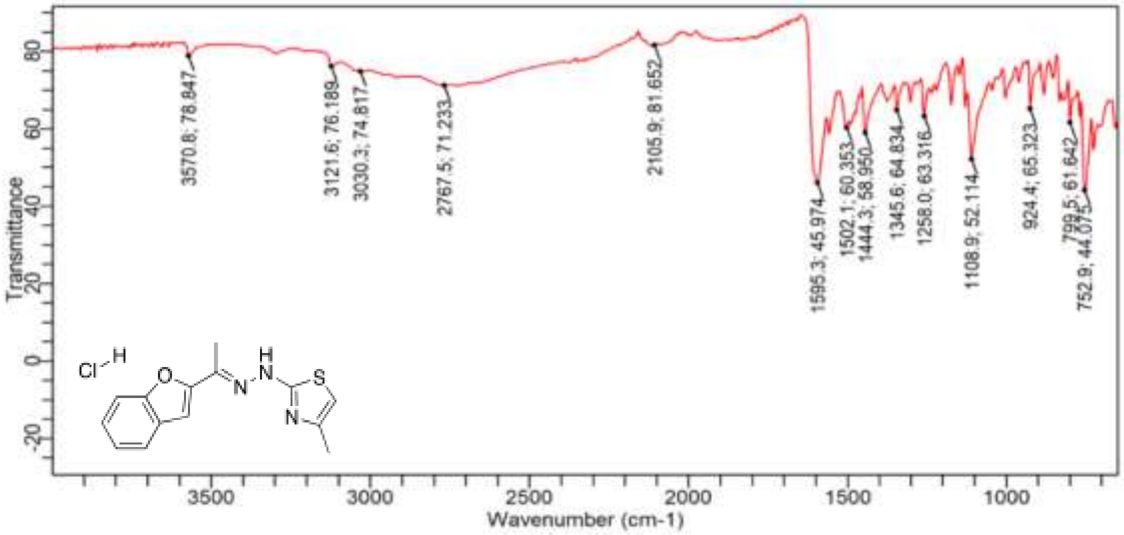

Figure S85. IR (up) and ^{1}H-NMR (down) of compound ***S*C10**.

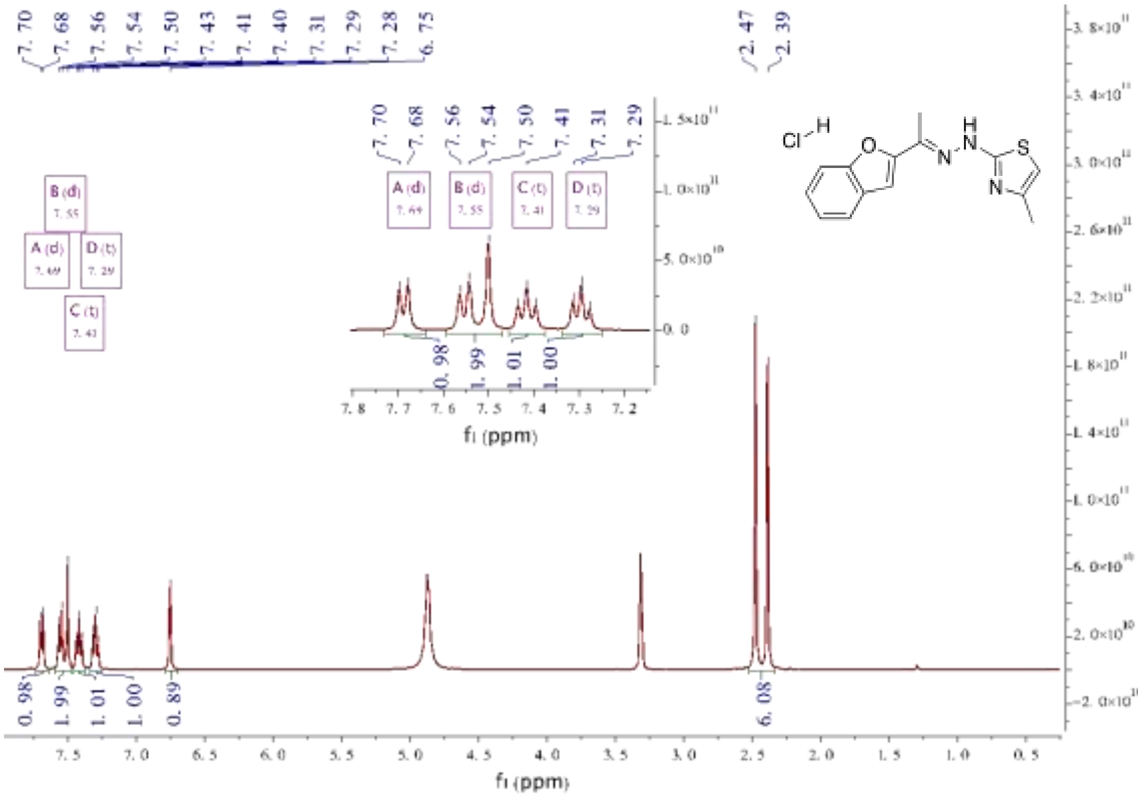

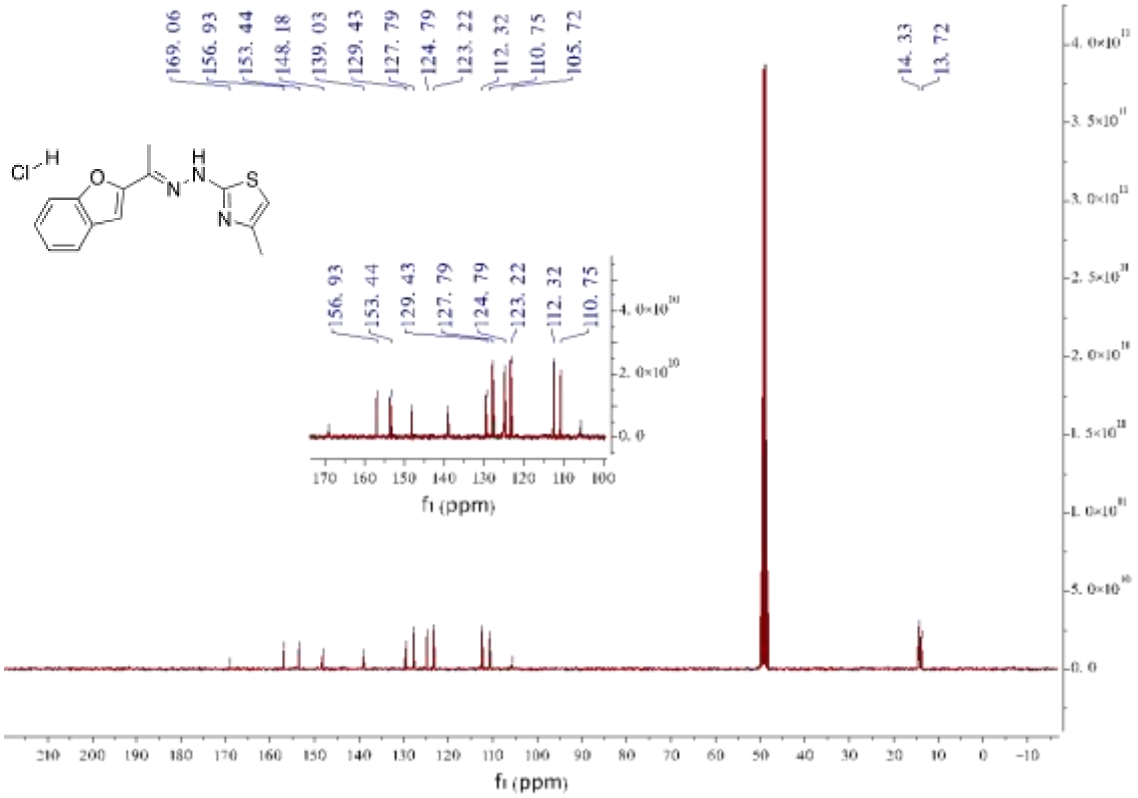

Figure S86. ^{13}C-NMR (up) and qNMR (down) of compound ***S*C10**.

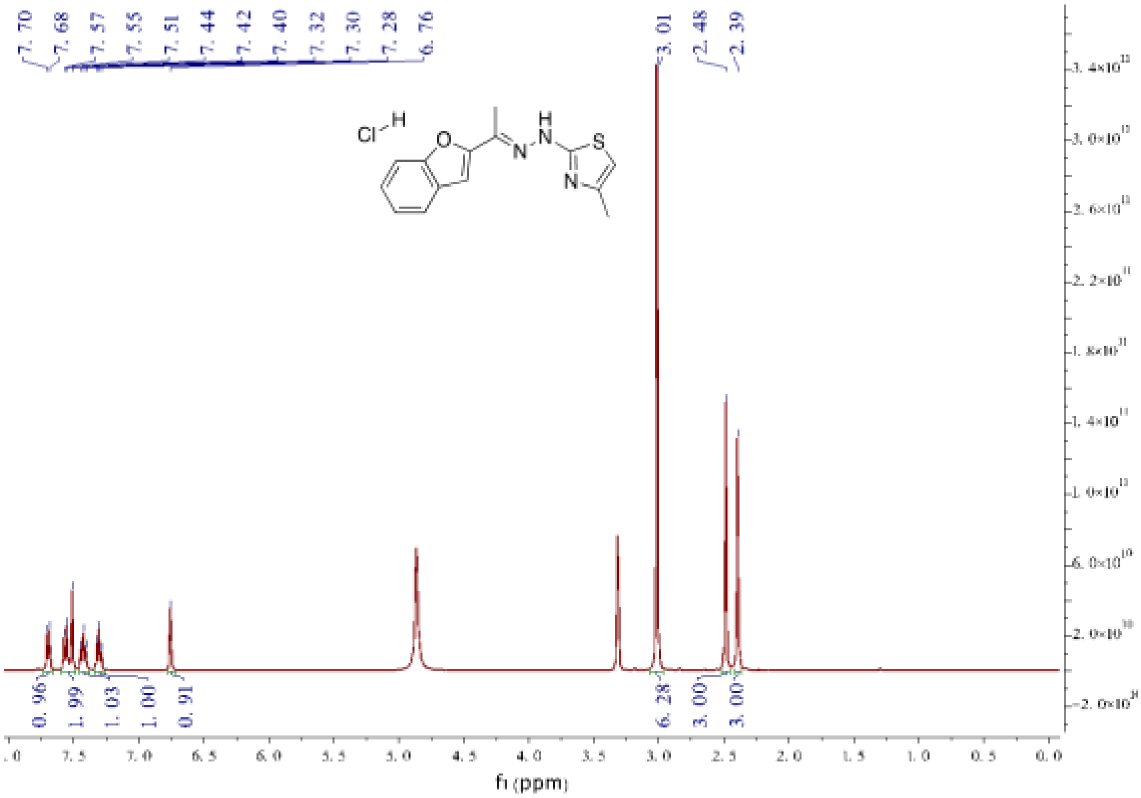

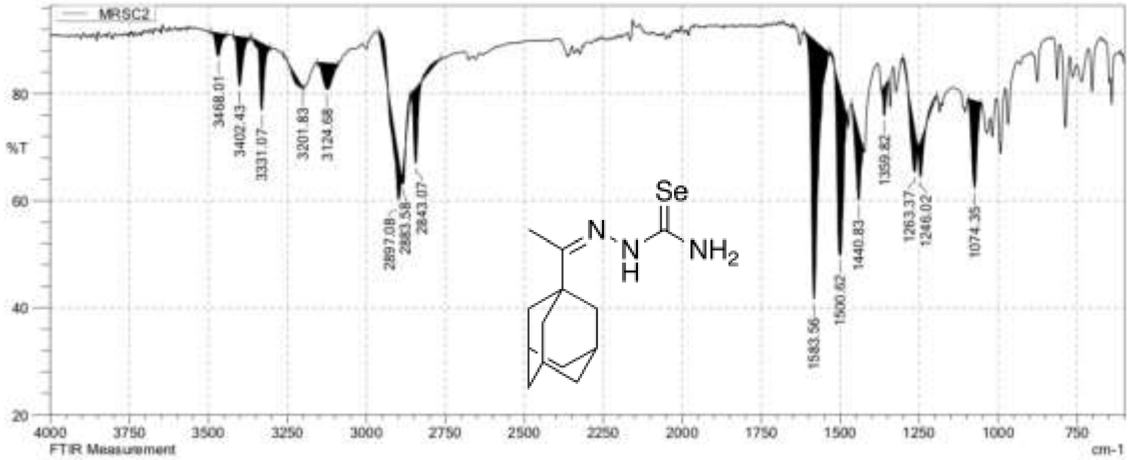

Figure S87. IR (up) and ^{1}H-NMR (down) of compound ***Se*O11**.

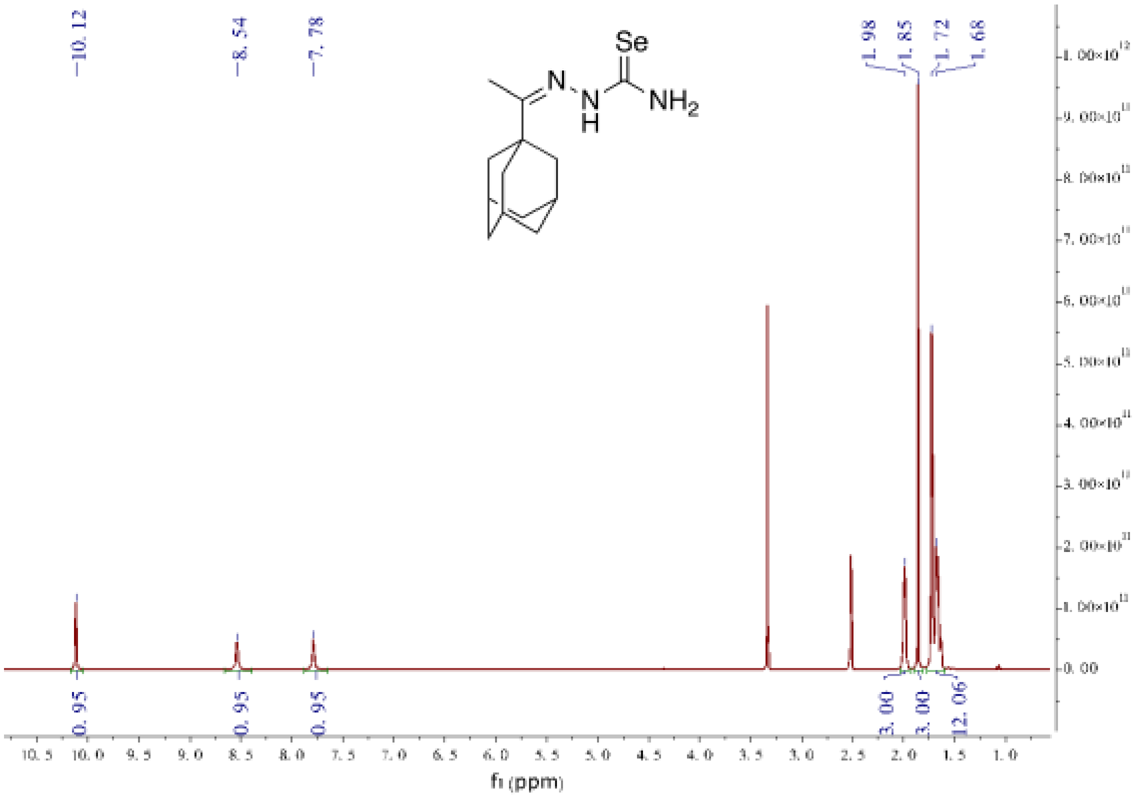

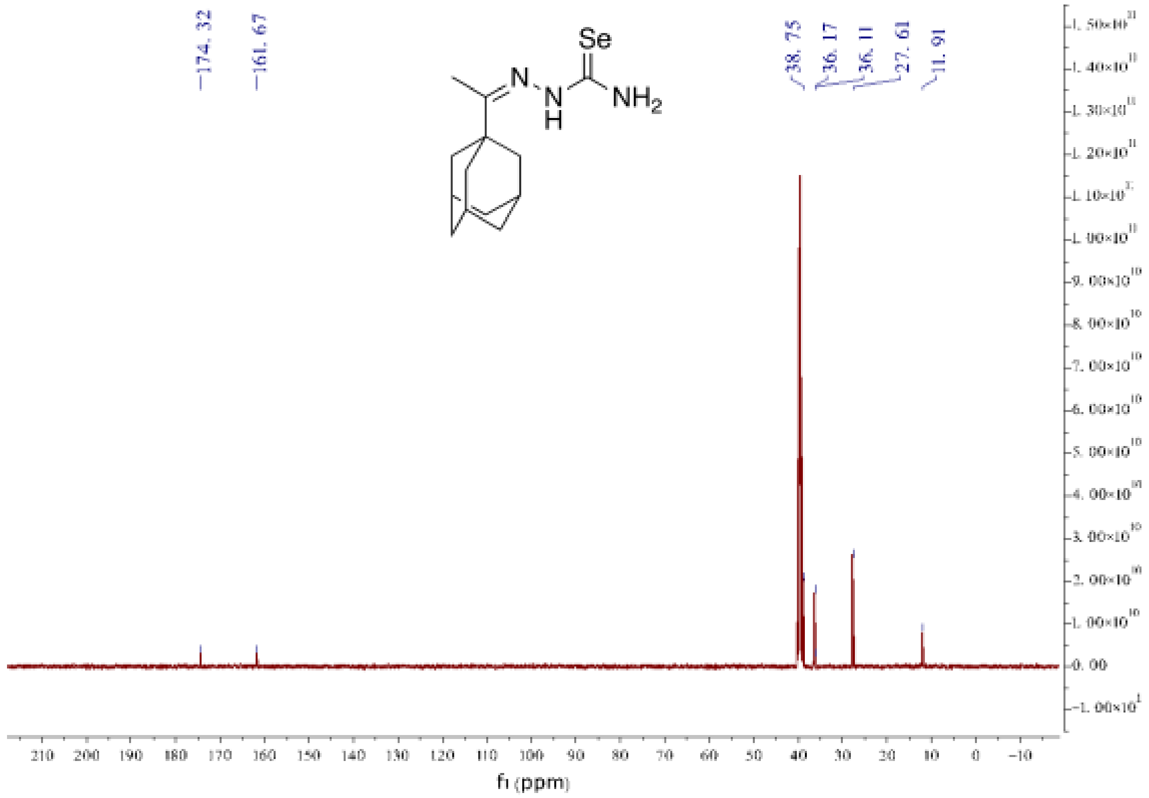

Figure S88. ^{13}C-NMR (up) and ^{77}Se-NMR (down) of compound ***Se*O11**.

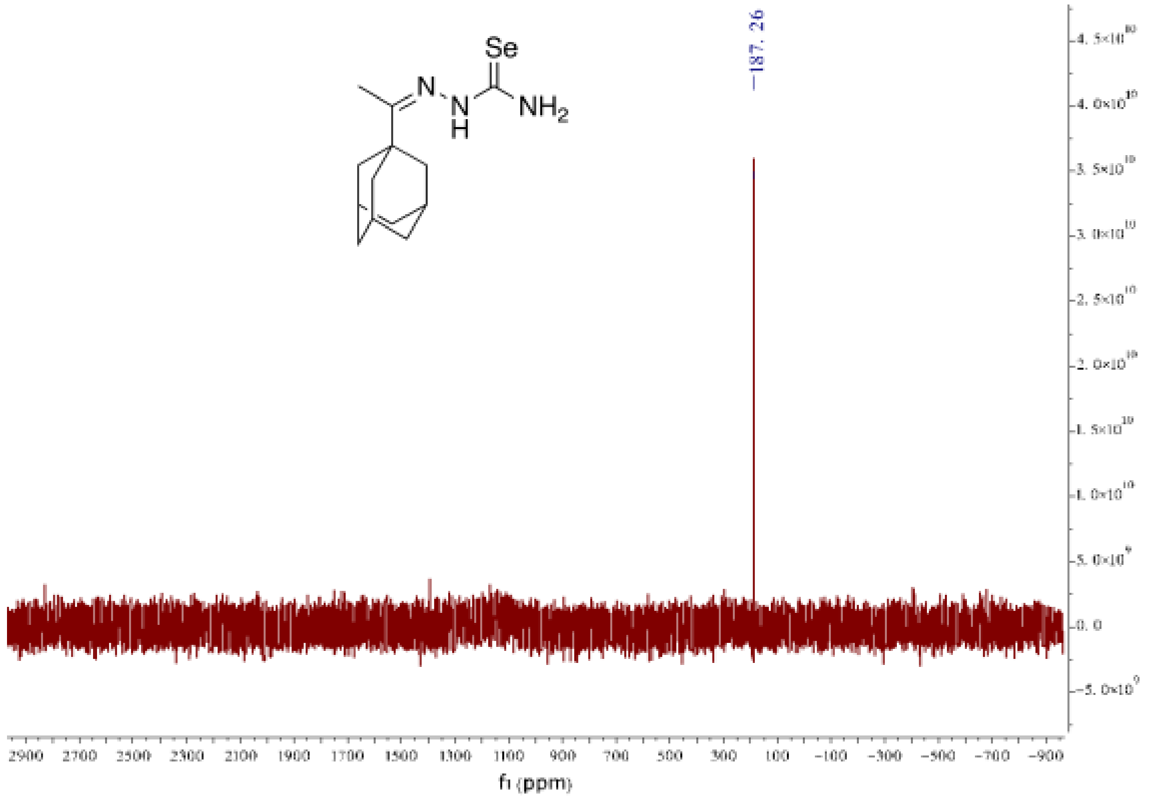

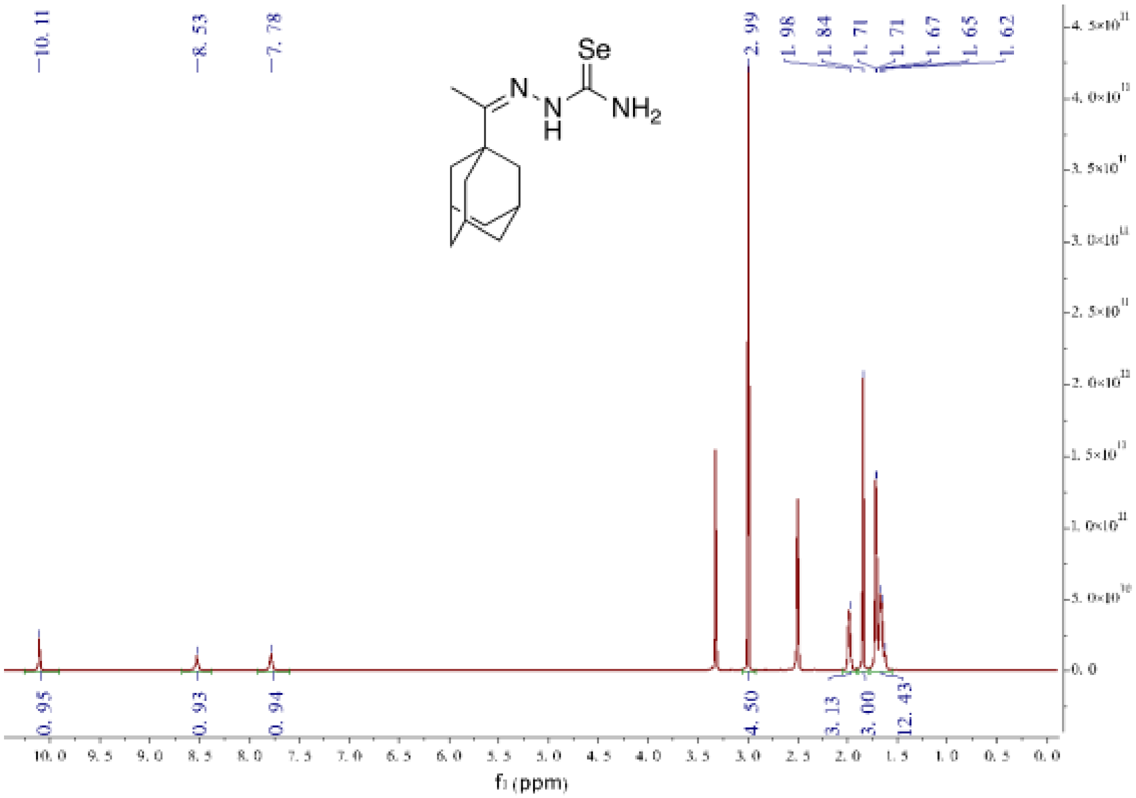

Figure S89. qNMR (up) of compound ***Se*C11** and IR (down) of compound ***Se*O11**.

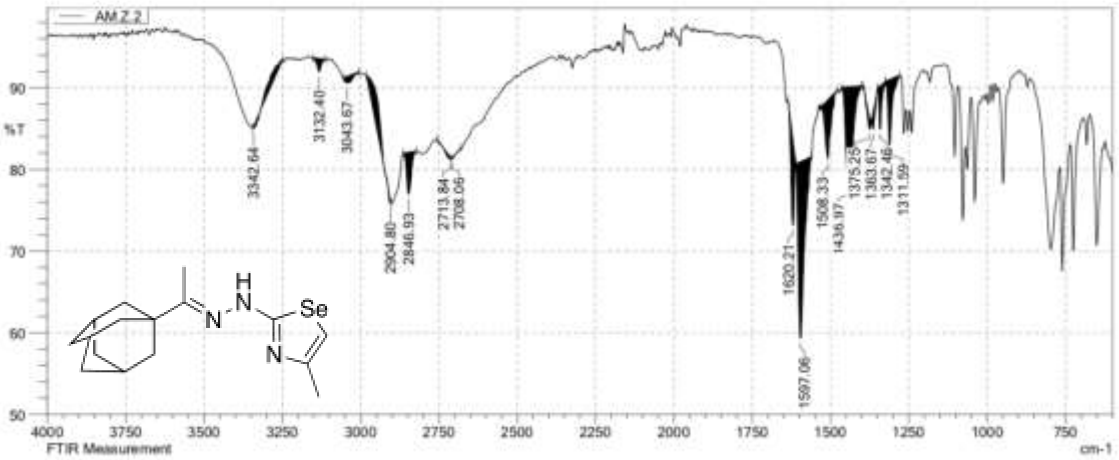

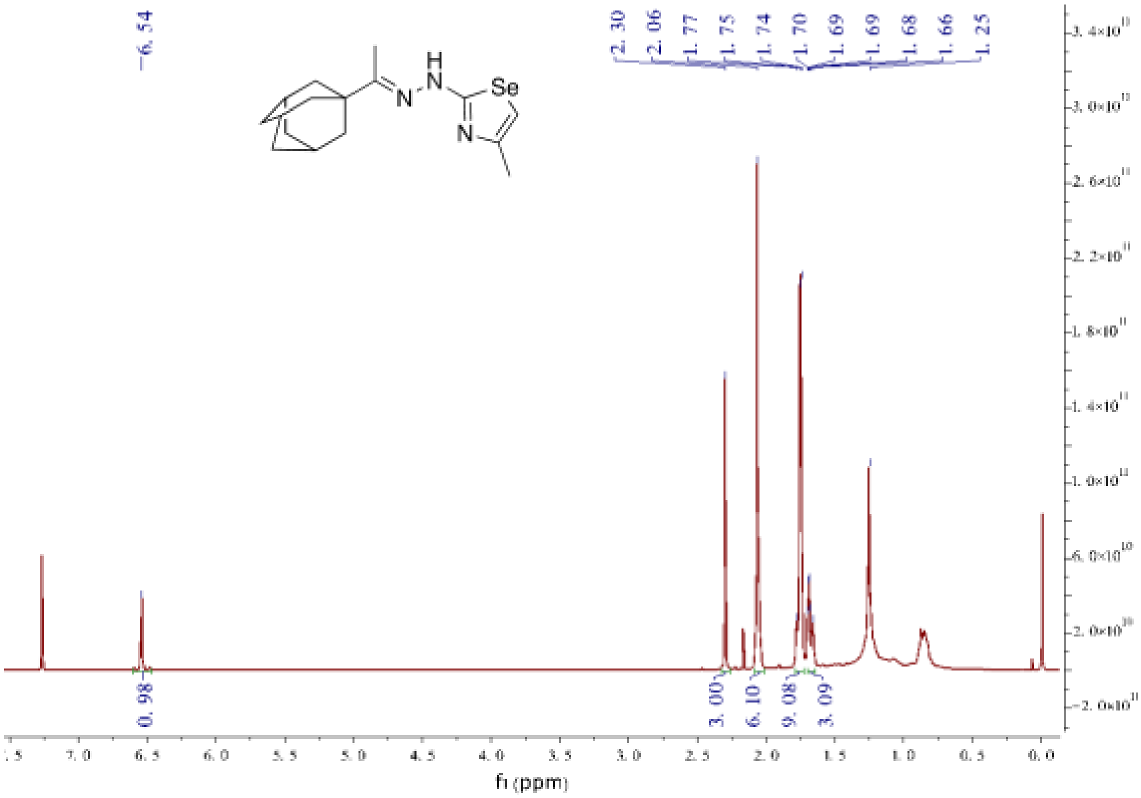

Figure S90. ^{1}H-NMR (up) and ^{13}C-NMR (down) of compound ***Se*C11**.

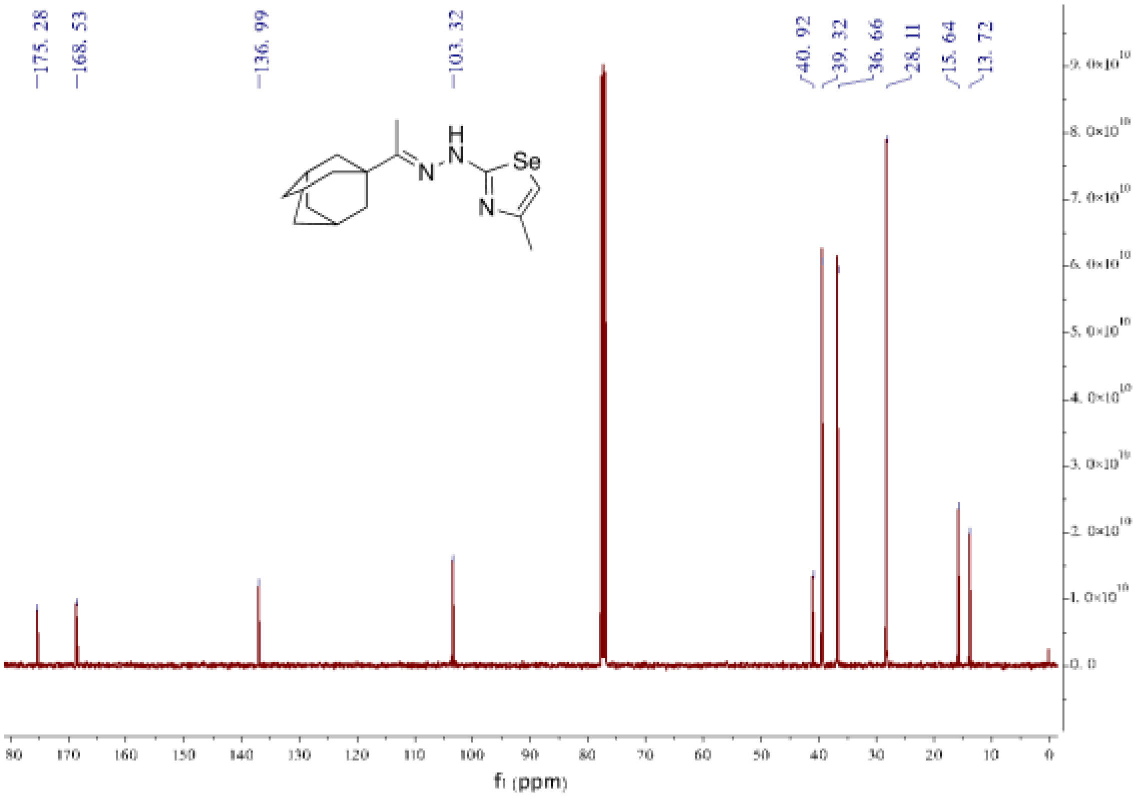

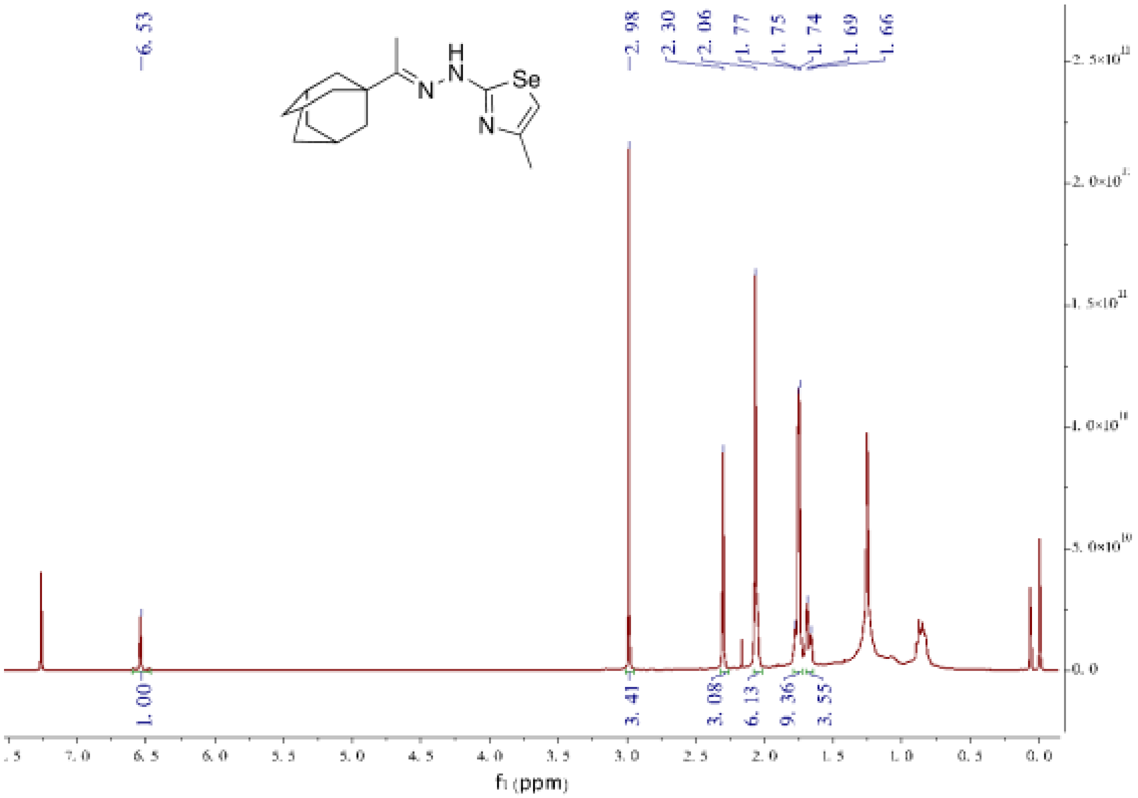

Figure S91. qNMR (up) of compound ***Se*C11** and IR (down) of compound **SO11**.

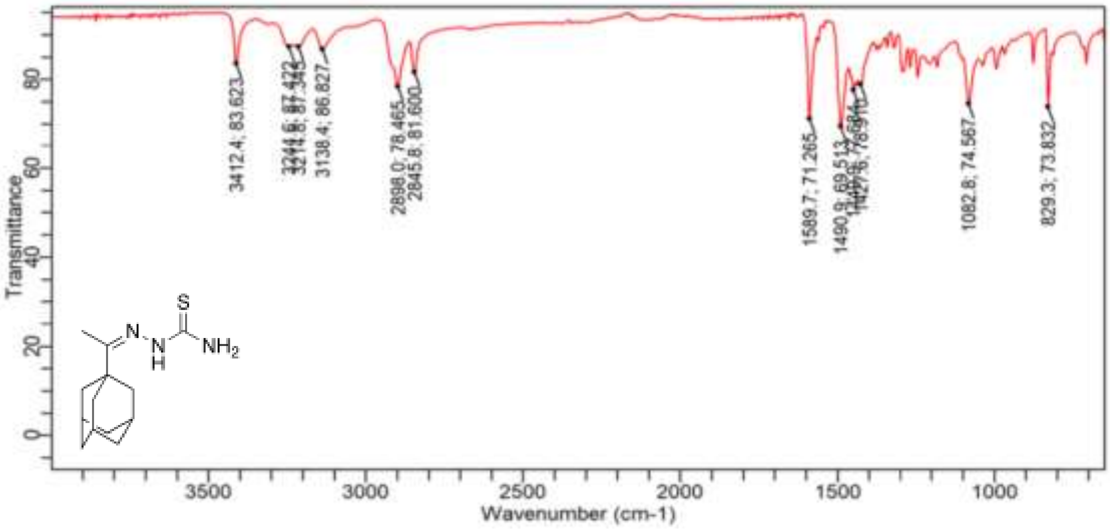

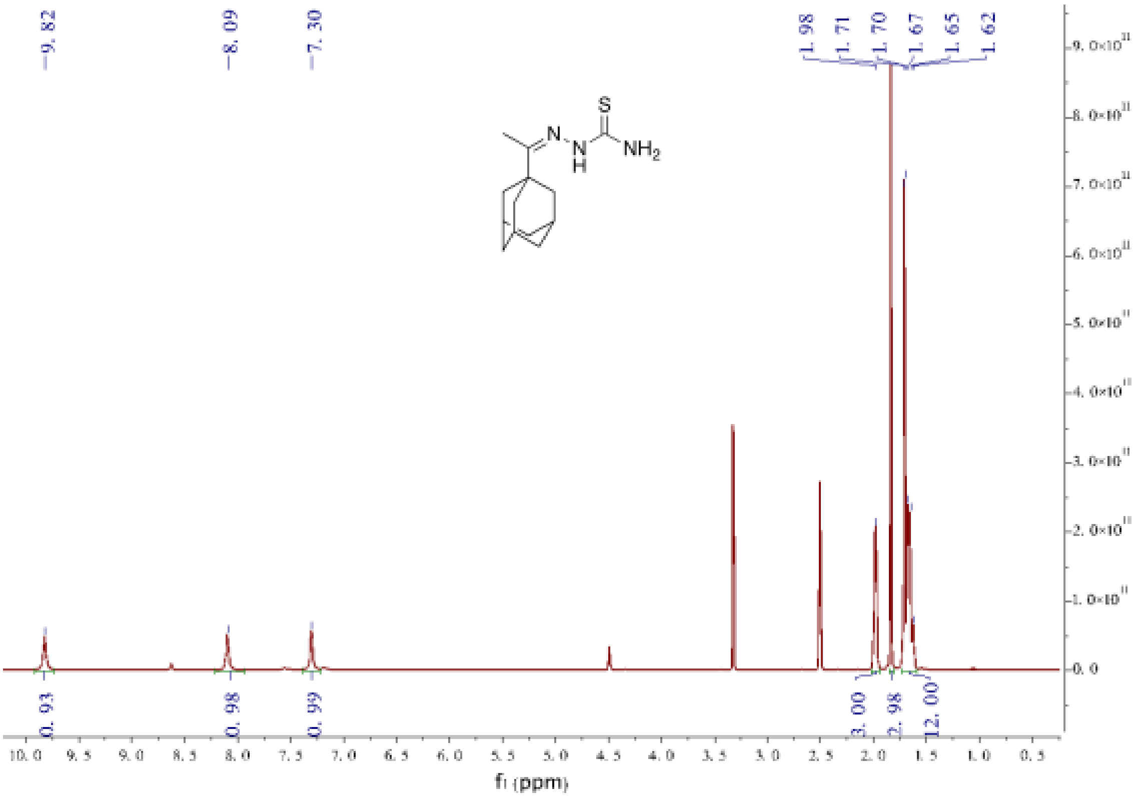

Figure S92. ^{1}H-NMR (up) and ^{13}C-NMR (down) of compound **SO11**.

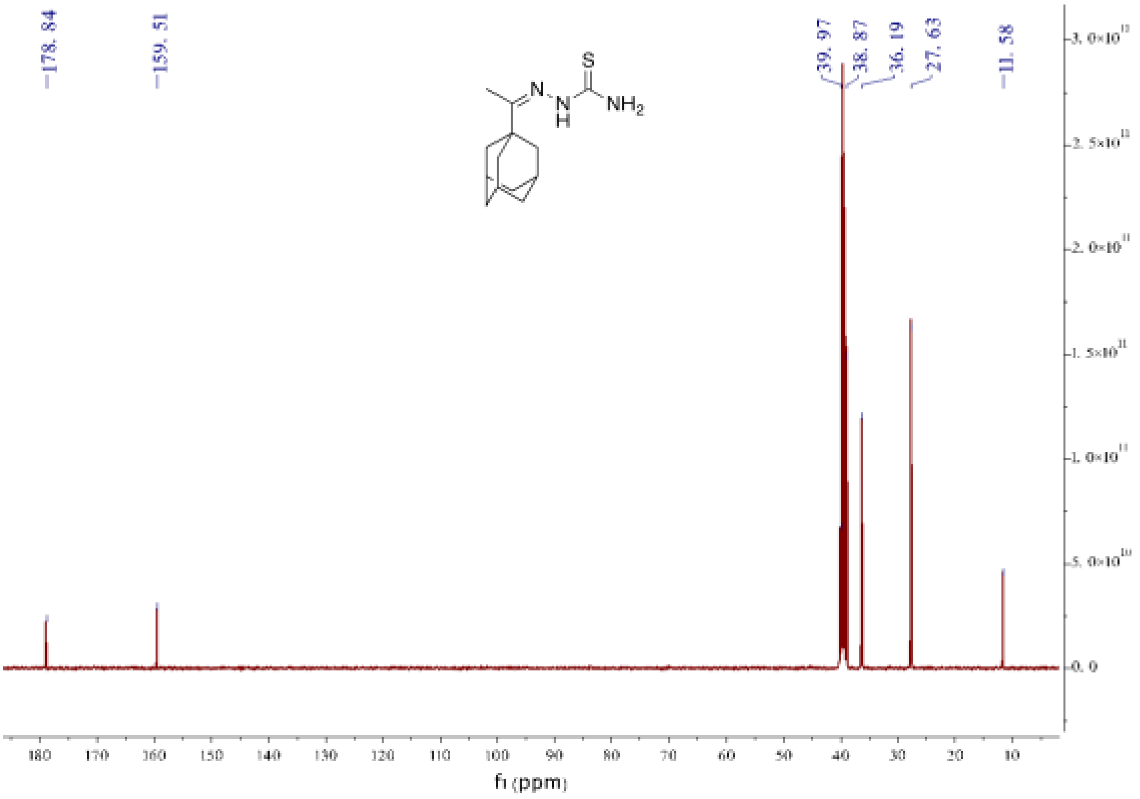

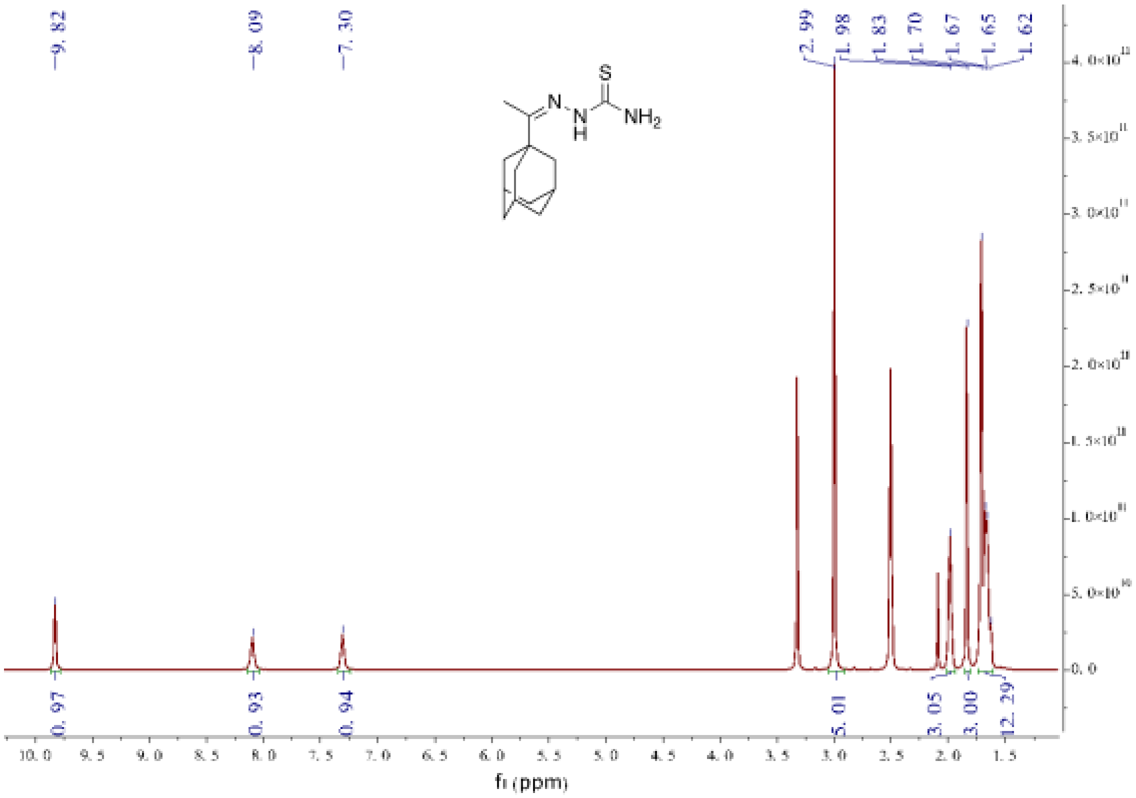

Figure S93. qNMR (up) of compound **SO11** and IR (down) of compound ***S*C11**.

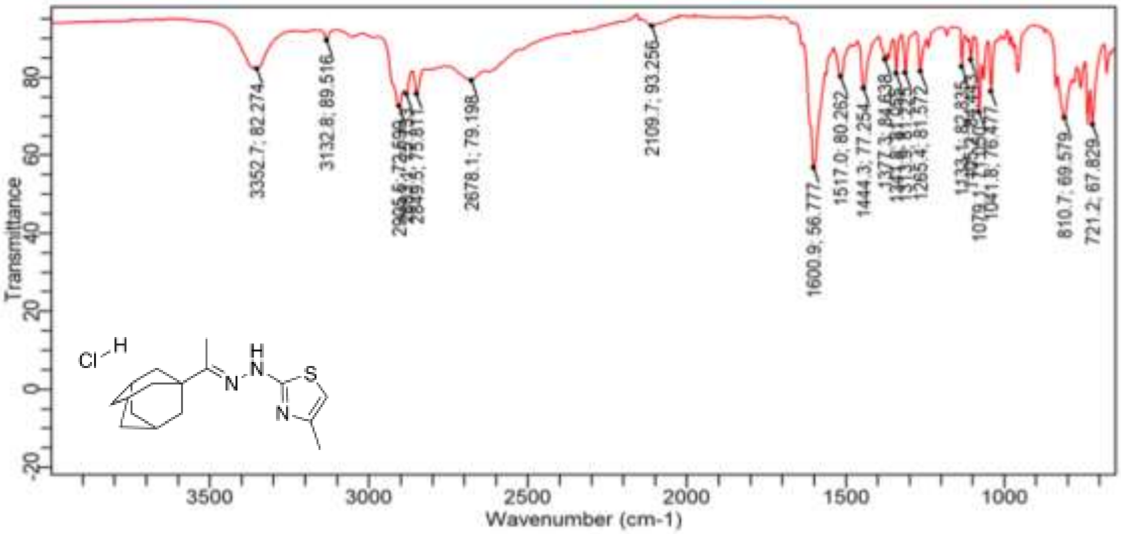

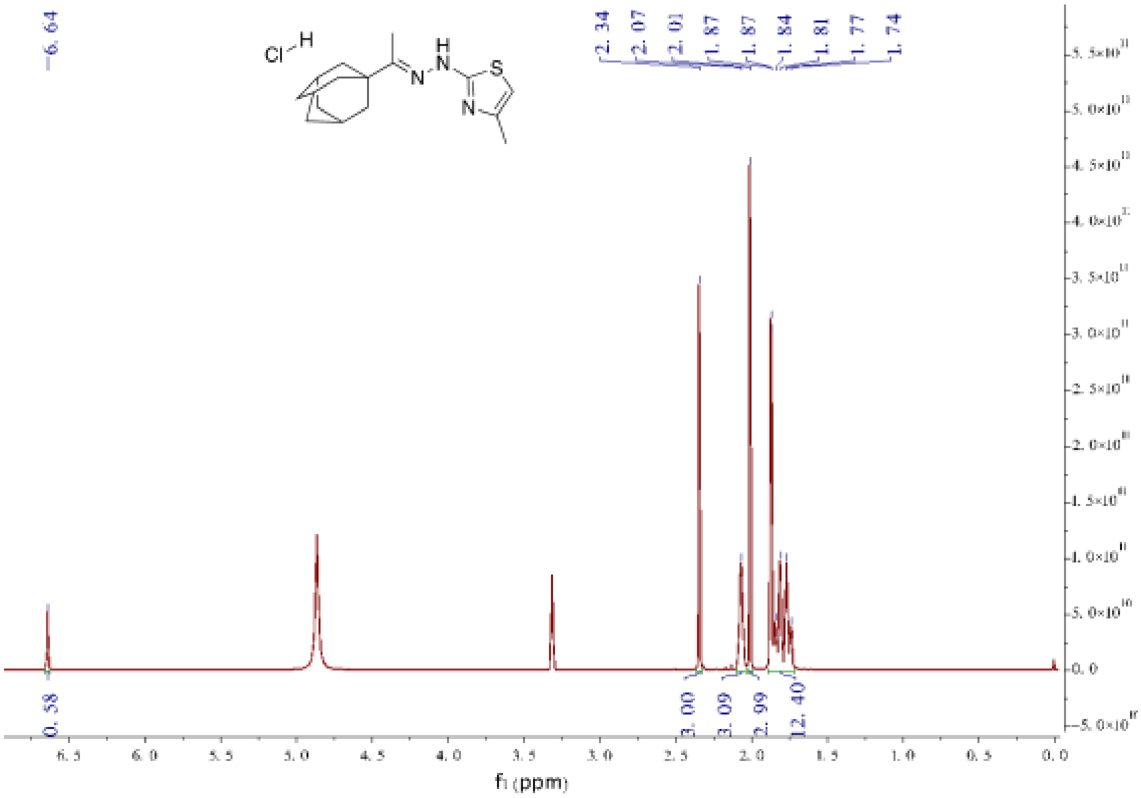

Figure S94. ^{1}H-NMR (up) and ^{13}C-NMR (down) of compound ***S*C11**.

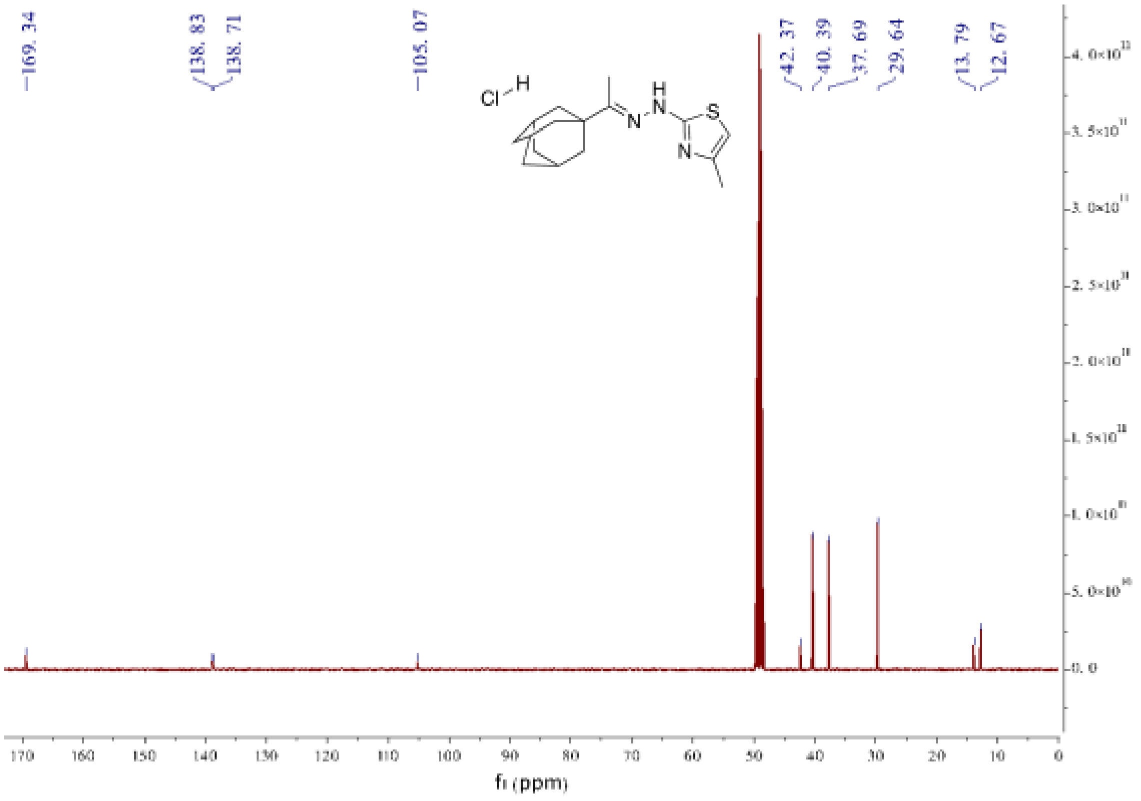

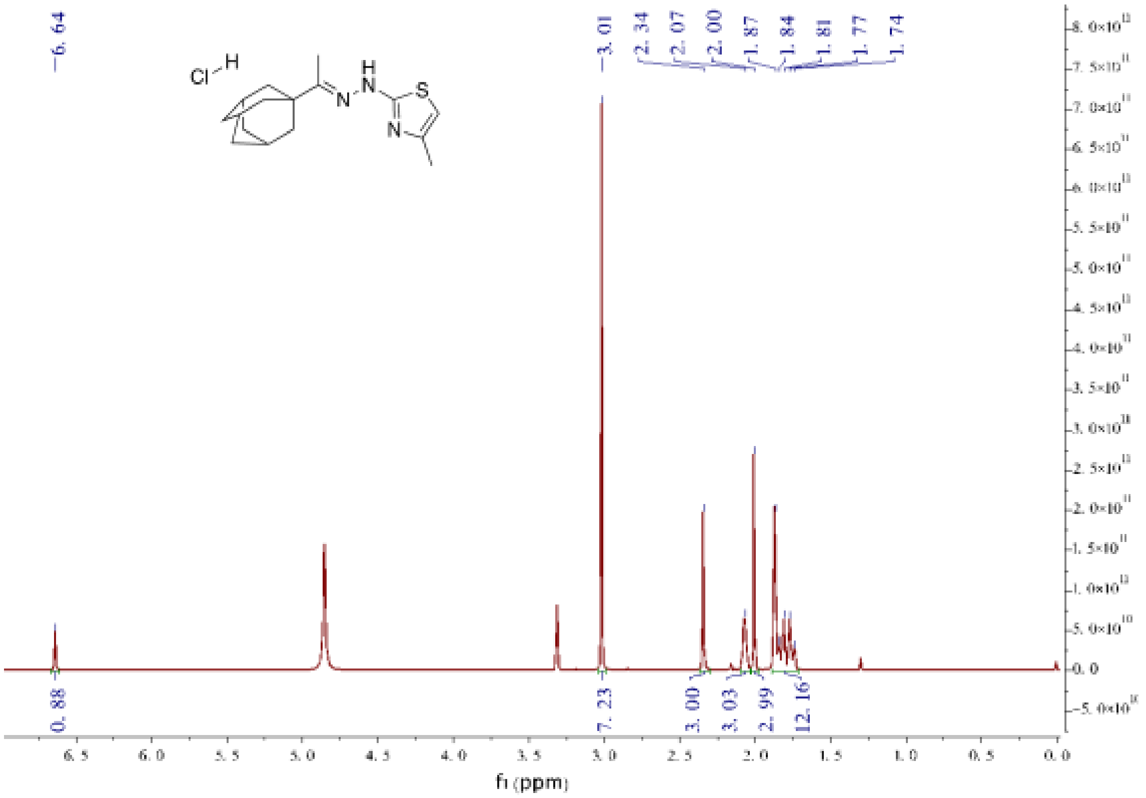

Figure S95. qNMR (up) of compound ***S*C11**.

7. Bidimensional spectra of compounds SO7 and SeO7

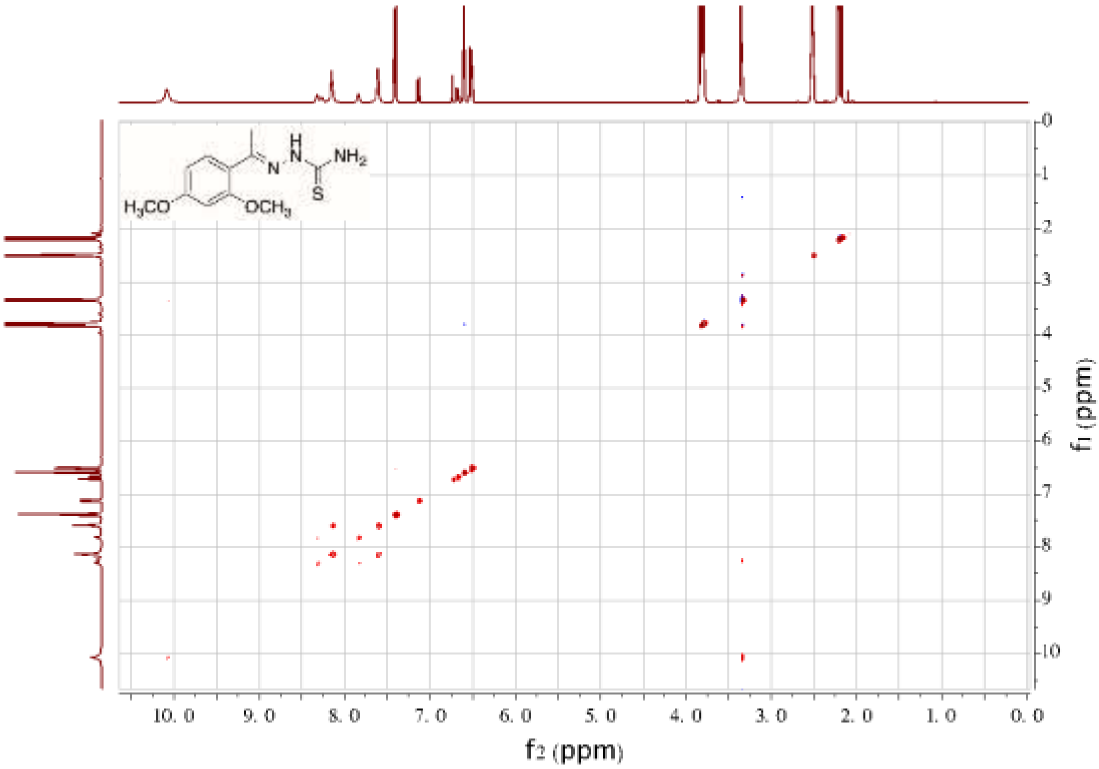

Figure S96. NOESY (up) and HMBC (down) of compound ***SO7***.

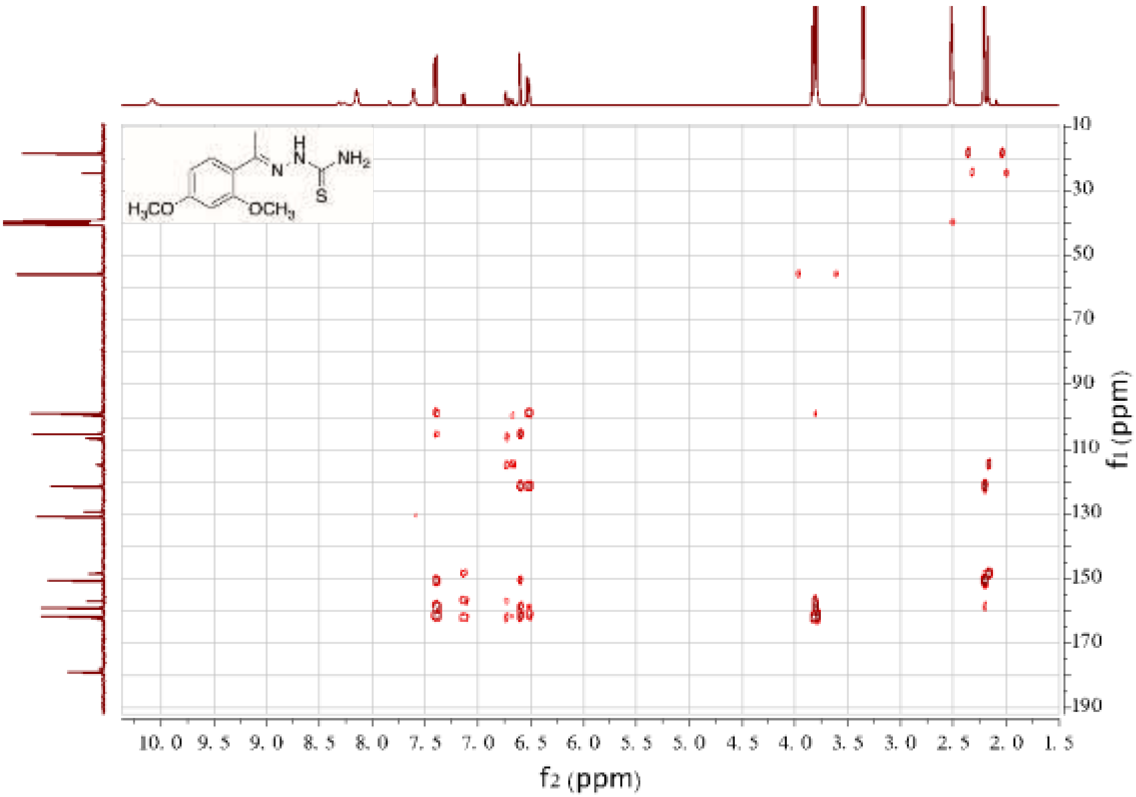

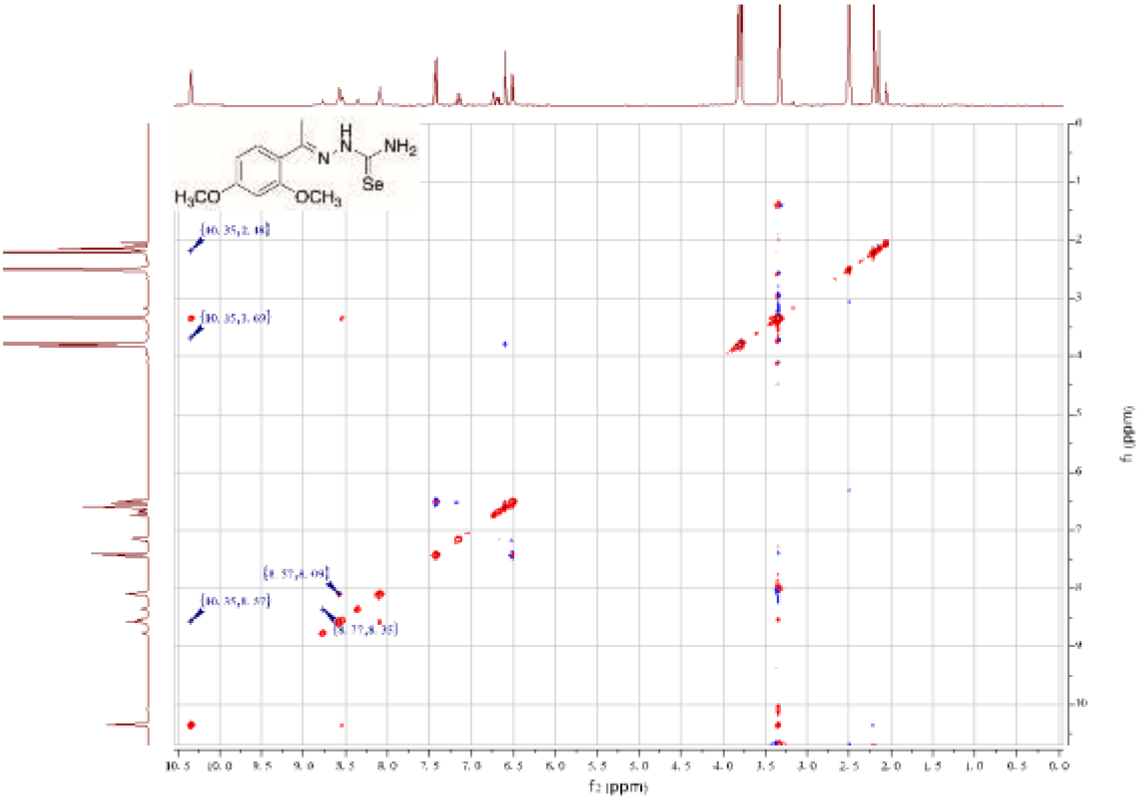

Figure S97. NOESY (up) and HMQC (down) of compound ***Se*O7**.

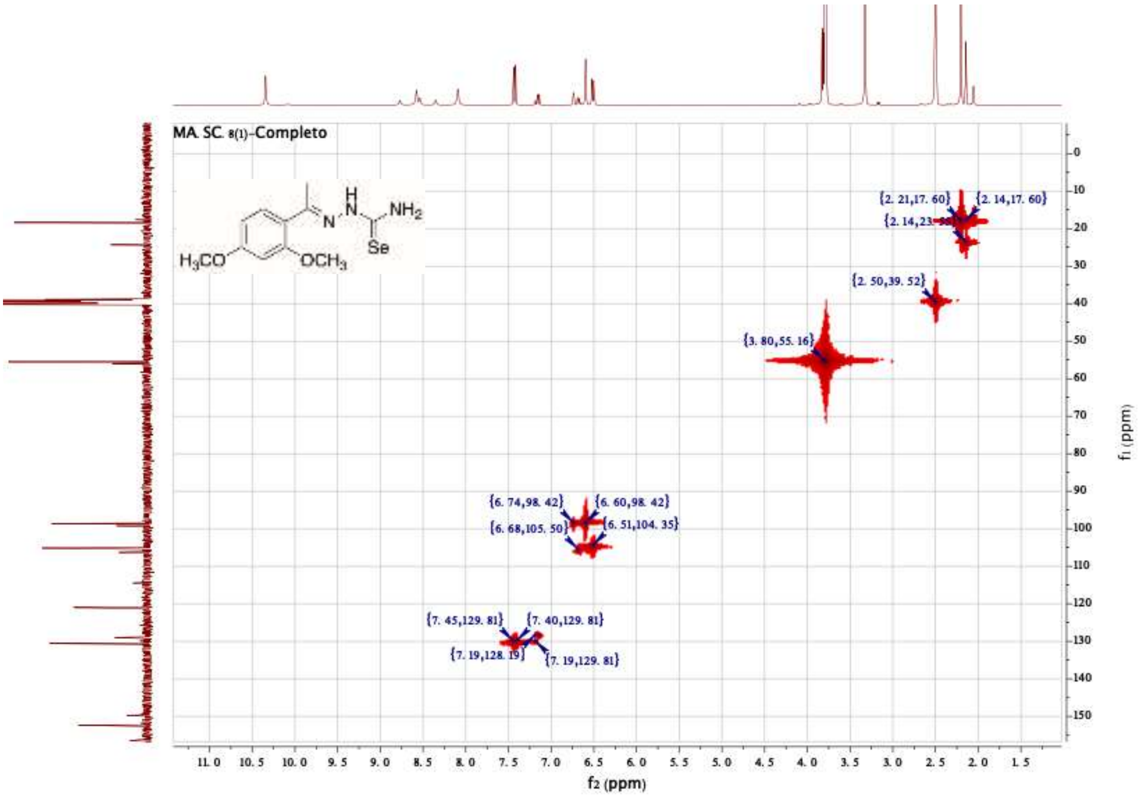

8. Comparative SARs for inhibition of *T. brucei* and *T. cruzi* growth, and the inhibition of their respective proteases, TbrCATL and Cz

Table S5. Comparative SARs for inhibition of *T. brucei* and *T. cruzi* growth, and the inhibition of their respective proteases, *Tbr*CATL and Cz

	Activity (EC_{50}, µM)		Toxicity (CC_{50}, µM)		Antioxidant activity	Protease inhibition (IC_{50}, nM)		Mechanism of binding
	T. cruzi CAI/72[*,a]	*T. brucei* Lister 427[b]	C2C12 cells[*,c]	HEK293 cells[d]	% inhibition of DPPH at 0.03 mg/ml (2 h)[e]	Cz[*,f]	*Tbr*CATL[g]	
***Se*O3**	0.77±0.17	0.47±0.02	>10	2.82±0.11	75.00±2.65	14.31±3.57	9.87±0.63	Covalent bond
***Se*C3**	NT	6.04±0.72	>10	5.90±1.22	71.00±3.06	NT	201.65±0.92	Aromatic ring interactions
***Se*O5**	NT	5.38±0.72	>10	2.29±0.26	63.33±6.89	0.91±0.18	0.51±0.08	Aromatic ring interactions
***Se*C5**	0.31±0.18	10.53±1.02	>10	9.00±1.36	78.00±1.73	8.99±5.56	174.70±43.70	Aromatic ring interactions

[*]Results for *T. cruzi* and Cz can be found in Rubio-Hernández, M. *et al.*, 2024. Ref [40].

[a]EC_{50} for *T. cruzi* is expressed as the average of three independent experiments, each performed in triplicate ± SD (n=9). [b]EC_{50} for *T. brucei* is expressed as the average of three independent experiments, each performed in duplicate ± SD (n=6). [c]CC_{50} for C2C12 cells is the concentration used for calculating cell-viability. [d]CC_{50} for HEK293 cells is the average of three independent experiments, each performed in triplicate (n=9). [e] % inhibition of DPPH was calculated at 0.03 mg/ml after 2 h. This value is expressed as the mean ± SEM of three independent experiments, each performed in triplicate (n=9). [f] IC_{50} for Cz is shown as the mean ± SEM of two different experiments, each performed in triplicate (n=6). [g] IC_{50} for *Tbr*CATL is shown as the mean ± SD of two different experiments, each performed in triplicate (n=6).
